# Interim results from a phase I randomized, placebo-controlled trial of novel SARS-CoV-2 beta variant receptor-binding domain recombinant protein and mRNA vaccines as a 4th dose booster

**DOI:** 10.1016/j.ebiom.2023.104878

**Published:** 2023-11-27

**Authors:** Terry M. Nolan, Georgia Deliyannis, Maryanne Griffith, Sabine Braat, Lilith F. Allen, Jennifer Audsley, Amy W. Chung, Marcin Ciula, Nicholas A. Gherardin, Michelle L. Giles, Tom P. Gordon, Samantha L. Grimley, Lana Horng, David C. Jackson, Jennifer A. Juno, Katherine Kedzierska, Stephen J. Kent, Sharon R. Lewin, Mason Littlejohn, Hayley A. McQuilten, Francesca L. Mordant, Thi H.O. Nguyen, Vanessa Pac Soo, Briony Price, Damian F.J. Purcell, Pradhipa Ramanathan, Samuel J. Redmond, Steven Rockman, Zheng Ruan, Joseph Sasadeusz, Julie A. Simpson, Kanta Subbarao, Stewart A. Fabb, Thomas J. Payne, Asuka Takanashi, Chee Wah Tan, Joseph Torresi, Jing Jing Wang, Lin-Fa Wang, Hareth Al-Wassiti, Chinn Yi Wong, Sophie Zaloumis, Colin W. Pouton, Dale I. Godfrey

**Affiliations:** a Department of Infectious Diseases, University of Melbourne at the Peter Doherty Institute for Infection & Immunity, Melbourne, Australia; b Murdoch Children's Research Institute, Melbourne, Australia; c Department of Microbiology & Immunology, University of Melbourne at the Peter Doherty Institute for Infection and Immunity, Melbourne, Australia; d Centre for Epidemiology and Biostatistics, Melbourne School of Population and Global Health, University of Melbourne, Melbourne, Australia; e Department of Immunology, Flinders University and SA Pathology, Flinders Medical Centre, Bedford Park, Adelaide, Australia; f Global Station for Zoonosis Control, Global Institution for Collaborative Research and Education (GI-CoRE), Hokkaido University, Sapporo, Japan; g Victorian Infectious Diseases Service, Royal Melbourne Hospital at the Peter Doherty Institute for Infection and Immunity, Melbourne, Australia; h Department of Infectious Diseases, Alfred Hospital and Monash University, Melbourne, Australia; i CSL Seqirus, Vaccine Innovation Unit, Parkville, Melbourne, Australia; j Monash Institute of Pharmaceutical Sciences, Parkville, Australia; k Duke NUS Medical School, Programme for Emerging Infectious Diseases, Singapore; l WHO Collaborating Centre for Reference and Research on Influenza at the Peter Doherty Institute for Infection and Immunity, Australia

**Keywords:** SARS-CoV-2, Vaccine, Receptor binding domain, Recombinant protein, mRNA, Beta variant, Phase I trial

## Abstract

**Background:**

SARS-CoV-2 booster vaccination should ideally enhance protection against variants and minimise immune imprinting. This Phase I trial evaluated two vaccines targeting SARS-CoV-2 beta-variant receptor-binding domain (RBD): a recombinant dimeric RBD-human IgG_1_ F_c_-fusion protein, and an mRNA encoding a membrane-anchored RBD.

**Methods:**

76 healthy adults aged 18–64 y, previously triple vaccinated with licensed SARS-CoV-2 vaccines, were randomised to receive a 4th dose of either an adjuvanted (MF59®, CSL Seqirus) protein vaccine (5, 15 or 45 μg, N = 32), mRNA vaccine (10, 20, or 50 μg, N = 32), or placebo (saline, N = 12) at least 90 days after a 3rd boost vaccination or SARS-CoV-2 infection. Bleeds occurred on days 1 (prior to vaccination), 8, and 29. ClinicalTrials.govNCT05272605.

**Findings:**

No vaccine-related serious or medically-attended adverse events occurred. The protein vaccine reactogenicity was mild, whereas the mRNA vaccine was moderately reactogenic at higher dose levels. Best anti-RBD antibody responses resulted from the higher doses of each vaccine. A similar pattern was seen with live virus neutralisation and surrogate, and pseudovirus neutralisation assays. Breadth of immune response was demonstrated against BA.5 and more recent omicron subvariants (XBB, XBB.1.5 and BQ.1.1). Binding antibody titres for both vaccines were comparable to those of a licensed bivalent mRNA vaccine. Both vaccines enhanced CD4^+^ and CD8^+^ T cell activation.

**Interpretation:**

There were no safety concerns and the reactogenicity profile was mild and similar to licensed SARS-CoV-2 vaccines. Both vaccines showed strong immune boosting against beta, ancestral and omicron strains.

**Funding:**

Australian Government Medical Research Future Fund, and philanthropies Jack Ma Foundation and IFM investors.

**Research in context**

**Evidence before this study**

Early in the COVID-19 pandemic, there were authoritative calls for RBD-based SARS-CoV-2 vaccines that would potentially reduce the risk of vaccine escape and imprinting, and provide a more efficient basis for mass production to meet global vaccine needs.

Recent post-marketing studies of omicron-directed whole Spike bivalent mRNA booster vaccines have shown only modest increases in immune responses to omicron variants compared to ancestral vaccine boosts. It is possible that imprinting is an important attenuating factor, and that it may become progressively more impactful as successive boosts are delivered.

We developed two RBD-based vaccines: a recombinant protein beta variant RBD-Fc vaccine, combined with MF59® adjuvant, and an mRNA-beta variant RBD vaccine delivered in a liponanoparticle solution. Our preclinical studies showed that these vaccines induce strong protection in mice when challenged with both beta and a mouse-tropic ancestral strain. Furthermore, a heterologous third dose booster following immunisation with whole Spike vaccine, induced increased titres of nAb against other variants including alpha, delta, delta+, gamma, lambda, mu, and omicron BA.1, BA.2 and BA.5.

Several other RBD vaccines are in various stages of clinical trials or implementation. Some key examples are: (1) ZF2001 is now approved for emergency use in China and some other countries. This dimeric RBD vaccine has two RBD subunits linked via an engineered single-chain construct and administered with alum adjuvant. (2) A similar dimeric RBD vaccine, combined with tetanus toxoid plus alum, is also approved in Cuba and Iran. (3) An RBD human IgG1-Fc dimer, fused to IFN-α and an MHC class–II binding element, combined with alum, has recently been in phase III clinical trial. (4) An RBD-human IgG_1_ Fc vaccine (ancestral strain) with montanide oil-in-water adjuvant was recently tested in a phase I/II trial. In general, these vaccines appear to be well-tolerated and capable of inducing strong neutralising antibody responses.

None of these recombinant protein vaccine trials has investigated RBD constructs targeting the beta SARS-CoV-2, as a fourth dose heterologous boost, and none has compared protein to mRNA RBD vaccines in the same trial.

**Added value of this study**

Novelty of this study relates to: a 4th dose boost using RBD rather than whole Spike; a beta variant-directed candidate (as opposed to omicron or ancestral); and MF59 (for Protein-RBD, previously studied only in a small subgroup of another RBD Phase I trial—ABNCoV2) and a new lipid nanoparticle (LNP) for mRNA-RBD.

This is one of very few head-to-head placebo-controlled clinical studies of recombinant protein and mRNA COVID vaccines. And also one of the few with a comparison with a licensed ‘gold standard vaccine’ (Moderna). Our results demonstrate strong boosting in a highly immune population, and a remarkable breadth of immune response including against recent omicron sub-variants and against other coronaviruses. No safety signals were observed with either candidate and both exhibited a modest and acceptable reactogenicity profile.

**Implications of all the available evidence**

Our results demonstrate a potentially better approach for boosting than is currently pursued with whole Spike vaccines targeting progressive generations of omicron variants. These vaccines focus the immune response to the RBD, the primary target for neutralising antibodies, while simultaneously avoiding imprinted responses against non-RBD Spike epitopes. As they appear to be capable of augmenting immune responses against a wide range of variants, there is a strong case to proceed to a Phase 2 study for both candidates. There is definite potential for both protein and mRNA combination vaccines (e.g. COVID with influenza, RSV, etc), because of the reduced ‘payload’ required for the COVID component (compared to whole Spike).

## Introduction

Severe acute respiratory syndrome coronavirus 2 (SARS-CoV-2), the cause of coronavirus disease 2019 (COVID-19), has created an enormous global health crisis, with more than 767 million confirmed cases and more than 6.9 million deaths.^1^

Although effective COVID-19 vaccines have been rapidly developed and deployed, a succession of variants of concern (VOC) have emerged since mid 2020. The beta VOC was first detected in South Africa in late 2020^2^ where it was shown in a clinical trial to substantially evade ChAdOx-1 vaccine protection.^3^ This variant was therefore the first highly immuno-evasive strain, largely as a result of three mutations at receptor-binding domain (RBD) sites K417N, E484K and N501Y.^2^ Other variants soon replaced beta, and most recently, the omicron variant, first identified in Gauteng province of South Africa in late 2021,^4^ has spread rapidly with multiple subvariants that have evaded vaccine- and/or infection-induced, and monoclonal antibody-mediated neutralisation. Of note, while the omicron RBD is more extensively mutated than beta, they share the K417N and N501Y mutations and while E484 is also mutated, in the case of omicron, it is E484A. Recent studies revealed that ancestral mRNA vaccine effectiveness during the omicron BA.4/5 waves was below 50% after two or three doses, with a fourth dose yielding only a modest increase in omicron-neutralising antibodies (nAb),^5^ while still maintaining a relatively high level of protection against severe SARS-CoV-2 infection and death. Interestingly, prior infection with the beta VOC,^6^ or immunisation with beta Spike protein-containing bivalent mRNA vaccine,^7^^,^^8^ provided enhanced nAb responses against more recent variants.

In addition to the emergence of vaccine-resistant variants, many challenges remain, including: vaccine stability during storage and transport and access for developing countries, waning vaccine-induced immunity, and concerns about rare, yet serious, adverse events associated with existing Spike-based vaccines. Immunological imprinting also seems to be limiting the impact of new variant-targeting bivalent Spike vaccines,^9^ possibly because most epitopes in Spike are non-neutralising.^10^ While RBD vaccines do not necessarily escape immunological imprinting, antibodies boosted by epitopes conserved between the RBD vaccine and prior vaccines or infections are more likely to be neutralising because >90% nAb target the RBD rather than other parts of the Spike.^11^^,^^12^ Accordingly, while other regions of Spike are also immunogenic and some can stimulate nAb, these account for <10% of the total nAb response, which is a salient point, considering that nAb strongly correlate with vaccine efficacy.^13^ Additionally, as RBD-based vaccines eliminate most of the Spike protein, this may reduce the risk of adverse events including myocarditis and amyloidosis.^14^^,^^15^

We have developed two vaccine candidates based on the SARS-CoV-2 beta variant RBD: an adjuvanted recombinant protein, and a nucleoside-modified mRNA delivered in lipid nanoparticles. The beta variant was selected because at the time we initiated production, this was the most immuno-evasive variant. This was fortuitous because, as mentioned above, beta variant infection and vaccines appear to drive superior cross reactivity compared to the ancestral strain.^7^^,^^8^ The protein (Protein-RBD) is generated as an Fc-fusion dimer to enhance immunogenicity by engaging Fc receptor^+^ antigen-presenting cells. The mRNA candidate (mRNA-RBD) is expressed with a transmembrane domain and cytoplasmic tail.

Our preclinical studies showed that the beta RBD protein vaccine as a third injection provided a stronger immune boost for generating RBD-targeting antibodies, including nAb, compared to a third ancestral or beta variant whole Spike vaccine boost.^16^ This boosted immunity was increased for the ancestral strain and other VOC including, alpha, gamma, delta, kappa and omicron. The membrane-anchored beta mRNA-RBD was chosen, rather than secreted RBD, so that presentation of the translated protein would mimic that of the successful whole Spike mRNA vaccines. Preclinical studies of our mRNA-RBD vaccine showed that when compared by molar dose of RBD mRNA, the immunogenicity of the membrane-anchored RBD was equivalent to whole Spike vaccines. In a dose comparison by units of mass of mRNA, membrane-anchored RBD was approximately four-fold more potent in inducing RBD-specific antibodies (pre-print details will be provided).

## Methods

We conducted a phase 1, randomised, double-blind, placebo-controlled, dose-escalation study to determine the safety and immunogenicity of single booster doses of a SARS-CoV-2 beta variant RBD recombinant protein vaccine adjuvanted with MF59® (Seqirus, Inc) (Protein-RBD), and a SARS-CoV-2 beta variant membrane-anchored RBD mRNA vaccine encapsulated within lipid nanoparticles (mRNA-RBD). The principal endpoints related to safety were serious adverse events (SAEs, Day 1–29), medically attended adverse events (MAAEs) and any adverse events (AEs) leading to study withdrawal at any time during the study, solicited local and systemic reactogenicity within 7 days after vaccination, and unsolicited AEs from Day 1 to Day 29, and to immunogenicity as measured by SARS-CoV-2 neutralising or RBD-specific antibody titres compared to baseline. Proportion of subjects with 4-fold or greater post-vaccination antibody responses was initially conceived as a principal outcome for a priming 2-dose series in non-immune (vaccine naïve and previously uninfected) participants, but this was considered inappropriate for a 4th dose booster criterion.

### Trial design and participants

Eligible participants were healthy adults 18–64 years of age previously vaccinated with 2 primary course doses of *Comirnaty* [(BNT162b2 mRNA, tozinameran], Pfizer) or *Vaxzevria* (ChAdOx1-S, AstraZeneca] and a third booster dose of either *Comirnaty* or *Spikevax* (elasomeran, Moderna). Participants were not screened for past or current SARS-CoV-2 infections by serology or PCR prior to enrolment. Those with a self-reported history of SARS-CoV-2 infection (confirmed by PCR or rapid antigen test) within the previous 90 days were excluded.

### Randomisation and masking

**Fig. 1:**
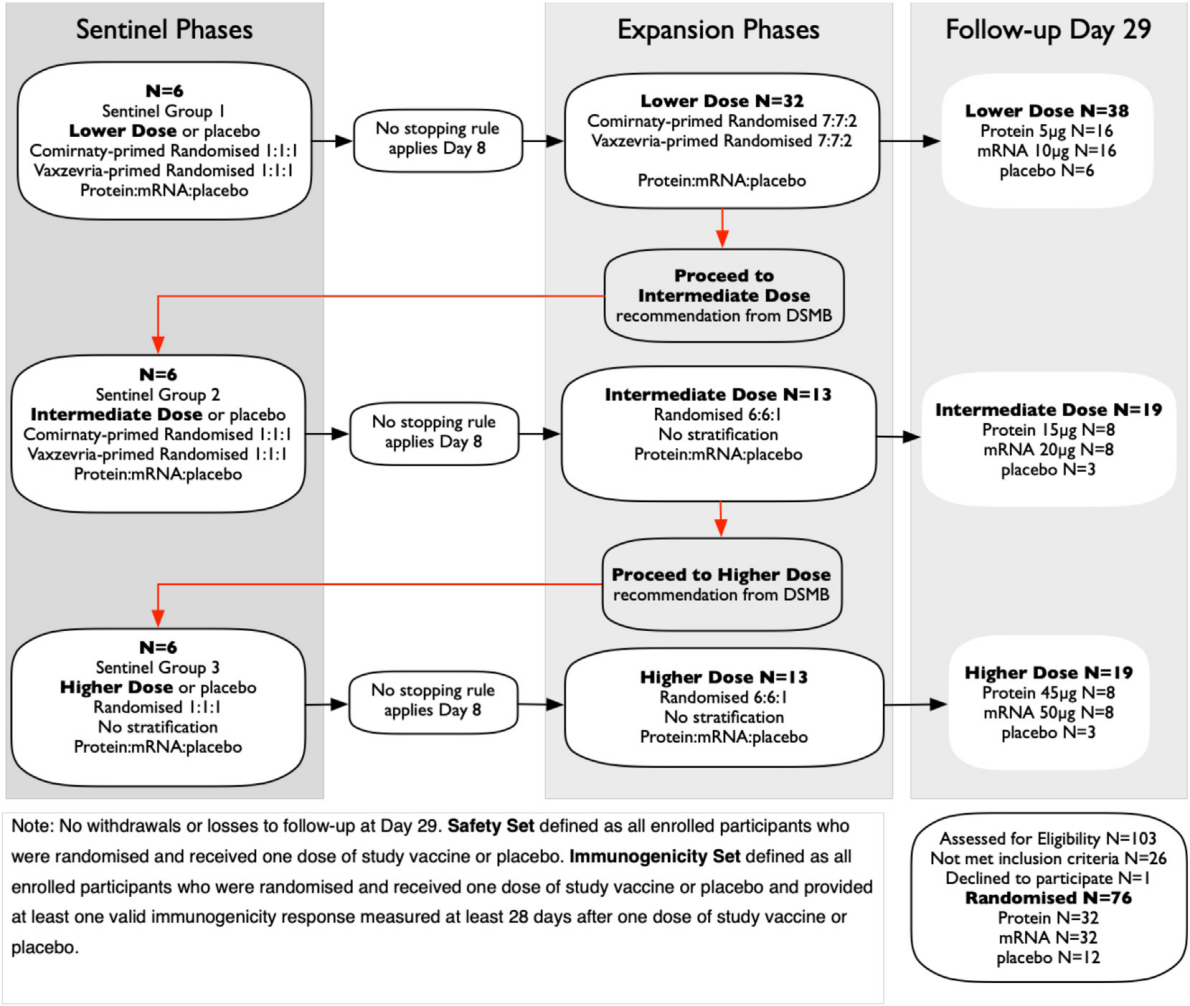
Trial profile.

Participants were randomised via an Interactive Web Response System in accordance with the randomisation list prepared by an independent statistician. Randomisation was stratified by prior COVID-19 primary vaccination with *Comirnaty* or *Vaxzevria* in the lower dose cohort, but not thereafter. Study participants, study site personnel (including the primary outcome assessors), the sponsor, the sponsor's delegated data management vendor and biostatisticians responsible for analysis and reporting of data were blinded to study group allocation. An unblinded dosing team, not involved with the study participant's evaluation prepared and administered the study vaccine/placebo doses. Due to subtle visual differences between the investigational study vaccines, study vaccine administration was performed in a closed area to ensure that other blinded site personnel were not unblinded. The study proceeded in a stepwise dose-escalation manner with 6 sentinel participants randomised in blocks of 1:1:1 ratio to receive a single 0.5 mL intramuscular dose of either Protein-RBD or mRNA-RBD vaccines at each of lower, intermediate or higher doses (protein 5, 15, 45 μg, mRNA 10, 20, 50 μg), or saline placebo (Fig. 1). Following an internal safety monitoring committee positive review of the sentinels' safety data at day 8, further participants were enrolled into each dose cohort randomised in blocks of 7:7:2 ratio of either Protein-RBD or mRNA-RBD vaccines or placebo for the lower dose cohort, and 6:6:1 ratio of either Protein-RBD or mRNA-RBD vaccines or placebo for intermediate and higher dose cohorts. Progression from the lower dose cohort to the intermediate dose cohort, and thereafter to the higher dose cohort occurred after data safety monitoring board (DSMB) review and approval of the 7-day post-vaccination safety data for all participants in each cohort.

### Trial conduct and reference

The study was conducted at the Doherty Institute for Infection and Immunity in Melbourne, and at its partner Royal Melbourne Hospital, and was approved by the Royal Melbourne Hospital Human Research Ethics Committee.

As an external reference, pre- and post-4th dose booster serum samples were evaluated from healthy adults boosted with Moderna Spikevax Omicron BA.1 bivalent vaccine as a subset of participants enrolled in another COVID booster clinical trial registered with The Australian/New Zealand clinical trials registry (ACTRN12622000411741) (https://www.anzctr.org.au/Trial/Registration/TrialReview.aspx?id=383572&isReview=true). These samples were separately assayed in our labs.

### Trial procedures

Once informed consent was provided, an eligibility assessment was performed for all study participants during a 14-day screening period. Screening assessments included the collection of significant medical history data, baseline safety laboratory assessments (blood and urine) and physical examination. Baseline and follow-up safety bloods included rheumatoid factor by standard commercial assay, and a positive result on this was an exclusion criterion at entry screening. Study vaccines were administered as a 0.5 mL intramuscular deltoid injection on day 1. Post-vaccination follow-up visits were planned for days 8, 29, 91 and 181. Participants recorded the frequency and severity of any solicited local and systemic adverse events in an electronic diary for 7 days after vaccination. Adverse events were graded according to the scale provided in Supplementary Table S1. Unsolicited adverse events and the use of any new medications were documented to day 29. Serious adverse events, medically attended adverse events and any adverse events leading to study withdrawal at any time are being collected through to study completion. Blood samples for post-vaccination safety laboratory assessments were collected on day 8 (sentinels only), 29 and day 181. Post-vaccination immunogenicity blood samples were collected on days 8, 29, 91, and 181. Here we report the results of an interim analysis up to and including day 29. Participants who experienced any symptoms indicative of COVID-19-like illness during the study, were encouraged to undergo diagnostic testing in accordance with local public health directives to confirm the presence or absence of SARS-CoV-2 infection. Those participants who tested positive (either by RAT or PCR) were asked to consent to the collection of nasopharyngeal samples for virus whole genome sequencing conducted, using standard procedures, by the Doherty Institute.

### Study oversight

The University of Melbourne served as the study sponsor. The study was reviewed and approved by the Royal Melbourne Hospital Human Research Ethics Committee and was conducted under the Clinical Trial Notification (CTN) Scheme (CTN ID: 04968-1) administered by the Australian Therapeutic Goods Administration. All participants provided written informed consent prior to enrolment. The study was conducted at the Royal Melbourne Hospital (initial Lower Dose sentinels N = 6) and the Peter Doherty Institute for Infection and Immunity in Melbourne, Australia (for all remaining participants). The study is registered at www.clinicaltrials.gov (NCT05272605). The study protocol is available in Supplementary Materials.

### Vaccines

#### Beta-RBD-Fc dimer recombinant protein vaccine

The beta-RBD-Fc dimer consisted of a truncated RBD (N334–P527) linked to the Fc-domain of human IgG1 from the core hinge region through to the C-terminal lysine via a GSGSG linker. DNA encoding this sequence was transfected into CHOK1SV GS-KO® cell lines for stable expression using the Lonza GS Xceed® system. Minipools were generated, and lead minipool selected using Lonza's abridged fed-batch shake flask screen at the National Biologics Facility, University of Queensland, Australia. ISO 9001:2015 standard. Beta-RBD-Fc protein was manufactured at the CSIRO tissue culture facility in Melbourne, Australia. The vaccine was formulated with oil-in-water adjuvant MF59® (CSL Seqirus proprietary adjuvant) at the time of injection.

#### Beta-RBD-TM mRNA-LNP vaccine

The mRNA-RBD vaccine consists of a lipid nanoparticle (LNP) dispersion that encapsulates mRNA encoding an engineered form of the RBD of the Spike protein of the SARS-CoV-2 beta variant. The nucleoside modified mRNA (uridines are replaced with N1-methyl-pseudouridine) codes for the RBD linked to the transmembrane domain and the ‘intracellular’ domain of the Spike protein. The intracellular domain remains on the inside of the viral envelope when the viral particles are formed. This mRNA is translated to produce a membrane-anchored RBD, as we wanted the translated protein to be expressed and then presented in a manner analogous to the membrane-anchored presentation of translated whole spike mRNA vaccine. The mRNA is encapsulated in a unique Monash Institute of Pharmaceutical Sciences, Melbourne, Australia (MIPS) LNP formulation, using four lipids all of which have been used in FDA-approved products: DLin-MC3-DMA, cholesterol, DSPC and DMG-PEG 2000. The mRNA drug substance was manufactured by eTheRNA immunotherapies (Niel, Belgium), with the linear DNA template provided by MIPS. Final manufacture and LNP formulation were carried out in collaboration with IDT Australia. The administered vaccine was diluted to the required dose level with an isotonic tromethamine/sucrose buffer solution (IDT Australia Ltd) at the time of vaccination.

#### Assessment of SARS-CoV-2 binding and nAb responses

**Enzyme-linked immunosorbent assay (ELISA)** RBD-specific antibody responses were determined by ELISA using 96-well plates coated with 2 μg/mL SARS-CoV-2 RBD monomers representing the ancestral, beta or omicron BA.5 strains. Ancestral and beta monomers were produced in house as previously described^12^ while BA.5 RBD monomer, which was 29 amino acid residues longer, was purchased from Sino Biological. Serial dilutions of sera were tested, using tetramethylbenzidine substrate, and antibody titres determined as the reciprocal of the highest dilution of serum required to achieve an optical density of 0.3. **Microneutralisation test:** Serial dilutions of sera were incubated with 100 TCID50 (50% tissue culture infectious dose) of SARS-CoV-2 ancestral, beta or omicron BA.5 viruses, and residual virus infectivity was assessed in Vero E6-TMPRSS2 cells. Viral cytopathic effect (CPE) was read 5 days later and dilution of serum that completely prevented CPE in 50% of the wells (ID50) was calculated. **Multiplex surrogate virus neutralisation test (sVNT)** AviTag-biotinylated RBD proteins were coated on MagPlex-Avidin microspheres. A cocktail of RBD-coated beads (600 per antigen) was pre-incubated with serial dilutions of sera before addition of soluble R-phycoerythrin-conjugated human ACE2 protein. After 1 h incubation, wells were washed and ACE2 binding was detected as phycoerythrin-labelled reporter measured as Median Fluorescence Intensity (MFI). Maximal ACE2 binding MFI was determined by the mean of ACE2 only (no inhibitor) controls. Results are expressed as half-maximal inhibitory dilution (ID50). **Pseudovirus neutralisation test (pVNT)** SARS-CoV-2 ancestral, beta, BQ.1.1, XBB.1 and XBB.1.5 full-length Spike-pseudotyped viruses were produced using HEK293T cells transfected with pCAGGS Spike plasmid. At 24 h post transfection, cells were incubated with VSVΔG luc seed virus. For pVNT assay, 3 million relative light units of pseudoviruses were pre-incubated with serially diluted sera, followed by infection of ACE2-stably-expressing A549 cells. At 20–24 h post infection, an equal volume of ONE-Glo luciferase substrate was added and luminescence signal measured and analysed with Gen5 software v3.10. Further details for all SARS-CoV-2 binding and nAb assays are provided in Supplementary Materials.

#### Assessment of SARS-CoV-2 T-cell responses

**T cell stimulation assay activation induced marker (AIM)** assays were performed on thawed PBMCs plated at 1e6 cells/well per stimulation condition. Cells were cultured for 24 h with either ancestral RBD^17^ or beta RBD overlapping 11-mer peptide pools (104 peptides). Cells were washed and stained with CD8-BV605, CD4-BV650, and activation markers (CD137-APC, CD69-PerCPCy5.5, CD134-PE) and cytokines by intracellular staining (ICS), before fixing with 1% paraformaldehyde.^18^, ^19^, ^20^ Cells were acquired on an LSRII Fortessa (Becton Dickinson) and data were analysed using FlowJo v10 (Becton Dickinson). **Cellular activation in whole blood** measuring the kinetics of antibody-secreting cells (ASCs) and activation of T follicular helper (Tfh)/CD8^+^/CD4^+^ T cell subsets were assessed by directly staining whole blood with antibodies for flow cytometry, essentially as described.^21^, ^22^ Further details are provided in Supplementary Materials.

#### Assessment of anti-Fc antibody responses

Anti-human Fc antibodies were measured by an in-house ELISA. Flat-bottomed maxisorp nunc immune plates (Thermofisher, 439,454) were coated with 100 μl of human IgG Fc fragment (Arotec Diagnostics, ATF01) at 2 μg/mL in PBS buffer overnight at 4 °C. Plates are blocked with PBS 1% BSA (Sigma–Aldrich, A3059) and subsequently incubated with human serum (diluted to 1:20 and 1:100) for 2 h at 37 °C. Well-characterised patient sera with IgG, IgA, and IgM anti-Fc antibodies, respectively, were included as positive controls. After washing plates four times with PBS 0.05% Tween 20, anti-human IgG (Fab specific; Sigma-A8542), anti-human IgA (Sigma, A3400) or anti-human IgM (Invitrogen-AH10605) secondary antibodies were used to detect individual anti-human Fc antibody isotypes. Following 1-h incubation with secondary antibodies, plates were washed six times with PBS 0.05% Tween 20 and phosphatase substrate (Sigma, S0942) is added. Optical density at 405 nm is measured by a plate reader (Spectramax Id5) at 30 min of incubation.

### Statistical analysis and sample size determination

Since this is a first-in-human dose-escalation single-dose Phase I study of 2 vaccines, the statistical analysis did not include formal hypothesis testing and therefore the sample size calculation was not based on statistical power. Group sizes were based on regulatory precedent. Accordingly, all analyses were descriptive, and no hypotheses were formally tested. All analyses included all randomised participants who took at least one dose of study vaccine or placebo. Confidence intervals (CIs) were calculated at the two-sided 95% level based on the t-distribution on the log base 10 transformed values, then back transformed (power of 10) on the original scale. Antibody titres were logarithmically transformed (base 10) and the geometric mean fold rise (GMFR) comparing the relative change in the geometric mean level at day 29 post-vaccination (and in some cases at day 8 as well) compared to baseline presented for each vaccine dosing and placebo group. Statistical analyses were performed using Stata version 16.1 and R version 4.2.2.

### Role of the funding source

Principal funding for this Phase I trial was provided by the Australian Government's Medical Research Future Fund (MRFF). Additional funding for lab and facility support was provided by the National Health & Medical Research Council of Australia, and philanthropic funding from the Jack Ma Foundation and IFM. The Melbourne WHO Collaborating Centre for Reference and Research on Influenza is supported by the Australian Govt Dept of Health. Duke-NUS authors were supported by the Singapore National Medical Research Council. None of these funders had any input into, review or influence on the conduct and reporting of this study.

## Results

### Trial population

The study was conducted in Melbourne, recruiting from April to November 2022. 103 adults aged 18–64 years previously vaccinated with 3 doses of licensed SARS-CoV-2 ancestral strain vaccines were screened, resulting in 76 eligible participants, (participant demographics shown in Table 1), who consented and were then randomised according to the dose-escalation study design (Fig. 1). All participants received one dose of study vaccine on day 1. No withdrawals or losses occurred in follow-up at day 29.

**Table 1:** Baseline demographics, prior COVID and vaccination.

	Placebo	Protein-RBD			mRNA-RBD		
	N = 12	5 μg	15 μg	45 μg	10 μg	20 μg	50 μg
		N = 16	N = 8	N = 8	N = 16	N = 8	N = 8
Age years Mean (SD)	44.7 (13.3)	48.8 (13.0)	48.6 (11.6)	31.8 (12.8)	48.1 (10.8)	35.8 (12.5)	40.8 (13.4)
**Sex n (%)**							
Male	5 (42%)	7 (44%)	4 (50%)	3 (38%)	7 (44%)	2 (25%)	4 (50%)
Female	7 (58%)	9 (56%)	4 (50%)	5 (62%)	9 (56%)	6 (75%)	4 (50%)
**Race n (%)**							
White	8 (67%)	12 (75%)	5 (62%)	6 (75%)	10 (62%)	6 (75%)	6 (75%)
Asian	1 (8%)	0	0	2 (25%)	4 (25%)	2 (25%)	2 (25%)
Black	0	0	1 (13%)	0	0	0	0
Other^a^	3 (25%)	4 (25%)	2 (25%)	0	2 (13%)	0	0
Body mass index (kg/m2) Mean (SD)	24.58 (3.67)	26.08 (3.71)	25.48 (4.45)	25.47 (5.42)	24.52 (3.95)	26.19 (2.90)	23.82 (3.17)
**Priming vaccine n (%)**							
ComirnatyTM (BNT162b2 mRNA)	6 (50%)	8 (50%)	5 (62.5%)	4 (50%)	8 (50%)	6 (75%)	4 (50%)
VaxzevriaTM (ChAdOx1-S)	6 (50%)	8 (50%)	3 (37.5%)	4 (50%)	8 (50%)	2 (25%)	4 (50%)
**1st booster vaccine n (%)**							
ComirnatyTM (BNT162b2 mRNA)	5 (42%)	12 (75%)	4 (50%)	7 (88%)	14 (88%)	7 (88%)	3 (37%)
SpikevaxTM (mRNA-1273, Moderna)	7 (58%)	4 (25%)	4 (50%)	1 (12%)	2 (12%)	1 (12%)	5 (63%)
**Previous COVID-19 history n (%)**							
No	12 (100%)	14 (88%)	4 (50%)	2 (25%)	13 (81%)	3 (37%)	0
Yes	0	2 (12%)	4 (50%)	6 (75%)	3 (19%)	5 (63%)	8 (100%)
**Days since 1st booster Mean (SD)**	195.3 (64.8)	159.8 (29.3)	236.5 (38.5)	291.3 (30.0)	172.4 (36.5)	234.1 (36.4)	276.4 (27.7)

a Note: Other race includes White-caucasian/European Heritage, African/White and White/Asian.

Prior SARS-CoV-2 infection was reported in 28 participants (37%, Table 1). The impact of prior infection on baseline antibodies was evident (Supplementary Fig. S3), as the proportion of previously infected participants increased during the period of recruitment when omicron variants surged in Melbourne.^17^ By the time the higher dose participants were dosed, 75% and 100% of those in the Protein-RBD and mRNA-RBD groups respectively reported prior SARS-CoV-2 infection. No placebo group participant reported prior SARS-CoV-2 infection. In addition, the time between licensed 3rd dose (booster) dose and study vaccination increased as the ascending dose groups were vaccinated, rising from means of 160 days and 172 days for the lower dose groups of Protein-RBD and mRNA-RBD respectively, to 291 days and 276 days for the higher dose groups (Table 1).

### Vaccine safety and reactogenicity

No vaccine-related SAEs occurred, nor any adverse events of special interest (e.g. myocarditis, pericarditis, thrombosis, thrombocytopenia, Guillain-Barré syndrome) or vaccine related medically-attended AEs. There were no biochemical, serologic, or haematologic safety signals following vaccination. No participant had a rheumatoid factor titre >40IU/mL (pre-specified laboratory abnormality of special interest). Detailed analyses of anti-Fc antibodies demonstrated no detectable signal in protein RBD recipients compared to placebo and mRNA recipients (Supplementary Table S14). In addition, a selection of 28 samples (day 1 and 29) from 14 study participants (who, at day 1, prior to vaccination, despite being negative for rheumatoid factor, appeared to have some anti-Fc antibodies based on our in-house ELISA) were also tested for anti-Fc antibodies by nephelometry, a technique that detects immune anti-Fc complexes in solution. These results also showed no subject with induction of anti-Fc antibodies (data not shown, but can be provided on request).

**Fig. 2:**
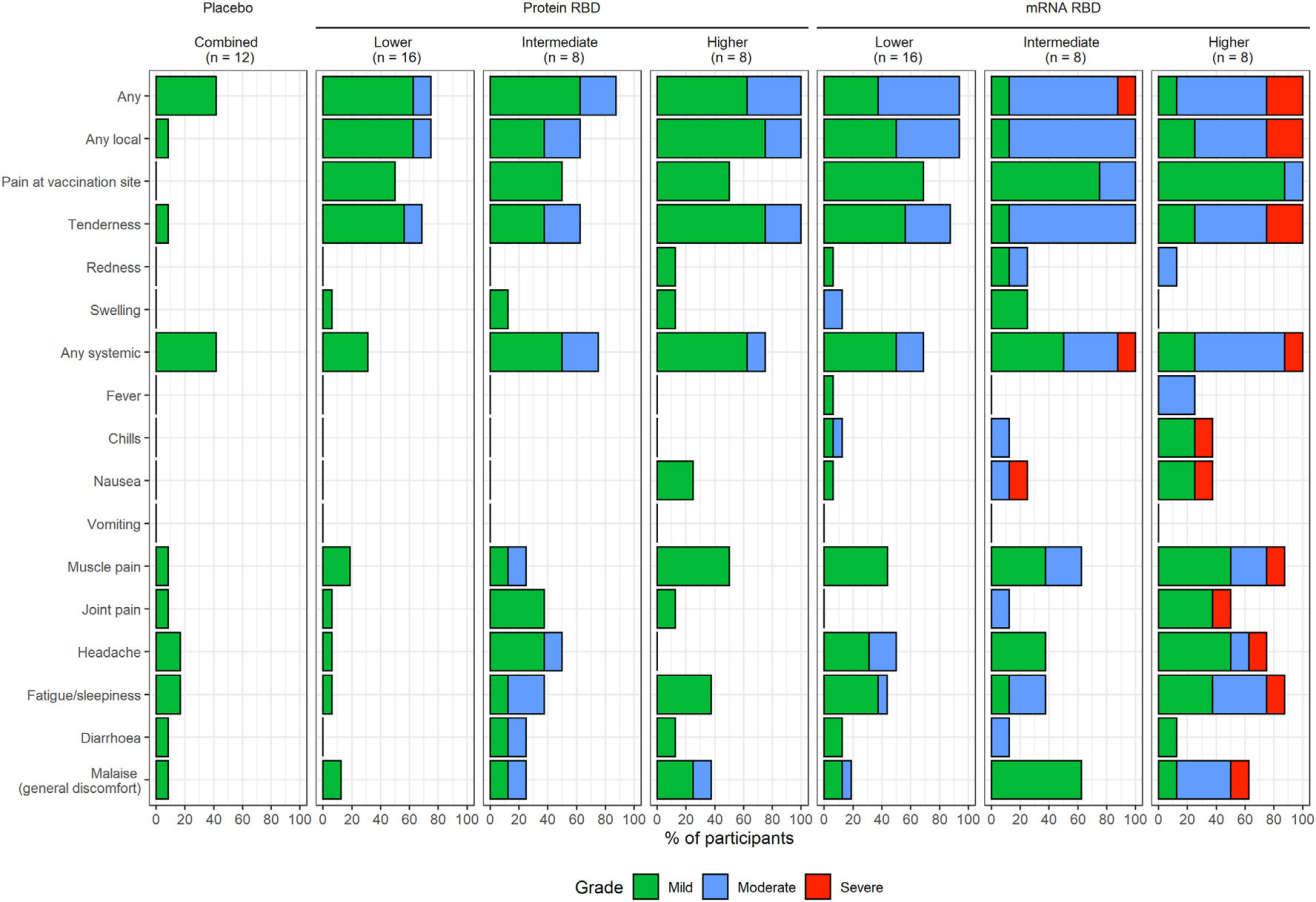
Adverse events.

The Protein-RBD vaccine reactogenicity profile was mild (no Grade-3 reactions). The mRNA-RBD vaccine was more reactogenic in terms of transient Grade-3 reactions at the intermediate (1 subject) and higher dose level (2 participants) (Fig. 2; Supplementary Table S2). No fever was recorded for any Protein-RBD or placebo subject, but mild transient fever occurred in 2 higher dose mRNA-RBD participants. Prior SARS-CoV-2 infection did not change the overall reactogenicity pattern.

### SARS-CoV-2 binding antibody responses

**Fig. 3:**
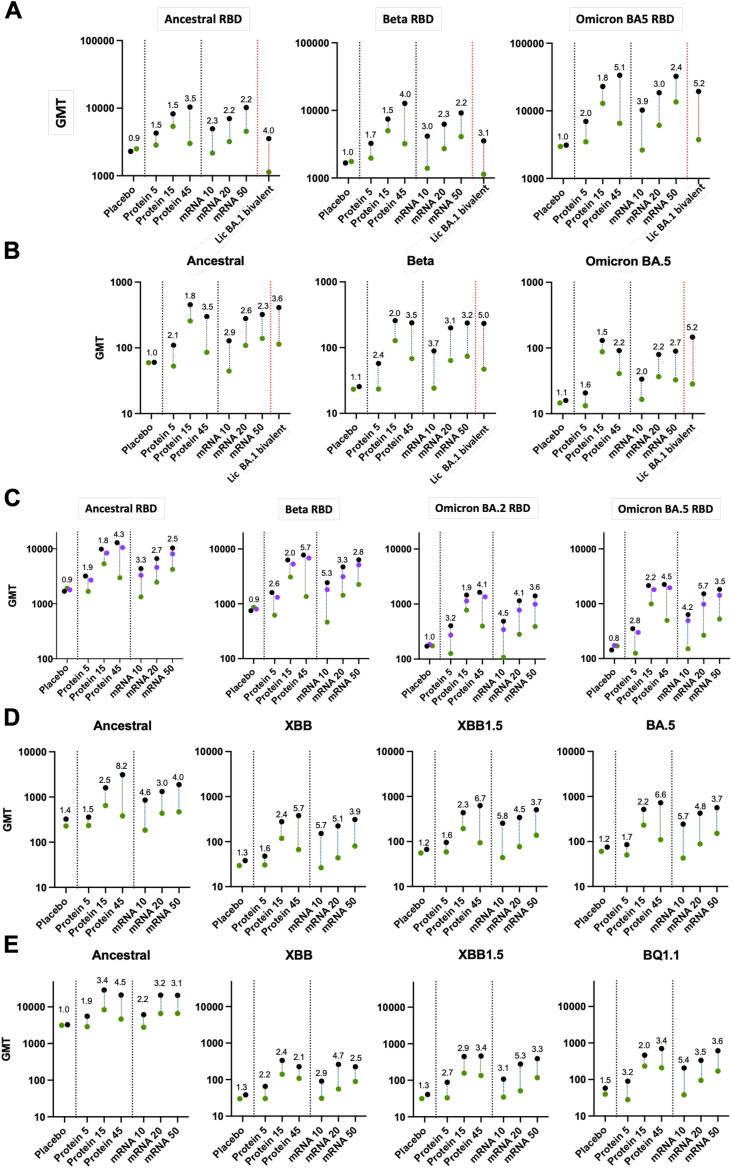
Antibody responses. Binding antibody (ELISA, row A); live virus microneutralisation (row B); sVNT multiplex using Method A (see Suppl. Materials) (row C); sVNT using Method B (see Supplementary Materials) (row D); and pVNT (row E). Numbers depict d29:d1 GMFR, dots are day 1 (green), day 8 (magenta) and day 29 (black). Rows A and B also show external cohort responses (N = 29) in participants from another study who received licensed (Moderna) BA.1/ancestral bivalent 4th dose.

**Fig. 4:**
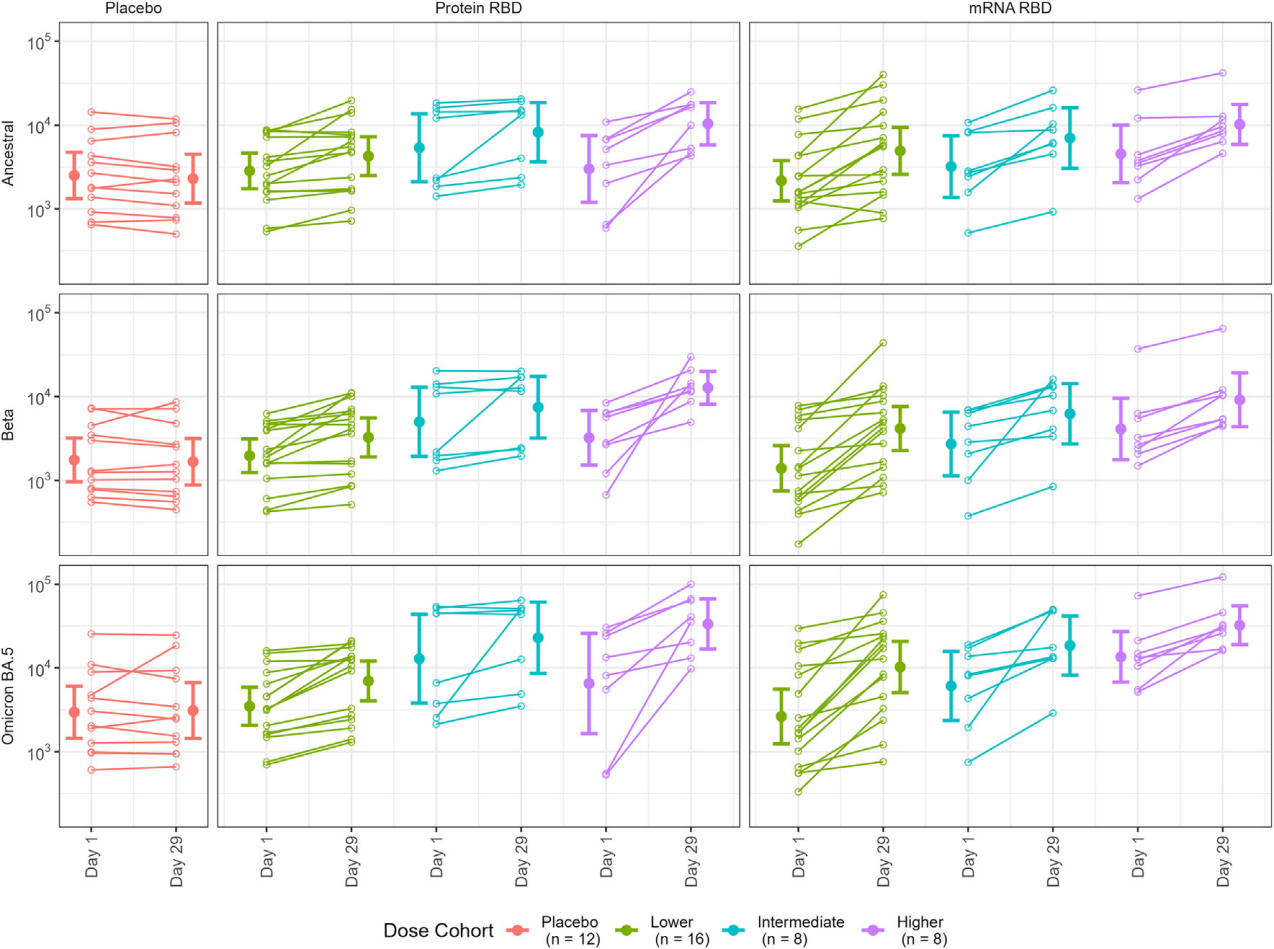
ELISA titres, individual plots d1/d29. WT = ancestral strain. Bars are geometric means and 95% confidence intervals.

**Fig. 5:**
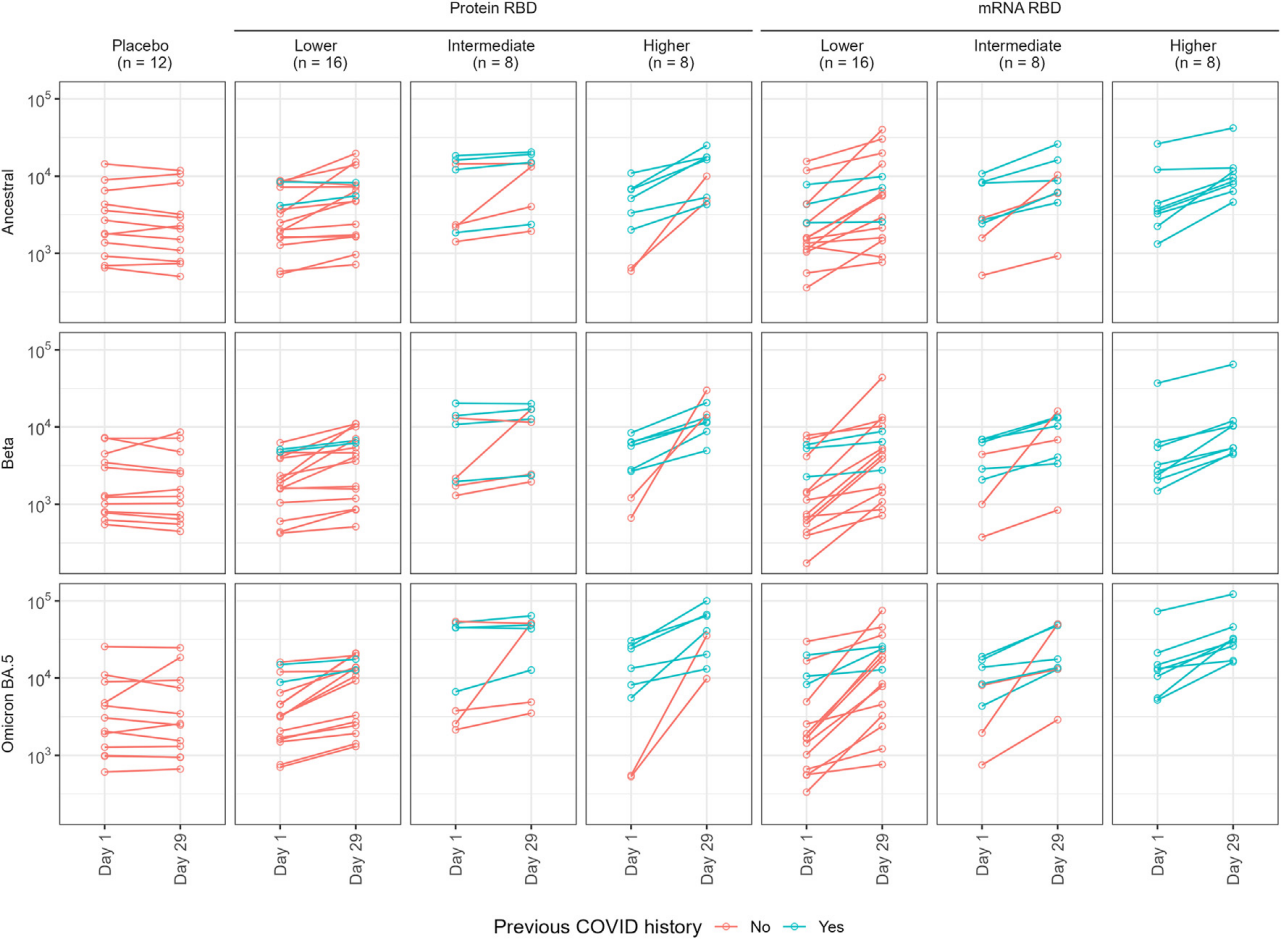
ELISA titres, d1/d29 by prior COVID-19. (WT = ancestral strain).

ELISA-based antibody titre analysis indicated that best responses for anti-RBD antibody were achieved for the 45 μg dose of protein vaccine with day 29 post-vaccination GM titre (GMT) of antibody against ancestral 10,390 (3.5-fold GMT rise, GMFR), beta of 12,730 (GMFR 4.0) and omicron BA.5 33,530 (GMFR 5.1) (Fig. 3, Fig. 4, Fig. 5). Highest mRNA-RBD GMFR responses were for the 10 μg dose: ancestral strain 4936 (GMFR 2.3), beta of 4155 (GMFR 3.0), and omicron BA.5 10,268 (GMFR 3.9), but the baseline antibody levels were substantially higher for the higher dose group. The post-boost antibody levels were higher for the 45 μg dose Protein-RBD than the 50 μg dose mRNA-RBD vaccines.

In terms of GMFR booster responses, there appeared to be a positive dose–response relationship for the Protein-RBD vaccine. Given that the baseline for the intermediate protein dose was the higher than the boosted response in the low dose group, it was difficult to determine whether the intermediate dose of protein vaccine provided a stronger boost than the lower dose because this will have reduced GMFR readings. A relationship was less clear for the mRNA-RBD vaccine in terms of GMFRs, where lower doses were quite immunogenic (Fig. 3, Fig. 4, Fig. 5; Supplementary Fig. S2; Supplementary Table S3). Again, higher baseline antibody levels in the intermediate and higher dose groups due to greater numbers of participants with prior SARS-CoV-2 infection may have reduced GMFR readings (Fig. 5).

BA.5 binding antibody titres were higher than those against ancestral and beta strains for both vaccines (33,530 for 45 μg Protein-RBD and 32,359 for 50 μg mRNA-RBD), with GMFRs of 5.1 and 2.4 for higher dose protein and mRNA vaccines respectively (Fig. 3, Fig. 4, Fig. 5; Supplementary Fig. S2; Supplementary Table S3). The higher readings for BA.5 may reflect the different source of BA.5 protein for ELISA assay, but nonetheless, the comparisons were internally controlled.

During the course of our trial, licensed mRNA vaccines were updated to a bivalent format including an omicron strain (BA.1). This provided the opportunity to determine if our beta variant protein and mRNA vaccines induced similar antibody responses to an omicron bivalent vaccine. While these samples were tested in a separate ELISA run, and notwithstanding differences in baseline antibody GMTs, the results showed GMFR of 4.0, 3.1 and 5.2 against ancestral RBD, beta RBD and omicron BA.5 RBD which were comparable to the GMFRs from 45 μg Protein-RBD and 10 μg mRNA-RBD vaccines (Fig. 3, row A).

### SARS-CoV-2 nAb responses

**Fig. 6:**
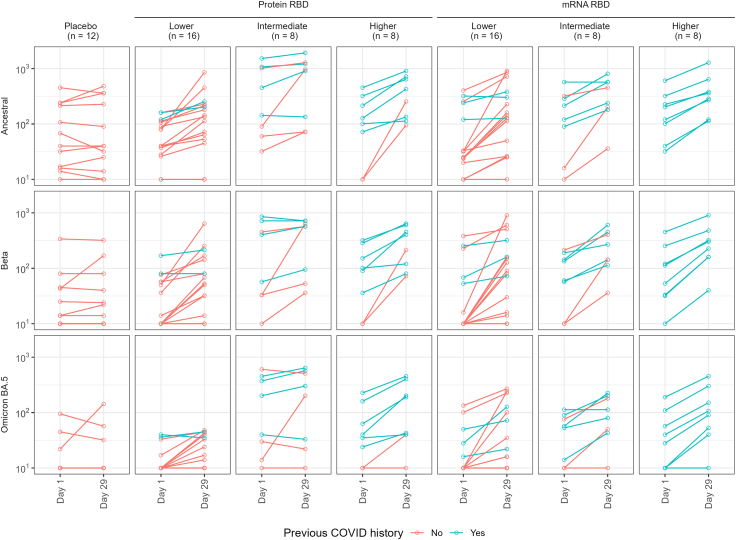
Microneutralisation, d1/d29 individual plots. (Vic01 = ancestral strain). Bars are geometric means and 95% confidence intervals.

A similar pattern to ELISA responses was seen with microneutralisation titres (Figs. 3 and 6, Supplementary Table S4), pseudovirus neutralisation (Fig. 3, row E; Supplementary Table S6; Supplementary Figs. S4 and S5) and multiplex bead-based surrogate virus neutralisation test (sVNT) (Fig. 3, rows C and D; Supplementary Tables S5 and S7; Supplementary Figs. S3, S6 and S7). Both vaccines demonstrated neutralising activity against a broad range of SARS-CoV variants, including the ancestral strain, beta and omicron variants (BA.1, BA.2, BA.5, BQ.1.1, XBB, and XBB.1.5), alpha, delta, delta^+^, gamma, lambda, and mu, as well as the more distantly related SARS-CoV-1. A comparable pattern to binding antibody results was evident in BA.5, XBB and XBB.1.5 surrogate virus neutralisation responses, though optimal GMFRs were greater at 6.6, 5.7 and 6.7 for 45 μg dose Protein-RBD, and 5.7, 5.7 and 5.8 for 10 μg dose mRNA-RBD vaccines, respectively.

BA.5 microneutralisation titres were substantially lower than those against beta and ancestral strains for both vaccines, despite strong GMFRs of 2.2 and 2.7 for higher dose protein and mRNA vaccines respectively (Fig. 3, row B and Fig. 6; Supplementary Table S4; Supplementary Fig. S2). For XBB, XBB.1.5 and BQ.1.1, pseudovirus neutralisation titres were about 3% of those elicited against beta for both vaccines (Supplementary Table S6), though GMFRs were 2.1, 3.4 and 3.4 for 45 μg dose Protein-RBD and 2.9, 3.1 and 5.4 for 10 μg dose mRNA-RBD vaccines, respectively.

### SARS-CoV-2 T-cell responses

**Fig. 7:**
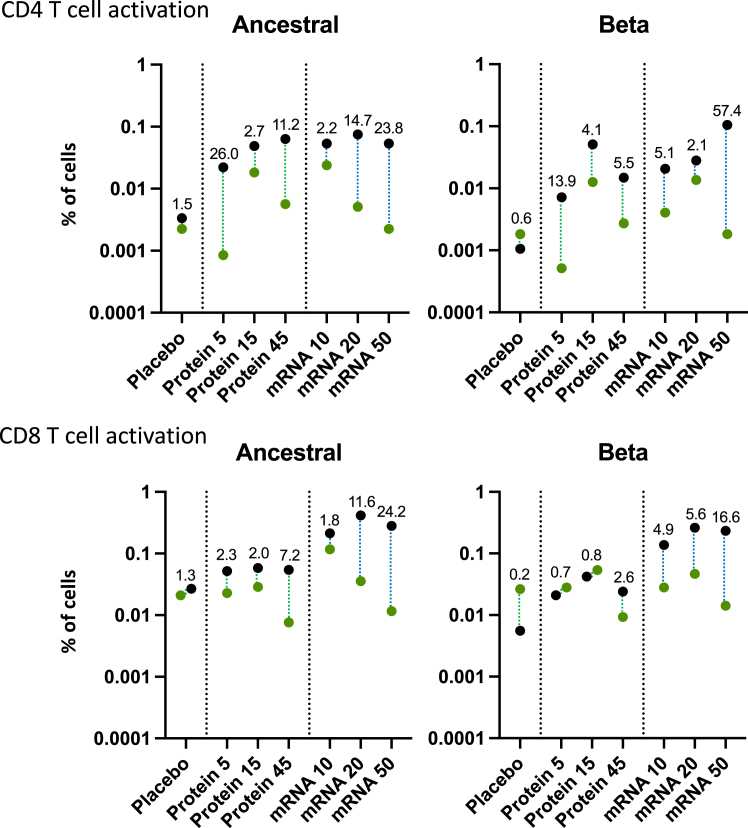
Activated T cell responses towards ancestral and beta RBD-derived peptides. Proportion of CD134^+^CD137^+^ and CD69^+^CD137^+^ activated cells of the total CD4^+^ and CD8^+^ T cell population, respectively, following AIM assay with RBD-derived peptides from ancestral and beta strains. Green (d1) and black (d29) circles depict GM datapoints. Numbers depict d29:d1 GMFR.

Both protein and mRNA vaccines enhanced CD4^+^ and CD8^+^ T cell activation as measured by upregulation of activation markers (CD69, CD134, CD137) in response to ancestral and beta RBD-derived peptides with comparable responses (mean % of activated T cells) (Data summarised in Fig. 7; full data provided in Supplementary Tables S10 and S11). However, CD8^+^ T cell activation was more pronounced for the mRNA-RBD vaccine compared to the Protein-RBD vaccine (higher dose fold-ratio of 7.2 for Protein-RBD versus 24.2 for mRNA-RBD vaccines against ancestral RBD. Importantly, levels of T cell activation were comparable between vaccine doses, as well as in response to ancestral or beta RBD-derived peptides. Therefore, the Protein-RBD vaccine, and to an even larger extent the mRNA-RBD vaccine, enhanced RBD-specific T cell responses that were cross-reactive towards ancestral and beta RBD.

Cytokine profiles from CD4 and CD8 T cells stimulated with ancestral and beta RBD-derived peptides were also assessed using intracellular cytokine staining. These data revealed a baseline displayed a mix of Th1 (IFN-γ, TNF) and Th2 cytokines (IL-5 and IL-13), presumably established by prior vaccination and/or prior SARS-CoV-2 infection. There was no consistent change in cytokine profiles in the day 29 samples for either the protein or mRNA vaccines (Supplementary Tables S8, S9, S12 and S13). While we did not see a consistent increase in any given T cell cytokine, this may be because cytokines are not absolute markers of T cell activation; with different T cells making different cytokines, or combinations thereof, at different times following activation. This will have been primarily shaped by the prior three vaccinations, and infections, that individual donors experienced prior to participating in our phase I trial. Thus, as the intracellular cytokine staining approach that is commonly used by us and others captures a small snapshot of what activated T cells will be capable of doing. Hence, the activation marker analysis is a more robust indicator of T cell activation.

### COVID cases during study follow-up

Only 3 participants tested positive for SARS-CoV-2 between d1 and d29, one of whom did not agree to PCR testing (RAT + only). PCR demonstrated omicron BA.2-like in both the PCR-tested participants (Supplementary Table S15).

## Discussion

This Phase I study provides evidence of the safety and tolerability of recombinant protein and mRNA-LNP vaccines directed at the beta variant RBD of SARS-CoV-2. The data also show that in a highly immunised cohort with a substantial degree of prior SARS-CoV-2 infection, robust boosting of antibody levels is achieved. The dose–response relationship for boosting, as measured by GMT, was approximately linear for both the Protein-RBD and mRNA-RBD vaccines. However, when examining GMFR relative to baseline responses that were higher in the intermediate and higher dose groups, dose relationships were less apparent and even reversed for the mRNA vaccine. Similarly, while there was evidence of CD4^+^ and CD8^+^ activation, prior vaccination and SARS-CoV-2 infection made T cell responses harder to interpret.

Breadth of immune responses boosted by the beta RBD vaccines was demonstrated against BA.5 for ELISA and all neutralisation assays, and in sVNT and pVNT assays also for more recent omicron subvariants (BA.1, BA.2, XBB, XBB.1.5 and BQ.1.1). This possibly reflects the benefit of beta variant-directed candidates and focussed RBD presentation that is less susceptible to immunological imprinting targeting conserved and non-neutralising epitopes.^5^, ^6^, ^7^, ^8^, ^9^, ^10^ Neutralisation and binding GMFR for both vaccines were broadly comparable (other than for BA.5 neutralisation) to those of the external comparator licensed bivalent mRNA vaccine (Fig. 3, rows A and B). While this comparison is complicated by the fact that the baseline for the higher dose trial vaccine groups was higher than it was for the licensed vaccine group, and that the assays were performed at a different time, the data suggest that both protein and mRNA beta RBD-based vaccines perform similarly to the licensed bivalent vaccine.

RBD-reactive CD4^+^ and CD8^+^ T cells were already detectable in baseline samples, presumably due to prior vaccination and/or SARS-CoV-2 infection. Nonetheless, the frequency of activated CD4^+^ and CD8^+^ T cells was enhanced following vaccination with either Protein-RBD or mRNA-RBD vaccine, as measured by AIM assay, a method previously used to study overlapping Spike peptide-specific T cell responses following SARS-CoV-2 infection^18^^,^^19^ and vaccination.^20^ CD8^+^ T cell activation was more pronounced for the mRNA vaccine compared to the protein vaccine, which is consistent with other observations that protein-based vaccines have been shown to generate less CD8^+^ T cell responses compared to their CD4^+^ counterparts, as is reported for COVID-19 vaccine candidates^23^ and inactivated influenza vaccines.^24^

The nature of the evolution of the SARS-CoV-2 virus has required updating of the vaccine due to the development of VOC. This resulted in calls for RBD-based COVID vaccines that would potentially reduce the risk of vaccine escape and imprinting, and provide a more efficient basis for mass production to meet global vaccine needs.^11^ Compounding the effect of variants, waning of immunity following vaccination and/or infection remains a significant challenge for SARS-CoV-2 vaccines.

Several RBD protein vaccines are now in various stages of development or implementation,^25^ and selected candidates in late-stage clinical trials or licensure are summarised in Table 2.^26^, ^27^, ^28^, ^29^, ^30^, ^31^, ^32^, ^33^, ^34^, ^35^, ^36^, ^37^, ^38^, ^39^, ^40^ Of particular interest are: (1) A dimeric RBD vaccine (ZF2001) where two RBD subunits are linked via an engineered single-chain construct, adjuvanted with alum, approved for emergency use in China and some other countries. This was tested as a three-dose schedule^28^; (2) Cuba has developed a series of RBD-based vaccines including Soberana-02 (RBD conjugated to tetanus toxoid) and a dimeric RBD vaccine to be used as a booster (Soberana-Plus), both adjuvanted with Alum. Both are licensed and deployed in Cuba and Iran^29^; (3) An RBD human IgG_1_-Fc dimer, fused to IFN-α and an MHC class–II binding element, combined with alum adjuvant (V-01), recently tested in phase III clinical trial^35^; (4) An RBD-human IgG_1_ Fc vaccine (ancestral strain) with montanide oil-in-water adjuvant (AKS-452) in a two-dose prime-boost schedule has progressed through a phase I/II trial.^26^ In general, these vaccines have been well-tolerated and are highly immunogenic. One candidate (PHH–1V) has a dimeric alpha-beta construct.^36^ Furthermore, there is only one mRNA-RBD candidate ARCOV-mRNA^40^ and has recently been granted emergency licence use authorisation in Indonesia. We are not aware of any clinical trials that directly compare protein-to mRNA-based RBD vaccines. While there are several RBD vaccines in clinical trials, it is not possible to compare their efficacy to ours for two key reasons: firstly, ours is a small phase I trial and deliberately not powered to assess or compare efficacy and secondly, this is the only study to test RBD vaccines as fourth dose boosts following three immunisations with licensed, highly immunogenic vaccines, with several subjects also having been infected with COVID in prior months. Consequently, baseline antibody levels were already very high in many of the subjects in our study (Fig. 4, Fig. 5, Fig. 6) which may limit how much enhancement can be achieved with a fourth dose boost.

**Table 2:** Selected SARS-CoV-2 RBD vaccine candidates in later-stage development or licensure.

Developer & vaccine (reference)	Platform	Construct	Adjuvant	Trial phase, licence
Akston Biosciences *AKS-452*^26^	CHO	Fc-dimeric RBD	Montanide (oil-in-water)	3
SK Bioscience *GBP510*^27^	CHO/E.Coli	Self-assembling protein nanoparticle displaying 60 RBDs	AS03	3
Anhui Zhifei Longcom, Chinese Acad. of Medical Sciences *ZF2001*^28^	CHO	Tandem RBD protein dimer	Alum hydroxide	3, EUA Uzbekistan, Indonesia, China, Colombia.
Finlay Vaccine Institute *Soberana-02 & Soberana-Plus*^29^	Baculovirus	Soberana-02 RBD conjugated to tetanus toxoid. Soberana-Plus (disulfide-linked dimeric RBD).	Alum hydroxide 500 μg (02) & 1250 μg (Plus)	3, license Cuba
Center for Genetic Engineering & Biotechnology Cuba, *Abdala CIGB-66*^30^	Yeast	Monomeric RBD	Alum hydroxide	3, licence Cuba, Mexico, Nicaragua, Venuzuela, Vietnam
Biological E, *Corbevax* *RBD219-N1C1*^31^	Yeast	Monomeric RBD	Alum hydroxide & CPG1018	3, Emerg. Licence India, Botswana
West China Hospital, WestVac Biopharma *SARS-Cov-2 RBD*^32^	Baculovirus	Monomeric RBD	Alum hydroxide	3, started June 2021
Covaxx, United Biomedical Asia & Vaxxinity, *UB-612*^33^	CHO	RBD-Fc plus 6 conserved T cell stimulatory peptides.	Aluminium phosphate & CPG	3, Licence submission UK, Australia.
EuBiologics *EuCorVac-19*^34^	CHO	RBD displayed on immunogenic nanoliposomes.	Monophosphoryl lipid A (MPLA) nanoliposomes	3, started October 2022.
Joincare, Livzon Mabpharm. *V-01*^35^	CHO	RBD-Fc dimer incorporating interferon-α (IFNα) and pan human leukocyte antigen-DR-binding epitope at N-terminus.	Alum hydroxide	3
HIPRA. *PHH-1V*^36^	CHO	RBD fusion heterodimer incorporating alpha and beta variant RBDs	SQBA (Squalene-based oil-in-water)	1/2a
Laboratorio Pablo Cassará *ARVAC CG*^37^	CHO	Single chain dimer of gamma variant RBD	Alum hydroxide	1
Radboud University, NL. *ABNCoV2*^38^	Schneider-2 (ExpreS2) cells	RBD-coated capsid virus-like particle	MF59 in a subgroup	1
LLC Betuvax *Betuvax-CoV-2*^39^	CHO	RBD-Fc dimer assembled as *Betuspheres* (betulin nanoparticles)	Betulin (triterpenoid of lupane structure)	1/2
Walvax/Abogen *ARC0V-mRNA (AWcorna)*^40^	mRNA	RBD mRNA encapsulated in Abogen proprietary LNP	N/A	3, emerg licence Indonesia (Sept 2022)

All but three are targeted at ancestral strain RBD—a beta variant version of the V-01 vaccine that was tested in a pilot study, the HIPRA candidate has a beta RBD target (as alpha/beta heterodimer), and ARVAC CG uses the gamma variant RBD.

Preclinical (mouse) studies of our Protein-RBD vaccine showed that the RBD presented as an Fc-linked dimer produced superior primary and secondary immune responses compared to RBD monomers and single-chain RBD dimers.^16^ Furthermore, the Fc dimer construct means that the vaccine can be rapidly mass-produced by facilities that are used to generate mAb therapeutics, and then efficiently purified using protein A-based purification. While anti-Fc responses from the RBD-Fc dimer vaccine were not expected due to self-tolerance, clinical anti-Fc (rheumatoid factor) assessment was included as part of our sentinel and ongoing safety analyses and more thoroughly examined by ELISA and nephelometry. No evidence was detected for an influence of Protein-RBD vaccine on pre-vaccination anti-Fc antibodies (Supplementary Table S14) or anti-Fc T cell responses (Supplementary Tables S12 and S13).

Recognising that this is a Phase I trial, there are some limitations which will need to be addressed in subsequent studies. There were no elderly participants (65 y+) because of ethical constraints around placebo receipt in the context of public health booster recommendations at the time of recruitment. Nevertheless, other licensed COVID-19 vaccines have not shown reduction in efficacy with increasing age.^41^ It was not possible to include a licensed COVID-19 vaccine as an internal blinded comparator because it was impossible to source vaccines for study in Australia due to government and manufacturer restrictions on access. Therefore, we sourced an external comparison group, being studied as part of an evaluation of licensed bivalent booster vaccination and were able to evaluate serum samples using the same assays in the same lab as used for our Phase I participants. Future studies (Phase 2 and beyond) should nonetheless include a suitable licensed comparator. While we have been able to evaluate surrogate and pseudovirus neutralisation of recent omicron sub-variants (XBB, XBB.1.5 and BQ.1.1), the most recent and current sub-variants have not been evaluated. The level of prior vaccine and disease-acquired immunity now complicates the immunologic evaluation of all SARS-CoV-2 vaccines particularly in small phase I trials. Finally, waning of immunity remains a significant challenge for SARS-CoV-2 vaccines.^42^ Our interim report cannot address this question, but we will have data from 3- and 6-month time points at the conclusion of our trial.

In summary, this Phase I study interim analysis identified no safety concerns and the reactogenicity profile was mild and similar to licensed COVID-19 vaccines. Both protein and mRNA beta RBD vaccines boosted immune responses against beta, ancestral and omicron BA.5 strains based on ELISA, microneutralisation, sVNT and pVNT assays, with optimal vaccine doses of 45 μg and possibly 50 μg respectively. Based on sVNT and/or pVNT data, neutralising antibody titres against several other variants, including alpha, delta, lambda, delta^+^, gamma, mu and omicron subvariants (BA.1, BA.2, XBB, XBB.1.5 and BQ.1.1) were also boosted by both protein and mRNA vaccines. Both vaccines boosted CD4^+^ and CD8^+^ T cell responses, with stronger CD8^+^ responses detected following the mRNA vaccine boost. While the use of beta variant RBD has strong cross-variant immunity, the vaccine platforms used in this study are rapidly adaptable to align with other variants if desirable. Further evaluation of these candidates, either alone or in combination with other respiratory disease vaccines is warranted.

## Contributors

Investigation and formal analysis: T.M.N., G.D., M.G., S.B., L.F.A., J.A., A.W.C., M.C., N.A.G., M.L.G., T.P.G., S.L.G., L.H., D.C.J., J.A.J., K.K., S.J.K., H.A.McQ., F.L.M., T.H.O.N., V.P.S., B.P., D.F.J.P., P.R., S.J.R., S.R., Z.R., J.S., J.A.S., K.S., S.A.F., T.J.P., A.T., C.W.T., J.T., J.J.W., L.W., H.Al.W., C.Y.W., S.Z., C.W.P., and D.I.G.

Resources: T.M.N., A.W.C., T.P.G., D.C.J., K.K., S.J.K., S.R.L., D.F.J.P., S.R., J.S., J.A.S., K.S., L.W., C.W.P., and D.I.G.

Project administration: T.M.N., M.G., G.D., J.A.S., D.I.G., and S.L.

Conceptualisation: T.M.N., G.D., M.G., S.R., D.C.J., D.F.J.P., C.W.P., and D.I.G.

Writing–original draft: T.M.N., D.I.G. and M.G.

Writing–reviewing and editing: All authors.

Verification of underlying data: T.M.N., M.G., S.B., G.D. and D.I.G.

All authors read and approved the final version of the manuscript.

## Data sharing statement

Data reported in this article is available upon reasonable request for 3 years after publication.

## Declaration of interests

The vaccines evaluated in this Phase I study were the result of independent University of Melbourne and Monash University research and development, with funding provided by the Australian Government's Medical Research Future Fund (MRFF), the National Health and Medical Research Council (NHMRC), the Victorian Government (mRNA Victoria) and philanthropic funders. The MF59 for the protein-RBD candidate was donated by CSL Seqirus. One author (S.R.) is an employee of CSL Seqirus and he also has an adjunct (honorary) appointment to the University of Melbourne. G.D., N.G., D.P., D.I.G. are named inventors on 2 provisional patents for the RBD-Fc dimer vaccine in this study. T.M.N. has been a DSMB member for vaccine studies conducted by Moderna, Clover, Novavax, CSL Seqirus, and SK Bioscience Korea, and has received payment for advisory roles on vaccines from AstraZeneca, Moderna, MSD, Sanofi, CSL, and Pfizer. G.D. received salary support from philanthropic funds from IFM Investors Pty Ltd. S.L. received consulting fees from several companies related to HIV research, and honoraria from MSD and Gilead. H.McQ. received consulting fees from Ena Respiratory Pty Ltd. K.S. was a member of a DSMB for a vaccine study in Thailand. H.alW. received consulting and research funding from CSL Seqirus. D.I.G. received research support from CSL.

## Appendix A Supplementary data

CONSOLIDATED Supplementary Appendices 2nd revision

Study Protocol Version
