# Supplementary material for: Interim results from a phase I randomized, placebo-controlled trial of novel SARS-CoV-2 beta variant receptor-binding domain recombinant protein and mRNA vaccines as a 4th dose booster
Source: eBioMedicine. 2023 Nov 27;98:104878. doi: 10.1016/j.ebiom.2023.104878 (PMC10696466; doi:10.1016/j.ebiom.2023.104878)
Supplement: CONSOLIDATED Supplementary Appendices 2nd revision [file mmc1.pdf]

Supplementary Tables, Figures and Methods

Title: Interim results from a Phase I trial of novel SARS-CoV-2 beta variant receptor-binding domain recombinant protein and mRNA vaccines as a 4th dose booster.

TABLE OF CONTENTS

Part A: SUPPLEMENTARY TABLES

Page	Table	Description
2	1	Adverse event grading scale
3	2	Solicited adverse reactions within 7 days after injection
4	3	Anti-RBD binding antibody (ELISA)
5	4	Live virus microneutralisation
6	5	Surrogate virus neutralization test (sVNT); Method B
7	6	Pseudovirus neutralization test (pVNT)
8	7	Multiplex surrogate virus neutralization test (sVNT); Method A
9	8	T cell whole blood stain absolute number
10	9	T cell whole blood stain frequency
11	10	CD4 T cell stimulation assay activation induced marker (AIM) responses
12	11	CD8 T cell stimulation assay activation induced marker (AIM) responses
13-14	12	CD4 T cell cytokine responses by intracellular staining (ICS)
15-16	13	CD8 T cell cytokine responses by intracellular staining (ICS)
17	14	Anti-Fc antibody responses
18	15	SARS-CoV-2+ participants

Part B: SUPPLEMENTARY FIGURES

Page	Figure	Description
19	1	Microneutralisation d1/d29 by prior COVID-19, individual plots
20	2	Pre-Post GMFR ELISA and microneutralisation
21	3	sVNT (Method B) geometric mean fold rises (GMFR)
22	4	pVNT geometric mean fold rises (GMFR)
23	5	pVNT individual plots, d1/d29 (Linfa Wang lab)
24	6	sVNT individual plots (Part 1), d1/d29 (Method B, Linfa Wang lab)
25	7	sVNT individual plots (Part 2), d1/d29 (Method B, Linfa Wang lab)

Part C: SUPPLEMENTARY METHODS

Text document (Pages 26-28)

Supplementary Table 1: Adverse Event Grading

	Mild (Grade 1)	Moderate (Grade 2)	Severe (Grade 3)
**Solicited Local Adverse Events**			
Pain at vaccination site	Does not interfere with daily activity	Repeated use of non-narcotic pain reliever >24 hours or interferes with daily activity	Any use of narcotic pain reliever or prevents daily activity
Tenderness	Mild discomfort to touch	Discomfort with movement	Significant discomfort at rest
Erythema/Redness	2.5 - 5 cm	5.1 - 10 cm	>10 cm
Induration/Swelling	2.5 - 5 cm and does not interfere with daily activity	5.1 - 10 cm or interferes with daily activity	>10cm or prevents daily activity
**Solicited Systemic Adverse Events**			
Fever °C (Oral)	38.0 – 38.4	38.5 – 38.9	39.0-40.0
Chills	Present but does not interfere with daily activity	Interferes with daily activity	Prevents daily activity
Nausea	Nausea present but not interfering with daily activities	Nausea present leading to decreased oral intake	Nausea present leading to minimal to no oral intake
Vomiting	No interference with activity or 1-2 episodes in 24 hours	Some interference with activity or >2 episodes in 24 hours	Prevents daily activity, ≥4 episodes in 24 hours, or 2 or more episodes per day prolonged on several days
Muscle pain	Present but does not interfere with activity	Interferes with activity, or some use of non-narcotic pain reliever	Prevents daily activity; use of narcotic pain reliever
Joint pain	Present but does not interfere with activity	Interferes with activity, or some use of non-narcotic pain reliever	Prevents daily activity; use of narcotic pain reliever
Headache	Present but does not interfere with activity	Interferes with activity, or some use of non-narcotic pain reliever	Prevents daily activity; use of narcotic pain reliever
Fatigue/Somnolence	Present but does not interfere with activity	Interferes with activity	Prevents daily activity
Diarrhoea	2 - 3 loose stools or <400g /24 hours	4 - 5 loose stools or 400-800g /24 hours	6 or more watery stools or >800g / 24 hours or requires outpatient IV hydration
Malaise (General discomfort)	No interference with daily activity	Some interference with usual and social activity, no treatment	Significant, prevents usual daily and social activity or requires treatment

Protocol Table 6 Severity Grading for Unsolicited Adverse Events

Mild (Grade 1)	Events that are usually transient and may require only minimal or no treatment or therapeutic intervention and generally do not interfere with the participant's usual activities of daily living.
Moderate (Grade 2)	Events that are usually alleviated with additional specific therapeutic intervention. The event interferes with usual activities of daily living, causing discomfort but poses no significant or permanent risk of harm to the participant.
Severe (Grade 3)	Events that interrupt usual activities of daily living, or significant affects clinical status, or may require intensive therapeutic intervention. Severe events are usually incapacitating.

Supplementary Table 2: Solicited adverse reactions within 7 days after injection by grade (Solicited Safety Set)

		**Placebo**	**Protein-RBD**			**mRNA-RBD**		
			**5µg**	**15µg**	**45µg**	**10µg**	**20µg**	**50µg**
	Grade	N=12	N=16	N=8	N=8	N=16	N=8	N=8
Any	3	0	0	0	0	0	1 (13%)	2 (25%)
	2	0	2 (13%)	2 (25%)	3 (38%)	9 (56%)	6 (75%)	5 (63%)
	1	5 (42%)	10 (63%)	5 (63%)	5 (63%)	6 (38%)	1 (13%)	1 (13%)
Any local	3	0	0	0	0	0	0	2 (25%)
	2	0	2 (13%)	2 (25%)	2 (25%)	7 (44%)	7 (88%)	4 (50%)
	1	1 (8%)	10 (63%)	3 (38%)	6 (75%)	8 (50%)	1 (13%)	2 (25%)
Pain at vaccination site	3	0	0	0	0	0	0	0
	2	0	0	0	0	0	2 (25%)	1 (13%)
	1	0	8 (50%)	4 (50%)	4 (50%)	11 (69%)	6 (75%)	7 (88%)
Tenderness	3	0	0	0	0	0	0	2 (25%)
	2	0	2 (13%)	2 (25%)	2 (25%)	5 (31%)	7 (88%)	4 (50%)
	1	1 (8%)	9 (56%)	3 (38%)	6 (75%)	9 (56%)	1 (13%)	2 (25%)
Redness	3	0	0	0	0	0	0	0
	2	0	0	0	0	0	1 (13%)	1 (13%)
	1	0	0	0	1 (13%)	1 (6%)	1 (13%)	0
Swelling	3	0	0	0	0	0	0	0
	2	0	0	0	0	2 (13%)	0	0
	1	0	1 (6%)	1 (13%)	1 (13%)	0	2 (25%)	0
Any systemic	3	0	0	0	0	0	1 (13%)	1 (13%)
	2	0	0	2 (25%)	1 (13%)	3 (19%)	3 (38%)	5 (63%)
	1	5 (42%)	5 (31%)	4 (50%)	5 (63%)	8 (50%)	4 (50%)	2 (25%)
Fever	3	0	0	0	0	0	0	0
	2	0	0	0	0	0	0	2 (25%)
	1	0	0	0	0	1 (6%)	0	0
Chills	3	0	0	0	0	0	0	1 (13%)
	2	0	0	0	0	1 (6%)	1 (13%)	0
	1	0	0	0	0	1 (6%)	0	2 (25%)
Nausea	3	0	0	0	0	0	1 (13%)	1 (13%)
	2	0	0	0	0	0	1 (13%)	0
	1	0	0	0	2 (25%)	1 (6%)	0	2 (25%)
Vomiting	3	0	0	0	0	0	0	0
	2	0	0	0	0	0	0	0
	1	0	0	0	0	0	0	0
Muscle pain	3	0	0	0	0	0	0	1 (13%)
	2	0	0	1 (13%)	0	0	2 (25%)	2 (25%)
	1	1 (8%)	3 (19%)	1 (13%)	4 (50%)	7 (44%)	3 (38%)	4 (50%)
Joint pain	3	0	0	0	0	0	0	1 (13%)
	2	0	0	0	0	0	1 (13%)	0
	1	1 (8%)	1 (6%)	3 (38%)	1 (13%)	0	0	3 (38%)
Headache	3	0	0	0	0	0	0	1 (13%)
	2	0	0	1 (13%)	0	3 (19%)	0	1 (13%)
	1	2 (17%)	1 (6%)	3 (38%)	0	5 (31%)	3 (38%)	4 (50%)
Fatigue/sleepiness	3	0	0	0	0	0	0	1 (13%)
	2	0	0	2 (25%)	0	1 (6%)	2 (25%)	3 (38%)
	1	2 (17%)	1 (6%)	1 (13%)	3 (38%)	6 (38%)	1 (13%)	3 (38%)
Diarrhoea	3	0	0	0	0	0	0	0
	2	0	0	1 (13%)	0	0	1 (13%)	0
	1	1 (8%)	0	1 (13%)	1 (13%)	2 (13%)	0	1 (13%)
Malaise	3	0	0	0	0	0	0	1 (13%)
(general discomfort)	2	0	0	1 (13%)	1 (13%)	1 (6%)	0	3 (38%)
	1	1 (8%)	2 (13%)	1 (13%)	2 (25%)	2 (13%)	5 (63%)	1 (13%)

Supplementary Table 3: SARS-CoV-2 RBD specific binding antibody ELISA (titres)

		Placebo		Protein-RBD						mRNA-RBD					
				5µg		15µg		45µg		10µg		20µg		50µg	
		Day 1	Day 29	Day 1	Day 29	Day 1	Day 29	Day 1	Day 29	Day 1	Day 29	Day 1	Day 29	Day 1	Day 29
	N	12	12	16	16	8	8	8	8	16	16	8	8	8	8
WT (ancestral)	GMT	2510·7	2299·0	2844·9	4268·3	5373·4	8240·2	3003·5	10390·2	2169·4	4935·6	3204·4	7023·6	4542·0	10215·3
	95% CI	1328·0-4746·6	1174·0-4502·1	1741·9-4646·1	2503·0-7278·3	2110·4-13681·5	3653·4-18585·6	1199·6-7520·1	5809·8-18581·7	1245·0-3780·1	2589·1-9408·8	1372·5-7481·6	3053·2-16157·5	2058·1-10024·0	5900·0-17686·8
	Median (IQR)	2244 (1149-5403)	2175 (939-5690)	2864 (1609-7642)	5191 (1691-7931)	7228 (2027-15239)	13891 (3200-17166)	4237 (1332-6769)	13150 (5011-17599)	1589 (1173-4335)	5631 (1870-12103)	2769 (1999-8261)	7463 (5265-13251)	3652 (2747-8292)	9115 (7134-12129)
	GMFR referencing Day 1		0·9		1·5		1·5		3·5		2·3		2·2		2·2
	95% CI		0·8-1·0		1·2-1·9		0·9-2·5		1·8-6·8		1·5-3·3		1·4-3·4		1·5-3·4
Beta	GMT	1754·4	1669·5	1968·2	3258·6	4993·9	7455·9	3222·5	12729·5	1392·3	4154·6	2718·0	6240·0	4103·2	9166·2
	95% CI	962·6-3197·5	880·1-3166·8	1238·7-3127·3	1907·1-5567·8	1931·2-12913·4	3199·7-17373·8	1519·2-6835·3	8110·3-19979·8	746·7-2595·9	2270·5-7602·4	1132·1-6525·5	2725·3-14287·7	1764·4-9542·2	4373·9-19208·9
	Median (IQR)	1266 (790-3984)	1411 (689-3724)	1978 (1320-4369)	4382 (1384-6877)	6496 (1848-13538)	12120 (2402-17002)	4239 (1942-6357)	12529 (10067-17566)	1266 (593-4719)	4615 (1549-9534)	3644 (1537-6606)	8573 (3712-13261)	2952 (2222-5875)	7849 (4998-11229)
	GMFR referencing Day 1		1·0		1·7		1·5		4·0		3·0		2·3		2·2
	95% CI		0·8-1·1		1·3-2·1		0·8-2·7		1·5-10·4		1·9-4·6		1·2-4·5		1·7-3·0
Omicron BA4/5	GMT	2965·4	3109·0	3493·9	6996·9	12901·1	22974·7	6532·2	33530·3	2640·1	10268·3	6098·9	18530·0	13579·2	32359·4
	95% CI	1449·4-6066·9	1444·7-6690·8	2071·7-5892·5	4049·5-12089·7	3806·2-43727·5	8631·3-61153·9	1646·9-25908·9	16819·2-66845·2	1246·2-5593·1	5078·9-20760·3	2360·6-15757·0	8200·7-41869·9	6775·7-27214·3	18975·8-55182·3
	Median (IQR)	2548 (1132-6859)	2515 (1126-8387)	3245 (1647-7626)	11472 (2572-15653)	25653 (3154-48525)	46071 (8786-51347)	10744 (3035-25055)	38139 (16688-65142)	1796 (838-9402)	14981 (3912-24774)	8219 (3141-15484)	15589 (13017-48791)	12983 (8014-18013)	30426 (21419-39361)
	GMFR referencing Day 1		1·0		2·0		1·8		5·1		3·9		3·0		2·4
	95% CI		0·8-1·4		1·5-2·6		0·8-4·2		1·7-15·5		2·4-6·4		1·4-6·7		1·6-3·5

CI denotes confidence interval, GMT geometric mean titre, IQR interquartile range (25th to 75th percentile), GMFR geometric mean fold rise, N number of subjects in the Immunogenicity Set within each visit with non-missing data.
The Immunogenicity Set consists of all enrolled participants who were randomised and received one dose of study vaccine or placebo and provided at least one valid immunogenicity response measured at least 28 days after one dose of study vaccine or placebo.
The 95% CI for GMT and GMFR were calculated based on the t-distribution on the log base 10 transformed values, then back transformed (power of 10) on the original score.

Supplementary Table 4: Microneutralisation antibody assay (titres)

		Placebo		Protein-RBD						mRNA-RBD					
				5µg		15µg		45µg		10µg		20µg		50µg	
		Day 1	Day 29	Day 1	Day 29	Day 1	Day 29	Day 1	Day 29	Day 1	Day 29	Day 1	Day 29	Day 1	Day 29
	N	12	12	16	16	8	8	8	8	16	16	8	8	8	8
Ancestral (Vic01)	GMT	59·5	60·2	52·8	109·3	256·2	453·5	85·5	299·6	44·4	127·9	108·5	279·4	138·7	322·3
	95% CI	25·8-137·5	24·2-149·9	33·0-84·5	57·4-208·0	74·4-882·7	142·5-1442·6	25·4-288·3	141·5-634·2	22·4-88·0	61·9-264·4	32·3-364·6	121·9-640·5	59·9-321·2	164·5-631·7
	Median (IQR)	54 (17-54)	40 (20-40)	60 (33-60)	139 (59-139)	298 (75-298)	932 (103-932)	114 (41-114)	341 (124-341)	29 (22-29)	135 (38-135)	167 (53-167)	346 (191-346)	161 (71-161)	322 (195-322)
	GMFR referencing Day 1		1·0		2·1		1·8		3·5		2·9		2·6		2·3
	95% CI		0·8-1·3		1·5-2·9		0·9-3·4		1·5-8·2		1·8-4·7		1·4-4·9		1·8-2·9
Beta	GMT	23·3	26·6	23·5	57·4	127·4	257·7	67·9	238·4	24·1	88·9	63·6	199·0	73·7	236·4
	95% CI	11·5-47·2	12·3-57·6	13·7-40·1	29·3-112·4	30·7-529·2	88·0-754·4	21·7-212·8	112·6-504·8	11·7-49·7	40·8-193·5	22·6-179·1	92·0-430·4	26·2-207·2	109·6-509·9
	Median (IQR)	14 (10-14)	18 (10-18)	12 (10-12)	61 (23-61)	230 (33-230)	570 (74-570)	96 (23-96)	309 (100-309)	10 (10-10)	109 (23-109)	97 (34-97)	206 (128-206)	83 (33-83)	265 (160-265)
	GMFR referencing Day 1		1·1		2·4		2·0		3·5		3·7		3·1		3·2
	95% CI		0·9-1·5		1·6-3·8		0·9-4·7		1·6-7·6		1·9-7·2		1·7-5·7		2·3-4·5
Omicron BA.5	GMT	14·6	15·9	13·2	20·7	87·6	130·1	40·8	91·3	16·5	33·6	36·7	79·5	32·7	89·8
	95% CI	9·1-23·6	9·0-28·1	10·0-17·4	14·4-29·6	22·0-347·8	32·7-517·7	15·6-106·7	29·5-282·2	10·3-26·5	16·8-67·3	15·7-85·8	33·2-190·5	12·6-85·1	32·8-245·5
	Median (IQR)	10 (10-10)	10 (10-10)	10 (10-10)	21 (10-21)	121 (22-121)	252 (28-252)	38 (17-38)	117 (40-117)	10 (10-10)	19 (10-19)	55 (12-55)	97 (47-97)	34 (10-34)	99 (47-99)
	GMFR referencing Day 1		1·1		1·6		1·5		2·2		2·0		2·2		2·7
	95% CI		0·7-1·6		1·2-2·1		0·7-3·3		1·4-3·6		1·2-3·3		1·3-3·6		1·8-4·1

CI denotes confidence interval, GMT geometric mean titre, IQR interquartile range (25th to 75th percentile), GMFR geometric mean fold rise, N number of subjects in the Immunogenicity Set within each visit with non-missing data.

The Immunogenicity Set consists of all enrolled participants who were randomised and received one dose of study vaccine or placebo and provided at least one valid immunogenicity response measured at least 28 days after one dose of study vaccine or placebo.

The 95% CI for GMT and GMFR were calculated based on the t-distribution on the log base 10 transformed values, then back transformed (power of 10) on the original score.
Assay values falling below the limit of detection were converted to half the limit of detection and this value was used in all relevant calculations.

Supplementary Table 5: Multiplex surrogate virus neutralization test (sVNT) – Method B

		Placebo			Protein-RBD									mRNA-RBD								
					5µg			15µg			45µg			10µg			20µg			50µg		
		Day 1	Day 29	Day 91	Day 1	Day 29	Day 91	Day 1	Day 29	Day 91	Day 1	Day 29	Day 91	Day 1	Day 29	Day 91	Day 1	Day 29	Day 91	Day 1	Day 29	Day 91
	N	12	12	12	16	16	16	8	8	8	8	8	8	16	16	16	8	8	8	8	8	8
WT SARS-CoV-2 RBD	GMT	227·9	324·2	609·1	234·4	357·2	623·3	646·1	1596·0	1060·6	381·7	3122·3	1487·4	184·2	852·2	718·3	434·4	1323·8	873·0	468·6	1875·7	1159·9
	95% CI	116·8-444·9	123·1-853·4	221·2-1677·5	134·6-408·3	181·8-701·7	366·4-1060·3	221·7-1883·1	597·0-4267·1	383·1-2936·3	110·5-1318·6	1413·5-6897·1	948·4-2332·8	90·3-375·5	412·4-1761·1	366·2-1408·9	164·1-1150·2	548·0-3197·5	285·0-2674·2	236·1-930·4	926·4-3797·8	751·6-1790·0
	Median (IQR)	166 (87-624)	175 (77-1485)	681 (214-1779)	259 (97-586)	273 (125-1271)	821 (271-1440)	770 (191-2206)	2678 (535-3947)	1934 (412-2811)	645 (198-1205)	2768 (1604-7185)	1464 (956-1985)	130 (78-469)	1241 (365-2675)	799 (335-2100)	553 (235-1113)	1468 (878-2955)	1131 (553-2128)	496 (224-742)	1727 (1236-2373)	1150 (851-1388)
	GMFR referencing Day 1		1·4			1·5			2·5			8·2			4·6			3·0			4·0	
	95% CI		0·9-2·1			0·7-3·2			1·3-4·6			2·8-24·2			2·5-8·5			1·3-6·9			3·1-5·2	
Alpha RBD	GMT	190·8	263·8	430·2	185·1	292·1	461·9	544·1	1275·9	826·7	302·7	2276·2	1089·9	151·5	707·0	548·0	374·4	1067·8	635·6	406·2	1482·1	935·8
	95% CI	96·4-377·4	99·7-698·3	157·4-1175·8	106·1-322·9	148·6-574·2	266·7-800·1	181·3-1633·0	507·1-3210·1	278·1-2457·6	84·4-1085·5	1114·8-4647·8	749·4-1585·1	73·3-313·2	342·5-1459·1	276·4-1086·3	142·6-983·3	420·5-2711·5	195·6-2066·1	207·8-793·8	697·5-3149·2	641·3-1365·6
	Median (IQR)	147 (70-560)	153 (61-1198)	418 (162-1293)	215 (73-467)	226 (102-1026)	641 (197-1162)	655 (166-1957)	2196 (470-3129)	1611 (310-2252)	544 (166-921)	2424 (1200-4768)	1182 (690-1365)	110 (61-415)	931 (324-2114)	630 (233-1765)	498 (207-838)	1164 (685-2584)	806 (343-1837)	427 (200-618)	1432 (894-1967)	960 (662-1189)
	GMFR referencing Day 1		1·4			1·6			2·3			7·5			4·7			2·9			3·6	
	95% CI		0·9-2·1			0·8-3·2			1·2-4·6			2·7-21·1			2·5-8·6			1·2-6·8			2·7-4·9	
Delta RBD	GMT	178·1	242·2	398·8	162·2	243·5	430·6	504·4	1095·6	750·6	289·0	1969·9	986·6	142·3	607·4	527·3	302·1	905·5	543·5	355·1	1292·7	879·2
	95% CI	91·6-346·2	94·9-618·0	155·5-1022·8	96·4-273·1	133·3-444·8	260·1-712·7	177·4-1433·9	438·3-2738·5	265·8-2119·4	85·6-975·3	1112·5-3487·9	717·0-1357·6	71·2-284·5	299·7-1230·7	272·6-1019·9	119·4-764·1	384·1-2134·7	172·8-1709·3	178·2-707·5	673·9-2480·0	585·4-1320·4
	Median (IQR)	135 (68-469)	136 (66-1048)	435 (145-1149)	177 (72-395)	183 (94-719)	528 (191-946)	616 (152-1770)	2000 (370-2390)	1398 (258-1984)	524 (164-876)	2338 (1119-3143)	996 (690-1402)	107 (62-338)	673 (267-1940)	569 (244-1522)	385 (171-699)	1029 (611-2085)	616 (338-1408)	310 (189-578)	1407 (740-1738)	902 (580-1148)
	GMFR referencing Day 1		1·4			1·5			2·2			6·8			4·3			3·0			3·6	
	95% CI		0·9-2·0			0·8-2·8			1·1-4·2			2·6-18·1			2·4-7·7			1·4-6·5			2·8-4·7	
Lambda RBD	GMT	169·9	234·4	401·8	156·8	248·9	443·0	512·1	1096·3	786·1	317·7	2313·3	1074·0	132·5	666·0	512·9	282·2	903·2	574·2	368·4	1382·3	881·5
	95% CI	82·7-348·8	88·4-621·8	150·7-1070·9	92·7-265·2	130·1-476·4	260·4-753·7	175·3-1495·9	452·2-2657·6	286·4-2157·7	94·0-1074·3	1339·5-3995·0	829·8-1390·1	65·0-270·0	314·2-1411·5	243·2-1081·6	114·8-693·4	370·2-2203·8	188·7-1747·3	173·4-783·0	679·3-2812·8	574·2-1353·4
	Median (IQR)	136 (60-472)	136 (58-1084)	424 (143-1151)	183 (80-383)	180 (98-887)	528 (199-1077)	719 (141-1779)	1994 (379-2253)	1545 (268-1918)	525 (189-859)	2525 (1573-2819)	1139 (826-1261)	95 (57-326)	849 (304-1869)	663 (246-1430)	387 (179-574)	1028 (616-2060)	633 (367-1516)	332 (195-682)	1469 (808-1797)	893 (621-1151)
	GMFR referencing Day 1		1·4			1·6			2·1			7·3			5·0			3·2			3·8	
	95% CI		1·0-2·0			0·8-3·1			1·0-4·4			2·4-22·5			2·7-9·4			1·4-7·2			3·0-4·7	
Delta Plus RBD	GMT	164·6	211·4	363·4	146·1	224·7	397·0	463·9	1056·3	662·1	247·5	2060·1	895·3	126·9	585·8	512·7	258·9	907·8	528·7	309·3	1319·3	791·7
	95% CI	87·0-311·4	84·8-527·1	142·8-924·7	88·2-241·9	121·6-415·0	244·2-645·6	160·3-1341·9	440·6-2532·4	234·9-1865·9	73·3-835·5	1095·6-3873·5	668·3-1199·4	64·6-249·3	288·6-1189·1	272·6-964·5	104·0-644·3	365·7-2253·5	166·4-1679·4	155·6-614·5	645·0-2698·2	537·4-1166·2
	Median (IQR)	133 (66-417)	119 (60-956)	419 (133-998)	155 (68-311)	166 (93-685)	472 (195-809)	562 (143-1648)	1893 (373-2256)	1181 (237-1742)	483 (141-726)	2387 (1076-3267)	939 (636-1174)	94 (53-295)	703 (253-1824)	542 (255-1308)	330 (135-611)	927 (574-2209)	570 (350-1351)	262 (173-497)	1250 (831-1803)	795 (526-1037)
	GMFR referencing Day 1		1·3			1·5			2·3			8·3			4·6			3·5			4·3	
	95% CI		0·9-1·9			0·8-2·9			1·1-4·7			3·1-22·6			2·5-8·6			1·4-8·5			3·3-5·5	
Beta RBD	GMT	125·7	166·4	241·6	118·1	207·3	312·0	474·5	977·8	630·4	217·0	1750·1	866·1	97·0	507·0	368·6	255·7	821·2	480·8	350·5	1148·8	826·5
	95% CI	66·1-239·3	61·8-448·0	86·1-678·3	62·6-222·7	104·5-411·2	182·0-535·1	153·8-1463·8	377·0-2536·2	209·7-1894·9	50·7-929·0	968·1-3163·9	688·5-1089·5	41·9-224·7	238·8-1076·4	169·8-800·2	104·4-626·0	333·0-2025·2	154·8-1493·3	177·6-691·8	546·9-2413·5	522·5-1307·3
	Median (IQR)	99 (46-283)	116 (37-794)	223 (116-806)	126 (51-329)	163 (72-638)	414 (153-637)	614 (125-1719)	1908 (295-2213)	1287 (196-1783)	445 (109-823)	2025 (1110-2615)	1001 (654-1083)	70 (34-292)	686 (216-1565)	447 (138-1185)	349 (136-593)	903 (532-2018)	592 (253-1338)	376 (181-456)	1230 (791-1588)	927 (535-1133)
	GMFR referencing Day 1		1·3			1·8			2·1			8·1			5·2			3·2			3·3	
	95% CI		0·8-2·2			0·8-3·7			1·0-4·3			2·3-28·7			2·8-9·7			1·4-7·5			2·5-4·3	
Gamma RBD	GMT	145·6	213·0	333·9	145·0	259·7	394·4	514·1	1192·3	774·4	238·7	2257·3	1048·4	129·3	658·0	493·3	303·1	999·2	605·7	401·7	1478·0	918·1
	95% CI	78·8-268·8	84·2-538·8	125·4-889·4	80·7-260·5	133·8-503·8	234·1-664·5	170·3-1551·6	477·5-2977·5	263·0-2280·0	56·1-1015·1	1111·7-4583·5	748·7-1468·0	61·0-274·2	316·3-1368·6	246·2-988·5	125·0-734·9	411·3-2427·4	194·8-1883·6	204·8-787·9	641·2-3406·9	581·6-1449·5
	Median (IQR)	113 (57-314)	137 (50-918)	276 (136-1057)	141 (61-402)	199 (99-878)	556 (174-791)	629 (142-1869)	2138 (406-2748)	1641 (252-2122)	494 (118-848)	2259 (1248-4914)	1136 (722-1256)	93 (41-347)	810 (276-2220)	539 (163-1566)	402 (160-717)	1095 (621-2489)	767 (318-1639)	424 (197-552)	1431 (926-1952)	1022 (606-1177)
	GMFR referencing Day 1		1·5			1·8			2·3			9·5			5·1			3·3			3·7	
	95% CI		0·9-2·4			0·9-3·7			1·2-4·6			2·9-31·0			2·8-9·3			1·4-7·8			2·7-5·0	
Mu RBD	GMT	112·9	157·1	228·1	96·5	179·6	288·2	402·3	961·0	586·7	192·6	1708·3	797·3	82·3	469·1	367·0	198·0	771·7	430·8	279·7	1096·2	708·4
	95% CI	57·0-223·3	60·0-411·3	78·4-663·8	51·2-181·7	84·9-379·9	169·0-491·6	131·2-1233·1	372·7-2477·7	203·0-1695·9	47·6-779·2	1021·3-2857·2	599·4-1060·5	35·6-190·5	202·3-1088·0	172·1-782·5	80·1-489·2	298·8-1992·5	129·4-1434·0	137·3-569·4	482·4-2490·9	448·0-1120·2
	Median (IQR)	98 (40-287)	104 (35-724)	200 (105-693)	110 (48-266)	130 (71-705)	397 (128-616)	543 (104-1502)	1892 (309-2116)	1163 (190-1490)	397 (92-684)	2094 (1064-2654)	840 (558-1046)	65 (32-228)	602 (207-1510)	445 (135-1041)	281 (105-430)	817 (449-2019)	506 (219-1219)	243 (148-479)	1143 (593-1502)	702 (493-949)
	GMFR referencing Day 1		1·4			1·9			2·4			8·9			5·7			3·9			3·9	
	95% CI		0·9-2·2			0·9-4·0			1·1-5·1			2·7-28·6			2·9-11·1			1·6-9·5			3·0-5·1	
BA.1 RBD	GMT	67·0	78·6	143·4	60·8	100·1	168·4	243·9	578·9	349·5	109·7	797·5	423·5	52·7	292·2	231·0	130·6	512·3	292·2	178·4	676·8	468·7
	95% CI	33·9-132·7	28·9-213·9	53·2-386·6	32·6-113·2	51·7-193·9	102·0-277·9	75·3-789·7	215·1-1558·2	121·4-1005·7	30·1-398·8	412·1-1543·6	288·7-621·1	24·1-115·5	136·4-626·2	113·3-471·1	52·2-327·2	202·7-1294·8	90·8-940·0	86·7-367·1	305·4-1499·9	276·5-794·8
	Median (IQR)	69 (33-154)	57 (28-346)	125 (60-375)	69 (26-146)	77 (40-336)	213 (92-309)	328 (73-928)	1194 (159-1444)	685 (112-918)	224 (60-315)	980 (381-1510)	434 (307-663)	32 (21-167)	387 (133-795)	241 (78-696)	148 (56-360)	509 (275-1360)	329 (157-892)	136 (98-303)	684 (413-970)	423 (311-672)
	GMFR referencing Day 1		1·2			1·6			2·4			7·3			5·5			3·9			3·8	
	95% CI		0·7-2·0			0·8-3·5			1·0-5·4			3·0-17·8			2·9-10·6			1·6-9·6			2·7-5·4	
BA.2 RBD	GMT	63·5	74·3	131·9	49·8	88·2	144·5	240·1	546·3	347·0	106·7	707·5	382·7	42·1	248·0	200·5	119·1	437·1	252·3	163·5	587·4	424·7
	95% CI	33·6-120·2	27·9-198·2	50·2-346·1	26·9-91·9	43·3-179·5	85·6-244·0	73·0-789·1	198·8-1500·7	119·6-1006·8	29·3-388·1	358·7-1395·6	252·2-580·6	18·4-96·3	115·8-531·1	98·5-408·0	45·5-312·0	172·2-1109·4	69·8-912·6	81·1-329·5	252·8-1364·9	239·4-753·5
	Median (IQR)	63 (32-121)	47 (29-302)	111 (53-368)	55 (19-122)	75 (33-311)	199 (85-286)	384 (71-867)	1134 (154-1356)	712 (114-883)	191 (55-328)	826 (322-1460)	367 (285-596)	31 (10-157)	290 (98-696)	192 (71-609)	135 (53-324)	432 (249-1124)	288 (168-835)	143 (88-296)	598 (387-923)	446 (295-619)
	GMFR referencing Day 1		1·2			1·8			2·3			6·6			5·9			3·7			3·6	
	95% CI		0·7-2·0			0·9-3·6			1·0-5·4			2·7-16·4			2·9-11·8			1·5-9·1			2·7-4·8	
BA.5 RBD	GMT	60·3	75·1	127·2	50·2	85·5	139·4	231·6	516·5	309·3	110·1	727·9	383·7	42·9	243·4	198·0	88·2	424·5	222·7	152·1	564·6	382·4
	95% CI	34·7-104·8	31·2-180·8	47·6-339·9	28·5-88·6	46·0-158·8	86·7-224·1	73·9-726·3	204·7-1303·2	113·8-840·8	28·3-428·4	344·4-1538·7	267·4-550·7	20·0-91·9	114·9-515·5	99·7-393·2	33·8-230·2	179·1-1005·8	66·3-747·7	74·7-310·0	257·6-1237·4	226·0-647·0
	Median (IQR)	59 (28-125)	59 (26-239)	101 (48-367)	54 (24-109)	80 (31-254)	173 (78-254)	297 (72-833)	945 (163-1081)	530 (97-766)	220 (46-418)	763 (387-1737)	402 (300-509)	33 (10-119)	307 (104-614)	191 (76-496)	126 (52-209)	451 (262-1056)	258 (168-532)	128 (79-294)	564 (332-926)	348 (274-632)
	GMFR referencing Day 1		1·2			1·7			2·2			6·6			5·7			4·8			3·7	
	95% CI		0·8-2·0			0·9-3·2			1·0-5·1			2·7-16·4			3·0-10·9			2·0-11·6			2·5-5·5	
XBB RBD	GMT	29·5	38·0	65·6	30·5	47·8	84·5	118·3	278·1	171·6	66·5	378·7	226·8	26·5	152·0	121·4	43·8	223·5	134·3	79·6	310·5	231·4
	95% CI	16·3-53·4	17·6-82·1	26·4-163·2	18·6-49·9	25·5-89·8	51·4-138·8	37·3-375·0	110·1-702·3	63·3-465·2	21·8-202·8	178·8-802·1	133·3-385·8	13·9-50·2	79·3-291·4	66·1-222·8	18·7-102·1	96·5-517·5	45·8-394·0	40·0-158·5	151·6-636·2	139·6-383·3
	Median (IQR)	33 (10-58)	34 (10-116)	59 (23-196)	30 (10-54)	54 (16-130)	93 (50-161)	126 (34-428)	469 (93-668)	267 (50-496)	103 (23-201)	410 (176-924)	262 (144-328)	18 (10-61)	164 (77-402)	115 (46-330)	49 (25-106)	172 (134-624)	124 (96-325)	65 (41-168)	236 (204-525)	207 (182-339)
	GMFR referencing Day 1		1·3			1·6			2·4			5·7			5·7			5·1			3·9	
	95% CI		0·9-1·9			0·8-3·0			1·0-5·7			3·1-10·5			3·2-10·4			2·0-13·2			2·8-5·5	
XBB 1.5 RBD	GMT	55·9	67·1	121·9	58·8	95·8	145·2	194·1	437·7	246·3	94·0	630·0	375·9	44·0	255·1	196·2	76·9	342·7	180·2	138·0	507·1	387·7
	95% CI	29·2-107·0	30·4-148·3	49·1-302·6	34·0-101·5	54·5-168·2	92·6-227·6	73·5-512·8	196·2-976·6	99·2-611·2	25·3-349·0	284·3-1396·1	226·2-624·4	21·3-91·1	125·9-516·9	102·4-376·1	33·4-177·2	145·6-806·2	54·5-595·7	68·4-278·6	248·9-1032·8	264·5-568·3
	Median (IQR)	66 (33-114)	60 (32-236)	105 (52-346)	70 (29-104)	76 (37-244)	172 (66-283)	186 (80-595)	688 (166-969)	340 (102-631)	160 (32-341)	601 (301-1282)	379 (230-633)	37 (10-105)	287 (119-808)	195 (79-493)	96 (49-174)	300 (203-801)	202 (119-410)	133 (65-258)	389 (288-859)	326 (294-521)
	GMFR referencing Day 1		1·2			1·6			2·3			6·7			5·8			4·5			3·7	
	95% CI		0·9-1·6			0·9-3·0			1·0-5·0			2·9-15·4			3·1-10·7			2·1-9·5			2·4-5·7	
SARS-CoV-1 RBD	GMT	18·4	19·4	28·1	17·1	22·6	24·2	32·5	62·8	35·3	24·5	95·2	61·0	16·1	49·4	34·9	21·2	58·8	43·6	26·8	81·2	57·7
	95% CI	11·9-28·5	11·9-31·8	15·7-50·3	12·4-23·5	14·3-35·7	15·5-37·8	15·8-67·0	33·5-117·6	17·3-71·9	12·8-47·1	71·0-127·5	42·6-87·4	10·8-24·1	29·1-83·9	21·7-55·9	12·1-37·1	26·7-129·8	20·3-93·5	13·7-52·3	46·1-142·9	34·8-95·8
	Median (IQR)	15 (10-31)	15 (10-43)	29 (10-58)	15 (10-27)	23 (10-46)	26 (10-39)	35 (17-68)	74 (30-109)	40 (22-66)	33 (10-49)	90 (87-123)	61 (49-81)	10 (10-33)	62 (28-110)	42 (15-59)	24 (10-41)	53 (40-125)	39 (27-79)	29 (16-34)	74 (60-94)	59 (33-92)
	GMFR referencing Day 1		1·1			1·3			1·9			3·9			3·1			2·8			3·0	
	95% CI		0·9-1·2			0·9-2·0			0·9-4·2			2·0-7·4			1·8-5·3			1·3-6·1			2·3-4·0	

CI denotes confidence interval, GMT geometric mean titre, IQR interquartile range (25th to 75th percentile), GMFR geometric mean fold rise, N number of subjects in the Immunogenicity Set within each visit with non-missing data.
The Immunogenicity Set consists of all enrolled participants who were randomised and received one dose of study vaccine or placebo and provided at least one valid immunogenicity response measured at least 28 days after one dose of study vaccine or placebo.
The 95% CI for GMT and GMFR were calculated based on the t-distribution on the log base 10 transformed values, then back transformed (power of 10) on the original score.
Assay values falling below the limit of detection were converted to half the limit of detection and this value was used in all relevant calculations.

Supplementary Table 6: Pseudovirus neutralization test (pVNT)

		Placebo		Protein-RBD						mRNA-RBD					
				5µg		15µg		45µg		10µg		20µg		50µg	
	N	Day 1	Day 29	Day 1	Day 29	Day 1	Day 29	Day 1	Day 29	Day 1	Day 29	Day 1	Day 29	Day 1	Day 29
		12	12	16	16	8	8	8	8	16	16	8	8	8	8
Ancestral (WT)	GM	3158·0	3258·2	2896·7	5565·4	8412·9	28868·7	4654·0	21053·2	2784·7	6157·5	6577·7	20941·8	6629·4	20686·6
	95% CI	1439·0-6930·7	1246·4-8517·2	1639·7-5117·4	3006·9-10300·8	2675·3-26455·1	5291·6-157496·2	1377·0-15729·8	12100·2-36630·6	1291·3-6005·3	3068·4-12356·5	2023·8-21379·0	8861·3-49491·6	2829·9-15530·3	8866·5-48263·9
	Median (IQR)	3951 (924-7734)	2466 (883-16847)	3430 (1204-7366)	8588 (2310-12446)	11759 (2620-27691)	25239 (6738-65255)	6324 (3553-12188)	23476 (13002-28769)	2285 (1004-9317)	7050 (3312-19142)	9313 (1812-23183)	18266 (13603-28555)	4420 (3687-11972)	17687 (9218-55848)
	GMFR referencing Day 1		1·0		1·9		3·4		4·5		2·2		3·2		3·1
	95% CI		0·7-1·6		1·3-2·8		0·9-13·2		1·4-15·0		1·3-3·7		1·3-8·0		1·5-6·4
Beta	GM	1504·8	1185·2	1331·5	4502·4	8649·2	13185·1	3898·3	15875·0	1097·6	6005·4	3561·5	15988·1	5122·5	13190·2
	95% CI	622·6-3637·0	394·2-3562·9	609·3-2909·7	2238·7-9054·8	1979·1-37799·0	5053·6-34401·1	676·2-22475·1	8995·0-28017·3	390·5-3085·1	3029·0-11906·5	1007·0-12596·4	5481·0-46637·8	2032·5-12910·7	7985·9-21786·1
	Median (IQR)	1951 (672-3400)	1250 (354-4418)	1310 (457-3891)	5417 (1809-15816)	12091 (3362-42735)	19121 (6375-25255)	8379 (1644-16024)	17654 (10154-22698)	848 (317-6479)	8357 (2297-14825)	5804 (2033-10056)	14646 (12440-17134)	7632 (2199-10849)	15702 (10444-19820)
	GMFR referencing Day 1		0·8		3·4		1·5		4·1		5·5		4·5		2·6
	95% CI		0·5-1·3		2·0-5·6		0·6-4·1		0·9-18·4		2·3-12·9		1·4-14·5		1·4-4·8
Omicron BQ1.1	GM	39·7	58·1	28·0	90·3	233·3	463·0	209·7	706·0	38·1	206·1	94·4	334·1	170·5	610·4
	95% CI	22·3-70·6	22·0-153·1	18·5-42·4	47·8-170·5	50·1-1086·9	117·8-1820·4	72·7-605·2	312·4-1595·6	21·9-66·4	92·2-460·6	35·6-250·6	85·2-1310·8	59·1-491·9	217·8-1710·6
	Median (IQR)	20 (20-95)	20 (20-235)	20 (20-20)	95 (20-225)	335 (58-1083)	820 (168-1495)	255 (129-397)	773 (352-1693)	20 (20-96)	184 (70-762)	107 (40-227)	234 (167-1190)	156 (93-368)	551 (412-1656)
	GMFR referencing Day 1		1·5		3·2		2·0		3·4		5·4		3·5		3·6
	95% CI		0·8-2·6		1·8-5·9		0·6-6·7		2·2-5·0		2·9-10·0		1·5-8·2		2·1-6·2
Omicron XBB	GM	29·9	38·5	30·3	65·4	139·2	334·6	107·8	225·6	30·7	90·4	55·7	260·7	88·9	224·7
	95% CI	18·6-48·3	20·1-73·8	21·1-43·4	35·4-120·8	30·3-640·6	90·3-1240·1	39·3-295·6	70·9-718·5	20·0-47·3	43·7-186·6	29·2-106·2	81·8-831·1	34·8-226·9	80·7-625·6
	Median (IQR)	20 (20-49)	20 (20-86)	20 (20-48)	61 (20-137)	146 (20-988)	670 (112-1119)	135 (52-248)	270 (96-526)	20 (20-35)	141 (20-270)	56 (35-88)	219 (155-878)	115 (32-241)	281 (154-482)
	GMFR referencing Day 1		1·3		2·2		2·4		2·1		2·9		4·7		2·5
	95% CI		0·8-2·1		1·3-3·7		0·7-8·0		1·1-3·8		1·6-5·3		2·1-10·7		1·5-4·4
Omicron XBB 1.5	GM	31·6	40·8	33·1	88·1	156·3	448·7	135·2	461·5	34·8	107·3	51·9	277·5	117·6	391·3
	95% CI	19·3-51·6	22·0-75·7	20·4-53·7	43·9-177·0	34·0-717·7	114·3-1761·2	42·7-427·6	185·2-1149·7	20·9-58·2	46·9-245·6	24·2-111·4	84·7-909·7	39·4-351·6	119·0-1287·2
	Median (IQR)	20 (20-52)	20 (20-89)	20 (20-69)	93 (20-291)	255 (20-914)	782 (175-1588)	146 (58-427)	420 (240-1063)	20 (20-61)	158 (20-359)	48 (20-133)	280 (164-861)	188 (34-306)	539 (284-901)
	GMFR referencing Day 1		1·3		2·7		2·9		3·4		3·1		5·3		3·3
	95% CI		0·8-2·1		1·5-4·8		0·7-11·5		1·7-6·9		1·6-5·9		2·5-11·5		1·5-7·3

CI denotes confidence interval, GM geometric mean, IQR interquartile range (25th to 75th percentile), GMFR geometric mean fold rise, N number of subjects in the Immunogenicity Set within each visit with non-missing data.
The Immunogenicity Set consists of all enrolled participants who were randomised and received one dose of study vaccine or placebo and provided at least one valid immunogenicity response measured at least 28 days after one dose of study vaccine or placebo.
The 95% CI for GM and GMFR were calculated based on the t-distribution on the log base 10 transformed values, then back transformed (power of 10) on the original score.
Assay values falling below the limit of detection were converted to half the limit of detection and this value was used in all relevant calculations.

Supplementary Table 7: Multiplex surrogate virus neutralization test (sVNT) – Method A

		Placebo			Protein-RBD								
					5µg			15µg			45µg		
	N	Day 1	Day 8	Day 29	Day 1	Day 8	Day 29	Day 1	Day 8	Day 29	Day 1	Day 8	Day 29
		12	12	12	16	15	16	8	8	8	8	8	8
S1 region WT SARS-CoV-2	GMT	2108.0	2020.6	1820.6	1708.0	2741.2	3163.9	5596.8	8474.9	9455.5	2762.2	9430.3	10653.3
	95% CI	1099.9-4040.0	1033.1-3952.2	884.7-3746.6	985.0-2961.7	1578.4-4760.7	1888.5-5300.9	1624.1-19286.7	3113.7-23067.0	3669.1-24367.3	879.4-8676.1	6378.3-13942.5	6843.0-16585.4
	Median (IQR)	2069 (920-5017)	2089 (831-4916)	1594 (672-5428)	1910 (793-3891)	3165 (1286-6703)	3754 (1211-6233)	9776 (1260-22381)	13490 (2493-23763)	19055 (3285-21800)	5008 (1617-6737)	10420 (7137-13930)	10463 (7146-17874)
	GMFR referencing Day 1		1.0	0.9		1.4	1.9		1.5	1.7		3.4	3.9
	95% CI		0.9-1.0	0.7-1.0		1.2-1.8	1.4-2.5		0.8-2.9	0.7-4.0		1.2-10.0	1.5-9.6
WT RBD	GMT	1915.4	1794.3	1686.3	1675.2	2688.7	3187.4	5325.2	8367.3	9844.8	2970.2	10525.6	12910.5
	95% CI	1011.3-3627.9	952.0-3382.1	836.2-3400.5	949.1-2957.0	1545.5-4677.6	1876.6-5413.5	1575.6-17997.9	3352.1-20886.2	3926.6-24682.7	930.9-9476.7	6619.9-16735.7	8054.5-20694.3
	Median (IQR)	1680 (824-4320)	1441 (776-4540)	1106 (673-5379)	1893 (824-4449)	3109 (1486-5230)	4631 (1177-6392)	9924 (1307-20081)	13933 (2526-21337)	19086 (3197-21902)	6212 (1525-7608)	12636 (7370-16008)	13290 (9107-20228)
	GMFR referencing Day 1		0.9	0.9		1.4	1.9		1.6	1.8		3.5	4.3
	95% CI		0.9-1.0	0.7-1.1		1.2-1.7	1.5-2.5		0.8-3.1	0.8-4.1		1.1-11.2	1.7-11.3
Alpha RBD	GMT	831.2	795.5	737.3	724.6	1220.6	1409.2	2547.2	3921.7	4322.2	965.4	4445.2	4961.0
	95% CI	441.6-1564.7	419.8-1507.6	365.6-1486.8	409.0-1283.6	728.9-2044.1	841.3-2360.5	814.9-7962.3	1562.7-9842.1	1787.8-10449.4	242.7-3840.9	2704.4-7306.3	3169.5-7765.1
	Median (IQR)	719 (359-1932)	655 (350-2117)	548 (321-2083)	898 (367-1644)	1476 (578-2553)	2326 (502-2874)	4107 (712-9464)	6609 (1185-10126)	8451 (1501-9160)	2007 (671-2707)	4948 (2837-7050)	5042 (3509-7620)
	GMFR referencing Day 1		1.0	0.9		1.4	1.9		1.5	1.7		4.6	5.1
	95% CI		0.9-1.1	0.7-1.1		1.1-1.8	1.4-2.7		0.8-3.0	0.8-3.7		1.2-17.8	1.6-16.6
Beta RBD	GMT	865.5	802.1	746.2	616.7	1317.3	1603.7	3085.6	5316.0	6305.5	1355.8	6820.2	7753.0
	95% CI	434.6-1723.6	400.0-1608.3	340.1-1637.0	313.0-1214.7	725.8-2390.6	933.3-2755.5	885.7-10749.1	2034.1-13892.8	2428.0-16375.1	368.6-4987.2	4197.9-11080.7	4732.1-12702.3
	Median (IQR)	829 (301-1915)	696 (313-1917)	579 (265-2366)	587 (340-1725)	1480 (609-3362)	2175 (587-3378)	6108 (774-11656)	9853 (1473-13196)	13038 (2000-14701)	2237 (962-3893)	7600 (4499-10689)	8196 (4571-12898)
	GMFR referencing Day 1		0.9	0.9		1.8	2.6		1.7	2.0		5.0	5.7
	95% CI		0.8-1.0	0.7-1.1		1.4-2.4	1.8-3.8		0.8-3.8	0.8-5.0		1.4-18.7	1.9-16.9
Delta RBD	GMT	1066.6	978.4	918.4	969.1	1479.0	1786.8	3426.6	5467.7	6551.6	1518.0	6328.2	7592.2
	95% CI	567.3-2005.3	521.5-1835.8	455.6-1851.7	587.4-1598.9	916.7-2386.1	1109.2-2878.3	1017.1-11544.3	2249.5-13290.2	2744.8-15637.9	474.0-4860.8	3829.3-10457.8	4669.9-12343.1
	Median (IQR)	1071 (440-2482)	764 (412-2549)	629 (369-2841)	1121 (464-1998)	1751 (776-3145)	2670 (732-3641)	5775 (898-13045)	9558 (1584-12736)	12015 (2247-14426)	3080 (817-3673)	7270 (4659-9471)	9181 (4381-12347)
	GMFR referencing Day 1		0.9	0.9		1.4	1.8		1.6	1.9		4.2	5.0
	95% CI		0.8-1.0	0.7-1.1		1.2-1.6	1.4-2.4		0.7-3.4	0.8-4.6		1.4-12.5	2.1-11.9
BA.2 RBD	GMT	174.4	183.6	171.7	126.3	272.8	402.9	773.8	1139.3	1457.8	398.4	1350.4	1630.4
	95% CI	91.0-334.1	91.9-366.4	76.1-387.4	70.9-225.0	139.2-534.5	231.0-702.6	165.7-3612.8	325.6-3986.6	458.5-4635.5	117.8-1347.2	616.2-2959.3	645.2-4119.9
	Median (IQR)	232 (50-323)	259 (50-373)	179 (50-372)	79 (50-292)	314 (110-561)	460 (232-651)	1148 (191-4395)	2207 (280-3537)	2340 (423-4576)	505 (172-1187)	1044 (663-2366)	1384 (690-4119)
	GMFR referencing Day 1		1.1	1.0		2.0	3.2		1.5	1.9		3.4	4.1
	95% CI		0.9-1.2	0.6-1.5		1.3-3.1	2.0-5.0		0.5-4.5	0.6-5.7		1.5-7.4	2.4-7.1
BA4/5 RBD	GMT	170.3	173.3	143.2	124.9	298.7	350.7	992.8	1802.8	2146.1	495.5	1954.3	2242.2
	95% CI	79.8-363.2	79.0-380.1	59.3-346.0	65.0-239.7	148.3-601.9	167.3-735.5	201.1-4902.7	630.6-5153.6	792.0-5815.1	145.1-1692.3	984.4-3880.0	1113.5-4514.7
	Median (IQR)	201 (50-442)	183 (50-553)	50 (50-549)	50 (50-339)	393 (50-1016)	534 (81-1195)	2055 (248-5604)	2662 (503-5565)	3450 (702-5997)	1018 (272-1163)	1947 (1265-3861)	2746 (1099-4590)
	GMFR referencing Day 1		1.0	0.8		2.3	2.8		1.8	2.2		3.9	4.5
	95% CI		0.9-1.1	0.6-1.3		1.3-3.9	1.5-5.2		0.7-5.0	0.7-6.7		1.5-10.6	2.0-10.1

		mRNA-RBD								
		10µg			20µg			50µg		
	N	Day 1	Day 8	Day 29	Day 1	Day 8	Day 29	Day 1	Day 8	Day 29
		16	16	16	8	8	8	8	8	8
S1 region WT SARS-CoV-2	GMT	1286.8	3108.8	4137.1	2244.9	4247.8	5518.4	4170.8	8090.2	9873.7
	95% CI	650.7-2544.5	1747.4-5530.9	2200.4-7778.3	949.6-5307.1	2352.7-7669.4	2545.4-11963.4	1898.8-9161.2	4041.6-16194.5	5425.3-17969.3
	Median (IQR)	813 (577-3895)	2905 (1701-8353)	4981 (2031-9973)	2136 (1188-5211)	4496 (2327-7600)	5768 (2875-12924)	4772 (2682-6600)	9978 (5763-13437)	11092 (6231-13792)
	GMFR referencing Day 1		2.4	3.2		1.9	2.5		1.9	2.4
	95% CI		1.7-3.5	2.0-5.3		1.3-2.8	1.2-5.1		1.5-2.6	1.8-3.1
WT RBD	GMT	1330.5	3297.9	4344.3	2455.9	4593.0	6619.2	4215.0	8083.5	10330.5
	95% CI	678.7-2608.1	1857.1-5856.3	2290.9-8238.3	1053.2-5726.7	2622.7-8043.4	3346.2-13093.5	2081.9-8533.9	4143.1-15771.8	5447.7-19590.0
	Median (IQR)	1095 (507-3474)	2950 (1841-8186)	5181 (2230-11310)	2947 (1216-5287)	4490 (3271-7692)	5745 (4330-15222)	4427 (2554-7014)	10235 (5167-14586)	12276 (5684-16758)
	GMFR referencing Day 1		2.5	3.3		1.9	2.7		1.9	2.5
	95% CI		1.8-3.5	2.0-5.3		1.3-2.7	1.3-5.4		1.4-2.7	2.0-3.1
Alpha RBD	GMT	495.6	1540.3	1967.9	1216.8	2312.0	3161.8	1657.2	3390.0	4023.0
	95% CI	222.3-1104.8	894.6-2652.2	1020.1-3796.6	569.4-2600.1	1356.6-3940.3	1594.9-6268.2	890.4-3084.6	1868.2-6151.3	2389.6-6773.0
	Median (IQR)	445 (263-1521)	1398 (950-3470)	2526 (1075-5040)	1605 (612-2508)	2557 (1564-3750)	2912 (2254-6704)	1837 (903-2423)	4126 (2325-5417)	3869 (2592-5669)
	GMFR referencing Day 1		3.1	4.0		1.9	2.6		2.0	2.4
	95% CI		2.1-4.6	2.5-6.4		1.3-2.7	1.4-4.8		1.5-2.8	2.0-3.0
Beta RBD	GMT	460.5	1810.3	2429.9	1427.7	3130.0	4709.8	2250.2	5126.3	6352.7
	95% CI	193.9-1094.0	964.4-3398.2	1161.2-5084.6	648.1-3144.9	1837.9-5330.6	2506.2-8850.8	1294.0-3913.2	2741.4-9586.0	3747.2-10770.1
	Median (IQR)	365 (144-1271)	2079 (933-4732)	3098 (1121-7059)	1755 (699-3291)	3428 (1841-5357)	4197 (3401-9405)	2040 (1458-3817)	6419 (3816-8777)	7380 (3865-9017)
	GMFR referencing Day 1		3.9	5.3		2.2	3.3		2.3	2.8
	95% CI		2.5-6.1	3.1-9.0		1.4-3.4	1.7-6.4		1.6-3.3	2.2-3.6
Delta RBD	GMT	736.0	2150.2	2838.0	1329.4	2665.7	3983.5	2191.2	4477.6	5785.3
	95% CI	353.1-1534.0	1221.3-3785.6	1479.1-5445.4	594.7-2971.7	1522.4-4667.7	2070.3-7664.7	1067.1-4499.5	2277.5-8803.1	3056.3-10951.0
	Median (IQR)	568 (320-2244)	2063 (1329-5418)	3317 (1461-7581)	1534 (694-3111)	3170 (1422-4495)	3913 (2195-8242)	2608 (1113-3944)	5603 (2855-8167)	7517 (2925-8803)
	GMFR referencing Day 1		2.9	3.9		2.0	3.0		2.0	2.6
	95% CI		1.9-4.5	2.2-6.9		1.3-3.0	1.5-5.9		1.4-2.9	2.1-3.4
BA.2 RBD	GMT	107.8	343.9	486.5	281.2	773.5	1147.2	389.7	992.8	1404.2
	95% CI	53.6-216.6	187.4-631.0	235.3-1005.7	103.4-765.0	365.9-1634.9	486.0-2708.2	180.5-841.7	497.4-1981.4	665.5-2962.5
	Median (IQR)	50 (50-257)	362 (181-653)	312 (250-1298)	417 (132-615)	847 (331-1724)	927 (535-2884)	532 (319-685)	879 (538-2248)	1125 (739-2721)
	GMFR referencing Day 1		3.2	4.5		2.8	4.1		2.5	3.6
	95% CI		2.0-5.2	2.4-8.6		1.6-4.6	1.9-8.7		1.6-3.9	2.0-6.5
BA4/5 RBD	GMT	150.5	492.4	631.6	265.0	979.2	1517.0	524.5	1425.7	1824.6
	95% CI	65.4-346.2	234.0-1036.1	270.8-1473.5	80.8-869.0	514.1-1865.4	757.0-3040.2	335.9-819.0	869.9-2336.7	1208.9-2753.8
	Median (IQR)	50 (50-719)	537 (279-1660)	582 (315-2408)	470 (50-852)	1198 (441-1977)	1686 (792-3173)	545 (381-724)	1527 (1003-2191)	2106 (1314-2736)
	GMFR referencing Day 1		3.3	4.2		3.7	5.7		2.7	3.5
	95% CI		1.9-5.7	2.2-8.0		1.8-7.4	2.3-14.5		1.6-4.5	2.2-5.6

CI denotes confidence interval, GMT geometric mean titre, IQR interquartile range (25th to 75th percentile), GMFR geometric mean fold rise, N number of subjects in the Immunogenicity Set within each visit with non-missing data.
The Immunogenicity Set consists of all enrolled participants who were randomised and received one dose of study vaccine or placebo and provided at least one valid immunogenicity response measured at least 28 days after one dose of study vaccine or placebo.
The 95% CI for GMT and GMFR were calculated based on the t-distribution on the log base 10 transformed values, then back transformed (power of 10) on the original score.
Assay values falling below the limit of detection were converted to half the limit of detection and this value was used in all relevant calculations.

Supplementary Table 8: T cell whole blood stain absolute number (per µL blood)

		Placebo		Protein-RBD						mRNA-RBD					
				5µg		15µg		45µg		10µg		20µg		50µg	
		**Day 1**	**Day 8**	**Day 1**	**Day 8**	**Day 1**	**Day 8**	**Day 1**	**Day 8**	**Day 1**	**Day 8**	**Day 1**	**Day 8**	**Day 1**	**Day 8**
	N	12	12	16	15	8	8	8	8	16	16	8	8	8	8
**CD38+CD27+ ASC**	GM	2·09	1·83	2·17	2·03	2·29	2·08	3·15	2·20	2·06	3·39	1·28	1·12	1·14	1·49
	95% CI	1·38-3·16	1·27-2·65	1·52-3·10	1·60-2·57	1·11-4·72	0·97-4·43	1·57-6·32	1·33-3·64	1·47-2·89	1·94-5·93	0·77-2·12	0·41-3·02	0·64-2·01	0·97-2·28
	Median (Min-Max)	2·2 (0·5-5·1)	2·3 (0·6-3·5)	2·6 (0·4-6·3)	2·3 (1·1-4·7)	3·1 (0·5-5·7)	2·4 (0·4-5·3)	2·6 (1·6-22·2)	2·0 (1·0-6·4)	2·0 (0·5-5·7)	2·9 (1·2-51·3)	1·1 (0·6-3·2)	1·0 (0·2-12·9)	1·2 (0·3-2·7)	1·5 (0·7-3·3)
	GMFR ref day 1		0·9		1·0		0·9		0·7		1·6		0·9		1·3
	95% CI		0·6-1·3		0·6-1·5		0·5-1·5		0·4-1·2		1·0-2·7		0·4-1·9		0·8-2·3
**ICOS+PD-1+ Tfh cells**	GM	6·77	9·57	8·45	6·52	6·86	10·79	8·18	15·61	5·76	11·35	7·67	11·41	8·55	9·32
	95% CI	4·87-9·39	7·74-11·83	5·97-11·98	4·23-10·03	4·98-9·47	7·69-15·13	4·95-13·54	11·19-21·79	1·63-20·38	9·13-14·10	5·65-10·40	8·42-15·47	4·86-15·04	6·19-14·02
	Median (Min-Max)	7·5 (2·8-16·0)	9·8 (5·6-17·7)	9·8 (1·3-20·0)	8·4 (1·5-23·4)	6·8 (3·5-11·4)	11·8 (5·2-16·5)	6·7 (4·8-27·7)	16·2 (8·7-26·6)	11·8 (0·0-22·2)	11·1 (6·1-30·1)	7·9 (4·4-11·6)	11·3 (7·1-19·1)	10·7 (3·2-19·0)	9·3 (5·3-21·4)
	GMFR ref day 1		1·4		0·8		1·6		1·9		2·0		1·5		1·1
	95% CI		1·1-1·8		0·4-1·3		1·1-2·3		1·0-3·7		0·6-6·7		1·1-2·0		0·5-2·3
**ICOS+PD-1+ Tfh1 cells**	GM	2·44	3·14	3·35	2·66	2·80	4·10	2·97	6·38	2·48	4·76	2·73	4·19	3·10	4·28
	95% CI	1·65-3·61	2·44-4·05	2·25-4·99	1·74-4·07	2·02-3·87	2·78-6·03	1·52-5·80	4·66-8·73	0·78-7·86	3·58-6·35	1·82-4·09	2·87-6·11	1·60-5·98	2·76-6·64
	Median (Min-Max)	2·3 (0·6-6·3)	2·8 (1·9-6·8)	3·9 (0·6-9·9)	2·9 (0·5-7·6)	3·1 (1·8-4·7)	3·9 (1·9-7·2)	2·4 (1·3-11·0)	6·3 (3·4-11·7)	4·4 (0·0-9·0)	4·3 (1·6-11·2)	2·7 (1·2-5·5)	4·3 (2·2-7·6)	4·3 (0·8-7·4)	4·1 (2·4-9·5)
	GMFR ref day 1		1·3		0·8		1·5		2·2		1·9		1·5		1·4
	95% CI		0·9-1·8		0·5-1·2		1·0-2·1		1·0-4·4		0·6-5·7		1·2-2·0		0·6-3·0
**ICOS+PD-1+ Tfh2 cells**	GM	1·89	2·21	1·92	1·65	1·79	2·63	1·84	3·53	1·27	2·05	1·74	2·07	2·18	2·01
	95% CI	1·21-2·95	1·60-3·05	1·18-3·13	1·02-2·69	1·33-2·41	1·86-3·74	1·04-3·25	2·17-5·76	0·44-3·68	1·65-2·56	1·11-2·72	1·42-3·02	1·13-4·18	1·28-3·17
	Median (Min-Max)	2·5 (0·6-4·4)	2·5 (0·8-3·8)	2·3 (0·1-6·9)	1·7 (0·4-6·1)	1·8 (1·2-3·3)	2·6 (1·6-5·2)	1·4 (1·1-8·9)	3·9 (1·2-7·7)	2·2 (0·0-5·5)	2·2 (1·0-3·7)	2·0 (0·6-3·5)	2·1 (0·9-3·8)	2·3 (0·7-5·2)	2·4 (0·9-4·3)
	GMFR ref day 1		1·2		0·9		1·5		1·9		1·6		1·2		0·9
	95% CI		0·9-1·6		0·5-1·6		1·0-2·1		0·9-4·0		0·5-4·9		0·8-1·7		0·4-2·1
**ICOS+PD-1+ Tfh17 cells**	GM	1·93	2·94	2·36	1·71	1·72	2·67	1·85	2·89	1·58	2·85	2·07	2·85	2·04	1·70
	95% CI	1·35-2·76	2·24-3·85	1·74-3·19	1·15-2·55	1·32-2·25	1·75-4·05	1·40-2·44	1·78-4·68	0·53-4·67	2·26-3·58	1·45-2·97	2·13-3·82	1·28-3·26	1·14-2·53
	Median (Min-Max)	2·5 (0·7-4·6)	3·5 (1·4-4·8)	2·5 (0·6-6·7)	2·0 (0·4-5·8)	1·6 (1·1-2·9)	2·8 (1·4-5·5)	1·8 (1·3-3·4)	3·5 (0·8-4·6)	2·9 (0·0-7·5)	2·7 (1·7-9·5)	2·3 (1·1-3·7)	2·6 (2·0-5·3)	2·3 (1·1-3·8)	1·4 (1·1-3·5)
	GMFR ref day 1		1·5		0·7		1·5		1·6		1·8		1·4		0·8
	95% CI		1·2-2·0		0·5-1·2		1·0-2·4		0·9-2·8		0·6-5·1		1·0-1·9		0·4-1·6
**CD38+HLA-DR+ CD4+ T cells**	GM	12·48	14·13	14·76	13·93	14·18	19·65	15·78	17·44	10·36	18·06	13·82	17·82	15·51	19·09
	95% CI	9·25-16·84	11·49-17·37	11·99-18·16	12·00-16·16	9·56-21·03	13·59-28·42	12·19-20·43	9·65-31·50	5·41-19·86	14·06-23·21	10·14-18·83	12·74-24·93	10·77-22·33	15·86-22·98
	Median (Min-Max)	11·9 (5·7-29·3)	14·4 (8·6-22·3)	15·1 (6·8-33·4)	13·2 (9·4-28·4)	14·8 (6·8-25·7)	20·9 (10·2-37·2)	14·2 (11·6-26·6)	20·2 (3·6-40·2)	13·7 (0·1-33·7)	16·1 (8·5-74·4)	13·7 (9·2-24·1)	16·6 (11·8-36·4)	14·3 (9·3-33·0)	19·3 (14·0-25·3)
	GMFR ref day 1		1·1		0·9		1·4		1·1		1·7		1·3		1·2
	95% CI		0·9-1·4		0·8-1·1		0·9-2·2		0·7-1·7		0·9-3·3		0·9-1·9		0·9-1·6
**CD38+HLA-DR+ CD8+ T cells**	GM	14·16	15·50	13·47	14·39	14·21	16·30	18·16	17·24	15·90	33·79	12·84	26·84	12·50	30·32
	95% CI	9·15-21·92	11·37-21·12	9·83-18·46	9·56-21·66	9·15-22·06	10·40-25·55	12·33-26·75	8·79-33·81	9·95-25·40	24·18-47·22	7·15-23·06	15·32-47·03	7·03-22·20	19·04-48·29
	Median (Min-Max)	13·6 (5·4-66·9)	17·4 (6·4-26·6)	13·6 (3·2-31·7)	12·9 (2·4-44·9)	15·8 (5·8-32·1)	17·9 (6·1-30·7)	18·7 (8·7-33·9)	18·6 (3·9-49·1)	17·9 (1·7-54·6)	35·2 (11·7-138·1)	10·4 (6·0-37·5)	33·7 (7·6-53·7)	12·8 (4·8-29·2)	27·4 (18·4-73·5)
	GMFR ref day 1		1·1		1·0		1·1		0·9		2·1		2·1		2·4
	95% CI		0·9-1·4		0·8-1·2		0·8-1·6		0·6-1·5		1·3-3·4		1·4-3·1		1·6-3·7

CI denotes confidence interval, GM geometric mean, N number of subjects in the Immunogenicity Set within each visit with non-missing data.

The 95% CI for GM and GMFR were calculated based on the t-distribution on the log base 10 transformed values, then back transformed (power of 10) on the original score.

To allow for log-transformation, a value of 0·001 was added to all values of Tfh, Tfh1, Tfh2 and Tfh17.

TFH cells were defined as CD4+CXCR5+ T cells. Within the TFH population, subsets of TFH1, TFH2 and TFH17 cells were defined as CXCR3+CCR6-, CXCR3-CCR6- and CXCR3-CCR6+, respectively.

Supplementary Table 9: T cell whole blood stain frequency

Proportion of		Placebo		Protein-RBD						mRNA-RBD					
				5µg		15µg		45µg		10µg		20µg		50µg	
		Day 1	Day 8	Day 1	Day 8	Day 1	Day 8	Day 1	Day 8	Day 1	Day 8	Day 1	Day 8	Day 1	Day 8
	N	12	12	16	15	8	8	8	8	16	16	8	8	8	8
CD38+CD27+ ASC of Total B cells	GM	1·11	0·95	1·52	1·39	1·17	1·33	1·80	1·14	1·70	2·34	0·86	0·72	0·59	0·75
	95% CI	0·82-1·49	0·64-1·40	0·97-2·38	1·02-1·89	0·68-2·02	0·82-2·14	0·83-3·89	0·63-2·06	1·15-2·51	1·31-4·17	0·50-1·47	0·29-1·78	0·34-1·02	0·47-1·19
	Median (Min-Max)	1·1 (0·4-2·1)	1·0 (0·3-2·8)	1·5 (0·5-11·5)	1·5 (0·6-4·3)	1·3 (0·3-3·4)	1·8 (0·4-2·3)	1·6 (0·6-9·1)	1·2 (0·5-3·7)	1·8 (0·3-6·2)	1·9 (0·7-15·9)	1·1 (0·4-2·2)	0·7 (0·2-5·0)	0·7 (0·2-1·4)	0·8 (0·4-1·6)
	GMFR ref day 1		0·9		1·0		1·1		0·6		1·4		0·8		1·3
	95% CI		0·5-1·4		0·7-1·6		0·7-1·7		0·3-1·2		0·8-2·4		0·4-1·8		0·8-2·1
ICOS+PD-1+ of Tfh subset	GM	4·56	5·84	6·54	5·21	3·91	6·63	6·13	11·15	4·98	9·01	6·19	7·96	7·14	6·83
	95% CI	3·47-6·00	4·64-7·35	4·71-9·09	3·41-7·95	2·75-5·56	4·85-9·08	4·56-8·24	5·71-21·78	1·45-17·07	7·73-10·51	3·67-10·44	5·40-11·74	4·49-11·36	4·47-10·42
	Median (Min-Max)	4·5 (1·9-8·6)	5·7 (2·9-10·9)	7·1 (1·1-14·2)	6·3 (1·2-12·9)	4·2 (1·9-6·7)	7·3 (3·9-12·0)	6·1 (3·9-9·3)	11·1 (5·0-47·8)	8·5 (0·0-15·3)	9·5 (5·3-15·9)	6·4 (1·8-14·2)	7·6 (3·7-17·0)	7·1 (3·4-13·7)	5·8 (4·1-15·1)
	GMFR ref day 1		1·3		0·8		1·7		1·8		1·8		1·3		1·0
	95% CI		0·9-1·8		0·5-1·3		1·2-2·4		0·8-4·0		0·5-6·2		1·1-1·5		0·5-2·0
ICOS+PD-1+ of Tfh1 subset	GM	6·22	7·67	9·32	7·55	6·60	10·07	8·55	17·16	6·50	12·34	8·23	11·04	10·61	11·69
	95% CI	4·16-9·30	5·92-9·92	6·87-12·65	4·97-11·46	4·73-9·20	7·17-14·14	5·38-13·60	9·68-30·43	1·84-22·96	10·83-14·07	5·02-13·48	7·56-16·13	5·69-19·81	7·39-18·50
	Median (Min-Max)	5·7 (1·3-12·6)	9·2 (3·1-13·9)	9·5 (2·0-19·3)	9·4 (1·5-19·0)	7·1 (3·0-10·6)	11·8 (5·1-15·4)	8·2 (4·0-21·0)	15·7 (7·1-40·3)	11·6 (0·0-19·9)	11·8 (7·9-19·4)	8·2 (3·1-17·5)	11·2 (5·4-19·5)	9·8 (3·0-30·4)	9·0 (7·0-30·6)
	GMFR ref day 1		1·2		0·8		1·5		2·0		1·9		1·3		1·1
	95% CI		0·8-1·9		0·5-1·3		1·1-2·2		0·8-5·0		0·5-6·7		1·1-1·7		0·5-2·6
ICOS+PD-1+ of Tfh2 subset	GM	3·62	3·96	4·51	3·97	2·65	4·43	3·64	6·77	3·32	5·37	4·48	4·81	4·75	3·91
	95% CI	2·39-5·47	2·72-5·77	2·86-7·10	2·62-6·00	1·90-3·71	3·28-5·99	2·70-4·90	2·99-15·32	1·02-10·78	4·41-6·53	2·82-7·12	2·98-7·76	2·95-7·66	2·47-6·17
	Median (Min-Max)	4·9 (1·1-7·6)	5·0 (1·3-10·3)	5·5 (0·3-11·1)	4·8 (0·8-12·3)	3·0 (1·4-4·9)	4·1 (2·4-7·0)	3·7 (2·0-5·6)	6·0 (3·1-53·5)	5·3 (0·0-11·4)	5·2 (3·0-9·8)	4·1 (2·1-10·8)	3·9 (2·3-11·2)	5·0 (2·4-9·1)	3·7 (1·8-9·4)
	GMFR ref day 1		1·1		0·9		1·7		1·9		1·6		1·1		0·8
	95% CI		0·8-1·6		0·5-1·7		1·2-2·4		0·8-4·2		0·5-5·5		0·9-1·3		0·4-1·7
ICOS+PD-1+ of Tfh17 subset	GM	4·81	6·31	6·78	5·16	3·58	5·82	6·15	9·32	4·86	8·47	6·23	7·10	6·45	5·07
	95% CI	3·84-6·03	4·75-8·39	5·03-9·15	3·39-7·87	2·49-5·16	4·11-8·24	4·55-8·31	4·46-19·47	1·41-16·76	6·89-10·42	3·37-11·53	4·44-11·34	4·07-10·22	3·22-7·99
	Median (Min-Max)	4·3 (3·3-10·9)	6·3 (3·1-11·3)	7·1 (1·4-16·7)	5·8 (1·1-14·4)	4·1 (1·4-5·1)	5·9 (2·9-11·0)	5·5 (3·7-10·3)	8·6 (2·7-53·9)	8·7 (0·0-17·4)	8·1 (4·4-18·5)	6·1 (1·8-20·9)	6·6 (3·1-18·3)	7·4 (3·0-11·0)	4·7 (2·6-13·1)
	GMFR ref day 1		1·3		0·8		1·6		1·5		1·7		1·1		0·8
	95% CI		1·0-1·8		0·5-1·2		1·1-2·4		0·7-3·1		0·5-6·0		0·9-1·4		0·4-1·5
CD38+HLA-DR+ of CD4+ T cells	GM	1·42	1·49	1·83	1·79	1·29	1·89	2·12	1·96	1·97	2·34	1·95	2·09	1·98	2·13
	95% CI	1·09-1·84	1·28-1·75	1·53-2·20	1·47-2·18	0·90-1·86	1·47-2·42	1·65-2·73	1·18-3·27	1·72-2·25	1·91-2·86	1·23-3·10	1·34-3·24	1·58-2·47	1·62-2·78
	Median (Min-Max)	1·6 (0·8-3·4)	1·4 (1·1-2·2)	2·1 (0·8-2·6)	1·6 (0·9-3·5)	1·1 (0·8-2·8)	1·8 (1·2-2·9)	2·2 (1·5-3·4)	2·0 (0·6-4·0)	1·9 (1·3-3·1)	2·4 (1·3-5·2)	2·1 (0·7-4·0)	2·4 (0·8-4·0)	2·0 (1·3-3·0)	2·2 (1·5-4·0)
	GMFR ref day 1		1·1		1·0		1·5		0·9		1·2		1·1		1·1
	95% CI		0·9-1·3		0·8-1·2		1·0-2·2		0·6-1·3		1·0-1·4		0·8-1·4		0·9-1·4
CD38+HLA-DR+ of CD8+ T cells	GM	3·30	3·39	3·67	4·01	3·78	4·50	4·43	3·65	4·39	7·02	4·39	7·63	3·50	6·56
	95% CI	2·26-4·82	2·45-4·69	2·74-4·91	2·77-5·82	2·78-5·14	3·58-5·65	2·46-7·99	1·61-8·29	3·26-5·91	5·23-9·43	2·74-7·03	4·98-11·67	1·98-6·21	4·50-9·56
	Median (Min-Max)	3·7 (1·2-8·6)	3·9 (1·4-7·6)	3·3 (1·7-9·7)	4·5 (1·4-14·7)	3·4 (2·7-7·7)	4·3 (3·1-6·9)	5·0 (1·3-9·3)	4·6 (0·5-11·4)	4·8 (1·7-10·6)	7·5 (3·0-19·3)	3·8 (2·6-12·3)	8·7 (3·0-13·4)	4·1 (1·2-8·3)	6·5 (3·6-14·0)
	GMFR ref day 1		1·0		1·1		1·2		0·8		1·6		1·7		1·9
	95% CI		0·8-1·3		0·9-1·3		0·9-1·6		0·6-1·1		1·2-2·2		1·1-2·7		1·3-2·7

CI denotes confidence interval, GM geometric mean, N number of subjects in the Immunogenicity Set within each visit with non-missing data.
The 95% CI for GM and GMFR were calculated based on the t-distribution on the log base 10 transformed values, then back transformed (power of 10) on the original score.
To allow for log-transformation, a value of 0·001 was added to all values of Tfh, Tfh1, Tfh2 and Tfh17.
TFH cells were defined as CD4+CXCR5+ T cells. Within the TFH population, subsets of TFH1, TFH2 and TFH17 cells were defined as CXCR3+CCR6-, CXCR3-CCR6- and CXCR3-CCR6+, respectively.

Supplementary Table 10: CD4 T cell stimulation assay activation induced marker (AIM) responses

		Placebo		Protein-RBD						mRNA-RBD					
				5µg		15µg		45µg		10µg		20µg		50µg	
		Day 1	Day 29	Day 1	Day 29	Day 1	Day 29	Day 1	Day 29	Day 1	Day 29	Day 1	Day 29	Day 1	Day 29
	N	12	12	16	16	8	8	8	8	16	16	8	8	8	8
WT RBD	GM	0·002	0·003	0·001	0·022	0·018	0·049	0·006	0·063	0·024	0·053	0·005	0·075	0·002	0·053
	95% CI	0·000-0·014	0·000-0·025	0·000-0·003	0·006-0·077	0·002-0·153	0·022-0·106	0·000-0·106	0·030-0·133	0·007-0·082	0·019-0·150	0·001-0·049	0·042-0·135	0·000-0·038	0·006-0·489
	Median (IQR)	0·01 (0·00-0·03)	0·01 (0·00-0·06)	0·00 (0·00-0·01)	0·05 (0·01-0·09)	0·03 (0·01-0·14)	0·04 (0·02-0·12)	0·03 (0·00-0·11)	0·08 (0·04-0·13)	0·07 (0·01-0·09)	0·08 (0·03-0·16)	0·01 (0·00-0·05)	0·06 (0·05-0·15)	0·01 (0·00-0·07)	0·12 (0·05-0·24)
	GMFR referencing Day 1		1·5		26·0		2·7		11·2		2·2		14·7		23·8
	95% CI		0·4-5·7		6·0-111·8		0·3-25·6		0·8-151·7		0·5-9·4		1·4-151·6		0·6-997·9
Beta RBD	GM	0·002	0·001	0·001	0·007	0·013	0·051	0·003	0·015	0·004	0·021	0·014	0·028	0·002	0·105
	95% CI	0·000-0·013	0·000-0·007	0·000-0·002	0·002-0·033	0·002-0·083	0·021-0·125	0·000-0·032	0·002-0·106	0·001-0·020	0·005-0·092	0·002-0·081	0·004-0·219	0·000-0·026	0·061-0·179
	Median (IQR)	0·00 (0·00-0·04)	0·00 (0·00-0·02)	0·00 (0·00-0·01)	0·03 (0·00-0·07)	0·02 (0·01-0·05)	0·08 (0·03-0·10)	0·01 (0·00-0·03)	0·03 (0·01-0·08)	0·02 (0·00-0·04)	0·08 (0·01-0·15)	0·03 (0·01-0·04)	0·07 (0·02-0·13)	0·00 (0·00-0·05)	0·12 (0·06-0·18)
	GMFR referencing Day 1		0·6		13·9		4·1		5·5		5·1		2·1		57·4
	95% CI		0·1-3·0		2·5-78·6		0·6-29·5		0·5-59·7		0·9-30·4		0·1-36·5		5·3-621·1
WT Spike	GM	0·161	0·141	0·076	0·122	0·154	0·241	0·040	0·187	0·127	0·123	0·175	0·203	0·145	0·228
	95% CI	0·092-0·279	0·072-0·274	0·027-0·214	0·076-0·196	0·041-0·577	0·112-0·518	0·002-0·902	0·114-0·307	0·082-0·197	0·042-0·362	0·086-0·355	0·118-0·350	0·060-0·351	0·147-0·354
	Median (IQR)	0·19 (0·12-0·28)	0·19 (0·07-0·32)	0·10 (0·08-0·22)	0·13 (0·08-0·23)	0·22 (0·10-0·52)	0·31 (0·10-0·48)	0·21 (0·09-0·30)	0·19 (0·14-0·32)	0·15 (0·07-0·24)	0·22 (0·10-0·33)	0·22 (0·10-0·33)	0·25 (0·14-0·31)	0·14 (0·08-0·41)	0·25 (0·16-0·33)
	GMFR referencing Day 1		0·9		1·6		1·6		4·7		1·0		1·2		1·6
	95% CI		0·6-1·2		0·6-4·3		0·4-5·7		0·3-72·2		0·3-2·9		0·8-1·8		0·9-2·7
Fc Region	GM	0·000	0·000	0·000	0·000	0·000	0·001	0·000	0·000	0·000	0·000	0·000	0·000	0·000	0·001
	95% CI	0·000-0·000	0·000-0·000	0·000-0·001	0·000-0·002	0·000-0·000	0·000-0·011	0·000-0·001	0·000-0·001	0·000-0·001	0·000-0·001	0·000-0·000	0·000-0·001	0·000-0·000	0·000-0·015
	Median (IQR)	0·00 (0·00-0·00)	0·00 (0·00-0·00)	0·00 (0·00-0·00)	0·00 (0·00-0·00)	0·00 (0·00-0·00)	0·00 (0·00-0·01)	0·00 (0·00-0·00)	0·00 (0·00-0·00)	0·00 (0·00-0·00)	0·00 (0·00-0·00)	0·00 (0·00-0·00)	0·00 (0·00-0·00)	0·00 (0·00-0·00)	0·00 (0·00-0·04)
	GMFR referencing Day 1		0·6		2·5		15·0		1·4		1·2		2·5		9·1
	95% CI		0·2-1·7		0·5-14·1		2·0-110·7		0·6-3·4		0·2-6·1		0·6-11·2		0·5-160·5

CI denotes confidence interval, GM geometric mean, IQR interquartile range (25th to 75th percentile), GMFR geometric mean fold rise, N number of subjects in the Immunogenicity Set within each visit with non-missing data.
The Immunogenicity Set consists of all enrolled participants who were randomised and received one dose of study vaccine or placebo and provided at least one valid immunogenicity response measured at least 28 days after one dose of study vaccine or placebo.
The 95% CI for GM and GMFR were calculated based on the t-distribution on the log base 10 transformed values, then back transformed (power of 10) on the original score.
To allow for log-transformation, a value of 0.0001 was added to all values of percentage of CD134+CD137+ double positive cells in the CD4 population.

Supplementary Table 11: CD8 T cell stimulation assay activation induced marker (AIM) responses

		Placebo		Protein-RBD						mRNA-RBD					
				5µg		15µg		45µg		10µg		20µg		50µg	
	N	Day 1	Day 29	Day 1	Day 29	Day 1	Day 29	Day 1	Day 29	Day 1	Day 29	Day 1	Day 29	Day 1	Day 29
		12	12	16	16	8	8	8	8	16	16	8	8	8	8
WT RBD (Ancestral)	GM	0·021	0·027	0·023	0·052	0·029	0·058	0·008	0·054	0·117	0·213	0·036	0·414	0·012	0·280
	95% CI	0·004-0·115	0·004-0·173	0·006-0·085	0·017-0·163	0·003-0·273	0·019-0·181	0·000-0·168	0·016-0·183	0·055-0·246	0·096-0·473	0·004-0·351	0·170-1·009	0·001-0·155	0·099-0·796
	Median (IQR)	0·04 (0·02-0·11)	0·05 (0·01-0·27)	0·04 (0·02-0·12)	0·08 (0·05-0·15)	0·07 (0·02-0·10)	0·07 (0·04-0·11)	0·05 (0·00-0·18)	0·08 (0·04-0·11)	0·09 (0·06-0·33)	0·20 (0·09-0·53)	0·08 (0·02-0·23)	0·42 (0·18-0·89)	0·04 (0·00-0·12)	0·20 (0·14-0·90)
	GMFR referencing Day 1		1·3		2·3		2·0		7·2		1·8		11·6		24·2
	95% CI		0·3-6·1		0·4-14·8		0·3-15·7		0·6-81·0		1·2-2·9		2·0-67·4		2·6-221·2
Beta RBD	GM	0·026	0·006	0·028	0·021	0·054	0·042	0·009	0·024	0·028	0·138	0·047	0·263	0·014	0·234
	95% CI	0·004-0·177	0·001-0·055	0·008-0·101	0·005-0·096	0·016-0·184	0·004-0·402	0·001-0·115	0·003-0·165	0·007-0·117	0·042-0·458	0·011-0·208	0·104-0·663	0·002-0·108	0·083-0·660
	Median (IQR)	0·07 (0·01-0·18)	0·04 (0·00-0·11)	0·05 (0·02-0·13)	0·04 (0·01-0·18)	0·05 (0·03-0·13)	0·09 (0·04-0·20)	0·02 (0·00-0·12)	0·06 (0·02-0·09)	0·05 (0·02-0·15)	0·25 (0·06-0·46)	0·09 (0·01-0·24)	0·24 (0·10-0·61)	0·02 (0·01-0·10)	0·22 (0·12-0·63)
	GMFR referencing Day 1		0·2		0·7		0·8		2·6		4·9		5·6		16·6
	95% CI		0·1-0·9		0·2-3·6		0·1-7·1		0·5-13·5		1·7-14·2		2·3-13·8		5·0-54·6
WT Spike (Ancestral)	GM	0·307	0·264	0·158	0·211	0·367	0·281	0·099	0·208	0·198	0·317	0·154	0·196	0·186	0·292
	95% CI	0·153-0·618	0·142-0·490	0·052-0·482	0·135-0·329	0·122-1·102	0·099-0·795	0·009-1·065	0·142-0·305	0·114-0·345	0·185-0·544	0·040-0·589	0·048-0·800	0·093-0·374	0·133-0·643
	Median (IQR)	0·31 (0·16-0·58)	0·26 (0·14-0·61)	0·30 (0·13-0·47)	0·19 (0·11-0·35)	0·48 (0·12-1·01)	0·32 (0·14-0·62)	0·22 (0·13-0·41)	0·24 (0·16-0·28)	0·20 (0·12-0·42)	0·27 (0·15-0·66)	0·25 (0·06-0·48)	0·30 (0·13-0·47)	0·15 (0·10-0·42)	0·29 (0·12-0·65)
	GMFR referencing Day 1		0·9		1·3		0·8		2·1		1·6		1·3		1·6
	95% CI		0·6-1·3		0·5-3·6		0·4-1·6		0·3-17·5		1·2-2·2		0·3-4·7		1·1-2·3
Fc Region	GM	0·006	0·001	0·004	0·000	0·001	0·003	0·000	0·000	0·004	0·004	0·015	0·001	0·001	0·001
	95% CI	0·001-0·041	0·000-0·005	0·001-0·018	0·000-0·002	0·000-0·006	0·000-0·036	0·000-0·000	0·000-0·003	0·001-0·020	0·001-0·017	0·005-0·043	0·000-0·006	0·000-0·015	0·000-0·011
	Median (IQR)	0·02 (0·00-0·04)	0·00 (0·00-0·01)	0·01 (0·00-0·03)	0·00 (0·00-0·00)	0·00 (0·00-0·01)	0·01 (0·00-0·02)	0·00 (0·00-0·00)	0·00 (0·00-0·00)	0·02 (0·00-0·06)	0·01 (0·00-0·05)	0·01 (0·01-0·04)	0·00 (0·00-0·02)	0·00 (0·00-0·03)	0·00 (0·00-0·02)
	GMFR referencing Day 1		0·1		0·1		4·6		1·8		0·9		0·0		0·8
	95% CI		0·0-0·6		0·0-1·0		0·6-36·4		0·4-9·3		0·1-8·1		0·0-0·4		0·0-16·5

CI denotes confidence interval, GM geometric mean, IQR interquartile range (25th to 75th percentile), GMFR geometric mean fold rise, N number of subjects in the Immunogenicity Set within each visit with non-missing data.
The Immunogenicity Set consists of all enrolled participants who were randomised and received one dose of study vaccine or placebo and provided at least one valid immunogenicity response measured at least 28 days after one dose of study vaccine or placebo.
The 95% CI for GM and GMFR were calculated based on the t-distribution on the log base 10 transformed values, then back transformed (power of 10) on the original score.
To allow for log-transformation, a value of 0.0001 was added to all values of percentage of CD69+CD137+ double positive cells in the CD8 population.

Supplementary Table 12: CD4 T cell cytokine responses by intracellular staining (ICS)

		Placebo		Protein-RBD						mRNA-RBD					
				5μg		15μg		45μg		10μg		20μg		50μg	
	N	Day 1	Day 29	Day 1	Day 29	Day 1	Day 29	Day 1	Day 29	Day 1	Day 29	Day 1	Day 29	Day 1	Day 29
		12	12	16	16	8	8	8	8	16	16	8	8	8	8
Total IFNg+															
WT RBD	GMT	0·005	0·002	0·001	0·003	0·002	0·015	0·095	0·091	0·002	0·005	0·002	0·019	0·025	0·040
	95% CI	0·001-0·039	0·000-0·033	0·000-0·005	0·000-0·019	0·000-0·046	0·001-0·287	0·042-0·218	0·067-0·124	0·000-0·018	0·001-0·042	0·000-0·101	0·001-0·317	0·002-0·374	0·002-0·701
	Median (IQR)	0·01 (0·01-0·02)	0·02 (0·00-0·04)	0·00 (0·00-0·03)	0·01 (0·00-0·03)	0·01 (0·00-0·04)	0·04 (0·00-0·24)	0·12 (0·06-0·16)	0·09 (0·08-0·12)	0·02 (0·00-0·04)	0·02 (0·00-0·06)	0·02 (0·00-0·10)	0·06 (0·01-0·14)	0·09 (0·04-0·12)	0·09 (0·07-0·22)
	GMFR referencing Day 1		0·4		5·2		6·3		1·0		2·8		8·7		1·6
	95% CI		0·0-7·0		0·4-69·7		0·2-216·7		0·5-1·7		0·2-43·3		0·6-120·6		0·0-61·7
Beta RBD	GMT	0·001	0·002	0·000	0·001	0·001	0·006	0·102	0·128	0·001	0·011	0·003	0·007	0·021	0·010
	95% CI	0·000-0·016	0·000-0·032	0·000-0·004	0·000-0·008	0·000-0·038	0·000-0·215	0·041-0·259	0·083-0·197	0·000-0·010	0·002-0·055	0·000-0·069	0·000-0·190	0·001-0·327	0·000-0·384
	Median (IQR)	0·02 (0·00-0·04)	0·03 (0·00-0·04)	0·00 (0·00-0·02)	0·00 (0·00-0·02)	0·01 (0·00-0·03)	0·04 (0·00-0·18)	0·17 (0·06-0·21)	0·12 (0·08-0·20)	0·01 (0·00-0·04)	0·02 (0·01-0·08)	0·01 (0·00-0·04)	0·05 (0·02-0·06)	0·06 (0·02-0·15)	0·06 (0·01-0·17)
	GMFR referencing Day 1		2·1		2·3		4·5		1·3		11·0		2·1		0·5
	95% CI		0·1-32·3		0·1-38·8		0·0-1058·3		0·6-2·7		0·9-133·1		0·9-5·1		0·0-35·5
WT Spike	GMT	0·001	0·005	0·005	0·009	0·004	0·041	0·012	0·006	0·012	0·043	0·032	0·015	0·013	0·018
	95% CI	0·000-0·017	0·001-0·036	0·001-0·028	0·002-0·044	0·000-0·097	0·018-0·094	0·001-0·160	0·000-0·181	0·004-0·040	0·029-0·065	0·016-0·064	0·001-0·243	0·001-0·148	0·001-0·250
	Median (IQR)	0·01 (0·00-0·05)	0·02 (0·00-0·03)	0·02 (0·01-0·04)	0·02 (0·00-0·05)	0·02 (0·00-0·06)	0·05 (0·03-0·07)	0·03 (0·01-0·06)	0·03 (0·01-0·08)	0·02 (0·01-0·05)	0·04 (0·02-0·08)	0·03 (0·02-0·07)	0·08 (0·02-0·09)	0·03 (0·02-0·04)	0·05 (0·02-0·08)
	GMFR referencing Day 1		3·5		1·8		10·0		0·5		3·6		0·5		1·4
	95% CI		0·2-65·5		0·2-13·9		0·8-122·1		0·1-3·7		1·3-9·9		0·0-7·0		0·0-41·7
Human IgG1 Fc Region	GMT	0·001	0·002	0·000	0·003	0·001	0·008	0·086	0·092	0·001	0·002	0·001	0·010	0·023	0·017
	95% CI	0·000-0·015	0·000-0·028	0·000-0·003	0·000-0·018	0·000-0·044	0·000-0·130	0·048-0·155	0·064-0·131	0·000-0·006	0·000-0·015	0·000-0·040	0·001-0·121	0·001-0·341	0·001-0·308
	Median (IQR)	0·01 (0·00-0·02)	0·02 (0·00-0·04)	0·00 (0·00-0·02)	0·01 (0·00-0·03)	0·01 (0·00-0·07)	0·01 (0·00-0·16)	0·09 (0·06-0·16)	0·09 (0·07-0·12)	0·01 (0·00-0·02)	0·01 (0·00-0·06)	0·01 (0·00-0·05)	0·03 (0·01-0·05)	0·07 (0·02-0·15)	0·06 (0·01-0·14)
	GMFR referencing Day 1		2·0		5·8		5·8		1·1		2·6		8·3		0·7
	95% CI		0·0-97·5		1·1-29·7		0·4-79·4		0·7-1·7		0·2-34·3		0·2-397·1		0·0-35·8
Total TNF+															
WT RBD	GMT	0·404	0·386	0·295	0·362	0·242	0·397	1·068	1·075	0·453	0·509	0·588	0·739	0·916	0·878
	95% CI	0·225-0·725	0·215-0·695	0·172-0·504	0·220-0·594	0·077-0·764	0·160-0·985	0·565-2·019	0·634-1·820	0·319-0·644	0·376-0·689	0·326-1·062	0·449-1·216	0·502-1·674	0·437-1·767
	Median (IQR)	0·54 (0·20-0·70)	0·46 (0·21-0·63)	0·41 (0·17-0·52)	0·44 (0·30-0·70)	0·39 (0·16-0·60)	0·55 (0·19-0·86)	1·25 (0·91-1·83)	1·22 (1·04-1·39)	0·52 (0·33-0·68)	0·51 (0·33-0·79)	0·51 (0·33-1·05)	0·59 (0·48-1·07)	0·84 (0·55-1·81)	0·77 (0·43-1·95)
	GMFR referencing Day 1		1·0		1·2		1·6		1·0		1·1		1·3		1·0
	95% CI		0·8-1·2		1·1-1·4		0·8-3·5		0·8-1·3		1·0-1·3		0·6-2·6		0·7-1·3
Beta RBD	GMT	0·206	0·218	0·093	0·062	0·112	0·040	0·185	0·073	0·180	0·216	0·195	0·213	0·232	0·249
	95% CI	0·090-0·471	0·102-0·465	0·022-0·390	0·010-0·408	0·036-0·345	0·002-0·720	0·053-0·643	0·003-1·640	0·106-0·306	0·137-0·339	0·084-0·451	0·106-0·429	0·066-0·817	0·096-0·643
	Median (IQR)	0·34 (0·09-0·53)	0·36 (0·11-0·43)	0·23 (0·05-0·32)	0·22 (0·07-0·36)	0·11 (0·05-0·37)	0·11 (0·04-0·24)	0·21 (0·09-0·64)	0·15 (0·09-0·66)	0·21 (0·11-0·37)	0·28 (0·14-0·37)	0·22 (0·12-0·44)	0·24 (0·11-0·44)	0·27 (0·12-0·58)	0·31 (0·11-0·55)
	GMFR referencing Day 1		1·1		0·7		0·4		0·4		1·2		1·1		1·1
	95% CI		0·9-1·3		0·2-1·9		0·0-5·2		0·0-3·1		1·0-1·4		0·8-1·4		0·6-1·8
WT Spike	GMT	0·021	0·028	0·011	0·017	0·023	0·015	0·013	0·023	0·026	0·037	0·040	0·019	0·007	0·006
	95% CI	0·012-0·038	0·017-0·048	0·003-0·036	0·006-0·052	0·009-0·057	0·001-0·191	0·001-0·156	0·007-0·071	0·016-0·042	0·024-0·056	0·021-0·076	0·001-0·243	0·001-0·087	0·000-0·161
	Median (IQR)	0·02 (0·01-0·04)	0·03 (0·01-0·05)	0·02 (0·01-0·03)	0·03 (0·02-0·04)	0·03 (0·01-0·06)	0·06 (0·02-0·07)	0·03 (0·02-0·05)	0·03 (0·01-0·07)	0·03 (0·01-0·04)	0·04 (0·02-0·05)	0·03 (0·02-0·08)	0·05 (0·03-0·08)	0·02 (0·01-0·05)	0·03 (0·01-0·06)
	GMFR referencing Day 1		1·3		1·6		0·7		1·8		1·4		0·5		0·9
	95% CI		1·0-1·8		0·3-9·1		0·1-7·4		0·3-11·2		1·0-2·0		0·0-4·8		0·0-17·1
Human IgG1 Fc Region	GMT	0·213	0·219	0·067	0·067	0·040	0·048	0·192	0·207	0·205	0·193	0·186	0·142	0·220	0·196
	95% CI	0·094-0·479	0·090-0·530	0·010-0·443	0·010-0·445	0·002-0·780	0·003-0·902	0·053-0·703	0·071-0·609	0·115-0·367	0·100-0·375	0·055-0·633	0·026-0·763	0·059-0·829	0·055-0·701
	Median (IQR)	0·41 (0·10-0·48)	0·38 (0·09-0·50)	0·29 (0·08-0·39)	0·27 (0·08-0·36)	0·09 (0·04-0·36)	0·10 (0·08-0·27)	0·24 (0·08-0·76)	0·17 (0·08-0·73)	0·27 (0·14-0·39)	0·30 (0·17-0·38)	0·27 (0·12-0·51)	0·27 (0·11-0·48)	0·25 (0·11-0·66)	0·25 (0·12-0·56)
	GMFR referencing Day 1		1·0		1·0		1·2		1·1		0·9		0·8		0·9
	95% CI		0·7-1·4		0·9-1·2		0·0-69·3		0·8-1·5		0·8-1·2		0·4-1·4		0·5-1·5
Total MIP1b+															
WT RBD	GMT	0·007	0·001	0·028	0·012	0·001	0·008	0·358	0·320	0·085	0·083	0·061	0·094	0·176	0·191
	95% CI	0·000-0·093	0·000-0·015	0·010-0·079	0·001-0·118	0·000-0·073	0·000-0·331	0·145-0·882	0·138-0·743	0·021-0·336	0·020-0·351	0·003-1·286	0·004-2·282	0·062-0·503	0·071-0·515
	Median (IQR)	0·02 (0·01-0·09)	0·01 (0·00-0·02)	0·05 (0·01-0·11)	0·12 (0·01-0·18)	0·02 (0·00-0·11)	0·04 (0·00-0·21)	0·50 (0·26-0·74)	0·35 (0·20-0·60)	0·17 (0·10-0·26)	0·18 (0·06-0·29)	0·15 (0·07-0·43)	0·29 (0·13-0·51)	0·25 (0·08-0·48)	0·18 (0·08-0·51)
	GMFR referencing Day 1		0·1		0·4		7·2		0·9		1·0		1·5		1·1
	95% CI		0·0-4·4		0·1-2·2		0·4-117·7		0·5-1·6		0·1-7·8		0·6-3·9		0·6-1·9
Beta RBD	GMT	0·000	0·000	0·000	0·000	0·000	0·000	0·008	0·003	0·000	0·000	0·000	0·000	0·000	0·000
	95% CI	0·000-0·000	0·000-0·000	0·000-0·000	0·000-0·000	0·000-0·000	0·000-0·000	0·001-0·086	0·000-0·066	0·000-0·000	0·000-0·000	0·000-0·000	0·000-0·000	0·000-0·005	0·000-0·001
	Median (IQR)	0·00 (0·00-0·00)	0·00 (0·00-0·00)	0·00 (0·00-0·00)	0·00 (0·00-0·00)	0·00 (0·00-0·00)	0·00 (0·00-0·00)	0·01 (0·01-0·04)	0·01 (0·00-0·03)	0·00 (0·00-0·00)	0·00 (0·00-0·00)	0·00 (0·00-0·00)	0·00 (0·00-0·00)	0·00 (0·00-0·02)	0·00 (0·00-0·00)
	GMFR referencing Day 1		0·3		0·7		1·0		0·4		0·5		1·0		0·3
	95% CI		0·1-1·5		0·3-1·5		1·0-1·0		0·1-2·7		0·2-1·8		1·0-1·0		0·0-10·3
WT Spike	GMT	0·001	0·001	0·001	0·001	0·001	0·002	0·000	0·000	0·002	0·002	0·065	0·004	0·001	0·000
	95% CI	0·000-0·009	0·000-0·016	0·000-0·005	0·000-0·007	0·000-0·025	0·000-0·077	0·000-0·013	0·000-0·008	0·000-0·012	0·000-0·016	0·032-0·130	0·000-0·109	0·000-0·020	0·000-0·016
	Median (IQR)	0·01 (0·00-0·02)	0·01 (0·00-0·03)	0·00 (0·00-0·01)	0·00 (0·00-0·03)	0·01 (0·00-0·02)	0·03 (0·00-0·05)	0·00 (0·00-0·02)	0·00 (0·00-0·01)	0·01 (0·00-0·01)	0·01 (0·00-0·07)	0·05 (0·03-0·16)	0·01 (0·00-0·13)	0·00 (0·00-0·01)	0·00 (0·00-0·04)
	GMFR referencing Day 1		1·4		1·0		2·0		0·8		0·8		0·1		0·4
	95% CI		0·0-54·1		0·1-14·3		0·0-86·8		0·0-66·4		0·1-7·4		0·0-1·5		0·0-16·5
Human IgG1 Fc Region	GMT	0·000	0·000	0·000	0·000	0·000	0·000	0·007	0·003	0·000	0·000	0·000	0·000	0·000	0·000
	95% CI	0·000-0·000	0·000-0·000	0·000-0·000	0·000-0·000	0·000-0·000	0·000-0·000	0·001-0·071	0·000-0·071	0·000-0·000	0·000-0·000	0·000-0·000	0·000-0·000	0·000-0·004	0·000-0·003
	Median (IQR)	0·00 (0·00-0·00)	0·00 (0·00-0·00)	0·00 (0·00-0·00)	0·00 (0·00-0·00)	0·00 (0·00-0·00)	0·00 (0·00-0·00)	0·01 (0·01-0·03)	0·02 (0·00-0·03)	0·00 (0·00-0·00)	0·00 (0·00-0·00)	0·00 (0·00-0·00)	0·00 (0·00-0·00)	0·00 (0·00-0·01)	0·00 (0·00-0·01)
	GMFR referencing Day 1		0·6		0·5		1·0		0·5		0·8		1·0		0·9
	95% CI		0·1-4·0		0·1-2·6		1·0-1·0		0·1-4·6		0·1-5·9		1·0-1·0		0·0-65·8
Total IL5+															
WT RBD	GMT	0·000	0·001	0·001	0·002	0·000	0·001	0·001	0·000	0·002	0·001	0·001	0·001	0·001	0·002
	95% CI	0·000-0·003	0·000-0·007	0·000-0·005	0·000-0·014	0·000-0·001	0·000-0·013	0·000-0·016	0·000-0·003	0·000-0·010	0·000-0·008	0·000-0·021	0·000-0·015	0·000-0·014	0·000-0·024
	Median (IQR)	0·00 (0·00-0·01)	0·00 (0·00-0·02)	0·00 (0·00-0·01)	0·01 (0·00-0·02)	0·00 (0·00-0·01)	0·00 (0·00-0·02)	0·01 (0·00-0·01)	0·00 (0·00-0·00)	0·01 (0·00-0·02)	0·01 (0·00-0·02)	0·01 (0·00-0·02)	0·01 (0·00-0·01)	0·00 (0·00-0·01)	0·01 (0·00-0·01)
	GMFR referencing Day 1		2·1		2·5		11·3		0·2		0·5		0·6		2·4
	95% CI		0·1-43·5		0·2-42·6		0·5-272·4		0·0-5·6		0·0-9·9		0·0-31·7		0·3-21·5
Beta RBD	GMT	0·000	0·000	0·002	0·004	0·000	0·001	0·000	0·001	0·001	0·003	0·003	0·001	0·001	0·001
	95% CI	0·000-0·003	0·000-0·003	0·000-0·017	0·001-0·021	0·000-0·006	0·000-0·018	0·000-0·003	0·000-0·012	0·000-0·010	0·000-0·018	0·000-0·019	0·000-0·024	0·000-0·006	0·000-0·017
	Median (IQR)	0·00 (0·00-0·01)	0·00 (0·00-0·01)	0·01 (0·00-0·02)	0·01 (0·00-0·02)	0·00 (0·00-0·01)	0·00 (0·00-0·02)	0·00 (0·00-0·01)	0·00 (0·00-0·01)	0·01 (0·00-0·02)	0·01 (0·00-0·02)	0·01 (0·00-0·01)	0·01 (0·00-0·02)	0·00 (0·00-0·00)	0·00 (0·00-0·01)
	GMFR referencing Day 1		1·1		1·6		4·9		3·4		2·0		0·4		1·1
	95% CI		0·0-30·8		0·1-22·6		0·0-720·2		0·4-31·1		0·1-29·9		0·0-7·5		0·0-30·5
WT Spike	GMT	0·000	0·000	0·000	0·001	0·000	0·000	0·000	0·000	0·000	0·000	0·000	0·000	0·001	0·000
	95% CI	0·000-0·001	0·000-0·003	0·000-0·001	0·000-0·004	0·000-0·002	0·000-0·009	0·000-0·000	0·000-0·002	0·000-0·002	0·000-0·001	0·000-0·004	0·000-0·003	0·000-0·010	0·000-0·003
	Median (IQR)	0·00 (0·00-0·00)	0·00 (0·00-0·01)	0·00 (0·00-0·01)	0·00 (0·00-0·01)	0·00 (0·00-0·01)	0·00 (0·00-0·01)	0·00 (0·00-0·00)	0·00 (0·00-0·00)	0·00 (0·00-0·01)	0·00 (0·00-0·01)	0·00 (0·00-0·00)	0·00 (0·00-0·01)	0·00 (0·00-0·01)	0·00 (0·00-0·01)
	GMFR referencing Day 1		2·1		5·3		3·9		4·8		0·4		0·6		0·4
	95% CI		0·1-66·4		0·3-98·7		0·0-354·0		0·7-31·7		0·0-7·7		0·0-50·0		0·0-9·0
Human IgG1 Fc Region	GMT	0·002	0·000	0·001	0·002	0·001	0·005	0·000	0·000	0·001	0·004	0·002	0·003	0·001	0·000
	95% CI	0·000-0·011	0·000-0·004	0·000-0·006	0·000-0·011	0·000-0·010	0·001-0·048	0·000-0·005	0·000-0·013	0·000-0·004	0·001-0·016	0·000-0·029	0·000-0·027	0·000-0·008	0·000-0·010
	Median (IQR)	0·00 (0·00-0·01)	0·00 (0·00-0·02)	0·01 (0·00-0·01)	0·01 (0·00-0·01)	0·00 (0·00-0·01)	0·01 (0·01-0·02)	0·00 (0·00-0·01)	0·00 (0·00-0·03)	0·00 (0·00-0·02)	0·01 (0·00-0·02)	0·01 (0·00-0·02)	0·01 (0·00-0·02)	0·00 (0·00-0·01)	0·00 (0·00-0·01)
	GMFR referencing Day 1		0·2		2·1		9·6		1·2		6·5		1·6		0·7
	95% CI		0·0-2·4		0·2-21·3		0·2-559·4		0·0-62·0		1·1-37·8		0·1-50·7		0·0-29·3
Total IL13+															
WT RBD	GMT	0·005	0·004	0·000	0·000	0·000	0·002	0·026	0·010	0·004	0·008	0·004	0·011	0·009	0·009
	95% CI	0·001-0·033	0·001-0·027	0·000-0·001	0·000-0·003	0·000-0·007	0·000-0·031	0·013-0·052	0·001-0·113	0·001-0·017	0·002-0·023	0·000-0·038	0·005-0·021	0·001-0·090	0·001-0·094
	Median (IQR)	0·02 (0·01-0·02)	0·02 (0·00-0·02)	0·00 (0·00-0·01)	0·00 (0·00-0·01)	0·00 (0·00-0·01)	0·01 (0·00-0·01)	0·04 (0·01-0·05)	0·02 (0·01-0·04)	0·01 (0·01-0·02)	0·01 (0·00-0·02)	0·01 (0·00-0·02)	0·01 (0·01-0·02)	0·02 (0·01-0·04)	0·03 (0·01-0·04)
	GMFR referencing Day 1		0·8		2·0		6·6		0·4		1·7		2·9		1·1
	95% CI		0·1-11·3		0·2-16·7		0·6-72·2		0·0-4·5		0·3-9·3		0·2-35·3		0·0-29·1
Beta RBD	GMT	0·003	0·005	0·002	0·003	0·003	0·016	0·033	0·027	0·002	0·018	0·017	0·023	0·006	0·006
	95% CI	0·000-0·026	0·001-0·033	0·000-0·010	0·001-0·013	0·000-0·067	0·012-0·022	0·016-0·068	0·011-0·066	0·000-0·011	0·012-0·028	0·006-0·045	0·011-0·048	0·001-0·058	0·001-0·055
	Median (IQR)	0·01 (0·00-0·02)	0·02 (0·01-0·02)	0·01 (0·00-0·01)	0·01 (0·00-0·02)	0·01 (0·01-0·02)	0·02 (0·01-0·02)	0·04 (0·03-0·06)	0·04 (0·02-0·05)	0·01 (0·00-0·02)	0·02 (0·01-0·03)	0·02 (0·01-0·04)	0·02 (0·01-0·05)	0·01 (0·01-0·03)	0·01 (0·01-0·03)
	GMFR referencing Day 1		1·8		1·5		5·1		0·8		8·9		1·4		1·0
	95% CI		0·1-39·7		0·2-12·5		0·3-102·2		0·3-2·4		2·1-38·1		0·5-3·7		0·0-34·6
WT Spike	GMT	0·000	0·000	0·000	0·000	0·000	0·000	0·000	0·000	0·000	0·000	0·000	0·000	0·000	0·000
	95% CI	0·000-0·001	0·000-0·002	0·000-0·000	0·000-0·001	0·000-0·002	0·000-0·003	0·000-0·006	0·000-0·004	0·000-0·000	0·000-0·002	0·000-0·003	0·000-0·001	0·000-0·003	0·000-0·003
	Median (IQR)	0·00 (0·00-0·01)	0·00 (0·00-0·01)	0·00 (0·00-0·00)	0·00 (0·00-0·01)	0·00 (0·00-0·00)	0·00 (0·00-0·01)	0·00 (0·00-0·01)	0·00 (0·00-0·00)	0·00 (0·00-0·00)	0·00 (0·00-0·01)	0·00 (0·00-0·00)	0·00 (0·00-0·00)	0·00 (0·00-0·01)	0·00 (0·00-0·00)
	GMFR referencing Day 1		1·9		10·3		1·4		0·5		3·2		0·3		1·0
	95% CI		0·1-37·8		1·1-96·2		0·1-18·5		0·0-34·7		0·2-48·4		0·0-4·8		0·1-10·9
Human IgG1 Fc Region	GMT	0·009	0·004	0·002	0·001	0·004	0·013	0·030	0·034	0·001	0·007	0·006	0·013	0·006	0·008
	95% CI	0·002-0·034	0·001-0·022	0·000-0·009	0·000-0·006	0·000-0·040	0·005-0·035	0·020-0·045	0·024-0·047	0·000-0·004	0·002-0·030	0·001-0·053	0·001-0·167	0·001-0·061	0·001-0·084
	Median (IQR)	0·02 (0·01-0·02)	0·01 (0·00-0·02)	0·01 (0·00-0·01)	0·01 (0·00-0·02)	0·01 (0·00-0·02)	0·01 (0·01-0·02)	0·03 (0·02-0·04)	0·03 (0·02-0·04)	0·01 (0·00-0·02)	0·02 (0·01-0·02)	0·01 (0·01-0·02)	0·03 (0·01-0·06)	0·02 (0·01-0·03)	0·02 (0·01-0·04)
	GMFR referencing Day 1		0·4		0·4		3·2		1·1		11·7		2·3		1·3
	95% CI		0·0-4·5		0·1-2·0		0·4-25·2		0·8-1·6		0·9-155·6		1·0-5·1		0·0-48·5
IFNg+															
WT RBD	GMT	0·002	0·001	0·001	0·005	0·008	0·020	0·029	0·041	0·001	0·006	0·007	0·029	0·017	0·062
	95% CI	0·000-0·015	0·000-0·008	0·000-0·007	0·001-0·023	0·001-0·086	0·003-0·159	0·010-0·086	0·028-0·059	0·000-0·007	0·001-0·030	0·001-0·076	0·012-0·071	0·001-0·219	0·028-0·141
	Median (IQR)	0·01 (0·00-0·01)	0·00 (0·00-0·03)	0·01 (0·00-0·02)	0·02 (0·00-0·02)	0·01 (0·01-0·04)	0·03 (0·01-0·13)	0·03 (0·01-0·10)	0·04 (0·03-0·06)	0·00 (0·00-0·02)	0·01 (0·00-0·02)	0·02 (0·00-0·03)	0·03 (0·01-0·06)	0·04 (0·03-0·07)	0·08 (0·02-0·14)
	GMFR referencing Day 1		0·4		5·6		2·5		1·4		7·9		4·3		3·7
	95% CI		0·1-1·7		1·2-26·0		0·1-69·2		0·6-3·1		1·0-60·1		0·8-23·3		0·4-32·2
Beta RBD	GMT	0·336	0·000	0·227	0·004	0·179	0·007	0·728	0·082	0·303	0·002	0·414	0·026	0·674	0·068
	95% CI	0·197-0·574	0·000-0·007	0·121-0·427	0·001-0·023	0·059-0·544	0·000-0·240	0·379-1·395	0·050-0·137	0·203-0·452	0·000-0·015	0·236-0·727	0·010-0·065	0·331-1·369	0·031-0·151
	Median (IQR)	0·50 (0·16-0·61)	0·00 (0·00-0·03)	0·35 (0·12-0·48)	0·01 (0·00-0·03)	0·18 (0·12-0·52)	0·03 (0·00-0·21)	0·96 (0·44-1·35)	0·08 (0·05-0·14)	0·35 (0·23-0·47)	0·01 (0·00-0·05)	0·44 (0·21-0·62)	0·03 (0·02-0·06)	0·76 (0·30-1·30)	0·08 (0·04-0·12)
	GMFR referencing Day 1		0·0		0·0		0·0		0·1		0·0		0·1		0·1
	95% CI		0·0-0·0		0·0-0·1		0·0-2·4		0·0-0·3		0·0-0·1		0·0-0·2		0·0-0·3
WT Spike	GMT	0·000	0·000	0·001	0·003	0·003	0·008	0·007	0·004	0·001	0·009	0·001	0·013	0·013	0·019
	95% CI	0·000-0·005	0·000-0·003	0·000-0·009	0·000-0·020	0·000-0·054	0·001-0·088	0·001-0·078	0·000-0·089	0·000-0·007	0·002-0·040	0·000-0·018	0·001-0·152	0·005-0·035	0·005-0·072
	Median (IQR)	0·00 (0·00-0·02)	0·00 (0·00-0·01)	0·01 (0·00-0·02)	0·01 (0·00-0·04)	0·02 (0·00-0·03)	0·02 (0·01-0·03)	0·01 (0·01-0·04)	0·02 (0·00-0·05)	0·00 (0·00-0·02)	0·02 (0·01-0·04)	0·00 (0·00-0·02)	0·04 (0·02-0·05)	0·02 (0·01-0·02)	0·03 (0·01-0·07)
	GMFR referencing Day 1		0·6		2·1		2·9		0·5		8·9		16·1		1·4
	95% CI		0·0-16·0		0·3-14·4		0·2-46·2		0·1-4·7		0·9-86·0		0·2-1461·2		0·4-4·8
Human IgG1 Fc Region	GMT	0·001	0·001	0·001	0·003	0·027	0·013	0·055	0·037	0·001	0·001	0·001	0·019	0·020	0·026
	95% CI	0·000-0·014	0·000-0·017	0·000-0·006	0·000-0·017	0·010-0·077	0·001-0·210	0·032-0·097	0·010-0·133	0·000-0·010	0·000-0·011	0·000-0·033	0·007-0·058	0·001-0·284	0·002-0·397
	Median (IQR)	0·01 (0·00-0·02)	0·01 (0·00-0·03)	0·01 (0·00-0·01)	0·01 (0·00-0·03)	0·03 (0·01-0·09)	0·02 (0·01-0·18)	0·07 (0·04-0·07)	0·05 (0·03-0·10)	0·00 (0·00-0·04)	0·01 (0·00-0·04)	0·00 (0·00-0·03)	0·02 (0·01-0·05)	0·06 (0·02-0·11)	0·06 (0·05-0·10)
	GMFR referencing Day 1		1·1		3·2		0·5		0·7		1·1		13·3		1·3
	95% CI		0·1-22·3		0·8-12·9		0·0-4·7		0·2-2·6		0·2-7·0		0·6-296·9		0·0-44·7
TNFa+															
WT RBD	GMT	0·336	0·331	0·227	0·272	0·179	0·220	0·728	0·757	0·303	0·320	0·414	0·443	0·674	0·646
	95% CI	0·197-0·574	0·188-0·585	0·121-0·427	0·158-0·468	0·059-0·544	0·077-0·632	0·379-1·395	0·433-1·324	0·203-0·452	0·226-0·453	0·236-0·727	0·289-0·677	0·331-1·369	0·296-1·413
	Median (IQR)	0·50 (0·16-0·61)	0·43 (0·19-0·59)	0·35 (0·12-0·48)	0·33 (0·22-0·48)	0·18 (0·12-0·52)	0·20 (0·10-0·64)	0·96 (0·44-1·35)	0·97 (0·55-1·06)	0·35 (0·23-0·47)	0·35 (0·22-0·51)	0·44 (0·21-0·62)	0·50 (0·28-0·59)	0·76 (0·30-1·30)	0·64 (0·29-1·62)
	GMFR referencing Day 1		1·0		1·2		1·2		1·0		1·1		1·1		1·0
	95% CI		0·8-1·2		1·0-1·4		0·6-2·6		0·9-1·3		0·9-1·2		0·6-1·9		0·7-1·2
Beta RBD	GMT	0·209	0·212	0·095	0·072	0·126	0·039	0·189	0·068	0·178	0·207	0·195	0·217	0·225	0·224
	95% CI	0·093-0·466	0·099-0·453	0·023-0·396	0·013-0·391	0·043-0·364	0·002-0·709	0·061-0·588	0·003-1·522	0·103-0·307	0·128-0·336	0·088-0·434	0·113-0·417	0·062-0·813	0·080-0·632
	Median (IQR)	0·34 (0·09-0·53)	0·35 (0·11-0·42)	0·23 (0·05-0·31)	0·22 (0·07-0·35)	0·12 (0·06-0·38)	0·11 (0·04-0·25)	0·21 (0·08-0·63)	0·15 (0·07-0·63)	0·21 (0·10-0·36)	0·29 (0·14-0·37)	0·22 (0·11-0·44)	0·24 (0·11-0·44)	0·27 (0·12-0·58)	0·27 (0·11-0·54)
	GMFR referencing Day 1		1·0		0·8		0·3		0·4		1·2		1·1		1·0
	95% CI		0·8-1·2		0·4-1·6		0·0-4·4		0·0-3·3		1·0-1·4		0·8-1·5		0·6-1·6
WT Spike	GMT	0·010	0·013	0·004	0·011	0·005	0·009	0·007	0·005	0·008	0·013	0·018	0·025	0·006	0·003
	95% CI	0·005-0·020	0·008-0·023	0·001-0·015	0·006-0·022	0·000-0·049	0·001-0·095	0·001-0·069	0·000-0·051	0·003-0·023	0·007-0·025	0·010-0·033	0·016-0·041	0·001-0·059	0·000-0·067
	Median (IQR)	0·01 (0·01-0·02)	0·01 (0·01-0·03)	0·01 (0·00-0·02)	0·01 (0·01-0·03)	0·01 (0·00-0·02)	0·03 (0·01-0·04)	0·01 (0·01-0·03)	0·01 (0·00-0·03)	0·01 (0·01-0·02)	0·02 (0·01-0·03)	0·02 (0·01-0·04)	0·03 (0·02-0·04)	0·01 (0·01-0·03)	0·01 (0·00-0·03)
	GMFR referencing Day 1		1·4		3·2		1·9		0·7		1·7		1·4		0·5
	95% CI		0·8-2·3		0·8-13·1		0·1-34·3		0·1-8·0		0·6-4·9		1·1-1·9		0·1-4·7
Human IgG1 Fc Region	GMT	0·210	0·217	0·067	0·068	0·086	0·049	0·199	0·204	0·204	0·190	0·203	0·203	0·216	0·181
	95% CI	0·094-0·473	0·091-0·519	0·010-0·447	0·010-0·452	0·019-0·402	0·003-0·925	0·061-0·648	0·071-0·586	0·114-0·365	0·097-0·370	0·075-0·552	0·082-0·503	0·057-0·824	0·047-0·699
	Median (IQR)	0·41 (0·10-0·47)	0·38 (0·09-0·48)	0·29 (0·08-0·38)	0·27 (0·09-0·35)	0·10 (0·06-0·37)	0·12 (0·08-0·26)	0·23 (0·07-0·73)	0·16 (0·08-0·71)	0·27 (0·13-0·39)	0·29 (0·17-0·37)	0·25 (0·12-0·51)	0·26 (0·11-0·48)	0·25 (0·11-0·65)	0·22 (0·12-0·55)
	GMFR referencing Day 1		1·0		1·0		0·6		1·0		0·9		1·0		0·8
	95% CI		0·8-1·4		0·9-1·2		0·0-12·2		0·8-1·3		0·7-1·2		0·7-1·3		0·5-1·4
MIP1b+															
WT RBD	GMT	0·000	0·000	0·001	0·003	0·000	0·000	0·067	0·034	0·003	0·003	0·013	0·003	0·017	0·013
	95% CI	0·000-0·006	0·000-0·001	0·000-0·011	0·000-0·024	0·000-0·002	0·000-0·004	0·019-0·234	0·002-0·567	0·000-0·030	0·000-0·027	0·001-0·183	0·000-0·186	0·001-0·248	0·001-0·242
	Median (IQR)	0·00 (0·00-0·02)	0·00 (0·00-0·00)	0·01 (0·00-0·03)	0·03 (0·00-0·06)	0·00 (0·00-0·00)	0·00 (0·00-0·01)	0·10 (0·06-0·15)	0·10 (0·04-0·19)	0·04 (0·00-0·07)	0·03 (0·00-0·07)	0·02 (0·01-0·04)	0·03 (0·00-0·15)	0·06 (0·01-0·12)	0·04 (0·00-0·14)
	GMFR referencing Day 1		0·3		2·2		1·4		0·5		0·9		0·2		0·7
	95% CI		0·1-1·3		0·4-13·8		0·1-34·1		0·1-2·7		0·1-16·1		0·0-6·6		0·3-1·9
Beta RBD	GMT	0·000	0·000	0·000	0·000	0·000	0·000	0·000	0·000	0·000	0·000	0·000	0·000	0·000	0·000
	95% CI	0·000-0·000	0·000-0·000	0·000-0·000	0·000-0·000	0·000-0·000	0·000-0·000	0·000-0·000	0·000-0·000	0·000-0·000	0·000-0·000	0·000-0·000	0·000-0·000	0·000-0·000	0·000-0·000
	Median (IQR)	0·00 (0·00-0·00)	0·00 (0·00-0·00)	0·00 (0·00-0·00)	0·00 (0·00-0·00)	0·00 (0·00-0·00)	0·00 (0·00-0·00)	0·00 (0·00-0·00)	0·00 (0·00-0·00)	0·00 (0·00-0·00)	0·00 (0·00-0·00)	0·00 (0·00-0·00)	0·00 (0·00-0·00)	0·00 (0·00-0·00)	0·00 (0·00-0·00)
	GMFR referencing Day 1		1·4		1·0		1·0		1·0		1·0		1·0		1·0
	95% CI		0·7-2·7		0·9-1·0		1·0-1·0		1·0-1·0		1·0-1·0		1·0-1·0		1·0-1·0
WT Spike	GMT	0·001	0·001	0·001	0·001	0·001	0·002	0·000	0·000	0·001	0·001	0·038	0·001	0·001	0·000
	95% CI	0·000-0·005	0·000-0·010	0·000-0·006	0·000-0·007	0·000-0·016	0·000-0·042	0·000-0·008	0·000-0·002	0·000-0·008	0·000-0·012	0·015-0·095	0·000-0·050	0·000-0·012	0·000-0·004
	Median (IQR)	0·00 (0·00-0·01)	0·00 (0·00-0·02)	0·01 (0·00-0·01)	0·00 (0·00-0·02)	0·00 (0·00-0·02)	0·01 (0·00-0·04)	0·00 (0·00-0·01)	0·00 (0·00-0·00)	0·01 (0·00-0·01)	0·00 (0·00-0·06)	0·03 (0·02-0·13)	0·00 (0·00-0·09)	0·00 (0·00-0·01)	0·00 (0·00-0·01)
	GMFR referencing Day 1		1·9		1·0		2·5		0·3		1·2		0·0		0·2
	95% CI		0·1-34·9		0·1-11·9		0·1-109·4		0·0-6·7		0·2-9·9		0·0-1·0		0·0-1·9
Human IgG1 Fc Region	GMT	0·000	0·000	0·000	0·000	0·000	0·000	0·000	0·000	0·000	0·000	0·000	0·000	0·000	0·000
	95% CI	0·000-0·000	0·000-0·000	0·000-0·000	0·000-0·000	0·000-0·000	0·000-0·000	0·000-0·000	0·000-0·000	0·000-0·000	0·000-0·000	0·000-0·000	0·000-0·000	0·000-0·000	0·000-0·000
	Median (IQR)	0·00 (0·00-0·00)	0·00 (0·00-0·00)	0·00 (0·00-0·00)	0·00 (0·00-0·00)	0·00 (0·00-0·00)	0·00 (0·00-0·00)	0·00 (0·00-0·00)	0·00 (0·00-0·00)	0·00 (0·00-0·00)	0·00 (0·00-0·00)	0·00 (0·00-0·00)	0·00 (0·00-0·00)	0·00 (0·00-0·00)	0·00 (0·00-0·00)
	GMFR referencing Day 1		1·4		0·7		1·0		1·0		1·6		1·0		1·0
	95% CI		0·7-3·0		0·3-1·5		1·0-1·0		1·0-1·0		0·8-3·2		1·0-1·0		1·0-1·0
IL5+															
WT RBD	GMT	0·000	0·001	0·000	0·001	0·000	0·001	0·000	0·000	0·001	0·000	0·002	0·001	0·001	0·001
	95% CI	0·000-0·001	0·000-0·006	0·000-0·003	0·000-0·006	0·000-0·001	0·000-0·010	0·000-0·006	0·000-0·000	0·000-0·007	0·000-0·003	0·000-0·022	0·000-0·011	0·000-0·011	0·000-0·013
	Median (IQR)	0·00 (0·00-0·00)	0·00 (0·00-0·01)	0·00 (0·00-0·01)	0·01 (0·00-0·01)	0·00 (0·00-0·00)	0·00 (0·00-0·01)	0·00 (0·00-0·01)	0·00 (0·00-0·00)	0·01 (0·00-0·01)	0·00 (0·00-0·01)	0·01 (0·00-0·01)	0·00 (0·00-0·01)	0·00 (0·00-0·01)	0·00 (0·00-0·01)
	GMFR referencing Day 1		5·7		1·8		11·1		0·1		0·3		0·4		0·7
	95% CI		0·3-95·5		0·1-31·6		0·5-243·4		0·0-3·8		0·0-4·4		0·0-40·6		0·0-18·3
Beta RBD	GMT	0·000	0·000	0·004	0·004	0·000	0·000	0·000	0·000	0·002	0·002	0·002	0·001	0·003	0·000
	95% CI	0·000-0·001	0·000-0·003	0·001-0·020	0·001-0·020	0·000-0·007	0·000-0·004	0·000-0·002	0·000-0·004	0·000-0·012	0·000-0·012	0·000-0·017	0·000-0·018	0·002-0·004	0·000-0·006
	Median (IQR)	0·00 (0·00-0·01)	0·00 (0·00-0·01)	0·01 (0·00-0·02)	0·01 (0·00-0·01)	0·00 (0·00-0·01)	0·00 (0·00-0·01)	0·00 (0·00-0·00)	0·00 (0·00-0·01)	0·01 (0·00-0·02)	0·01 (0·00-0·02)	0·01 (0·00-0·01)	0·01 (0·00-0·01)	0·00 (0·00-0·00)	0·00 (0·00-0·01)

	GMFR referencing Day 1		2·6		1·0		0·6		1·4		0·8		0·4		0·1
	95% CI		0·1-46·4		0·1-10·6		0·0-183·9		0·0-69·6		0·1-12·2		0·0-7·8		0·0-2·2
WT Spike	GMT	0·000	0·001	0·000	0·001	0·000	0·001	0·000	0·000	0·001	0·000	0·000	0·000	0·001	0·000
	95% CI	0·000-0·000	0·000-0·004	0·000-0·001	0·000-0·003	0·000-0·002	0·000-0·009	0·000-0·002	0·000-0·001	0·000-0·003	0·000-0·001	0·000-0·005	0·000-0·002	0·000-0·009	0·000-0·001
	Median (IQR)	0·00 (0·00-0·00)	0·00 (0·00-0·01)	0·00 (0·00-0·01)	0·00 (0·00-0·01)	0·00 (0·00-0·01)	0·00 (0·00-0·01)	0·00 (0·00-0·00)	0·00 (0·00-0·00)	0·00 (0·00-0·01)	0·00 (0·00-0·00)	0·00 (0·00-0·01)	0·00 (0·00-0·00)	0·00 (0·00-0·01)	0·00 (0·00-0·00)
	GMFR referencing Day 1		15·8		4·4		4·5		0·5		0·2		0·5		0·1
	95% CI		1·7-144·7		0·3-69·3		0·1-264·6		0·0-13·3		0·0-2·6		0·0-67·2		0·0-1·1
Human IgG1 Fc Region	GMT	0·001	0·001	0·001	0·002	0·001	0·002	0·001	0·001	0·001	0·001	0·003	0·003	0·002	0·000
	95% CI	0·000-0·009	0·000-0·006	0·000-0·004	0·000-0·009	0·000-0·009	0·000-0·030	0·000-0·009	0·000-0·013	0·000-0·006	0·000-0·009	0·000-0·026	0·000-0·024	0·000-0·014	0·000-0·006
	Median (IQR)	0·01 (0·00-0·01)	0·00 (0·00-0·01)	0·00 (0·00-0·01)	0·01 (0·00-0·01)	0·00 (0·00-0·01)	0·01 (0·00-0·02)	0·00 (0·00-0·01)	0·00 (0·00-0·01)	0·00 (0·00-0·01)	0·01 (0·00-0·01)	0·01 (0·00-0·02)	0·01 (0·00-0·01)	0·00 (0·00-0·01)	0·00 (0·00-0·01)
	GMFR referencing Day 1		0·6		2·5		2·6		0·5		1·3		0·9		0·1
	95% CI		0·0-11·8		0·3-23·8		0·1-78·7		0·0-37·5		0·3-5·4		0·0-20·0		0·0-2·6
IL13+															
WT RBD	GMT	0·002	0·001	0·000	0·000	0·000	0·001	0·000	0·000	0·000	0·002	0·002	0·001	0·002	0·001
	95% CI	0·000-0·008	0·000-0·007	0·000-0·002	0·000-0·002	0·000-0·006	0·000-0·017	0·000-0·004	0·000-0·005	0·000-0·001	0·000-0·007	0·000-0·033	0·000-0·017	0·000-0·011	0·000-0·007
	Median (IQR)	0·00 (0·00-0·01)	0·00 (0·00-0·01)	0·00 (0·00-0·00)	0·00 (0·00-0·01)	0·00 (0·00-0·01)	0·00 (0·00-0·01)	0·00 (0·00-0·00)	0·00 (0·00-0·00)	0·00 (0·00-0·00)	0·01 (0·00-0·01)	0·01 (0·00-0·01)	0·01 (0·00-0·01)	0·00 (0·00-0·00)	0·00 (0·00-0·00)
	GMFR referencing Day 1		0·7		1·1		3·5		0·7		7·9		0·6		0·4
	95% CI		0·1-5·4		0·2-7·2		0·1-97·3		0·0-17·0		0·7-90·0		0·0-45·7		0·0-12·0
Beta RBD	GMT	0·001	0·002	0·001	0·003	0·003	0·005	0·001	0·001	0·001	0·008	0·016	0·004	0·001	0·001
	95% CI	0·000-0·006	0·000-0·008	0·000-0·005	0·001-0·012	0·000-0·051	0·001-0·019	0·000-0·014	0·000-0·013	0·000-0·004	0·005-0·013	0·007-0·034	0·000-0·049	0·000-0·010	0·000-0·016
	Median (IQR)	0·00 (0·00-0·01)	0·00 (0·00-0·01)	0·00 (0·00-0·01)	0·01 (0·00-0·02)	0·01 (0·00-0·02)	0·01 (0·00-0·01)	0·00 (0·00-0·01)	0·00 (0·00-0·01)	0·00 (0·00-0·01)	0·01 (0·01-0·01)	0·01 (0·01-0·04)	0·01 (0·00-0·03)	0·00 (0·00-0·01)	0·01 (0·00-0·01)
	GMFR referencing Day 1		1·7		3·2		1·8		0·9		8·5		0·3		1·2
	95% CI		0·2-14·7		0·5-21·1		0·2-16·2		0·0-46·0		1·8-40·9		0·0-2·4		0·0-68·5
WT Spike	GMT	0·000	0·000	0·000	0·000	0·000	0·000	0·000	0·000	0·000	0·000	0·000	0·000	0·000	0·000
	95% CI	0·000-0·001	0·000-0·001	0·000-0·000	0·000-0·000	0·000-0·002	0·000-0·002	0·000-0·001	0·000-0·001	0·000-0·001	0·000-0·001	0·000-0·001	0·000-0·001	0·000-0·003	0·000-0·000
	Median (IQR)	0·00 (0·00-0·00)	0·00 (0·00-0·00)	0·00 (0·00-0·00)	0·00 (0·00-0·00)	0·00 (0·00-0·00)	0·00 (0·00-0·00)	0·00 (0·00-0·00)	0·00 (0·00-0·00)	0·00 (0·00-0·00)	0·00 (0·00-0·00)	0·00 (0·00-0·00)	0·00 (0·00-0·00)	0·00 (0·00-0·00)	0·00 (0·00-0·00)
	GMFR referencing Day 1		1·0		1·7		0·9		1·3		1·3		0·9		0·2
	95% CI		0·1-14·9		0·2-18·1		0·0-26·2		0·0-65·1		0·1-22·3		0·1-7·9		0·0-2·8
Human IgG1 Fc Region	GMT	0·001	0·001	0·001	0·002	0·001	0·008	0·000	0·001	0·000	0·003	0·002	0·003	0·000	0·001
	95% CI	0·000-0·008	0·000-0·004	0·000-0·007	0·000-0·008	0·000-0·019	0·003-0·025	0·000-0·008	0·000-0·012	0·000-0·002	0·001-0·012	0·000-0·027	0·000-0·077	0·000-0·006	0·000-0·012
	Median (IQR)	0·01 (0·00-0·01)	0·00 (0·00-0·01)	0·00 (0·00-0·01)	0·01 (0·00-0·01)	0·01 (0·00-0·01)	0·01 (0·00-0·02)	0·00 (0·00-0·01)	0·00 (0·00-0·01)	0·00 (0·00-0·01)	0·01 (0·00-0·02)	0·01 (0·00-0·02)	0·01 (0·00-0·04)	0·00 (0·00-0·01)	0·00 (0·00-0·00)
	GMFR referencing Day 1		0·5		1·1		10·0		2·1		9·0		1·9		2·4
	95% CI		0·1-4·6		0·3-4·2		0·5-193·1		0·1-49·8		0·8-105·2		0·1-31·3		0·3-21·0
IFNg+ TNFa+															
WT RBD	GMT	0·001	0·002	0·000	0·001	0·000	0·002	0·006	0·007	0·002	0·003	0·003	0·012	0·010	0·019
	95% CI	0·000-0·008	0·000-0·013	0·000-0·002	0·000-0·003	0·000-0·001	0·000-0·018	0·000-0·068	0·001-0·064	0·001-0·007	0·001-0·012	0·000-0·048	0·004-0·031	0·005-0·023	0·007-0·050
	Median (IQR)	0·00 (0·00-0·01)	0·01 (0·00-0·01)	0·00 (0·00-0·00)	0·00 (0·00-0·01)	0·00 (0·00-0·00)	0·01 (0·00-0·01)	0·02 (0·00-0·04)	0·02 (0·01-0·02)	0·01 (0·00-0·01)	0·01 (0·01-0·01)	0·01 (0·00-0·03)	0·01 (0·00-0·02)	0·01 (0·00-0·02)	0·02 (0·01-0·06)
	GMFR referencing Day 1		2·5		1·7		26·2		1·2		1·2		4·6		1·9
	95% CI		0·4-14·8		0·6-4·7		0·5-1471·6		0·4-3·2		0·5-2·7		0·4-47·6		0·9-4·0
Beta RBD	GMT	0·001	0·001	0·000	0·000	0·000	0·001	0·002	0·005	0·001	0·003	0·001	0·003	0·003	0·006
	95% CI	0·000-0·005	0·000-0·010	0·000-0·001	0·000-0·001	0·000-0·001	0·000-0·006	0·000-0·040	0·001-0·044	0·000-0·003	0·001-0·009	0·000-0·016	0·000-0·020	0·000-0·028	0·001-0·065
	Median (IQR)	0·00 (0·00-0·01)	0·00 (0·00-0·01)	0·00 (0·00-0·00)	0·00 (0·00-0·00)	0·00 (0·00-0·00)	0·00 (0·00-0·00)	0·01 (0·00-0·03)	0·01 (0·00-0·03)	0·00 (0·00-0·01)	0·01 (0·00-0·01)	0·00 (0·00-0·01)	0·01 (0·00-0·01)	0·00 (0·00-0·02)	0·01 (0·01-0·03)
	GMFR referencing Day 1		2·0		1·9		9·0		2·2		3·9		2·1		2·1
	95% CI		0·2-19·7		0·4-9·1		0·3-289·5		0·3-14·8		1·1-14·3		0·1-45·6		0·9-5·3
WT Spike	GMT	0·008	0·008	0·007	0·010	0·011	0·010	0·009	0·013	0·011	0·014	0·015	0·016	0·008	0·011
	95% CI	0·004-0·014	0·004-0·016	0·003-0·020	0·007-0·016	0·005-0·026	0·005-0·021	0·002-0·046	0·005-0·034	0·007-0·019	0·009-0·023	0·007-0·029	0·010-0·025	0·004-0·018	0·003-0·033
	Median (IQR)	0·01 (0·00-0·02)	0·01 (0·00-0·02)	0·01 (0·01-0·02)	0·01 (0·01-0·02)	0·02 (0·00-0·02)	0·01 (0·01-0·02)	0·01 (0·01-0·03)	0·01 (0·01-0·04)	0·01 (0·01-0·02)	0·01 (0·01-0·02)	0·01 (0·01-0·03)	0·01 (0·01-0·03)	0·01 (0·01-0·01)	0·01 (0·01-0·02)
	GMFR referencing Day 1		1·1		1·4		0·9		1·5		1·2		1·1		1·3
	95% CI		0·7-1·6		0·6-3·2		0·4-2·0		0·5-4·5		1·0-1·6		0·8-1·6		0·5-3·7
Human IgG1 Fc Region	GMT	0·000	0·000	0·000	0·000	0·000	0·000	0·001	0·001	0·000	0·000	0·000	0·000	0·001	0·002
	95% CI	0·000-0·003	0·000-0·003	0·000-0·001	0·000-0·001	0·000-0·000	0·000-0·001	0·000-0·022	0·000-0·009	0·000-0·002	0·000-0·002	0·000-0·006	0·000-0·005	0·000-0·020	0·000-0·025
	Median (IQR)	0·00 (0·00-0·01)	0·00 (0·00-0·01)	0·00 (0·00-0·00)	0·00 (0·00-0·00)	0·00 (0·00-0·00)	0·00 (0·00-0·00)	0·01 (0·00-0·02)	0·00 (0·00-0·01)	0·00 (0·00-0·00)	0·00 (0·00-0·01)	0·00 (0·00-0·01)	0·00 (0·00-0·01)	0·00 (0·00-0·02)	0·00 (0·00-0·02)
	GMFR referencing Day 1		1·2		1·2		3·0		0·7		1·2		1·3		1·2
	95% CI		0·1-20·7		0·4-3·7		0·2-54·8		0·1-6·0		0·3-5·0		0·1-29·6		0·1-13·4
IL5+ IL13+															
WT RBD	GMT	0·000	0·000	0·000	0·000	0·000	0·000	0·000	0·000	0·000	0·000	0·000	0·000	0·000	0·000
	95% CI	0·000-0·000	0·000-0·000	0·000-0·000	0·000-0·000	0·000-0·000	0·000-0·000	0·000-0·000	0·000-0·000	0·000-0·000	0·000-0·000	0·000-0·000	0·000-0·000	0·000-0·000	0·000-0·000
	Median (IQR)	0·00 (0·00-0·00)	0·00 (0·00-0·00)	0·00 (0·00-0·00)	0·00 (0·00-0·00)	0·00 (0·00-0·00)	0·00 (0·00-0·00)	0·00 (0·00-0·00)	0·00 (0·00-0·00)	0·00 (0·00-0·00)	0·00 (0·00-0·00)	0·00 (0·00-0·00)	0·00 (0·00-0·00)	0·00 (0·00-0·00)	0·00 (0·00-0·00)
	GMFR referencing Day 1		0·7		0·6		1·0		1·0		1·0		0·6		1·0
	95% CI		0·3-1·6		0·2-1·8		1·0-1·0		1·0-1·0		1·0-1·0		0·1-6·2		1·0-1·0
Beta RBD	GMT	0·000	0·000	0·000	0·000	0·000	0·000	0·000	0·000	0·000	0·000	0·000	0·000	0·000	0·000
	95% CI	0·000-0·000	0·000-0·000	0·000-0·000	0·000-0·000	0·000-0·000	0·000-0·000	0·000-0·000	0·000-0·000	0·000-0·000	0·000-0·000	0·000-0·000	0·000-0·000	0·000-0·000	0·000-0·000
	Median (IQR)	0·00 (0·00-0·00)	0·00 (0·00-0·00)	0·00 (0·00-0·00)	0·00 (0·00-0·00)	0·00 (0·00-0·00)	0·00 (0·00-0·00)	0·00 (0·00-0·00)	0·00 (0·00-0·00)	0·00 (0·00-0·00)	0·00 (0·00-0·00)	0·00 (0·00-0·00)	0·00 (0·00-0·00)	0·00 (0·00-0·00)	0·00 (0·00-0·00)
	GMFR referencing Day 1		1·9		0·8		1·0		1·8		1·3		0·3		1·9
	95% CI		0·7-4·9		0·4-1·7		0·2-6·1		0·5-7·1		0·7-2·4		0·1-1·4		0·4-8·5
WT Spike	GMT	0·000	0·000	0·000	0·000	0·000	0·000	0·000	0·000	0·000	0·000	0·000	0·000	0·000	0·000
	95% CI	0·000-0·000	0·000-0·000	0·000-0·000	0·000-0·000	0·000-0·000	0·000-0·000	0·000-0·000	0·000-0·000	0·000-0·000	0·000-0·000	0·000-0·000	0·000-0·000	0·000-0·000	0·000-0·000
	Median (IQR)	0·00 (0·00-0·00)	0·00 (0·00-0·00)	0·00 (0·00-0·00)	0·00 (0·00-0·00)	0·00 (0·00-0·00)	0·00 (0·00-0·00)	0·00 (0·00-0·00)	0·00 (0·00-0·00)	0·00 (0·00-0·00)	0·00 (0·00-0·00)	0·00 (0·00-0·00)	0·00 (0·00-0·00)	0·00 (0·00-0·00)	0·00 (0·00-0·00)
	GMFR referencing Day 1		2·5		1·2		0·9		0·4		1·0		1·7		1·6
	95% CI		0·9-7·2		0·5-3·2		0·1-9·8		0·1-1·7		1·0-1·0		0·5-5·4		0·5-4·6
Human IgG1 Fc Region	GMT	0·000	0·000	0·000	0·000	0·000	0·000	0·000	0·000	0·000	0·000	0·000	0·000	0·000	0·000
	95% CI	0·000-0·000	0·000-0·000	0·000-0·000	0·000-0·000	0·000-0·000	0·000-0·000	0·000-0·000	0·000-0·000	0·000-0·000	0·000-0·000	0·000-0·000	0·000-0·000	0·000-0·000	0·000-0·000
	Median (IQR)	0·00 (0·00-0·00)	0·00 (0·00-0·00)	0·00 (0·00-0·00)	0·00 (0·00-0·00)	0·00 (0·00-0·00)	0·00 (0·00-0·00)	0·00 (0·00-0·00)	0·00 (0·00-0·00)	0·00 (0·00-0·00)	0·00 (0·00-0·00)	0·00 (0·00-0·00)	0·00 (0·00-0·00)	0·00 (0·00-0·00)	0·00 (0·00-0·00)
	GMFR referencing Day 1		1·4		1·0		0·6		1·0		2·3		0·9		1·7
	95% CI		0·7-2·9		1·0-1·0		0·2-2·0		1·0-1·0		0·9-6·2		0·1-7·2		0·5-6·1
IFNg+ MIP1b+															
WT RBD	GMT	0·000	0·000	0·000	0·000	0·000	0·000	0·000	0·000	0·000	0·000	0·000	0·000	0·000	0·000
	95% CI	0·000-0·000	0·000-0·000	0·000-0·000	0·000-0·000	0·000-0·000	0·000-0·001	0·000-0·001	0·000-0·004	0·000-0·001	0·000-0·000	0·000-0·002	0·000-0·006	0·000-0·000	0·000-0·002
	Median (IQR)	0·00 (0·00-0·00)	0·00 (0·00-0·00)	0·00 (0·00-0·00)	0·00 (0·00-0·00)	0·00 (0·00-0·00)	0·00 (0·00-0·00)	0·00 (0·00-0·01)	0·00 (0·00-0·01)	0·00 (0·00-0·01)	0·00 (0·00-0·00)	0·00 (0·00-0·01)	0·00 (0·00-0·01)	0·00 (0·00-0·00)	0·00 (0·00-0·00)
	GMFR referencing Day 1		0·8		0·5		1·2		3·5		0·2		2·3		10·2
	95% CI		0·1-3·9		0·1-3·4		0·8-1·6		0·2-52·5		0·0-2·6		0·1-41·0		1·0-104·1
Beta RBD	GMT	0·000	0·000	0·000	0·000	0·000	0·000	0·000	0·000	0·000	0·000	0·000	0·000	0·000	0·000
	95% CI	0·000-0·000	0·000-0·000	0·000-0·000	0·000-0·000	0·000-0·000	0·000-0·000	0·000-0·002	0·000-0·001	0·000-0·000	0·000-0·000	0·000-0·000	0·000-0·000	0·000-0·000	0·000-0·000
	Median (IQR)	0·00 (0·00-0·00)	0·00 (0·00-0·00)	0·00 (0·00-0·00)	0·00 (0·00-0·00)	0·00 (0·00-0·00)	0·00 (0·00-0·00)	0·00 (0·00-0·00)	0·00 (0·00-0·00)	0·00 (0·00-0·00)	0·00 (0·00-0·00)	0·00 (0·00-0·00)	0·00 (0·00-0·00)	0·00 (0·00-0·00)	0·00 (0·00-0·00)
	GMFR referencing Day 1		2·6		0·3		1·0		0·9		1·2		0·5		1·7
	95% CI		0·5-14·6		0·1-1·6		1·0-1·0		0·1-13·8		0·1-9·2		0·1-2·9		0·3-10·7
WT Spike	GMT	0·000	0·000	0·000	0·000	0·000	0·002	0·001	0·001	0·001	0·001	0·006	0·002	0·001	0·001
	95% CI	0·000-0·001	0·000-0·003	0·000-0·002	0·000-0·002	0·000-0·008	0·000-0·043	0·000-0·008	0·000-0·008	0·000-0·005	0·000-0·004	0·002-0·022	0·000-0·038	0·000-0·009	0·000-0·016
	Median (IQR)	0·00 (0·00-0·00)	0·00 (0·00-0·01)	0·00 (0·00-0·00)	0·00 (0·00-0·00)	0·00 (0·00-0·01)	0·01 (0·00-0·03)	0·00 (0·00-0·01)	0·00 (0·00-0·01)	0·00 (0·00-0·01)	0·00 (0·00-0·01)	0·01 (0·00-0·02)	0·01 (0·00-0·02)	0·00 (0·00-0·01)	0·01 (0·00-0·02)
	GMFR referencing Day 1		3·4		0·9		4·8		0·7		0·7		0·4		1·1
	95% CI		0·1-82·9		0·1-5·9		0·2-139·2		0·0-16·1		0·1-7·6		0·0-3·4		0·0-50·3
Human IgG1 Fc Region	GMT	0·000	0·000	0·000	0·000	0·000	0·000	0·000	0·000	0·000	0·000	0·000	0·000	0·000	0·000
	95% CI	0·000-0·000	0·000-0·000	0·000-0·000	0·000-0·000	0·000-0·000	0·000-0·000	0·000-0·001	0·000-0·000	0·000-0·000	0·000-0·000	0·000-0·000	0·000-0·000	0·000-0·000	0·000-0·000
	Median (IQR)	0·00 (0·00-0·00)	0·00 (0·00-0·00)	0·00 (0·00-0·00)	0·00 (0·00-0·00)	0·00 (0·00-0·00)	0·00 (0·00-0·00)	0·00 (0·00-0·00)	0·00 (0·00-0·00)	0·00 (0·00-0·00)	0·00 (0·00-0·00)	0·00 (0·00-0·00)	0·00 (0·00-0·00)	0·00 (0·00-0·00)	0·00 (0·00-0·00)
	GMFR referencing Day 1		1·5		0·9		1·0		0·5		0·4		1·0		1·1
	95% CI		0·6-3·9		0·1-6·2		1·0-1·0		0·1-3·0		0·1-2·5		1·0-1·0		0·8-1·6
TNFa+ MIP1b+															
WT RBD	GMT	0·023	0·021	0·041	0·048	0·040	0·082	0·221	0·199	0·109	0·110	0·121	0·182	0·112	0·107
	95% CI	0·007-0·073	0·010-0·048	0·025-0·069	0·023-0·104	0·013-0·125	0·028-0·245	0·083-0·587	0·083-0·480	0·066-0·180	0·055-0·220	0·054-0·268	0·071-0·465	0·035-0·364	0·031-0·365
	Median (IQR)	0·02 (0·01-0·05)	0·02 (0·01-0·03)	0·05 (0·02-0·08)	0·07 (0·02-0·13)	0·05 (0·01-0·13)	0·07 (0·04-0·23)	0·28 (0·15-0·52)	0·21 (0·12-0·37)	0·11 (0·08-0·19)	0·12 (0·07-0·22)	0·09 (0·06-0·32)	0·14 (0·08-0·42)	0·17 (0·04-0·35)	0·13 (0·04-0·36)
	GMFR referencing Day 1		0·9		1·2		2·1		0·9		1·0		1·5		1·0
	95% CI		0·4-1·9		0·8-1·8		1·0-4·4		0·5-1·7		0·7-1·6		0·6-4·1		0·6-1·5
Beta RBD	GMT	0·000	0·000	0·000	0·000	0·000	0·000	0·000	0·000	0·000	0·000	0·000	0·000	0·000	0·000
	95% CI	0·000-0·000	0·000-0·000	0·000-0·000	0·000-0·000	0·000-0·000	0·000-0·000	0·000-0·000	0·000-0·000	0·000-0·000	0·000-0·000	0·000-0·000	0·000-0·000	0·000-0·000	0·000-0·000
	Median (IQR)	0·00 (0·00-0·00)	0·00 (0·00-0·00)	0·00 (0·00-0·00)	0·00 (0·00-0·00)	0·00 (0·00-0·00)	0·00 (0·00-0·00)	0·00 (0·00-0·00)	0·00 (0·00-0·00)	0·00 (0·00-0·00)	0·00 (0·00-0·00)	0·00 (0·00-0·00)	0·00 (0·00-0·00)	0·00 (0·00-0·00)	0·00 (0·00-0·00)
	GMFR referencing Day 1		2·5		0·6		1·0		1·6		1·5		1·3		1·2
	95% CI		0·7-8·2		0·2-2·2		1·0-1·0		0·4-5·7		0·3-7·2		0·1-22·0		0·0-32·6
WT Spike	GMT	0·000	0·000	0·000	0·000	0·001	0·001	0·000	0·000	0·000	0·001	0·002	0·002	0·000	0·000
	95% CI	0·000-0·001	0·000-0·001	0·000-0·001	0·000-0·001	0·000-0·005	0·000-0·007	0·000-0·001	0·000-0·002	0·000-0·001	0·001-0·004	0·000-0·023	0·000-0·015	0·000-0·002	0·000-0·005
	Median (IQR)	0·00 (0·00-0·00)	0·00 (0·00-0·00)	0·00 (0·00-0·00)	0·00 (0·00-0·00)	0·00 (0·00-0·00)	0·00 (0·00-0·00)	0·00 (0·00-0·00)	0·00 (0·00-0·00)	0·00 (0·00-0·00)	0·00 (0·00-0·00)	0·01 (0·00-0·01)	0·01 (0·00-0·01)	0·00 (0·00-0·00)	0·00 (0·00-0·01)
	GMFR referencing Day 1		1·2		0·6		1·3		3·2		5·6		0·8		1·7
	95% CI		0·1-15·8		0·2-2·3		0·1-10·7		0·2-47·8		1·1-28·4		0·1-11·1		0·1-24·7
Human IgG1 Fc Region	GMT	0·000	0·000	0·000	0·000	0·000	0·000	0·001	0·000	0·000	0·000	0·000	0·001	0·000	0·000
	95% CI	0·000-0·001	0·000-0·000	0·000-0·000	0·000-0·001	0·000-0·001	0·000-0·001	0·000-0·008	0·000-0·002	0·000-0·000	0·000-0·001	0·000-0·005	0·000-0·008	0·000-0·001	0·000-0·004
	Median (IQR)	0·00 (0·00-0·00)	0·00 (0·00-0·00)	0·00 (0·00-0·00)	0·00 (0·00-0·00)	0·00 (0·00-0·00)	0·00 (0·00-0·00)	0·00 (0·00-0·01)	0·00 (0·00-0·00)	0·00 (0·00-0·00)	0·00 (0·00-0·00)	0·00 (0·00-0·00)	0·00 (0·00-0·01)	0·00 (0·00-0·00)	0·00 (0·00-0·00)
	GMFR referencing Day 1		0·4		1·9		0·9		0·2		2·6		1·5		2·8
	95% CI		0·0-3·0		0·5-7·5		0·1-7·4		0·0-3·2		0·7-9·0		0·3-8·5		0·1-62·4
IFNg+ TNFa+ MIP1b+															
WT RBD	GMT	0·000	0·001	0·000	0·000	0·001	0·003	0·007	0·006	0·000	0·001	0·002	0·002	0·000	0·001
	95% CI	0·000-0·002	0·000-0·004	0·000-0·001	0·000-0·002	0·000-0·012	0·000-0·053	0·002-0·029	0·002-0·017	0·000-0·002	0·000-0·004	0·000-0·033	0·000-0·052	0·000-0·004	0·000-0·016
	Median (IQR)	0·00 (0·00-0·00)	0·00 (0·00-0·00)	0·00 (0·00-0·00)	0·00 (0·00-0·00)	0·00 (0·00-0·01)	0·01 (0·00-0·05)	0·01 (0·00-0·03)	0·01 (0·00-0·02)	0·00 (0·00-0·00)	0·00 (0·00-0·00)	0·00 (0·00-0·03)	0·01 (0·00-0·05)	0·00 (0·00-0·01)	0·00 (0·00-0·01)
	GMFR referencing Day 1		2·0		1·8		3·9		0·9		2·8		1·3		3·3
	95% CI		0·4-10·6		0·4-8·0		0·2-66·5		0·5-1·7		0·5-15·4		0·4-4·1		0·9-11·7
Beta RBD	GMT	0·000	0·000	0·000	0·000	0·000	0·000	0·000	0·000	0·000	0·000	0·000	0·000	0·000	0·000
	95% CI	0·000-0·000	0·000-0·000	0·000-0·000	0·000-0·000	0·000-0·000	0·000-0·000	0·000-0·000	0·000-0·000	0·000-0·000	0·000-0·000	0·000-0·000	0·000-0·000	0·000-0·000	0·000-0·000
	Median (IQR)	0·00 (0·00-0·00)	0·00 (0·00-0·00)	0·00 (0·00-0·00)	0·00 (0·00-0·00)	0·00 (0·00-0·00)	0·00 (0·00-0·00)	0·00 (0·00-0·00)	0·00 (0·00-0·00)	0·00 (0·00-0·00)	0·00 (0·00-0·00)	0·00 (0·00-0·00)	0·00 (0·00-0·00)	0·00 (0·00-0·00)	0·00 (0·00-0·00)
	GMFR referencing Day 1		1·3		0·9		1·0		1·9		2·7		3·0		1·0
	95% CI		0·2-7·4		0·4-1·8		1·0-1·1		0·4-8·5		0·7-10·2		0·4-21·7		1·0-1·0
WT Spike	GMT	0·000	0·000	0·000	0·000	0·001	0·001	0·001	0·000	0·000	0·001	0·001	0·000	0·000	0·003
	95% CI	0·000-0·001	0·000-0·001	0·000-0·001	0·000-0·001	0·000-0·006	0·000-0·004	0·001-0·002	0·000-0·001	0·000-0·001	0·000-0·002	0·000-0·004	0·000-0·002	0·000-0·001	0·002-0·005
	Median (IQR)	0·00 (0·00-0·00)	0·00 (0·00-0·00)	0·00 (0·00-0·00)	0·00 (0·00-0·00)	0·00 (0·00-0·00)	0·00 (0·00-0·00)	0·00 (0·00-0·00)	0·00 (0·00-0·00)	0·00 (0·00-0·00)	0·00 (0·00-0·00)	0·00 (0·00-0·00)	0·00 (0·00-0·00)	0·00 (0·00-0·00)	0·00 (0·00-0·00)
	GMFR referencing Day 1		0·4		1·1		0·9		0·2		6·2		0·2		35·8
	95% CI		0·1-1·4		0·2-4·5		0·1-14·9		0·0-0·8		1·2-32·8		0·0-2·4		4·7-271·9
Human IgG1 Fc Region	GMT	0·000	0·000	0·000	0·000	0·000	0·000	0·000	0·000	0·000	0·000	0·000	0·000	0·000	0·000
	95% CI	0·000-0·000	0·000-0·000	0·000-0·000	0·000-0·000	0·000-0·000	0·000-0·000	0·000-0·000	0·000-0·000	0·000-0·000	0·000-0·000	0·000-0·002	0·000-0·001	0·000-0·000	0·000-0·000
	Median (IQR)	0·00 (0·00-0·00)	0·00 (0·00-0·00)	0·00 (0·00-0·00)	0·00 (0·00-0·00)	0·00 (0·00-0·00)	0·00 (0·00-0·00)	0·00 (0·00-0·00)	0·00 (0·00-0·00)	0·00 (0·00-0·00)	0·00 (0·00-0·00)	0·00 (0·00-0·00)	0·00 (0·00-0·00)	0·00 (0·00-0·00)	0·00 (0·00-0·00)
	GMFR referencing Day 1		2·2		1·9		1·2		0·8		1·8		0·5		1·5
	95% CI		0·5-10·4		0·7-4·9		0·1-10·9		0·1-5·6		0·6-5·3		0·0-7·5		0·6-4·1
IFNg+ MIP1b+ IL13+															
WT RBD	GMT	0·003	0·002	0·000	0·000	0·000	0·000	0·023	0·018	0·002	0·001	0·001	0·001	0·014	0·004
	95% CI	0·001-0·018	0·000-0·012	0·000-0·002	0·000-0·002	0·000-0·001	0·000-0·006	0·011-0·052	0·008-0·043	0·000-0·010	0·000-0·004	0·000-0·005	0·000-0·010	0·004-0·054	0·000-0·074
	Median (IQR)	0·01 (0·00-0·02)	0·01 (0·00-0·02)	0·00 (0·00-0·01)	0·00 (0·00-0·00)	0·00 (0·00-0·00)	0·00 (0·00-0·01)	0·04 (0·01-0·04)	0·02 (0·02-0·03)	0·01 (0·00-0·01)	0·00 (0·00-0·01)	0·00 (0·00-0·00)	0·00 (0·00-0·00)	0·02 (0·01-0·03)	0·03 (0·01-0·03)
	GMFR referencing Day 1		0·5		1·2		3·6		0·8		0·3		1·8		0·3
	95% CI		0·0-8·1		0·1-11·1		0·1-148·5		0·5-1·3		0·0-2·2		0·1-41·7		0·0-2·7
Beta RBD	GMT	0·003	0·002	0·000	0·001	0·000	0·002	0·013	0·027	0·001	0·003	0·001	0·003	0·011	0·002
	95% CI	0·000-0·017	0·000-0·015	0·000-0·001	0·000-0·003	0·000-0·004	0·000-0·014	0·001-0·154	0·017-0·043	0·000-0·005	0·001-0·011	0·000-0·016	0·000-0·026	0·004-0·034	0·000-0·028
	Median (IQR)	0·01 (0·00-0·02)	0·01 (0·00-0·02)	0·00 (0·00-0·00)	0·00 (0·00-0·01)	0·00 (0·00-0·01)	0·00 (0·00-0·01)	0·03 (0·02-0·06)	0·03 (0·02-0·04)	0·00 (0·00-0·01)	0·01 (0·00-0·01)	0·01 (0·00-0·01)	0·01 (0·00-0·02)	0·02 (0·00-0·03)	0·01 (0·00-0·02)
	GMFR referencing Day 1		0·7		2·0		9·9		2·1		3·6		4·3		0·2
	95% CI		0·1-7·1		0·2-16·7		0·2-543·5		0·2-22·2		0·6-19·8		0·0-368·0		0·0-2·0
WT Spike	GMT	0·000	0·000	0·000	0·000	0·000	0·000	0·001	0·000	0·000	0·000	0·000	0·000	0·000	0·000
	95% CI	0·000-0·000	0·000-0·001	0·000-0·000	0·000-0·001	0·000-0·000	0·000-0·002	0·000-0·009	0·000-0·003	0·000-0·000	0·000-0·000	0·000-0·003	0·000-0·001	0·000-0·002	0·000-0·001
	Median (IQR)	0·00 (0·00-0·00)	0·00 (0·00-0·00)	0·00 (0·00-0·00)	0·00 (0·00-0·00)	0·00 (0·00-0·00)	0·00 (0·00-0·00)	0·00 (0·00-0·01)	0·00 (0·00-0·00)	0·00 (0·00-0·00)	0·00 (0·00-0·00)	0·00 (0·00-0·00)	0·00 (0·00-0·00)	0·00 (0·00-0·01)	0·00 (0·00-0·00)
	GMFR referencing Day 1		3·7		2·7		5·2		0·3		0·4		0·4		0·8
	95% CI		0·1-108·5		0·5-14·8		0·2-146·0		0·0-11·1		0·0-3·2		0·0-41·7		0·0-30·6
Human IgG1 Fc Region	GMT	0·005	0·001	0·000	0·000	0·001	0·001	0·028	0·024	0·001	0·001	0·001	0·002	0·012	0·002
	95% CI	0·001-0·018	0·000-0·006	0·000-0·002	0·000-0·002	0·000-0·008	0·000-0·007	0·017-0·047	0·013-0·046	0·000-0·005	0·000-0·007	0·000-0·018	0·000-0·034	0·003-0·044	0·000-0·047
	Median (IQR)	0·01 (0·00-0·01)	0·00 (0·00-0·01)	0·00 (0·00-0·01)	0·00 (0·00-0·01)	0·00 (0·00-0·01)	0·00 (0·00-0·00)	0·03 (0·02-0·04)	0·03 (0·01-0·04)	0·00 (0·00-0·01)	0·01 (0·00-0·01)	0·01 (0·00-0·01)	0·01 (0·00-0·01)	0·02 (0·01-0·03)	0·01 (0·00-0·03)
	GMFR referencing Day 1		0·1		0·8		2·1		0·9		1·9		1·6		0·2
	95% CI		0·0-1·8		0·1-3·9		0·1-42·6		0·6-1·3		0·2-21·7		0·0-144·3		0·0-2·3

CI denotes confidence interval, GMT geometric mean titre, IQR interquartile range (25th to 75th percentile), GMFR geometric mean fold rise, N number of subjects in the Immunogenicity Set within each visit with non-missing data.
The Immunogenicity Set consists of all enrolled participants who were randomised and received one dose of study vaccine or placebo and provided at least one valid immunogenicity response measured at least 28 days after one dose of study vaccine or placebo.
The 95% CI for GMT and GMFR were calculated based on the t-distribution on the log base 10 transformed values, then back transformed (power of 10) on the original score.
To allow for log-transformation, a value of 0.00001 was added to all values of percentage of total cytokine+ secreting cells in the CD4 population, except the WT RBD peptides for the Total TNF+, TNFa+ and TNFa+ MIP1b+ secreting cells.

Supplementary Table 13: CD8 T cell cytokine responses by intracellular staining (ICS)

		Placebo		Protein-RBD						mRNA-RBD					
				5µg		15µg		45µg		10µg		20µg		50µg	
	N	Day 1	Day 29	Day 1	Day 29	Day 1	Day 29	Day 1	Day 29	Day 1	Day 29	Day 1	Day 29	Day 1	Day 29
		12	12	16	16	8	8	8	8	16	16	8	8	8	8
Total IFNg+															
WT RBD	GMT	0·000	0·001	0·000	0·000	0·000	0·002	0·006	0·020	0·000	0·002	0·001	0·088	0·001	0·006
	95% CI	0·000-0·005	0·000-0·016	0·000-0·003	0·000-0·004	0·000-0·016	0·000-0·072	0·000-0·163	0·001-0·276	0·000-0·006	0·000-0·038	0·000-0·039	0·028-0·274	0·000-0·032	0·000-0·240
	Median (IQR)	0·00 (0·00-0·03)	0·01 (0·00-0·08)	0·00 (0·00-0·05)	0·00 (0·00-0·02)	0·00 (0·00-0·07)	0·01 (0·00-0·08)	0·04 (0·00-0·06)	0·05 (0·03-0·09)	0·00 (0·00-0·05)	0·03 (0·00-0·28)	0·02 (0·00-0·06)	0·10 (0·04-0·18)	0·01 (0·00-0·05)	0·04 (0·00-0·15)
	GMFR referencing Day 1		2·5		1·2		5·8		3·6		5·2		112·4		9·3
	95% CI		0·1-73·8		0·1-9·9		0·3-119·3		0·3-38·8		0·3-104·4		3·1-4074·2		0·9-100·2
Beta RBD	GMT	0·000	0·000	0·000	0·000	0·000	0·001	0·005	0·017	0·001	0·004	0·000	0·010	0·002	0·003
	95% CI	0·000-0·003	0·000-0·006	0·000-0·004	0·000-0·003	0·000-0·012	0·000-0·037	0·000-0·142	0·001-0·225	0·000-0·005	0·000-0·039	0·000-0·007	0·000-0·400	0·000-0·063	0·000-0·178
	Median (IQR)	0·00 (0·00-0·02)	0·00 (0·00-0·03)	0·00 (0·00-0·04)	0·00 (0·00-0·02)	0·00 (0·00-0·02)	0·01 (0·00-0·07)	0·03 (0·00-0·07)	0·05 (0·02-0·07)	0·00 (0·00-0·04)	0·02 (0·00-0·14)	0·00 (0·00-0·03)	0·07 (0·02-0·12)	0·02 (0·00-0·06)	0·05 (0·00-0·15)
	GMFR referencing Day 1		1·5		0·8		1·8		3·3		7·4		50·0		1·9
	95% CI		0·1-44·4		0·1-11·0		0·1-50·2		0·2-46·1		0·4-154·8		0·1-27385·1		0·0-104·3
WT Spike	GMT	0·042	0·013	0·006	0·008	0·009	0·011	0·007	0·001	0·018	0·053	0·020	0·098	0·004	0·171
	95% CI	0·006-0·304	0·001-0·244	0·001-0·052	0·001-0·051	0·000-0·316	0·000-0·453	0·000-0·206	0·000-0·035	0·003-0·102	0·008-0·348	0·001-0·272	0·004-2·405	0·000-0·082	0·034-0·861
	Median (IQR)	0·05 (0·03-0·09)	0·05 (0·01-0·27)	0·03 (0·01-0·08)	0·03 (0·00-0·09)	0·05 (0·01-0·17)	0·11 (0·01-0·22)	0·03 (0·01-0·11)	0·00 (0·00-0·11)	0·05 (0·01-0·11)	0·12 (0·05-0·26)	0·05 (0·03-0·08)	0·32 (0·12-0·70)	0·01 (0·00-0·05)	0·18 (0·07-0·97)
	GMFR referencing Day 1		0·3		1·2		1·3		0·1		2·9		5·0		48·0
	95% CI		0·0-2·3		0·1-12·8		0·0-43·9		0·0-1·8		0·6-13·4		0·1-330·0		1·0-2286·1
Human IgG1 Fc Region	GMT	0·001	0·000	0·000	0·001	0·000	0·001	0·002	0·015	0·000	0·000	0·001	0·002	0·001	0·001
	95% CI	0·000-0·024	0·000-0·009	0·000-0·004	0·000-0·006	0·000-0·008	0·000-0·037	0·000-0·059	0·001-0·227	0·000-0·002	0·000-0·001	0·000-0·019	0·000-0·063	0·000-0·040	0·000-0·035
	Median (IQR)	0·02 (0·00-0·06)	0·00 (0·00-0·06)	0·00 (0·00-0·03)	0·01 (0·00-0·05)	0·00 (0·00-0·03)	0·01 (0·00-0·03)	0·02 (0·00-0·06)	0·07 (0·02-0·09)	0·00 (0·00-0·03)	0·00 (0·00-0·02)	0·01 (0·00-0·03)	0·01 (0·00-0·06)	0·02 (0·00-0·02)	0·02 (0·00-0·05)
	GMFR referencing Day 1		0·3		1·5		5·6		9·8		0·5		3·3		0·6
	95% CI		0·0-2·1		0·2-11·3		0·3-123·4		0·3-368·1		0·1-4·1		0·1-140·4		0·0-6·6
Total TNF+															
WT RBD	GMT	0·000	0·002	0·001	0·000	0·001	0·001	0·000	0·002	0·000	0·002	0·003	0·011	0·001	0·000
	95% CI	0·000-0·004	0·000-0·015	0·000-0·006	0·000-0·001	0·000-0·033	0·000-0·035	0·000-0·015	0·000-0·025	0·000-0·003	0·000-0·015	0·000-0·054	0·001-0·137	0·000-0·018	0·000-0·012
	Median (IQR)	0·00 (0·00-0·01)	0·01 (0·00-0·01)	0·01 (0·00-0·01)	0·00 (0·00-0·01)	0·01 (0·00-0·04)	0·01 (0·00-0·03)	0·00 (0·00-0·02)	0·00 (0·00-0·01)	0·00 (0·00-0·01)	0·01 (0·00-0·05)	0·02 (0·00-0·02)	0·02 (0·02-0·05)	0·00 (0·00-0·04)	0·00 (0·00-0·05)
	GMFR referencing Day 1		6·0		0·3		1·1		3·4		4·3		4·0		0·7
	95% CI		0·8-43·4		0·0-2·0		0·7-1·6		0·5-25·2		0·6-32·2		0·3-51·6		0·0-15·4
Beta RBD	GMT	0·000	0·000	0·000	0·000	0·001	0·002	0·000	0·002	0·000	0·001	0·001	0·003	0·000	0·001
	95% CI	0·000-0·002	0·000-0·005	0·000-0·003	0·000-0·001	0·000-0·035	0·000-0·032	0·000-0·012	0·000-0·034	0·000-0·002	0·000-0·005	0·000-0·019	0·000-0·053	0·000-0·009	0·000-0·026
	Median (IQR)	0·00 (0·00-0·01)	0·00 (0·00-0·01)	0·00 (0·00-0·01)	0·00 (0·00-0·01)	0·01 (0·00-0·02)	0·01 (0·00-0·02)	0·00 (0·00-0·02)	0·01 (0·00-0·02)	0·00 (0·00-0·01)	0·00 (0·00-0·02)	0·01 (0·00-0·01)	0·01 (0·00-0·03)	0·00 (0·00-0·01)	0·01 (0·00-0·02)
	GMFR referencing Day 1		1·9		0·3		1·5		4·9		2·1		3·2		2·8
	95% CI		0·6-6·3		0·0-2·3		0·1-15·0		0·4-54·9		0·4-9·5		0·3-36·2		0·5-15·8
WT Spike	GMT	0·012	0·026	0·002	0·003	0·009	0·006	0·004	0·001	0·019	0·016	0·007	0·022	0·005	0·012
	95% CI	0·001-0·134	0·004-0·172	0·000-0·014	0·000-0·017	0·001-0·102	0·001-0·066	0·000-0·085	0·000-0·019	0·006-0·067	0·003-0·083	0·003-0·019	0·006-0·076	0·000-0·047	0·001-0·199
	Median (IQR)	0·03 (0·01-0·06)	0·03 (0·02-0·06)	0·01 (0·00-0·03)	0·01 (0·00-0·03)	0·02 (0·00-0·04)	0·01 (0·00-0·03)	0·02 (0·00-0·04)	0·00 (0·00-0·05)	0·03 (0·01-0·06)	0·03 (0·02-0·08)	0·00 (0·00-0·01)	0·02 (0·01-0·07)	0·01 (0·00-0·03)	0·04 (0·01-0·11)
	GMFR referencing Day 1		2·1		1·4		0·7		0·2		0·9		3·1		2·5
	95% CI		0·2-23·4		0·2-10·4		0·0-21·6		0·0-2·3		0·3-2·5		1·2-8·1		0·1-51·7
Human IgG1 Fc Region	GMT	0·000	0·001	0·000	0·000	0·001	0·000	0·000	0·002	0·000	0·000	0·000	0·002	0·000	0·000
	95% CI	0·000-0·003	0·000-0·008	0·000-0·002	0·000-0·002	0·000-0·020	0·000-0·013	0·000-0·005	0·000-0·036	0·000-0·000	0·000-0·002	0·000-0·006	0·000-0·033	0·000-0·003	0·000-0·008
	Median (IQR)	0·00 (0·00-0·02)	0·01 (0·00-0·02)	0·00 (0·00-0·01)	0·00 (0·00-0·01)	0·00 (0·00-0·02)	0·00 (0·00-0·01)	0·00 (0·00-0·02)	0·01 (0·00-0·02)	0·00 (0·00-0·00)	0·00 (0·00-0·01)	0·00 (0·00-0·01)	0·01 (0·00-0·02)	0·00 (0·00-0·00)	0·00 (0·00-0·01)
	GMFR referencing Day 1		2·8		0·6		0·5		11·4		3·8		7·3		2·0
	95% CI		0·5-14·5		0·1-3·0		0·1-2·4		0·6-234·3		0·6-22·1		0·1-361·5		0·8-5·1
Total MIP1b+															
WT RBD	GMT	0·000	0·000	0·000	0·000	0·000	0·000	0·000	0·000	0·000	0·000	0·000	0·000	0·000	0·000
	95% CI	0·000-0·000	0·000-0·000	0·000-0·000	0·000-0·000	0·000-0·000	0·000-0·000	0·000-0·002	0·000-0·008	0·000-0·000	0·000-0·000	0·000-0·000	0·000-0·000	0·000-0·000	0·000-0·000
	Median (IQR)	0·00 (0·00-0·00)	0·00 (0·00-0·00)	0·00 (0·00-0·00)	0·00 (0·00-0·00)	0·00 (0·00-0·00)	0·00 (0·00-0·00)	0·00 (0·00-0·01)	0·00 (0·00-0·03)	0·00 (0·00-0·00)	0·00 (0·00-0·00)	0·00 (0·00-0·00)	0·00 (0·00-0·00)	0·00 (0·00-0·00)	0·00 (0·00-0·00)
	GMFR referencing Day 1		0·7		1·5		2·3		2·8		1·0		1·2		2·5
	95% CI		0·3-1·7		0·6-3·7		0·3-17·5		0·2-35·7		1·0-1·0		0·0-30·4		0·3-21·3
Beta RBD	GMT	0·000	0·000	0·000	0·000	0·000	0·000	0·000	0·000	0·000	0·000	0·000	0·000	0·000	0·000
	95% CI	0·000-0·000	0·000-0·000	0·000-0·000	0·000-0·000	0·000-0·000	0·000-0·000	0·000-0·000	0·000-0·004	0·000-0·000	0·000-0·000	0·000-0·000	0·000-0·000	0·000-0·000	0·000-0·000
	Median (IQR)	0·00 (0·00-0·00)	0·00 (0·00-0·00)	0·00 (0·00-0·00)	0·00 (0·00-0·00)	0·00 (0·00-0·00)	0·00 (0·00-0·00)	0·00 (0·00-0·00)	0·00 (0·00-0·01)	0·00 (0·00-0·00)	0·00 (0·00-0·00)	0·00 (0·00-0·00)	0·00 (0·00-0·00)	0·00 (0·00-0·00)	0·00 (0·00-0·00)
	GMFR referencing Day 1		0·6		1·0		1·0		5·8		0·5		0·7		2·1
	95% CI		0·2-1·9		1·0-1·1		1·0-1·0		0·4-76·6		0·1-2·0		0·0-16·7		0·4-12·0
WT Spike	GMT	0·033	0·030	0·008	0·008	0·000	0·021	0·005	0·015	0·031	0·056	0·016	0·070	0·003	0·126
	95% CI	0·004-0·275	0·002-0·406	0·001-0·058	0·001-0·069	0·000-0·025	0·000-1·203	0·000-0·151	0·001-0·194	0·005-0·198	0·008-0·402	0·001-0·241	0·003-1·478	0·000-0·069	0·026-0·618
	Median (IQR)	0·05 (0·02-0·13)	0·11 (0·02-0·18)	0·03 (0·01-0·11)	0·03 (0·00-0·15)	0·00 (0·00-0·12)	0·22 (0·01-0·65)	0·02 (0·00-0·12)	0·03 (0·01-0·10)	0·09 (0·03-0·30)	0·13 (0·04-0·39)	0·05 (0·01-0·13)	0·23 (0·13-0·41)	0·01 (0·00-0·05)	0·23 (0·05-0·56)
	GMFR referencing Day 1		0·9		0·9		55·3		2·8		1·8		4·3		40·7
	95% CI		0·1-6·5		0·2-4·1		0·8-3827·6		0·0-172·6		0·8-4·0		0·1-226·9		1·9-856·2
Human IgG1 Fc Region	GMT	0·000	0·000	0·000	0·000	0·000	0·000	0·000	0·000	0·000	0·000	0·000	0·000	0·000	0·000
	95% CI	0·000-0·000	0·000-0·000	0·000-0·000	0·000-0·000	0·000-0·000	0·000-0·000	0·000-0·002	0·000-0·008	0·000-0·000	0·000-0·000	0·000-0·000	0·000-0·000	0·000-0·000	0·000-0·001
	Median (IQR)	0·00 (0·00-0·00)	0·00 (0·00-0·00)	0·00 (0·00-0·00)	0·00 (0·00-0·00)	0·00 (0·00-0·00)	0·00 (0·00-0·00)	0·00 (0·00-0·01)	0·00 (0·00-0·03)	0·00 (0·00-0·00)	0·00 (0·00-0·00)	0·00 (0·00-0·00)	0·00 (0·00-0·00)	0·00 (0·00-0·00)	0·00 (0·00-0·00)
	GMFR referencing Day 1		0·5		1·1		1·0		2·9		1·0		2·8		4·8
	95% CI		0·1-2·1		0·9-1·3		1·0-1·0		0·2-36·2		1·0-1·0		0·2-33·7		0·4-56·1
Total IL5+															
WT RBD	GMT	0·000	0·001	0·000	0·000	0·000	0·000	0·001	0·000	0·000	0·001	0·000	0·000	0·001	0·000
	95% CI	0·000-0·001	0·000-0·005	0·000-0·001	0·000-0·002	0·000-0·001	0·000-0·004	0·000-0·014	0·000-0·002	0·000-0·002	0·000-0·005	0·000-0·009	0·000-0·009	0·000-0·015	0·000-0·005
	Median (IQR)	0·00 (0·00-0·00)	0·00 (0·00-0·01)	0·00 (0·00-0·00)	0·00 (0·00-0·01)	0·00 (0·00-0·01)	0·00 (0·00-0·02)	0·00 (0·00-0·01)	0·00 (0·00-0·01)	0·00 (0·00-0·02)	0·00 (0·00-0·01)	0·00 (0·00-0·01)	0·01 (0·00-0·01)	0·00 (0·00-0·01)	0·00 (0·00-0·01)
	GMFR referencing Day 1		5·0		2·4		2·4		0·1		2·4		1·0		0·3
	95% CI		0·4-68·0		0·1-46·4		0·0-493·8		0·0-1·1		0·2-37·7		0·0-306·5		0·0-24·1
Beta RBD	GMT	0·001	0·000	0·001	0·000	0·000	0·000	0·001	0·000	0·001	0·002	0·000	0·001	0·001	0·001
	95% CI	0·000-0·006	0·000-0·002	0·000-0·008	0·000-0·002	0·000-0·006	0·000-0·006	0·000-0·013	0·000-0·003	0·000-0·006	0·000-0·012	0·000-0·006	0·000-0·018	0·000-0·014	0·000-0·018
	Median (IQR)	0·01 (0·00-0·01)	0·00 (0·00-0·02)	0·00 (0·00-0·02)	0·00 (0·00-0·01)	0·00 (0·00-0·02)	0·00 (0·00-0·01)	0·00 (0·00-0·01)	0·00 (0·00-0·00)	0·01 (0·00-0·02)	0·01 (0·00-0·02)	0·00 (0·00-0·02)	0·01 (0·00-0·01)	0·00 (0·00-0·01)	0·00 (0·00-0·03)
	GMFR referencing Day 1		0·3		0·3		1·1		0·2		2·4		4·6		0·7
	95% CI		0·0-4·6		0·0-4·7		0·0-351·7		0·0-8·8		0·2-24·1		0·2-135·9		0·1-9·5
WT Spike	GMT	0·000	0·000	0·000	0·000	0·000	0·001	0·000	0·000	0·000	0·000	0·001	0·000	0·000	0·000
	95% CI	0·000-0·004	0·000-0·004	0·000-0·001	0·000-0·000	0·000-0·005	0·000-0·013	0·000-0·003	0·000-0·000	0·000-0·002	0·000-0·001	0·000-0·012	0·000-0·001	0·000-0·003	0·000-0·005
	Median (IQR)	0·00 (0·00-0·01)	0·00 (0·00-0·01)	0·00 (0·00-0·01)	0·00 (0·00-0·00)	0·00 (0·00-0·02)	0·01 (0·00-0·01)	0·00 (0·00-0·00)	0·00 (0·00-0·00)	0·00 (0·00-0·01)	0·00 (0·00-0·01)	0·00 (0·00-0·02)	0·00 (0·00-0·00)	0·00 (0·00-0·00)	0·00 (0·00-0·01)
	GMFR referencing Day 1		0·9		0·6		3·8		0·2		0·4		0·1		1·4
	95% CI		0·0-21·7		0·0-9·3		0·0-770·5		0·0-6·4		0·0-7·9		0·0-6·1		0·0-83·2
Human IgG1 Fc Region	GMT	0·001	0·000	0·000	0·000	0·000	0·000	0·001	0·000	0·000	0·001	0·000	0·000	0·005	0·000
	95% CI	0·000-0·008	0·000-0·001	0·000-0·002	0·000-0·001	0·000-0·004	0·000-0·005	0·000-0·009	0·000-0·005	0·000-0·001	0·000-0·004	0·000-0·007	0·000-0·005	0·002-0·011	0·000-0·009
	Median (IQR)	0·00 (0·00-0·02)	0·00 (0·00-0·01)	0·00 (0·00-0·01)	0·00 (0·00-0·02)	0·00 (0·00-0·01)	0·00 (0·00-0·01)	0·00 (0·00-0·01)	0·00 (0·00-0·01)	0·00 (0·00-0·01)	0·00 (0·00-0·01)	0·00 (0·00-0·01)	0·00 (0·00-0·01)	0·00 (0·00-0·01)	0·00 (0·00-0·02)
	GMFR referencing Day 1		0·2		0·5		1·6		0·5		2·7		0·8		0·1
	95% CI		0·0-2·5		0·0-7·8		0·0-558·9		0·0-12·5		0·7-10·6		0·0-78·4		0·0-1·2
Total IL13+															
WT RBD	GMT	0·011	0·003	0·004	0·009	0·003	0·026	0·024	0·020	0·007	0·004	0·008	0·004	0·003	0·017
	95% CI	0·002-0·047	0·000-0·032	0·001-0·019	0·002-0·039	0·000-0·052	0·015-0·046	0·008-0·068	0·006-0·068	0·001-0·039	0·001-0·031	0·001-0·080	0·000-0·043	0·000-0·074	0·007-0·040
	Median (IQR)	0·02 (0·01-0·04)	0·02 (0·00-0·04)	0·02 (0·00-0·03)	0·02 (0·01-0·03)	0·02 (0·00-0·02)	0·03 (0·01-0·05)	0·05 (0·01-0·06)	0·03 (0·01-0·07)	0·02 (0·01-0·05)	0·03 (0·00-0·06)	0·02 (0·01-0·03)	0·01 (0·00-0·03)	0·02 (0·00-0·03)	0·02 (0·01-0·04)
	GMFR referencing Day 1		0·3		2·5		9·4		0·8		0·7		0·5		5·0
	95% CI		0·0-4·9		0·2-25·3		0·6-154·3		0·3-2·2		0·1-6·2		0·0-14·5		0·2-132·5
Beta RBD	GMT	0·021	0·006	0·009	0·006	0·009	0·013	0·012	0·019	0·008	0·011	0·021	0·018	0·003	0·023
	95% CI	0·015-0·032	0·001-0·041	0·002-0·038	0·001-0·033	0·001-0·101	0·005-0·038	0·002-0·085	0·005-0·071	0·002-0·027	0·002-0·051	0·010-0·042	0·013-0·026	0·000-0·065	0·008-0·062
	Median (IQR)	0·02 (0·02-0·03)	0·02 (0·01-0·03)	0·02 (0·01-0·04)	0·02 (0·01-0·03)	0·03 (0·01-0·03)	0·02 (0·00-0·04)	0·03 (0·01-0·06)	0·03 (0·00-0·08)	0·01 (0·00-0·02)	0·02 (0·01-0·05)	0·02 (0·01-0·04)	0·02 (0·01-0·03)	0·01 (0·00-0·03)	0·03 (0·02-0·04)
	GMFR referencing Day 1		0·3		0·7		1·4		1·5		1·4		0·9		7·7
	95% CI		0·0-2·0		0·1-7·2		0·2-13·2		0·4-6·1		0·4-4·8		0·4-2·0		0·4-159·2
WT Spike	GMT	0·000	0·001	0·000	0·000	0·000	0·000	0·000	0·000	0·000	0·000	0·000	0·001	0·000	0·000
	95% CI	0·000-0·001	0·000-0·006	0·000-0·000	0·000-0·001	0·000-0·001	0·000-0·003	0·000-0·000	0·000-0·003	0·000-0·000	0·000-0·001	0·000-0·004	0·000-0·014	0·000-0·001	0·000-0·003
	Median (IQR)	0·00 (0·00-0·01)	0·01 (0·00-0·01)	0·00 (0·00-0·00)	0·00 (0·00-0·01)	0·00 (0·00-0·00)	0·00 (0·00-0·00)	0·00 (0·00-0·00)	0·00 (0·00-0·01)	0·00 (0·00-0·01)	0·00 (0·00-0·00)	0·00 (0·00-0·01)	0·01 (0·00-0·01)	0·00 (0·00-0·00)	0·00 (0·00-0·01)
	GMFR referencing Day 1		3·9		2·0		5·0		4·9		1·4		4·5		2·7
	95% CI		0·8-18·6		0·1-33·8		0·2-139·8		0·4-67·9		0·2-12·2		0·1-336·4		0·0-268·6
Human IgG1 Fc Region	GMT	0·007	0·004	0·004	0·009	0·004	0·004	0·013	0·024	0·006	0·013	0·007	0·003	0·005	0·031
	95% CI	0·001-0·051	0·001-0·024	0·001-0·022	0·002-0·039	0·000-0·042	0·000-0·082	0·001-0·160	0·006-0·096	0·001-0·036	0·004-0·040	0·001-0·074	0·000-0·056	0·000-0·063	0·021-0·048
	Median (IQR)	0·02 (0·02-0·03)	0·01 (0·00-0·03)	0·01 (0·01-0·02)	0·02 (0·01-0·04)	0·01 (0·00-0·02)	0·02 (0·00-0·04)	0·04 (0·01-0·08)	0·05 (0·01-0·09)	0·02 (0·01-0·04)	0·02 (0·01-0·04)	0·01 (0·01-0·04)	0·02 (0·00-0·03)	0·01 (0·00-0·04)	0·04 (0·02-0·05)
	GMFR referencing Day 1		0·5		2·0		0·8		1·9		2·0		0·4		6·0
	95% CI		0·0-10·8		0·2-17·3		0·0-48·8		0·2-17·9		0·3-15·6		0·0-22·0		0·5-76·0
IFNg+															
WT RBD	GMT	0·003	0·003	0·002	0·007	0·006	0·004	0·006	0·026	0·002	0·045	0·009	0·101	0·011	0·049
	95% CI	0·000-0·035	0·000-0·050	0·000-0·015	0·001-0·041	0·000-0·111	0·000-0·123	0·000-0·174	0·011-0·059	0·000-0·021	0·011-0·183	0·001-0·130	0·044-0·231	0·001-0·128	0·012-0·195
	Median (IQR)	0·02 (0·00-0·05)	0·03 (0·00-0·08)	0·02 (0·00-0·05)	0·02 (0·01-0·05)	0·03 (0·00-0·09)	0·02 (0·00-0·10)	0·04 (0·01-0·09)	0·03 (0·01-0·06)	0·02 (0·00-0·08)	0·05 (0·02-0·23)	0·03 (0·00-0·09)	0·09 (0·04-0·18)	0·03 (0·01-0·05)	0·07 (0·03-0·14)
	GMFR referencing Day 1		1·0		3·7		0·7		4·3		22·1		10·9		4·4
	95% CI		0·1-12·5		0·6-22·1		0·0-35·0		0·2-90·8		2·3-212·7		0·9-134·5		0·3-74·3
Beta RBD	GMT	0·002	0·002	0·003	0·005	0·004	0·005	0·007	0·002	0·003	0·043	0·003	0·060	0·004	0·051
	95% CI	0·000-0·023	0·000-0·032	0·000-0·019	0·001-0·035	0·000-0·110	0·000-0·141	0·000-0·199	0·000-0·071	0·000-0·022	0·023-0·081	0·000-0·069	0·021-0·167	0·000-0·096	0·020-0·130
	Median (IQR)	0·02 (0·00-0·04)	0·03 (0·00-0·05)	0·01 (0·00-0·05)	0·03 (0·01-0·05)	0·03 (0·00-0·08)	0·03 (0·00-0·09)	0·05 (0·01-0·07)	0·03 (0·00-0·05)	0·01 (0·00-0·07)	0·04 (0·02-0·11)	0·02 (0·00-0·03)	0·06 (0·03-0·12)	0·02 (0·01-0·04)	0·05 (0·03-0·13)
	GMFR referencing Day 1		1·2		1·8		1·3		0·3		13·9		19·6		12·2
	95% CI		0·1-25·9		0·2-18·1		0·1-28·8		0·0-92·3		2·8-69·3		1·0-373·9		0·8-182·8
WT Spike	GMT	0·005	0·001	0·006	0·001	0·002	0·022	0·007	0·000	0·001	0·011	0·012	0·060	0·002	0·016
	95% CI	0·000-0·051	0·000-0·022	0·001-0·025	0·000-0·008	0·000-0·052	0·008-0·062	0·001-0·078	0·000-0·002	0·000-0·008	0·002-0·075	0·001-0·152	0·003-1·232	0·000-0·024	0·000-0·786
	Median (IQR)	0·03 (0·00-0·04)	0·01 (0·00-0·05)	0·01 (0·01-0·03)	0·01 (0·00-0·02)	0·02 (0·00-0·04)	0·03 (0·01-0·06)	0·02 (0·01-0·03)	0·00 (0·00-0·02)	0·00 (0·00-0·02)	0·03 (0·01-0·11)	0·03 (0·01-0·06)	0·18 (0·07-0·34)	0·01 (0·00-0·01)	0·09 (0·02-0·45)
	GMFR referencing Day 1		0·3		0·2		14·5		0·0		8·5		4·8		9·6
	95% CI		0·0-2·4		0·0-1·6		0·4-480·2		0·0-0·3		0·5-135·9		0·1-194·2		0·0-2100·5
Human IgG1 Fc Region	GMT	0·006	0·002	0·005	0·007	0·007	0·020	0·011	0·027	0·015	0·004	0·001	0·021	0·009	0·015
	95% CI	0·001-0·050	0·000-0·024	0·001-0·028	0·001-0·041	0·001-0·089	0·009-0·048	0·001-0·131	0·017-0·044	0·005-0·047	0·001-0·021	0·000-0·042	0·006-0·067	0·001-0·091	0·001-0·185
	Median (IQR)	0·02 (0·00-0·07)	0·00 (0·00-0·10)	0·02 (0·01-0·04)	0·02 (0·01-0·06)	0·02 (0·00-0·04)	0·02 (0·01-0·04)	0·03 (0·01-0·06)	0·02 (0·02-0·04)	0·02 (0·01-0·05)	0·01 (0·00-0·04)	0·00 (0·00-0·06)	0·03 (0·02-0·05)	0·02 (0·01-0·04)	0·04 (0·02-0·07)
	GMFR referencing Day 1		0·3		1·4		2·8		2·4		0·3		17·0		1·7
	95% CI		0·0-3·4		0·2-10·2		0·4-19·9		0·2-38·9		0·0-2·2		0·9-317·0		0·1-58·7
TNFa+															
WT RBD	GMT	0·002	0·009	0·007	0·007	0·005	0·005	0·009	0·006	0·008	0·012	0·011	0·012	0·010	0·005
	95% CI	0·000-0·017	0·005-0·016	0·002-0·019	0·004-0·011	0·001-0·044	0·001-0·047	0·003-0·024	0·002-0·017	0·003-0·020	0·007-0·019	0·008-0·015	0·005-0·028	0·004-0·026	0·001-0·044
	Median (IQR)	0·01 (0·00-0·01)	0·01 (0·01-0·01)	0·01 (0·01-0·02)	0·01 (0·00-0·02)	0·01 (0·00-0·02)	0·01 (0·01-0·02)	0·01 (0·00-0·02)	0·01 (0·00-0·01)	0·01 (0·01-0·02)	0·01 (0·01-0·02)	0·01 (0·01-0·01)	0·01 (0·01-0·03)	0·01 (0·00-0·02)	0·01 (0·00-0·02)
	GMFR referencing Day 1		4·2		1·0		1·1		0·7		1·5		1·1		0·5
	95% CI		0·7-24·5		0·3-2·7		0·7-1·5		0·2-2·4		0·6-3·8		0·5-2·5		0·1-2·6
Beta RBD	GMT	0·003	0·008	0·003	0·002	0·003	0·003	0·006	0·005	0·006	0·007	0·007	0·003	0·004	0·002
	95% CI	0·001-0·014	0·006-0·011	0·001-0·011	0·000-0·008	0·000-0·054	0·000-0·029	0·002-0·019	0·002-0·017	0·003-0·014	0·003-0·020	0·004-0·015	0·000-0·023	0·000-0·040	0·000-0·031
	Median (IQR)	0·01 (0·00-0·01)	0·01 (0·01-0·01)	0·01 (0·00-0·01)	0·01 (0·00-0·01)	0·01 (0·01-0·02)	0·01 (0·00-0·01)	0·01 (0·00-0·02)	0·01 (0·00-0·01)	0·01 (0·01-0·02)	0·01 (0·01-0·02)	0·01 (0·00-0·01)	0·00 (0·00-0·02)	0·01 (0·00-0·01)	0·01 (0·00-0·01)
	GMFR referencing Day 1		2·3		0·6		1·2		0·8		1·1		0·4		0·4
	95% CI		0·7-7·7		0·2-2·5		0·1-13·1		0·2-2·7		0·4-3·4		0·1-2·0		0·0-4·0
WT Spike	GMT	0·000	0·000	0·000	0·000	0·000	0·000	0·000	0·000	0·001	0·000	0·001	0·001	0·000	0·000
	95% CI	0·000-0·002	0·000-0·003	0·000-0·000	0·000-0·000	0·000-0·004	0·000-0·002	0·000-0·002	0·000-0·002	0·000-0·003	0·000-0·001	0·000-0·005	0·000-0·005	0·000-0·002	0·000-0·002
	Median (IQR)	0·00 (0·00-0·00)	0·00 (0·00-0·00)	0·00 (0·00-0·00)	0·00 (0·00-0·00)	0·00 (0·00-0·00)	0·00 (0·00-0·00)	0·00 (0·00-0·00)	0·00 (0·00-0·00)	0·00 (0·00-0·00)	0·00 (0·00-0·00)	0·00 (0·00-0·00)	0·00 (0·00-0·00)	0·00 (0·00-0·00)	0·00 (0·00-0·00)
	GMFR referencing Day 1		1·4		1·0		0·7		0·7		0·3		0·6		1·0
	95% CI		0·1-23·1		0·2-5·9		0·0-13·2		0·0-9·0		0·1-1·0		0·0-13·6		0·0-19·6
Human IgG1 Fc Region	GMT	0·005	0·006	0·003	0·002	0·004	0·002	0·002	0·007	0·007	0·004	0·008	0·004	0·002	0·004
	95% CI	0·001-0·020	0·001-0·021	0·001-0·012	0·001-0·011	0·000-0·045	0·000-0·036	0·000-0·032	0·003-0·016	0·004-0·013	0·001-0·014	0·005-0·013	0·001-0·019	0·000-0·028	0·000-0·035
	Median (IQR)	0·01 (0·01-0·01)	0·01 (0·00-0·02)	0·01 (0·00-0·01)	0·01 (0·00-0·02)	0·01 (0·00-0·02)	0·01 (0·00-0·02)	0·01 (0·00-0·02)	0·01 (0·00-0·01)	0·01 (0·01-0·01)	0·01 (0·00-0·02)	0·01 (0·00-0·01)	0·01 (0·00-0·02)	0·01 (0·00-0·01)	0·01 (0·00-0·01)
	GMFR referencing Day 1		1·0		0·8		0·5		3·6		0·5		0·6		2·3
	95% CI		0·5-2·0		0·2-2·6		0·1-3·1		0·4-33·5		0·2-1·5		0·2-1·9		0·1-57·2
MIP1b+															
WT RBD	GMT	0·000	0·000	0·000	0·000	0·000	0·000	0·000	0·000	0·000	0·000	0·000	0·000	0·000	0·000
	95% CI	0·000-0·000	0·000-0·000	0·000-0·000	0·000-0·000	0·000-0·000	0·000-0·000	0·000-0·000	0·000-0·000	0·000-0·000	0·000-0·000	0·000-0·000	0·000-0·000	0·000-0·000	0·000-0·000
	Median (IQR)	0·00 (0·00-0·00)	0·00 (0·00-0·00)	0·00 (0·00-0·00)	0·00 (0·00-0·00)	0·00 (0·00-0·00)	0·00 (0·00-0·00)	0·00 (0·00-0·00)	0·00 (0·00-0·00)	0·00 (0·00-0·00)	0·00 (0·00-0·00)	0·00 (0·00-0·00)	0·00 (0·00-0·00)	0·00 (0·00-0·00)	0·00 (0·00-0·00)
	GMFR referencing Day 1		0·7		1·0		1·0		1·0		1·0		0·5		1·0
	95% CI		0·3-1·7		1·0-1·0		1·0-1·0		1·0-1·0		1·0-1·0		0·1-2·6		1·0-1·0
Beta RBD	GMT	0·000	0·000	0·000	0·000	0·000	0·000	0·000	0·000	0·000	0·000	0·000	0·000	0·000	0·000
	95% CI	0·000-0·000	0·000-0·000	0·000-0·000	0·000-0·000	0·000-0·000	0·000-0·000	0·000-0·000	0·000-0·000	0·000-0·000	0·000-0·000	0·000-0·000	0·000-0·000	0·000-0·000	0·000-0·000
	Median (IQR)	0·00 (0·00-0·00)	0·00 (0·00-0·00)	0·00 (0·00-0·00)	0·00 (0·00-0·00)	0·00 (0·00-0·00)	0·00 (0·00-0·00)	0·00 (0·00-0·00)	0·00 (0·00-0·00)	0·00 (0·00-0·00)	0·00 (0·00-0·00)	0·00 (0·00-0·00)	0·00 (0·00-0·00)	0·00 (0·00-0·00)	0·00 (0·00-0·00)
	GMFR referencing Day 1		1·0		1·0		1·0		1·0		1·0		0·5		1·0
	95% CI		1·0-1·0		1·0-1·0		1·0-1·0		1·0-1·0		1·0-1·0		0·1-2·5		1·0-1·0
WT Spike	GMT	0·008	0·004	0·003	0·003	0·001	0·010	0·002	0·017	0·008	0·020	0·001	0·014	0·000	0·015
	95% CI	0·001-0·072	0·000-0·070	0·001-0·023	0·000-0·027	0·000-0·024	0·000-0·507	0·000-0·043	0·009-0·031	0·001-0·068	0·002-0·163	0·000-0·055	0·001-0·217	0·000-0·005	0·001-0·208
	Median (IQR)	0·02 (0·00-0·06)	0·04 (0·00-0·10)	0·01 (0·00-0·04)	0·02 (0·00-0·08)	0·01 (0·00-0·05)	0·05 (0·00-0·54)	0·01 (0·00-0·03)	0·02 (0·01-0·03)	0·03 (0·00-0·13)	0·07 (0·02-0·16)	0·01 (0·00-0·08)	0·03 (0·01-0·13)	0·00 (0·00-0·01)	0·04 (0·01-0·11)
	GMFR referencing Day 1		0·5		0·9		17·2		7·2		2·6		15·3		56·9
	95% CI		0·0-9·9		0·1-7·0		0·1-2918·5		0·4-138·9		0·3-21·6		0·7-347·2		1·7-1938·3
Human IgG1 Fc Region	GMT	0·000	0·000	0·000	0·000	0·000	0·000	0·000	0·000	0·000	0·000	0·000	0·000	0·000	0·000
	95% CI	0·000-0·000	0·000-0·000	0·000-0·000	0·000-0·000	0·000-0·000	0·000-0·000	0·000-0·000	0·000-0·000	0·000-0·000	0·000-0·000	0·000-0·000	0·000-0·000	0·000-0·000	0·000-0·000
	Median (IQR)	0·00 (0·00-0·00)	0·00 (0·00-0·00)	0·00 (0·00-0·00)	0·00 (0·00-0·00)	0·00 (0·00-0·00)	0·00 (0·00-0·00)	0·00 (0·00-0·00)	0·00 (0·00-0·00)	0·00 (0·00-0·00)	0·00 (0·00-0·00)	0·00 (0·00-0·00)	0·00 (0·00-0·00)	0·00 (0·00-0·00)	0·00 (0·00-0·00)

	GMFR referencing Day 1		0·6		1·0		1·0		1·0		1·0		1·0		1·0
	95% CI		0·2-1·8		1·0-1·0		1·0-1·0		1·0-1·0		1·0-1·0		1·0-1·0		1·0-1·0
IL5+															
WT RBD	GMT	0·000	0·000	0·000	0·000	0·000	0·000	0·002	0·000	0·000	0·000	0·001	0·000	0·003	0·000
	95% CI	0·000-0·001	0·000-0·004	0·000-0·000	0·000-0·002	0·000-0·001	0·000-0·003	0·000-0·015	0·000-0·002	0·000-0·002	0·000-0·003	0·000-0·022	0·000-0·008	0·000-0·021	0·000-0·003
	Median (IQR)	0·00 (0·00-0·01)	0·00 (0·00-0·01)	0·00 (0·00-0·00)	0·00 (0·00-0·01)	0·00 (0·00-0·01)	0·00 (0·00-0·01)	0·01 (0·00-0·01)	0·00 (0·00-0·01)	0·00 (0·00-0·01)	0·00 (0·00-0·01)	0·01 (0·00-0·02)	0·00 (0·00-0·01)	0·01 (0·00-0·01)	0·00 (0·00-0·01)
	GMFR referencing Day 1		2·9		3·5		2·3		0·1		1·1		0·4		0·0
	95% CI		0·2-38·4		0·2-74·8		0·0-394·7		0·0-0·6		0·1-14·4		0·0-63·9		0·0-0·9
Beta RBD	GMT	0·001	0·000	0·002	0·000	0·000	0·000	0·001	0·000	0·001	0·002	0·001	0·001	0·002	0·000
	95% CI	0·000-0·009	0·000-0·004	0·000-0·012	0·000-0·002	0·000-0·004	0·000-0·003	0·000-0·014	0·000-0·001	0·000-0·004	0·000-0·008	0·000-0·021	0·000-0·014	0·000-0·017	0·000-0·011
	Median (IQR)	0·01 (0·00-0·01)	0·00 (0·00-0·01)	0·01 (0·00-0·01)	0·00 (0·00-0·01)	0·00 (0·00-0·01)	0·00 (0·00-0·01)	0·00 (0·00-0·01)	0·00 (0·00-0·00)	0·00 (0·00-0·02)	0·00 (0·00-0·01)	0·01 (0·00-0·01)	0·00 (0·00-0·01)	0·00 (0·00-0·01)	0·00 (0·00-0·03)
	GMFR referencing Day 1		0·4		0·1		0·8		0·1		2·5		1·1		0·2
	95% CI		0·0-10·4		0·0-2·3		0·0-229·2		0·0-10·0		0·2-25·1		0·0-59·0		0·0-3·4
WT Spike	GMT	0·000	0·000	0·000	0·000	0·000	0·000	0·001	0·000	0·000	0·000	0·001	0·000	0·000	0·000
	95% CI	0·000-0·002	0·000-0·004	0·000-0·001	0·000-0·000	0·000-0·003	0·000-0·007	0·000-0·010	0·000-0·000	0·000-0·002	0·000-0·001	0·000-0·022	0·000-0·001	0·000-0·004	0·000-0·003
	Median (IQR)	0·00 (0·00-0·01)	0·00 (0·00-0·01)	0·00 (0·00-0·01)	0·00 (0·00-0·00)	0·00 (0·00-0·01)	0·00 (0·00-0·01)	0·00 (0·00-0·01)	0·00 (0·00-0·00)	0·00 (0·00-0·01)	0·00 (0·00-0·00)	0·01 (0·00-0·02)	0·00 (0·00-0·00)	0·00 (0·00-0·00)	0·00 (0·00-0·00)
	GMFR referencing Day 1		1·6		0·3		2·4		0·1		0·5		0·1		0·5
	95% CI		0·1-45·5		0·0-4·8		0·0-264·1		0·0-3·4		0·0-9·0		0·0-6·0		0·0-30·5
Human IgG1 Fc Region	GMT	0·002	0·000	0·000	0·000	0·000	0·000	0·003	0·000	0·000	0·000	0·000	0·000	0·006	0·000
	95% CI	0·000-0·014	0·000-0·002	0·000-0·002	0·000-0·001	0·000-0·007	0·000-0·002	0·000-0·017	0·000-0·000	0·000-0·002	0·000-0·003	0·000-0·004	0·000-0·003	0·003-0·013	0·000-0·007
	Median (IQR)	0·01 (0·00-0·02)	0·00 (0·00-0·01)	0·00 (0·00-0·01)	0·00 (0·00-0·01)	0·00 (0·00-0·01)	0·00 (0·00-0·01)	0·00 (0·00-0·01)	0·00 (0·00-0·00)	0·00 (0·00-0·01)	0·00 (0·00-0·01)	0·00 (0·00-0·00)	0·00 (0·00-0·00)	0·01 (0·00-0·01)	0·00 (0·00-0·01)
	GMFR referencing Day 1		0·1		0·4		0·3		0·0		1·7		0·9		0·1
	95% CI		0·0-2·4		0·0-6·7		0·0-47·1		0·0-0·3		0·3-9·0		0·0-42·7		0·0-0·8
IL13+															
WT RBD	GMT	0·001	0·000	0·001	0·001	0·000	0·007	0·001	0·000	0·001	0·001	0·004	0·000	0·000	0·002
	95% CI	0·000-0·004	0·000-0·002	0·000-0·003	0·000-0·007	0·000-0·002	0·003-0·014	0·000-0·014	0·000-0·007	0·000-0·003	0·000-0·006	0·000-0·029	0·000-0·001	0·000-0·009	0·000-0·026
	Median (IQR)	0·00 (0·00-0·00)	0·00 (0·00-0·01)	0·00 (0·00-0·01)	0·01 (0·00-0·01)	0·00 (0·00-0·01)	0·01 (0·00-0·01)	0·00 (0·00-0·01)	0·00 (0·00-0·01)	0·00 (0·00-0·01)	0·00 (0·00-0·01)	0·01 (0·00-0·01)	0·00 (0·00-0·00)	0·00 (0·00-0·01)	0·00 (0·00-0·02)
	GMFR referencing Day 1		0·4		1·9		60·0		0·4		2·1		0·0		4·5
	95% CI		0·0-7·9		0·1-35·8		2·2-1607·5		0·1-2·8		0·3-15·1		0·0-0·2		0·1-140·5
Beta RBD	GMT	0·001	0·001	0·001	0·002	0·001	0·003	0·001	0·000	0·000	0·002	0·002	0·000	0·003	0·000
	95% CI	0·000-0·005	0·000-0·006	0·000-0·005	0·001-0·010	0·000-0·012	0·000-0·029	0·000-0·009	0·000-0·008	0·000-0·001	0·000-0·008	0·000-0·026	0·000-0·004	0·000-0·020	0·000-0·005
	Median (IQR)	0·00 (0·00-0·01)	0·00 (0·00-0·01)	0·00 (0·00-0·02)	0·01 (0·00-0·01)	0·00 (0·00-0·01)	0·01 (0·00-0·02)	0·00 (0·00-0·01)	0·00 (0·00-0·01)	0·00 (0·00-0·01)	0·01 (0·00-0·01)	0·01 (0·00-0·02)	0·00 (0·00-0·01)	0·00 (0·00-0·01)	0·00 (0·00-0·01)
	GMFR referencing Day 1		1·5		3·1		3·3		0·9		6·1		0·2		0·1
	95% CI		0·2-9·3		0·6-16·3		0·1-188·0		0·0-42·5		0·8-46·1		0·0-8·0		0·0-1·0
WT Spike	GMT	0·000	0·000	0·000	0·000	0·000	0·000	0·001	0·000	0·000	0·000	0·001	0·000	0·002	0·001
	95% CI	0·000-0·001	0·000-0·001	0·000-0·001	0·000-0·003	0·000-0·001	0·000-0·007	0·000-0·011	0·000-0·005	0·000-0·001	0·000-0·000	0·000-0·010	0·000-0·001	0·001-0·006	0·000-0·013
	Median (IQR)	0·00 (0·00-0·00)	0·00 (0·00-0·00)	0·00 (0·00-0·01)	0·00 (0·00-0·01)	0·00 (0·00-0·01)	0·00 (0·00-0·01)	0·00 (0·00-0·01)	0·00 (0·00-0·00)	0·00 (0·00-0·00)	0·00 (0·00-0·00)	0·00 (0·00-0·01)	0·00 (0·00-0·00)	0·00 (0·00-0·00)	0·00 (0·00-0·01)
	GMFR referencing Day 1		1·3		4·0		8·0		0·4		0·6		0·2		0·3
	95% CI		0·1-24·4		0·5-29·6		0·1-514·4		0·1-2·5		0·1-6·8		0·0-1·6		0·0-5·9
Human IgG1 Fc Region	GMT	0·001	0·001	0·000	0·002	0·000	0·001	0·000	0·000	0·000	0·001	0·002	0·000	0·001	0·001
	95% CI	0·000-0·007	0·000-0·005	0·000-0·002	0·000-0·008	0·000-0·002	0·000-0·025	0·000-0·007	0·000-0·006	0·000-0·003	0·000-0·008	0·000-0·025	0·000-0·001	0·000-0·009	0·000-0·008
	Median (IQR)	0·00 (0·00-0·01)	0·00 (0·00-0·01)	0·00 (0·00-0·01)	0·01 (0·00-0·01)	0·00 (0·00-0·01)	0·01 (0·00-0·02)	0·00 (0·00-0·01)	0·00 (0·00-0·00)	0·00 (0·00-0·01)	0·01 (0·00-0·01)	0·01 (0·00-0·01)	0·00 (0·00-0·00)	0·00 (0·00-0·01)	0·00 (0·00-0·00)
	GMFR referencing Day 1		0·5		5·5		8·6		1·3		3·4		0·0		1·3
	95% CI		0·1-2·3		0·6-47·3		0·0-1545·0		0·0-70·3		0·3-43·4		0·0-0·8		0·0-69·9
IFNg+ TNFa+															
WT RBD	GMT	0·004	0·001	0·001	0·001	0·001	0·000	0·003	0·001	0·001	0·003	0·004	0·006	0·000	0·000
	95% CI	0·001-0·015	0·000-0·008	0·000-0·002	0·000-0·003	0·000-0·010	0·000-0·001	0·000-0·027	0·000-0·020	0·000-0·004	0·001-0·016	0·000-0·033	0·001-0·062	0·000-0·004	0·000-0·006
	Median (IQR)	0·00 (0·00-0·01)	0·00 (0·00-0·01)	0·00 (0·00-0·00)	0·00 (0·00-0·00)	0·00 (0·00-0·01)	0·00 (0·00-0·00)	0·00 (0·00-0·01)	0·00 (0·00-0·01)	0·00 (0·00-0·02)	0·00 (0·00-0·04)	0·01 (0·00-0·02)	0·01 (0·00-0·03)	0·00 (0·00-0·00)	0·00 (0·00-0·01)
	GMFR referencing Day 1		0·3		1·3		0·1		0·5		5·8		1·5		0·8
	95% CI		0·0-1·8		0·3-6·1		0·0-1·6		0·1-4·1		1·0-33·6		0·6-3·6		0·0-21·3
Beta RBD	GMT	0·002	0·001	0·000	0·000	0·000	0·000	0·001	0·001	0·001	0·001	0·001	0·001	0·000	0·006
	95% CI	0·001-0·010	0·000-0·007	0·000-0·001	0·000-0·001	0·000-0·003	0·000-0·003	0·000-0·016	0·000-0·015	0·000-0·003	0·000-0·007	0·000-0·010	0·000-0·022	0·000-0·002	0·003-0·014
	Median (IQR)	0·00 (0·00-0·00)	0·00 (0·00-0·01)	0·00 (0·00-0·00)	0·00 (0·00-0·00)	0·00 (0·00-0·00)	0·00 (0·00-0·00)	0·00 (0·00-0·01)	0·01 (0·00-0·01)	0·00 (0·00-0·02)	0·00 (0·00-0·03)	0·00 (0·00-0·00)	0·00 (0·00-0·01)	0·00 (0·00-0·01)	0·01 (0·00-0·01)
	GMFR referencing Day 1		0·4		1·2		1·1		1·0		1·7		1·0		53·5
	95% CI		0·2-1·3		0·1-9·5		0·1-13·0		0·0-70·8		0·4-8·1		0·0-22·3		3·9-735·6
WT Spike	GMT	0·002	0·003	0·001	0·001	0·001	0·000	0·001	0·000	0·001	0·002	0·000	0·003	0·000	0·001
	95% CI	0·000-0·014	0·000-0·016	0·000-0·003	0·000-0·004	0·000-0·016	0·000-0·007	0·000-0·008	0·000-0·001	0·000-0·004	0·000-0·007	0·000-0·000	0·000-0·032	0·000-0·001	0·000-0·008
	Median (IQR)	0·00 (0·00-0·02)	0·00 (0·00-0·01)	0·00 (0·00-0·00)	0·00 (0·00-0·00)	0·00 (0·00-0·01)	0·00 (0·00-0·01)	0·00 (0·00-0·01)	0·00 (0·00-0·00)	0·00 (0·00-0·01)	0·00 (0·00-0·01)	0·00 (0·00-0·00)	0·00 (0·00-0·02)	0·00 (0·00-0·00)	0·00 (0·00-0·00)
	GMFR referencing Day 1		1·2		2·3		0·4		0·2		1·6		70·2		8·8
	95% CI		0·6-2·2		0·5-11·3		0·0-6·0		0·0-5·7		0·4-6·6		5·3-923·3		0·8-104·0
Human IgG1 Fc Region	GMT	0·001	0·001	0·000	0·000	0·000	0·000	0·001	0·001	0·000	0·000	0·000	0·000	0·000	0·004
	95% CI	0·000-0·006	0·000-0·006	0·000-0·002	0·000-0·001	0·000-0·003	0·000-0·004	0·000-0·012	0·000-0·013	0·000-0·002	0·000-0·001	0·000-0·001	0·000-0·001	0·000-0·004	0·001-0·035
	Median (IQR)	0·00 (0·00-0·01)	0·00 (0·00-0·01)	0·00 (0·00-0·00)	0·00 (0·00-0·00)	0·00 (0·00-0·00)	0·00 (0·00-0·00)	0·00 (0·00-0·01)	0·00 (0·00-0·01)	0·00 (0·00-0·00)	0·00 (0·00-0·00)	0·00 (0·00-0·00)	0·00 (0·00-0·00)	0·00 (0·00-0·01)	0·01 (0·00-0·01)
	GMFR referencing Day 1		0·8		0·7		1·2		1·1		0·7		0·8		17·5
	95% CI		0·1-10·7		0·1-5·9		0·5-2·7		0·0-30·3		0·1-5·7		0·1-7·3		0·8-382·9
IL5+ IL13+															
WT RBD	GMT	0·000	0·000	0·000	0·000	0·000	0·000	0·000	0·000	0·000	0·000	0·000	0·000	0·000	0·000
	95% CI	0·000-0·000	0·000-0·000	0·000-0·000	0·000-0·000	0·000-0·000	0·000-0·000	0·000-0·000	0·000-0·000	0·000-0·000	0·000-0·000	0·000-0·000	0·000-0·000	0·000-0·000	0·000-0·000
	Median (IQR)	0·00 (0·00-0·00)	0·00 (0·00-0·00)	0·00 (0·00-0·00)	0·00 (0·00-0·00)	0·00 (0·00-0·00)	0·00 (0·00-0·00)	0·00 (0·00-0·00)	0·00 (0·00-0·00)	0·00 (0·00-0·00)	0·00 (0·00-0·00)	0·00 (0·00-0·00)	0·00 (0·00-0·00)	0·00 (0·00-0·00)	0·00 (0·00-0·00)
	GMFR referencing Day 1		1·2		1·0		1·0		1·0		1·0		0·5		1·0
	95% CI		0·2-6·2		1·0-1·0		1·0-1·0		1·0-1·0		1·0-1·0		0·1-2·6		1·0-1·0
Beta RBD	GMT	0·000	0·000	0·000	0·000	0·000	0·000	0·000	0·000	0·000	0·000	0·000	0·000	0·000	0·000
	95% CI	0·000-0·000	0·000-0·000	0·000-0·000	0·000-0·000	0·000-0·000	0·000-0·000	0·000-0·000	0·000-0·000	0·000-0·000	0·000-0·000	0·000-0·000	0·000-0·000	0·000-0·000	0·000-0·000
	Median (IQR)	0·00 (0·00-0·00)	0·00 (0·00-0·00)	0·00 (0·00-0·00)	0·00 (0·00-0·00)	0·00 (0·00-0·00)	0·00 (0·00-0·00)	0·00 (0·00-0·00)	0·00 (0·00-0·00)	0·00 (0·00-0·00)	0·00 (0·00-0·00)	0·00 (0·00-0·00)	0·00 (0·00-0·00)	0·00 (0·00-0·00)	0·00 (0·00-0·00)
	GMFR referencing Day 1		1·0		0·7		1·0		1·0		1·0		1·0		1·0
	95% CI		0·3-3·5		0·4-1·5		1·0-1·0		1·0-1·0		1·0-1·0		1·0-1·0		1·0-1·0
WT Spike	GMT	0·000	0·000	0·000	0·000	0·000	0·000	0·000	0·000	0·000	0·000	0·000	0·000	0·000	0·000
	95% CI	0·000-0·000	0·000-0·000	0·000-0·000	0·000-0·000	0·000-0·000	0·000-0·000	0·000-0·000	0·000-0·000	0·000-0·000	0·000-0·000	0·000-0·000	0·000-0·000	0·000-0·000	0·000-0·000
	Median (IQR)	0·00 (0·00-0·00)	0·00 (0·00-0·00)	0·00 (0·00-0·00)	0·00 (0·00-0·00)	0·00 (0·00-0·00)	0·00 (0·00-0·00)	0·00 (0·00-0·00)	0·00 (0·00-0·00)	0·00 (0·00-0·00)	0·00 (0·00-0·00)	0·00 (0·00-0·00)	0·00 (0·00-0·00)	0·00 (0·00-0·00)	0·00 (0·00-0·00)
	GMFR referencing Day 1		1·0		0·8		0·6		1·7		0·8		1·0		1·0
	95% CI		1·0-1·0		0·4-1·4		0·2-2·1		0·5-5·7		0·4-1·4		1·0-1·0		0·2-6·1
Human IgG1 Fc Region	GMT	0·000	0·000	0·000	0·000	0·000	0·000	0·000	0·000	0·000	0·000	0·000	0·000	0·000	0·000
	95% CI	0·000-0·000	0·000-0·000	0·000-0·000	0·000-0·000	0·000-0·000	0·000-0·000	0·000-0·000	0·000-0·000	0·000-0·000	0·000-0·000	0·000-0·000	0·000-0·000	0·000-0·000	0·000-0·000
	Median (IQR)	0·00 (0·00-0·00)	0·00 (0·00-0·00)	0·00 (0·00-0·00)	0·00 (0·00-0·00)	0·00 (0·00-0·00)	0·00 (0·00-0·00)	0·00 (0·00-0·00)	0·00 (0·00-0·00)	0·00 (0·00-0·00)	0·00 (0·00-0·00)	0·00 (0·00-0·00)	0·00 (0·00-0·00)	0·00 (0·00-0·00)	0·00 (0·00-0·00)
	GMFR referencing Day 1		1·0		1·0		2·0		1·0		0·7		1·0		1·8
	95% CI		1·0-1·0		1·0-1·0		0·4-9·7		1·0-1·0		0·4-1·4		1·0-1·0		0·4-7·2
IFNg+ MIP1b+															
WT RBD	GMT	0·000	0·000	0·000	0·000	0·000	0·000	0·000	0·000	0·000	0·000	0·000	0·000	0·000	0·000
	95% CI	0·000-0·000	0·000-0·000	0·000-0·000	0·000-0·000	0·000-0·000	0·000-0·000	0·000-0·000	0·000-0·000	0·000-0·000	0·000-0·000	0·000-0·000	0·000-0·000	0·000-0·000	0·000-0·000
	Median (IQR)	0·00 (0·00-0·00)	0·00 (0·00-0·00)	0·00 (0·00-0·00)	0·00 (0·00-0·00)	0·00 (0·00-0·00)	0·00 (0·00-0·00)	0·00 (0·00-0·00)	0·00 (0·00-0·00)	0·00 (0·00-0·00)	0·00 (0·00-0·00)	0·00 (0·00-0·00)	0·00 (0·00-0·00)	0·00 (0·00-0·00)	0·00 (0·00-0·00)
	GMFR referencing Day 1		1·0		0·7		1·0		0·4		1·1		0·4		1·7
	95% CI		1·0-1·0		0·4-1·5		1·0-1·0		0·1-3·4		0·9-1·4		0·0-3·6		0·5-6·3
Beta RBD	GMT	0·000	0·000	0·000	0·000	0·000	0·000	0·000	0·000	0·000	0·000	0·000	0·000	0·000	0·000
	95% CI	0·000-0·000	0·000-0·000	0·000-0·000	0·000-0·000	0·000-0·000	0·000-0·000	0·000-0·000	0·000-0·000	0·000-0·000	0·000-0·000	0·000-0·000	0·000-0·000	0·000-0·000	0·000-0·000
	Median (IQR)	0·00 (0·00-0·00)	0·00 (0·00-0·00)	0·00 (0·00-0·00)	0·00 (0·00-0·00)	0·00 (0·00-0·00)	0·00 (0·00-0·00)	0·00 (0·00-0·00)	0·00 (0·00-0·00)	0·00 (0·00-0·00)	0·00 (0·00-0·00)	0·00 (0·00-0·00)	0·00 (0·00-0·00)	0·00 (0·00-0·00)	0·00 (0·00-0·00)
	GMFR referencing Day 1		1·0		2·0		1·0		1·3		0·4		0·4		1·0
	95% CI		1·0-1·0		0·7-5·4		1·0-1·0		0·7-2·6		0·1-1·5		0·1-3·2		1·0-1·0
WT Spike	GMT	0·014	0·013	0·005	0·003	0·006	0·007	0·003	0·002	0·008	0·026	0·004	0·035	0·003	0·035
	95% CI	0·003-0·055	0·002-0·104	0·001-0·024	0·000-0·024	0·000-0·156	0·000-0·228	0·000-0·057	0·000-0·041	0·001-0·050	0·004-0·152	0·000-0·080	0·002-0·626	0·000-0·032	0·002-0·717
	Median (IQR)	0·01 (0·00-0·03)	0·02 (0·01-0·09)	0·02 (0·00-0·04)	0·02 (0·00-0·05)	0·04 (0·01-0·07)	0·06 (0·01-0·10)	0·01 (0·00-0·03)	0·01 (0·00-0·03)	0·02 (0·01-0·05)	0·05 (0·02-0·12)	0·01 (0·00-0·04)	0·10 (0·03-0·24)	0·01 (0·00-0·02)	0·10 (0·02-0·42)
	GMFR referencing Day 1		0·9		0·7		1·3		0·8		3·3		9·8		13·2
	95% CI		0·2-4·0		0·2-2·7		0·0-37·2		0·4-1·4		0·6-18·9		0·1-873·3		0·5-321·0
Human IgG1 Fc Region	GMT	0·000	0·000	0·000	0·000	0·000	0·000	0·000	0·000	0·000	0·000	0·000	0·000	0·000	0·000
	95% CI	0·000-0·000	0·000-0·000	0·000-0·000	0·000-0·000	0·000-0·000	0·000-0·000	0·000-0·000	0·000-0·000	0·000-0·000	0·000-0·000	0·000-0·001	0·000-0·000	0·000-0·000	0·000-0·000
	Median (IQR)	0·00 (0·00-0·00)	0·00 (0·00-0·00)	0·00 (0·00-0·00)	0·00 (0·00-0·00)	0·00 (0·00-0·00)	0·00 (0·00-0·00)	0·00 (0·00-0·00)	0·00 (0·00-0·00)	0·00 (0·00-0·00)	0·00 (0·00-0·00)	0·00 (0·00-0·00)	0·00 (0·00-0·00)	0·00 (0·00-0·00)	0·00 (0·00-0·00)
	GMFR referencing Day 1		0·7		1·0		1·0		0·5		1·4		0·2		1·7
	95% CI		0·3-1·6		0·3-3·5		1·0-1·0		0·1-2·7		0·7-2·7		0·0-2·4		0·5-6·0
TNFa+ MIP1b+															
WT RBD	GMT	0·000	0·000	0·000	0·000	0·000	0·000	0·000	0·000	0·000	0·000	0·000	0·000	0·000	0·000
	95% CI	0·000-0·000	0·000-0·000	0·000-0·000	0·000-0·000	0·000-0·000	0·000-0·000	0·000-0·000	0·000-0·000	0·000-0·000	0·000-0·000	0·000-0·000	0·000-0·000	0·000-0·000	0·000-0·000
	Median (IQR)	0·00 (0·00-0·00)	0·00 (0·00-0·00)	0·00 (0·00-0·00)	0·00 (0·00-0·00)	0·00 (0·00-0·00)	0·00 (0·00-0·00)	0·00 (0·00-0·00)	0·00 (0·00-0·00)	0·00 (0·00-0·00)	0·00 (0·00-0·00)	0·00 (0·00-0·00)	0·00 (0·00-0·00)	0·00 (0·00-0·00)	0·00 (0·00-0·00)
	GMFR referencing Day 1		0·7		0·8		1·0		1·0		2·0		3·3		0·6
	95% CI		0·3-1·6		0·4-1·4		1·0-1·0		1·0-1·0		0·7-5·5		0·5-20·9		0·2-2·0
Beta RBD	GMT	0·000	0·000	0·000	0·000	0·000	0·000	0·000	0·000	0·000	0·000	0·000	0·000	0·000	0·000
	95% CI	0·000-0·000	0·000-0·000	0·000-0·000	0·000-0·000	0·000-0·000	0·000-0·000	0·000-0·000	0·000-0·000	0·000-0·000	0·000-0·000	0·000-0·000	0·000-0·000	0·000-0·000	0·000-0·000
	Median (IQR)	0·00 (0·00-0·00)	0·00 (0·00-0·00)	0·00 (0·00-0·00)	0·00 (0·00-0·00)	0·00 (0·00-0·00)	0·00 (0·00-0·00)	0·00 (0·00-0·00)	0·00 (0·00-0·00)	0·00 (0·00-0·00)	0·00 (0·00-0·00)	0·00 (0·00-0·00)	0·00 (0·00-0·00)	0·00 (0·00-0·00)	0·00 (0·00-0·00)
	GMFR referencing Day 1		1·0		1·4		1·0		1·0		1·0		0·5		1·0
	95% CI		1·0-1·0		0·7-2·8		1·0-1·0		1·0-1·0		1·0-1·0		0·1-2·5		1·0-1·0
WT Spike	GMT	0·000	0·001	0·000	0·000	0·001	0·000	0·000	0·000	0·001	0·001	0·000	0·000	0·000	0·000
	95% CI	0·000-0·003	0·000-0·007	0·000-0·001	0·000-0·001	0·000-0·005	0·000-0·004	0·000-0·001	0·000-0·001	0·000-0·004	0·000-0·003	0·000-0·002	0·000-0·004	0·000-0·001	0·000-0·001
	Median (IQR)	0·00 (0·00-0·01)	0·00 (0·00-0·01)	0·00 (0·00-0·00)	0·00 (0·00-0·00)	0·00 (0·00-0·00)	0·00 (0·00-0·00)	0·00 (0·00-0·00)	0·00 (0·00-0·00)	0·00 (0·00-0·01)	0·00 (0·00-0·01)	0·00 (0·00-0·00)	0·00 (0·00-0·00)	0·00 (0·00-0·00)	0·00 (0·00-0·00)
	GMFR referencing Day 1		2·6		0·9		0·7		0·8		0·8		3·0		0·5
	95% CI		0·2-35·3		0·1-6·3		0·1-10·7		0·3-2·0		0·1-6·0		0·2-47·0		0·0-17·9
Human IgG1 Fc Region	GMT	0·000	0·000	0·000	0·000	0·000	0·000	0·000	0·000	0·000	0·000	0·000	0·000	0·000	0·000
	95% CI	0·000-0·000	0·000-0·000	0·000-0·000	0·000-0·000	0·000-0·000	0·000-0·000	0·000-0·000	0·000-0·000	0·000-0·000	0·000-0·000	0·000-0·000	0·000-0·000	0·000-0·000	0·000-0·000
	Median (IQR)	0·00 (0·00-0·00)	0·00 (0·00-0·00)	0·00 (0·00-0·00)	0·00 (0·00-0·00)	0·00 (0·00-0·00)	0·00 (0·00-0·00)	0·00 (0·00-0·00)	0·00 (0·00-0·00)	0·00 (0·00-0·00)	0·00 (0·00-0·00)	0·00 (0·00-0·00)	0·00 (0·00-0·00)	0·00 (0·00-0·00)	0·00 (0·00-0·00)
	GMFR referencing Day 1		1·0		1·0		1·0		1·0		1·4		1·0		1·0
	95% CI		1·0-1·0		1·0-1·0		1·0-1·0		1·0-1·0		0·7-2·8		1·0-1·0		1·0-1·0
IFNg+ TNFa+ MIP1b+															
WT RBD	GMT	0·000	0·000	0·000	0·000	0·000	0·000	0·000	0·000	0·000	0·000	0·000	0·000	0·000	0·000
	95% CI	0·000-0·000	0·000-0·000	0·000-0·000	0·000-0·000	0·000-0·000	0·000-0·000	0·000-0·000	0·000-0·000	0·000-0·000	0·000-0·000	0·000-0·000	0·000-0·000	0·000-0·000	0·000-0·000
	Median (IQR)	0·00 (0·00-0·00)	0·00 (0·00-0·00)	0·00 (0·00-0·00)	0·00 (0·00-0·00)	0·00 (0·00-0·00)	0·00 (0·00-0·00)	0·00 (0·00-0·00)	0·00 (0·00-0·00)	0·00 (0·00-0·00)	0·00 (0·00-0·00)	0·00 (0·00-0·00)	0·00 (0·00-0·00)	0·00 (0·00-0·00)	0·00 (0·00-0·00)
	GMFR referencing Day 1		1·0		1·0		1·0		1·0		1·4		1·1		1·0
	95% CI		1·0-1·0		1·0-1·0		1·0-1·0		1·0-1·0		0·7-3·0		0·1-9·3		1·0-1·0
Beta RBD	GMT	0·000	0·000	0·000	0·000	0·000	0·000	0·000	0·000	0·000	0·000	0·000	0·000	0·000	0·000
	95% CI	0·000-0·000	0·000-0·000	0·000-0·000	0·000-0·000	0·000-0·000	0·000-0·000	0·000-0·000	0·000-0·000	0·000-0·000	0·000-0·000	0·000-0·000	0·000-0·000	0·000-0·000	0·000-0·000
	Median (IQR)	0·00 (0·00-0·00)	0·00 (0·00-0·00)	0·00 (0·00-0·00)	0·00 (0·00-0·00)	0·00 (0·00-0·00)	0·00 (0·00-0·00)	0·00 (0·00-0·00)	0·00 (0·00-0·00)	0·00 (0·00-0·00)	0·00 (0·00-0·00)	0·00 (0·00-0·00)	0·00 (0·00-0·00)	0·00 (0·00-0·00)	0·00 (0·00-0·00)
	GMFR referencing Day 1		1·0		1·2		1·0		0·5		1·0		1·9		1·0
	95% CI		1·0-1·0		0·8-1·9		1·0-1·0		0·1-2·4		1·0-1·0		0·4-8·0		1·0-1·0
WT Spike	GMT	0·014	0·025	0·002	0·002	0·001	0·005	0·003	0·001	0·010	0·013	0·004	0·006	0·003	0·008
	95% CI	0·002-0·098	0·007-0·093	0·000-0·014	0·000-0·011	0·000-0·047	0·001-0·054	0·000-0·062	0·000-0·027	0·002-0·046	0·003-0·066	0·000-0·031	0·001-0·075	0·000-0·033	0·000-0·133
	Median (IQR)	0·02 (0·01-0·03)	0·02 (0·01-0·04)	0·01 (0·00-0·02)	0·01 (0·00-0·02)	0·01 (0·00-0·03)	0·01 (0·00-0·02)	0·01 (0·00-0·03)	0·00 (0·00-0·04)	0·02 (0·01-0·05)	0·03 (0·01-0·07)	0·00 (0·00-0·01)	0·01 (0·00-0·05)	0·00 (0·00-0·02)	0·04 (0·00-0·09)
	GMFR referencing Day 1		1·8		0·8		3·8		0·4		1·4		1·7		2·4
	95% CI		0·6-5·2		0·1-5·9		0·3-56·4		0·0-9·7		0·4-5·0		0·8-4·0		0·1-61·7
Human IgG1 Fc Region	GMT	0·000	0·000	0·000	0·000	0·000	0·000	0·000	0·000	0·000	0·000	0·000	0·000	0·000	0·000
	95% CI	0·000-0·000	0·000-0·000	0·000-0·000	0·000-0·000	0·000-0·000	0·000-0·000	0·000-0·000	0·000-0·000	0·000-0·000	0·000-0·000	0·000-0·000	0·000-0·000	0·000-0·000	0·000-0·000
	Median (IQR)	0·00 (0·00-0·00)	0·00 (0·00-0·00)	0·00 (0·00-0·00)	0·00 (0·00-0·00)	0·00 (0·00-0·00)	0·00 (0·00-0·00)	0·00 (0·00-0·00)	0·00 (0·00-0·00)	0·00 (0·00-0·00)	0·00 (0·00-0·00)	0·00 (0·00-0·00)	0·00 (0·00-0·00)	0·00 (0·00-0·00)	0·00 (0·00-0·00)
	GMFR referencing Day 1		1·0		1·0		1·0		1·0		1·0		1·8		1·0
	95% CI		1·0-1·0		1·0-1·0		1·0-1·0		1·0-1·0		1·0-1·0		0·4-7·8		1·0-1·0
IFNg+ MIP1b+ IL13+															
WT RBD	GMT	0·008	0·009	0·003	0·004	0·002	0·006	0·006	0·002	0·007	0·001	0·001	0·002	0·006	0·003
	95% CI	0·002-0·038	0·002-0·037	0·000-0·016	0·001-0·023	0·000-0·028	0·001-0·058	0·000-0·094	0·000-0·064	0·002-0·033	0·000-0·012	0·000-0·040	0·000-0·050	0·001-0·056	0·000-0·038
	Median (IQR)	0·02 (0·01-0·04)	0·02 (0·01-0·03)	0·01 (0·00-0·02)	0·01 (0·01-0·02)	0·01 (0·00-0·03)	0·02 (0·00-0·03)	0·03 (0·00-0·05)	0·03 (0·00-0·07)	0·02 (0·00-0·04)	0·02 (0·00-0·03)	0·02 (0·00-0·03)	0·02 (0·00-0·03)	0·02 (0·00-0·03)	0·01 (0·00-0·03)
	GMFR referencing Day 1		1·0		1·7		3·4		0·2		0·2		1·9		0·6
	95% CI		0·1-10·0		0·2-14·5		0·2-76·2		0·0-4·3		0·0-1·0		0·0-439·7		0·0-22·1
Beta RBD	GMT	0·012	0·008	0·003	0·004	0·003	0·002	0·005	0·008	0·002	0·003	0·006	0·006	0·006	0·004
	95% CI	0·005-0·027	0·002-0·032	0·001-0·013	0·001-0·018	0·000-0·064	0·000-0·031	0·000-0·069	0·001-0·113	0·000-0·013	0·000-0·021	0·001-0·069	0·001-0·061	0·001-0·034	0·000-0·082
	Median (IQR)	0·02 (0·01-0·03)	0·01 (0·01-0·02)	0·01 (0·00-0·02)	0·01 (0·00-0·02)	0·02 (0·01-0·03)	0·01 (0·00-0·02)	0·02 (0·00-0·05)	0·03 (0·00-0·07)	0·01 (0·00-0·02)	0·02 (0·00-0·04)	0·02 (0·00-0·05)	0·02 (0·01-0·02)	0·01 (0·00-0·02)	0·02 (0·01-0·03)
	GMFR referencing Day 1		0·6		1·3		0·5		1·7		1·5		1·0		0·6
	95% CI		0·1-3·0		0·2-10·5		0·0-16·3		0·2-14·5		0·2-13·9		0·0-37·5		0·0-14·7
WT Spike	GMT	0·000	0·000	0·000	0·000	0·000	0·000	0·000	0·000	0·000	0·000	0·000	0·001	0·000	0·000
	95% CI	0·000-0·002	0·000-0·001	0·000-0·000	0·000-0·001	0·000-0·001	0·000-0·002	0·000-0·001	0·000-0·007	0·000-0·001	0·000-0·001	0·000-0·008	0·000-0·019	0·000-0·000	0·000-0·003
	Median (IQR)	0·00 (0·00-0·01)	0·00 (0·00-0·00)	0·00 (0·00-0·00)	0·00 (0·00-0·01)	0·00 (0·00-0·00)	0·00 (0·00-0·00)	0·00 (0·00-0·00)	0·00 (0·00-0·01)	0·00 (0·00-0·00)	0·00 (0·00-0·01)	0·00 (0·00-0·02)	0·01 (0·00-0·01)	0·00 (0·00-0·00)	0·00 (0·00-0·01)
	GMFR referencing Day 1		0·6		2·8		1·5		7·0		1·1		4·2		5·0
	95% CI		0·0-10·6		0·1-55·8		0·0-51·7		0·4-122·0		0·1-17·2		0·1-342·9		0·1-175·7
Human IgG1 Fc Region	GMT	0·006	0·008	0·003	0·009	0·004	0·001	0·007	0·004	0·004	0·003	0·003	0·001	0·006	0·019
	95% CI	0·001-0·037	0·002-0·031	0·001-0·014	0·003-0·027	0·000-0·039	0·000-0·021	0·000-0·110	0·000-0·115	0·001-0·023	0·000-0·016	0·000-0·056	0·000-0·034	0·001-0·063	0·008-0·044
	Median (IQR)	0·02 (0·01-0·03)	0·01 (0·01-0·02)	0·01 (0·00-0·02)	0·02 (0·01-0·03)	0·01 (0·00-0·03)	0·01 (0·00-0·01)	0·04 (0·00-0·07)	0·04 (0·00-0·08)	0·02 (0·00-0·03)	0·01 (0·00-0·03)	0·01 (0·00-0·03)	0·01 (0·00-0·03)	0·01 (0·00-0·03)	0·02 (0·01-0·04)
	GMFR referencing Day 1		1·4		3·0		0·2		0·6		0·6		0·4		3·1
	95% CI		0·1-17·9		0·8-11·7		0·0-3·5		0·1-6·1		0·1-4·4		0·0-62·1		0·3-35·7

CI denotes confidence interval, GMT geometric mean titre, IQR interquartile range (25th to 75th percentile), GMFR geometric mean fold rise, N number of subjects in the Immunogenicity Set within each visit with non-missing data.
The Immunogenicity Set consists of all enrolled participants who were randomised and received one dose of study vaccine or placebo and provided at least one valid immunogenicity response measured at least 28 days after one dose of study vaccine or placebo.
The 95% CI for GMT and GMFR were calculated based on the t-distribution on the log base 10 transformed values, then back transformed (power of 10) on the original score.
To allow for log-transformation, a value of 0.00001 was added to all values of percentage of total cytokine+ secreting cells in the CD8 population.

Supplementary Table 14: Anti-Fc antibody (binding to human IgG Fc)

		Placebo		Protein-RBD						mRNA-RBD					
				5µg		15µg		45µg		10µg		20µg		50µg	
	N	Day 1	Day 29	Day 1	Day 29	Day 1	Day 29	Day 1	Day 29	Day 1	Day 29	Day 1	Day 29	Day 1	Day 29
		**12**	**12**	**16**	**16**	**8**	**8**	**8**	**8**	**16**	**16**	**8**	**8**	**8**	**8**
IgM binding to human IgG Fc	GM	0·30	0·32	0·31	0·30	0·37	0·39	0·35	0·36	0·33	0·34	0·37	0·34	0·28	0·28
	95% CI	0·21-0·44	0·21-0·49	0·23-0·41	0·23-0·38	0·30-0·45	0·34-0·45	0·15-0·82	0·16-0·84	0·25-0·43	0·25-0·45	0·26-0·52	0·25-0·47	0·21-0·39	0·21-0·39
	Median (IQR)	0·3 (0·2-0·4)	0·3 (0·2-0·4)	0·3 (0·2-0·4)	0·3 (0·2-0·4)	0·4 (0·3-0·4)	0·4 (0·4-0·4)	0·3 (0·2-0·4)	0·3 (0·2-0·4)	0·3 (0·2-0·5)	0·3 (0·2-0·5)	0·3 (0·3-0·5)	0·3 (0·3-0·4)	0·3 (0·2-0·3)	0·3 (0·2-0·3)
IgG binding to human IgG Fc	GM	0·28	0·29	0·30	0·30	0·46	0·43	0·20	0·20	0·32	0·33	0·39	0·40	0·19	0·19
	95% CI	0·23-0·34	0·24-0·36	0·27-0·33	0·27-0·33	0·41-0·51	0·37-0·51	0·18-0·22	0·19-0·22	0·28-0·37	0·28-0·38	0·33-0·46	0·34-0·46	0·15-0·23	0·15-0·24
	Median (IQR)	0·3 (0·2-0·3)	0·3 (0·2-0·4)	0·3 (0·3-0·3)	0·3 (0·3-0·3)	0·5 (0·4-0·5)	0·5 (0·4-0·5)	0·2 (0·2-0·2)	0·2 (0·2-0·2)	0·3 (0·3-0·4)	0·4 (0·3-0·4)	0·4 (0·3-0·5)	0·4 (0·3-0·5)	0·2 (0·1-0·2)	0·2 (0·2-0·2)
IgA binding to human IgG Fc	GM	0·16	0·16	0·17	0·17	0·18	0·18	0·16	0·15	0·15	0·15	0·16	0·18	0·15	0·15
	95% CI	0·15-0·17	0·14-0·17	0·15-0·20	0·15-0·19	0·16-0·20	0·15-0·20	0·12-0·21	0·12-0·20	0·14-0·16	0·14-0·17	0·15-0·18	0·16-0·19	0·14-0·17	0·14-0·17
	Median (IQR)	0·2 (0·1-0·2)	0·2 (0·1-0·2)	0·2 (0·2-0·2)	0·2 (0·1-0·2)	0·2 (0·2-0·2)	0·2 (0·2-0·2)	0·1 (0·1-0·1)	0·1 (0·1-0·2)	0·1 (0·1-0·2)	0·1 (0·1-0·2)	0·2 (0·2-0·2)	0·2 (0·2-0·2)	0·2 (0·1-0·2)	0·1 (0·1-0·2)

CI denotes confidence interval, GM geometric mean, IQR interquartile range (25th to 75th percentile), N number of subjects in the Immunogenicity Set within each visit with non-missing data.

The Immunogenicity Set consists of all enrolled participants who were randomised and received one dose of study vaccine or placebo and provided at least one valid immunogenicity response measured at least 28 days after one dose of study vaccine or placebo.

The 95% CI for GM was calculated based on the t-distribution on the log base 10 transformed values, then back transformed (power of 10) on the original score.

Supplementary Table 15: SARS-CoV-2+ participants

Pt	Vaccination Date	Sample Collection Date	Analysis Date	VOC Status
1	13-Apr-22	17-May-22	22-May-22	Omicron (BA.2-like)
2	14-Apr-22	28-May-22	3-Jun-22	Omicron (BA.2 -like)
3	24-May-22	9-Jun-22	21-Jun-22	Omicron (BA.2 -like)
4	5-Jul-22	14-Jul-22	20-Jul-22	Omicron (BA.2-like)
5	6-Jun-22	28-Jul-22	2-Aug-22	Omicron (Unassigned)
6	30-Jun-22	13-Oct-22	18-Oct-22	Omicron (BA.5 -like)
7	13-Oct-22	23-Nov-22	2-Dec-22	Unable to be determined
8	20-Oct-22	5-Dec-22	12-Dec-22	Unable to be determined (RNA not detected)
9	11-Jul-22	7-Dec-22	17-Dec-22	Omicron (BA.2-like)
10	8-Jun-22	12-Dec-22	17-Dec-22	Omicron (BA.2-like)
11	18-Aug-22	12-Dec-22	17-Dec-22	Unable to be determined
12	15-Jun-22	13-Dec-22	17-Dec-22	Omicron (BA.5 -like)
13	23-Jun-22	19-Dec-22	3-Jan-23	Omicron (BA.5 -like)
14	27-Jun-22	19-Dec-22	3-Jan-23	Omicron (BA.5 -like)

15 Participant xxxxx was vaccinated on 20 May 22 and tested positive for Covid-19 by RAT on 11 Jul 22 but not inform study personnel of their positive result until their subsequent study visit on 15 Aug 22.

16 Participant xxxxx vaccinated on 7 Sep 22 and tested positive for Covid-19 by RAT on 14 Sep 22 but declined to provide a sample for genotyping.

17 Participant xxxxx vaccinated on 19 Oct 22 and tested positive for Covid-19 by RAT on 3 Jan 23 but did not inform study personne of their positive result collected until their V6 attendance (18 Jan 23). A sample for genotyping was not obtained.

Supplementary Figure 1: Microneutralisation d1/d29 by prior COVID-19, individual plots

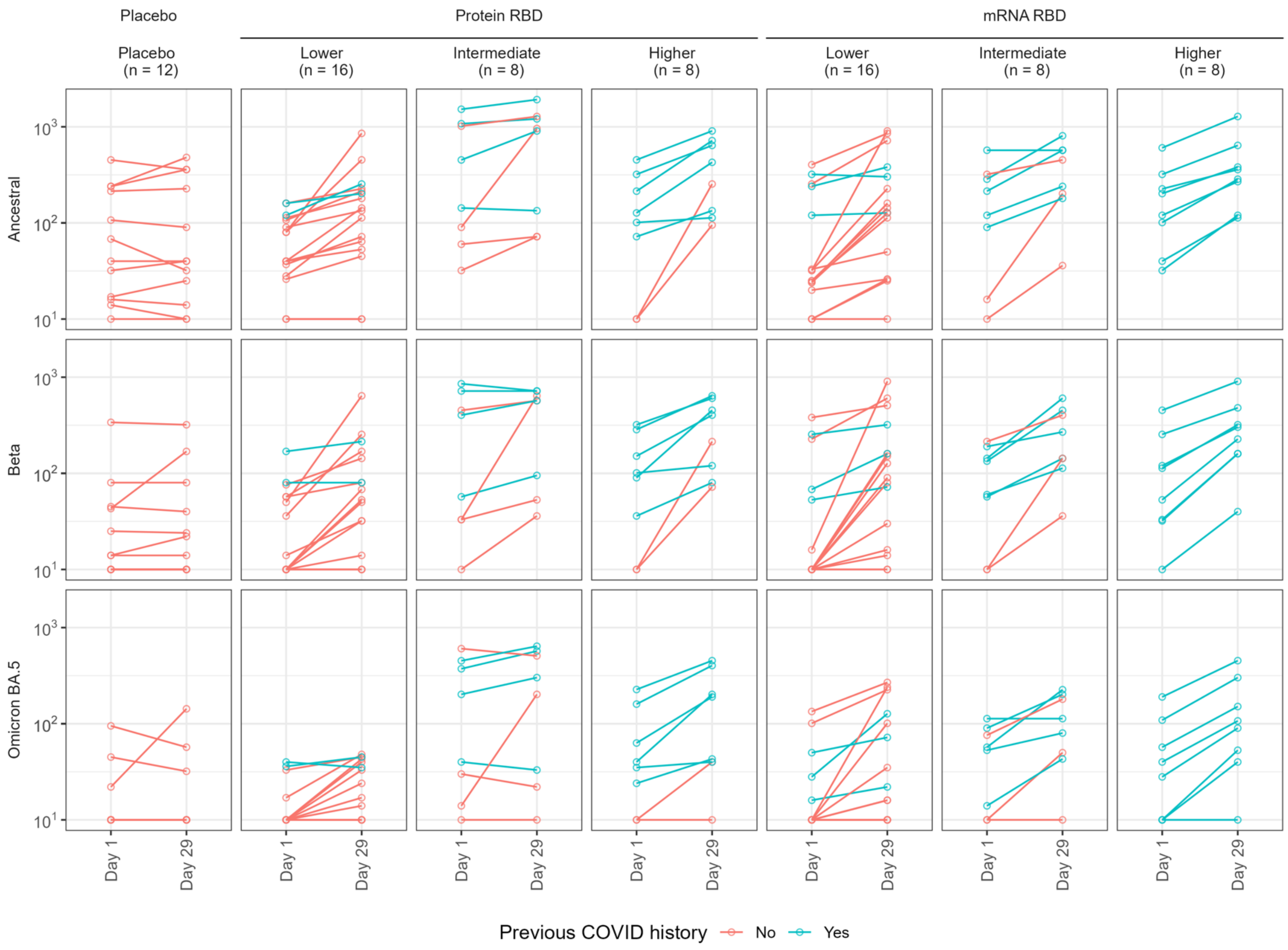

Supplementary Figure 2: ELISA and Microneutralisation geometric mean fold rises (GMFR). (WT/Vic01 = ancestral strain)

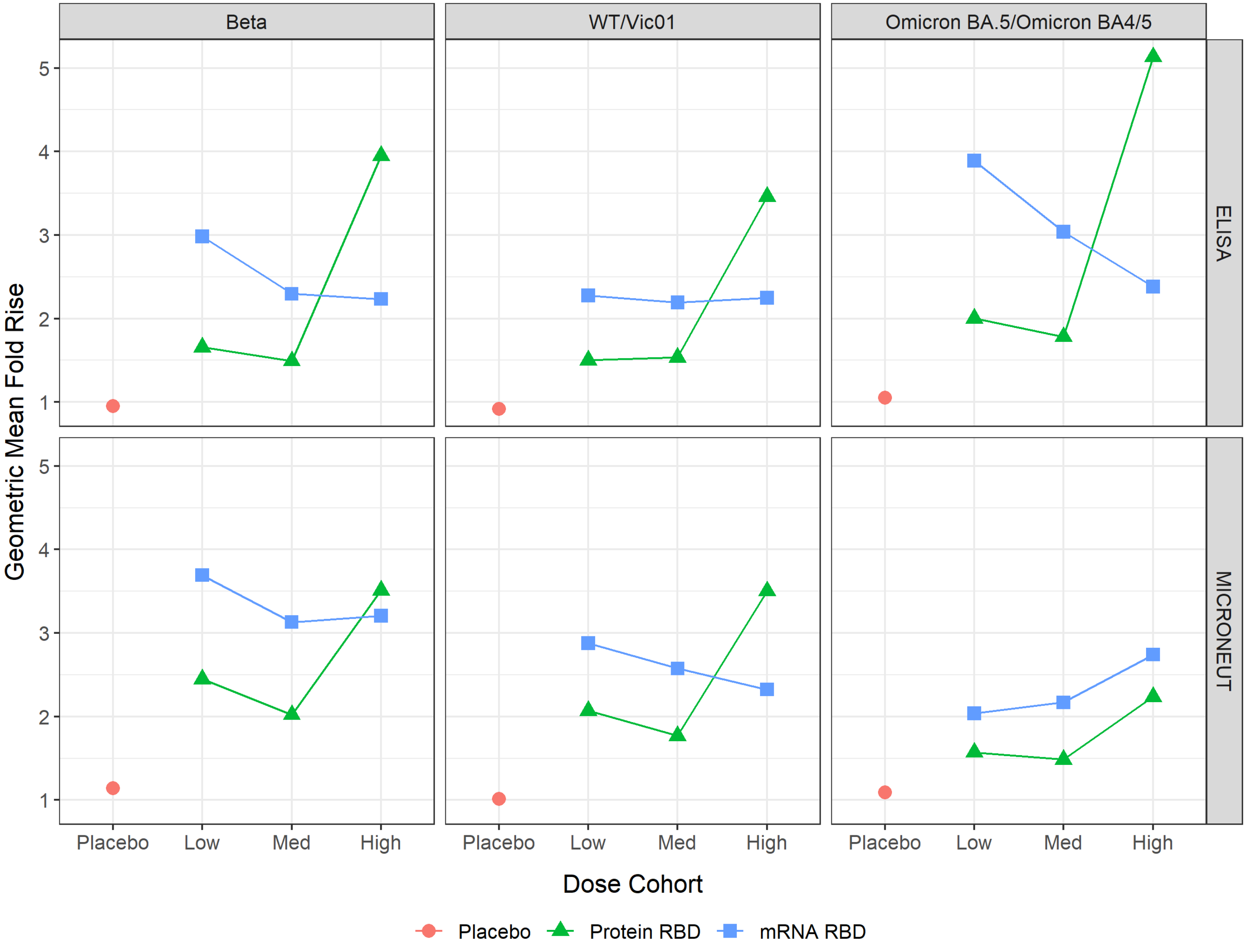

Supplementary Figure 3: sVNT (Method B) geometric mean fold rises (GMFR). (WT = ancestral strain)

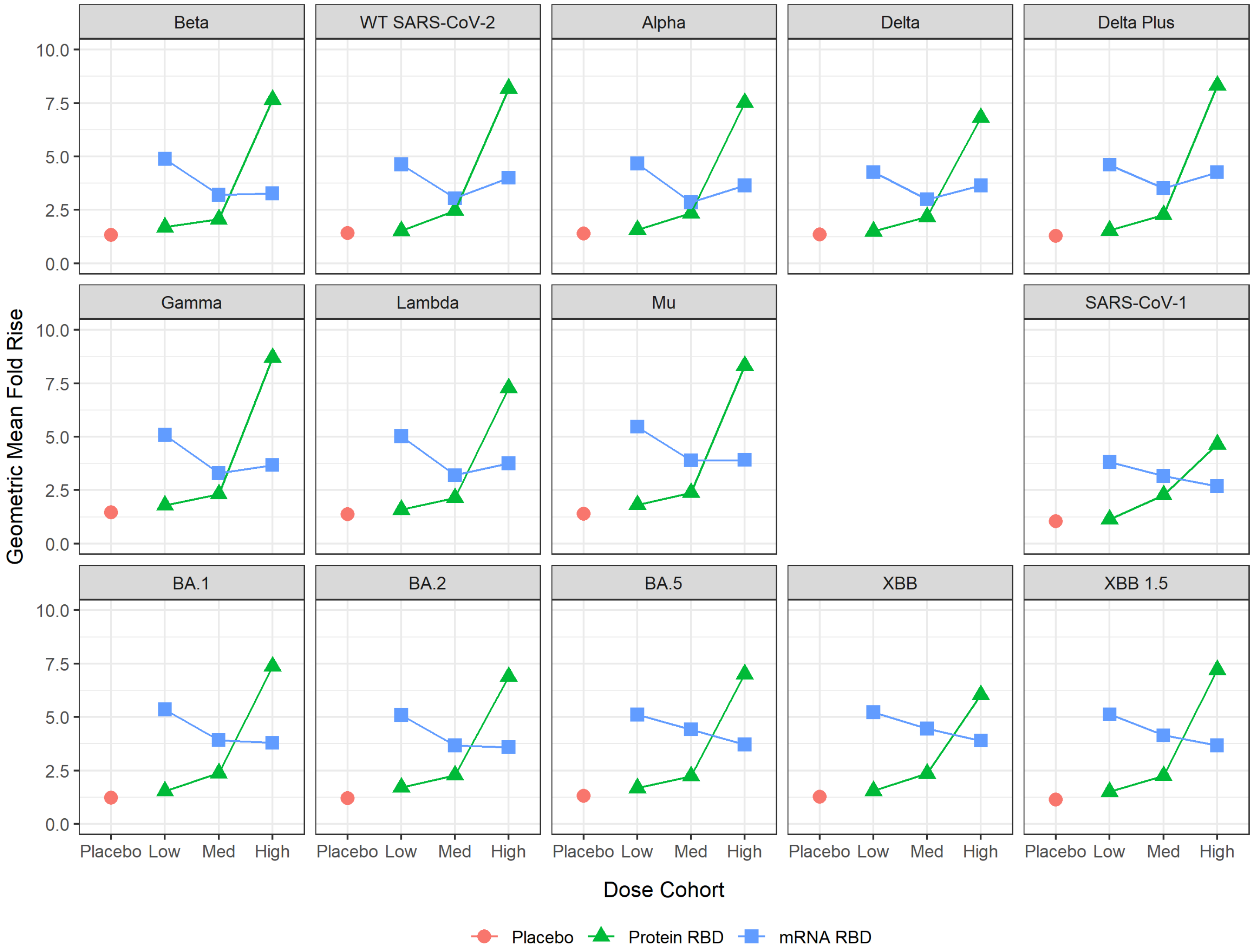

Supplementary Figure 4: pVNT geometric mean fold rises (GMFR).

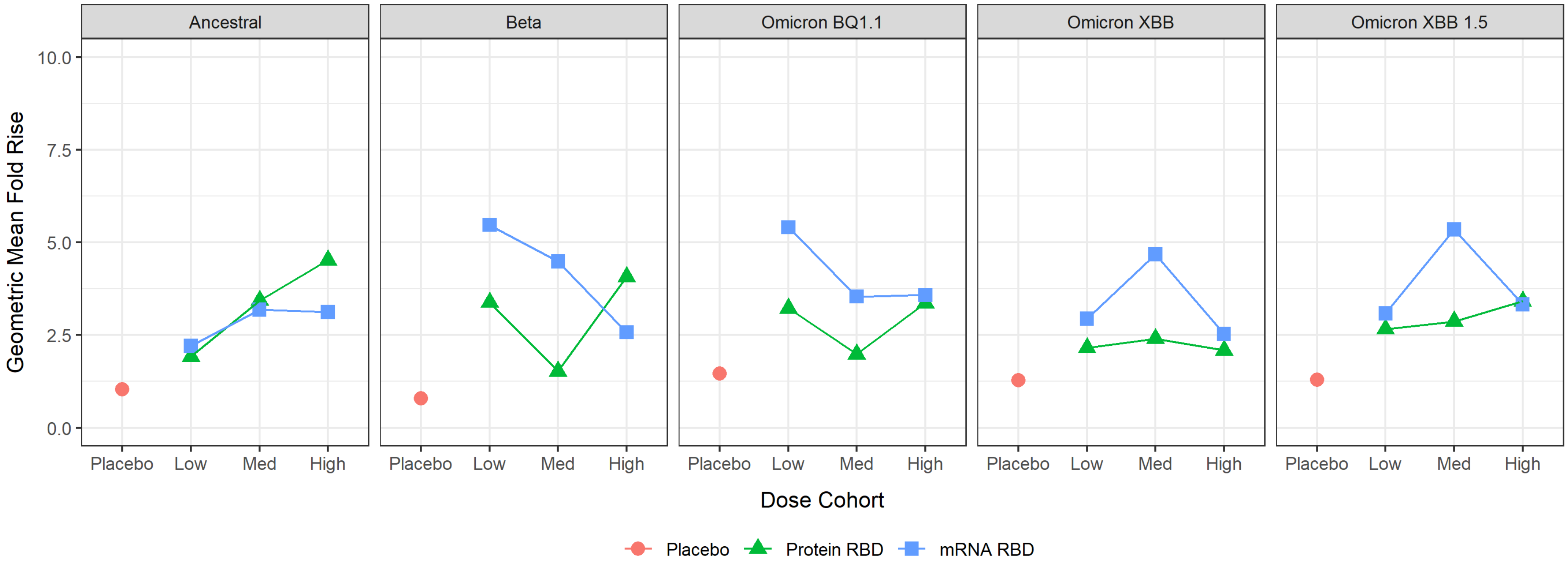

Supplementary Figure 5: pVNT individual plots, d1/d29 (Linfa Wang lab). Bars are geometric means and 95% confidence intervals.

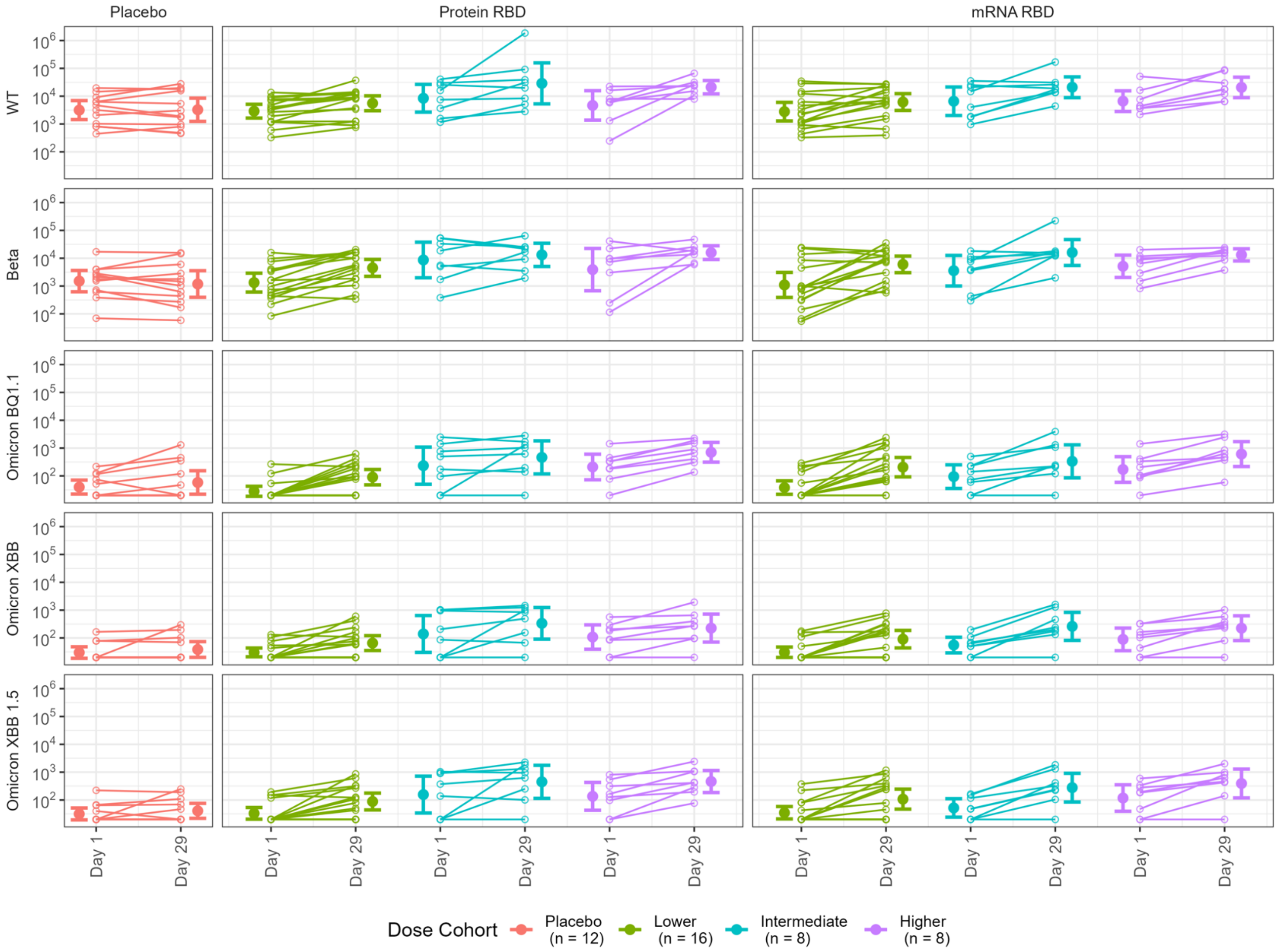

Supplementary Figure 6: sVNT individual plots (Part 1), d1/d29 (Method B, Linfa Wang lab). Bars are geometric means and 95% confidence intervals.

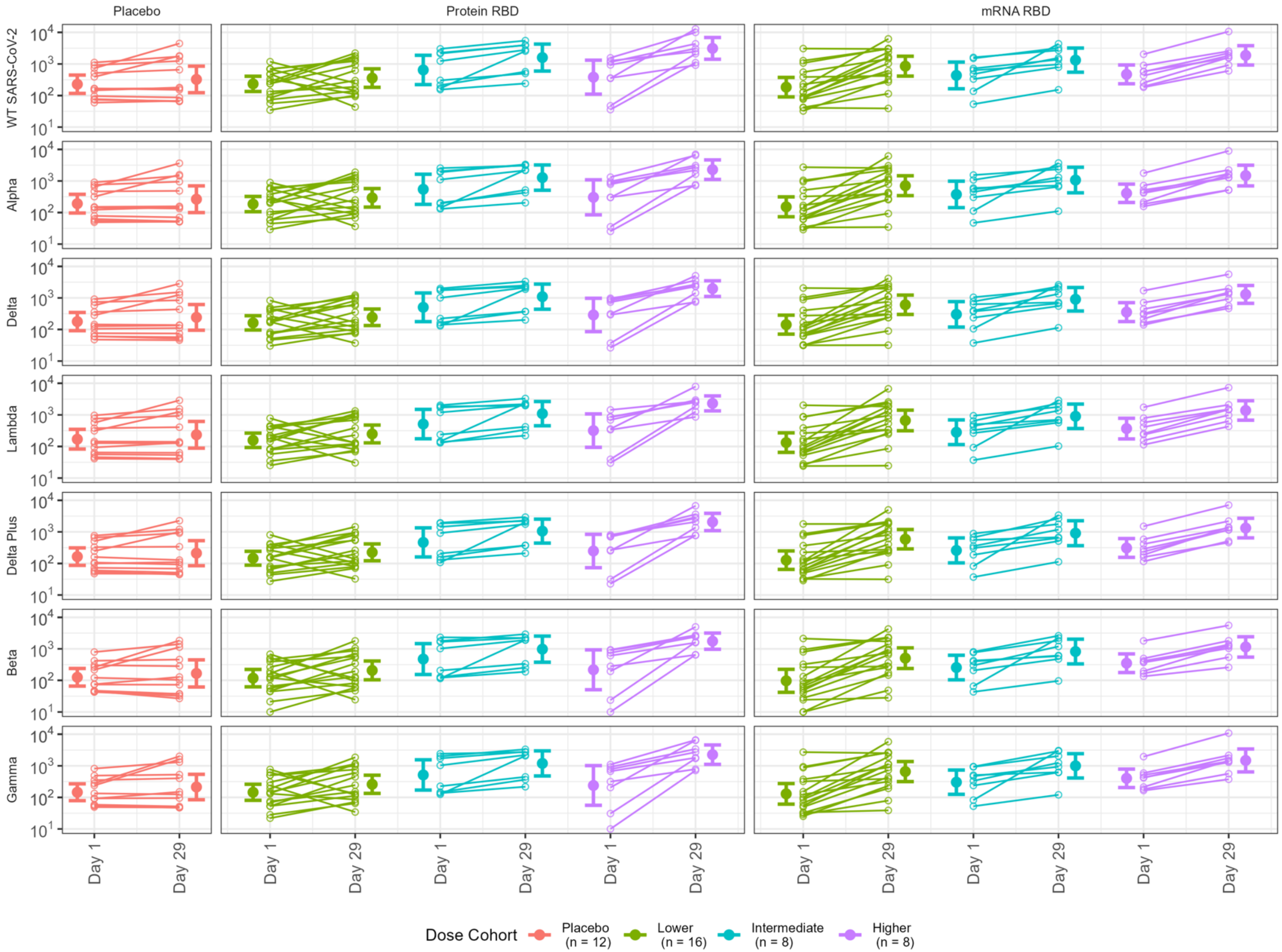

Supplementary Figure 7: sVNT individual plots (Part 2), d1/d29 (Method B, Linfa Wang lab). Bars are geometric means and 95% confidence intervals.

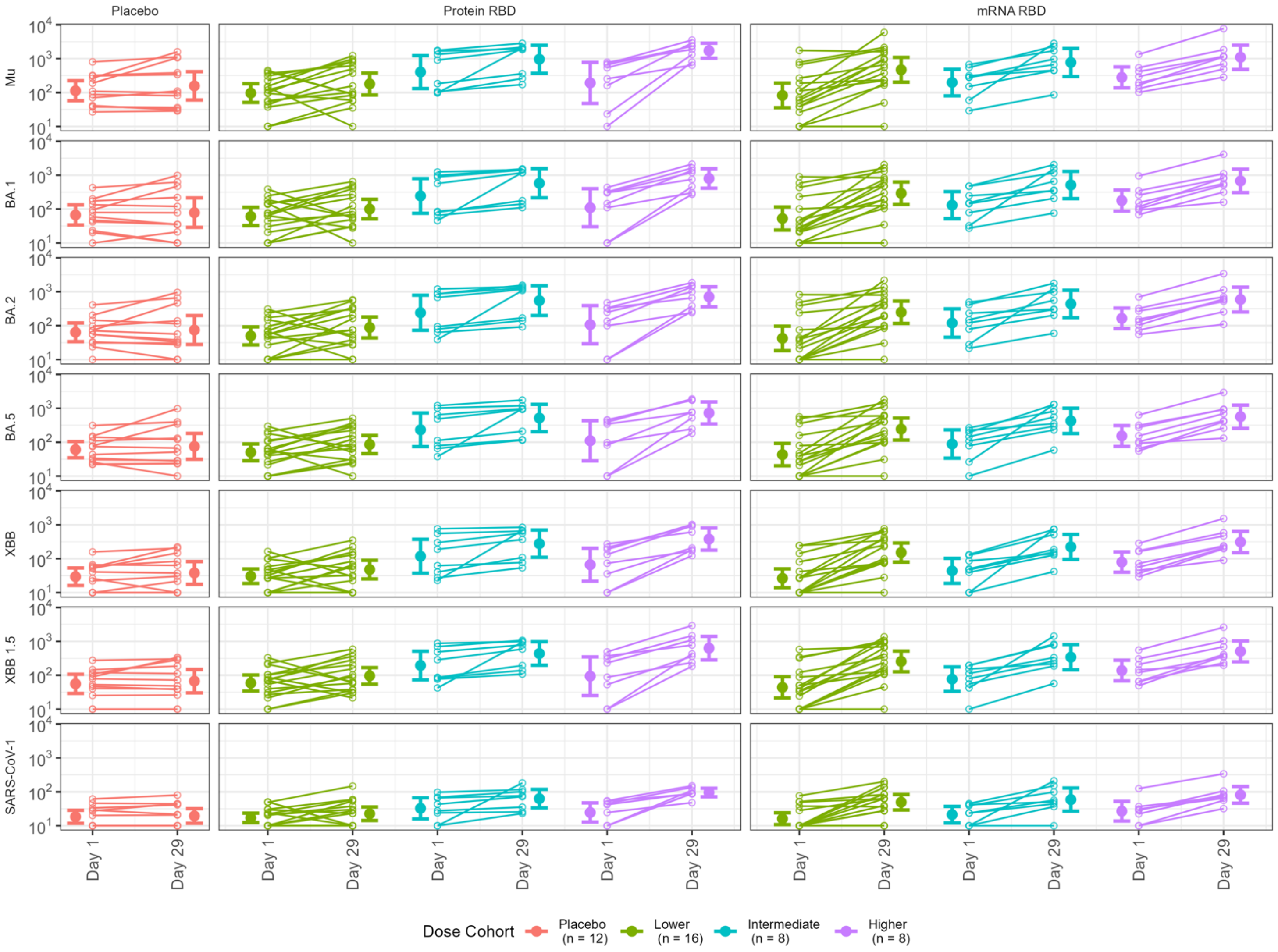

Supplementary Methods

Enzyme-linked immunosorbent assay (ELISA) for measurement of RBD-specific antibody responses RBD-specific IgG antibody responses in day 1 and day 29 sera were investigated by ELISA using ancestral RBD, beta RBD or omicron BA.5 RBD. Flat bottom 96 well maxisorp plates (Thermo Fisher Scientific) were coated with 50µl/well of RBD monomer at a concentration of 2 µg/ml in Dulbecco's phosphate buffered saline (DPBS; Gibco Life Technologies). Plates were incubated overnight at 4°C in a humidified atmosphere. Unbound antibody was removed, and wells were blocked with 100µl/well of 1% bovine serum albumin (BSA fraction V, Invitrogen Corporation, Gibco) in PBS for more than 1 hour before washing 2 times with PBS containing 0.05% v/v Tween-20 (PBST). Serial dilutions of human sera were added to wells and left to incubate overnight in a humidified atmosphere at room temperature. After washing, bound Ab was detected using horseradish peroxidase (HRP)-conjugated goat anti-human IgG Ab (Jackson ImmunoResearch Laboratories Inc, USA). The detection antibody was incubated for 1 hour at room temperature in a humidified atmosphere and the plates then washed four times with PBST. 100µl of tetramethylbenzidine substrate (TMB, BD Biosciences) was then added to each well and the reaction was stopped after 5 minutes by the addition of 100 µl/well of 1M orthophosphoric acid (BDH Chemicals, Australia). A Labsystems Multiskan microplate reader (Labsystems, Finland) was used to measure the optical density (OD) of each well at wavelengths of 450 nm and 540 nm. The titres of Ab are expressed as the reciprocal of the highest dilution of serum required to achieve an OD of 0.3 which represents at least five times the background level of binding.

In vitro microneutralisation test (mNT) An in vitro micro-neutralisation assay measured the level of SARS-CoV-2-specific nAb in day 1 and day 29 serum samples. SARS-CoV-2 isolates used in the mNT were propagated in VerohSLAM or Calu-3 cell cultures and stored at -80°C. Flat-bottom 96-well plates were seeded with Vero E6-TMPRSS2 cells (Cell Bank Australia, code JCRB1819) at 1.3 x 10^4 cells/well, two days before assay. Serial 2-fold dilutions of sera heat-inactivated at 56°C for 30 min were incubated with 100 TCID50 (50% tissue culture infectious dose) of the ancestral SARS-CoV-2 strain VIC01 (SARS-CoV-2/Australia/VIC01/2020, GenBank accession number: MT007544.1 or GISAID accession number: EPI_ISL_406844); beta variant (SARS-CoV-2/Australia/QLD1520/2020, GISAID accession number: EPI_ISL_968081); or omicron BA.5 (SARS-CoV-2/Australia/NSW/RPAH-1933/2023 (B.1.1.529.1), GISAID accession number: EPI_ISL_6814922), for 1 hour and residual virus infectivity was assessed in quadruplicate wells of Vero E6-TMPRSS2 cells. Viruses were provided by Dr Julian Druce, VIDRL, Doherty Institute, Australia. Plates were incubated at 37°C and viral CPE was read on day 5. The dilution of serum that inhibited CPE in 50% of the wells (ID50) was calculated by the Reed-Muench formula[1]. Limit of detection (LoD) based on lowest sera dilution tested is indicated in figures. Samples that lacked neutralising activity at this dose were assigned a value 50% lower to visually distinguish them from samples that displayed neutralising activity at the LoD.

RBD-ACE2 multiplex sVNT To investigate neutralising activity against multiple SARS-CoV-2 variants at once, Multiplex surrogate virus neutralisation tests (sVNT) were performed by two different laboratories using either Method A or B as outlined below.

RBD-ACE2 multiplex sVNT (Method A) RBD proteins from SARS-CoV-2 variants (Sino Biological) were coupled to magnetic multiplex beads as previously described[2]. A cocktail of these RBD-variant coupled beads was incubated in black polystyrene 384-well microplates (Greiner bio-one) with serial dilutions of day 1, day 8 and day 29 sera. Samples and controls were incubated overnight at 4°C with agitation (650 rpm), before addition of biotinylated human ACE2 (produced in house). After further 1hr incubation wells were washed with PBST. To detect ACE2 binding, Streptavidin-Phycoerythrin (Thermo Fisher Scientific) diluted to 4µg/ml was added to the wells and incubated for 1 hour at room temperature. The beads were again washed with PBST and resuspended in sheath fluid (Luminex). After 10 minutes agitation, the plate was loaded onto a Luminex INTELLIFLEX reader, high sensitivity mode. The binding of phycoerythrin-labelled ACE2 was reported as MFI (Median Fluorescence Intensity). Maximal ACE2 binding MFI was determined by the mean (quadruplicate) of ACE2 only (no inhibitor) controls. Results were calculated as half-maximal inhibitory dilution (ID50).

RBD-ACE2 multiplex sVNT (Method B) Biotinylated RBD proteins from SARS-CoV-2 variants were custom-made by Genscript, except for omicron BA.5, XBB and XBB1.5 which were produced in-house using Expi293T expression system according to manufacturer's instructions. RBD proteins were purified using Ni Sepharose (GE Healthcare) and desalted using Amicon Ultra-4, 10 KDa cut-off (Merck). Enzymatic biotinylation of AviTag was performed using BirA protein-biotin ligase kit (Avidity) according to the manufacturer's instructions. Excessive biotin was removed by Amicon Ultra-4, 10 MW (Merck). Protein concentration was determined by Nanodrop (Thermo Scientific).

Multiplex sVNTs were established as previously described[3,4]. Briefly, AviTag-biotinylated RBD proteins were coated on MagPlex-Avidin microspheres (Luminex) at 5 µg $million^{-1}$ beads. RBD-coated beads (600 per antigen) were pre-incubated with day 1 and day 29 sera at final dilutions of 1:20, 1:80, 1:320 and 1:1,280 for 60 min at 24 °C with agitation, followed by addition of 50 µl R-phycoerythrin-conjugated human ACE2 (2 µg ml^{-1}; Genscript) and incubation for an additional 1h at 24°C with agitation. After two washes with 1% BSA in PBS, the final readings were acquired using the MAGPIX system (Luminex) following the manufacturer's instructions. Results were calculated as half-maximal inhibitory dilution (ID50).

Pseudovirus neutralisation test (pVNT) Ancestral SARS-CoV-2, BQ1.1, XBB and XBB.1.5 full-length spike-pseudotyped viruses were produced and packaged as previously described[4]. In brief, HEK293T cells were transfected with 20 µg of pCAGGS spike plasmid using FuGene6 (Promega). At 24 h post transfection, cells were incubated with VSVΔG luc seed virus (at a multiplicity of infection of 5) for 2 h. Following two PBS washes, infected cells were replenished with complete growth media supplemented with 1:5000 diluted anti-VSV-G mAb (Clone 8GF11, Kerafast). At 24 h post infection, pseudoviruses were collected by centrifugation at 2,000g for 5 min. For pVNT assay, 3 million relative light units of pseudoviruses were pre-incubated with 6-fold serial-diluted day 1 and day 29 sera in a final volume of 50 µl for 1 h at 37°C, followed by infection of ACE2-stably-expressing A549 cells. At 20–24 h post infection, an equal volume of ONE-Glo luciferase substrate (Promega) was added and the luminescence signal was measured using the Cytation 5 microplate reader (BioTek) with Gen5 software version 3.10.

T-cell studies: 24-hr stimulation with RBD and Spike overlapping peptide pools. Thawed PBMCs on day 1, 29 and 91 were plated into a 96-well plate at 1e6 PBMCs/well. For AIM assay, cells were stimulated in complete-RPMI with 10μg/ml SARS-CoV-2 Spike peptide pool (181 peptides, 0.06μg/ml per peptide; BEI Resources, NIAID, NIH, SARS-Related Coronavirus 2 Spike (S) Glycoprotein, NR-52402), *other peptide pools*, or DMSO (0.1%; Sigma), as a negative control, and cultured at 37 ºC/5% CO_2 for 24 hours. Cells were washed and stained with CXCR5-BV421 (562747; BD Biosciences), CD3-BV510 (317332; BioLegend), CD8-BV605 (564116; BD Biosciences), CD4-BV650 (563875; BD Biosciences), CD25-BV711 (563159; BD Biosciences), CXCR3-BV786 (353738; BD Biosciences), CD137-APC (309810; BioLegend), CD27-AF700 (560611; BD Biosciences), CD14/CD19-APC-H7 (560180/560252; BD Biosciences), Live/Dead NIR (L34976; Invitrogen), CD69-PerCPCy5.5 (310925; BioLegend), CD134-PE (340420; BD Biosciences), CD95-PE-CF594 (562395; BD Biosciences), CD45RA-PeCy7 (337167; BD Biosciences) before fixing with 1% PFA.

For cytokine responses by intracellular staining (ICS), cells were stimulated in complete-RPMI with 100 μg/ml overlapping Spike peptide pool (181 peptides, 0.6μg/ml per peptide; BEI Resources, NR-52402), *other peptide pools,* or DMSO (1%), as a negative control, in combination with anti-CD28/CD49d (1:100, 347690; BD Biosciences) and 10U/ml IL-2 (11147528001; Roche), with Brefeldin A (1/2000 dilution; 555029; BD Biosciences) added after 6 hours. Following further 18 hours of the stimulation, cells were washed twice with MACS buffer (PBS/0.5% BSA, 2mM EDTA), then stained with surface antibodies: CD3-BV510 (317332; BioLegend), CD4-BV650 (563875; BD Biosciences), CD8-PerCPCy5.5 (565310; BD Biosciences), and Live/Dead NIR (L34976; Invitrogen) for 30 mins on ice. Cells were washed twice, then fixed using the BD Cytofix/Cytoperm kit (554723; BD Biosciences) according to the manufacturer's instructions, washed twice and intracellularly stained with IFNγ-v450 (560371; BD Biosciences), MIP-1β-FITC (560565; BD BioSciences), TNF-AF700 (557996; BD Biosciences), IL-5-APC (504306; BioLegend) and IL-13-PE (559328; BD BioSciences) for 30 mins on ice. Following two further washes, lymphocytes were resuspended in MACS buffer and acquisition was on a LSRII Fortessa.

Data were analysed using FlowJo v10. Values obtained for PBMCs cultured with DMSO under the same conditions (negative controls) were subtracted from peptide-stimulated values. As a negative control, PBMCs cultured with DMSO also had IL-2, thus this control accounts for any background cytokine production triggered by IL-2 by $CD4^+$ and $CD8^+$ T cells.

References

1. Reed L.J., Muench H. A simple method for estimating fifty per cent endpoints. The American Journal of Epidemiology. 1938;27(3):493-7.
2. Lopez E., Haycroft E.R., Adair A., Mordant F.L., O'Neill M.T., Pymm P., et al. Simultaneous evaluation of antibodies that inhibit SARS-CoV-2 variants via multiplex assay. JCI Insight. 2021;6:16-31.
3. Tan C.W., Chia W-N, BE Y, et al. Pan-Sarbecovirus neutralizing antibodies in BNT162b2-immunized SARS-CoV-1 survivors. N Engl J Med. 2021;385:1401–1406.
4. Tan C.W., Chia, W.N., Zhu, F. et al. SARS-CoV-2 Omicron variant emerged under immune selection. Nat Microbiol. 2022;7:1756–1761.
