# Supplementary material for: Interim results from a phase I randomized, placebo-controlled trial of novel SARS-CoV-2 beta variant receptor-binding domain recombinant protein and mRNA vaccines as a 4th dose booster
Source: eBioMedicine. 2023 Nov 27;98:104878. doi: 10.1016/j.ebiom.2023.104878 (PMC10696466; doi:10.1016/j.ebiom.2023.104878)
Supplement: Study Protocol Version [file mmc2.pdf]

CLINICAL STUDY PROTOCOL

Protocol Title: A Phase I, randomised, double-blind, placebo-controlled, dose-escalation study of adjuvanted SARS-CoV-2 beta variant receptor-binding domain recombinant protein (DoCo-Pro-RBD-1 + MF59®) and mRNA ®(MIPSCo-mRNA-RBD-1) vaccines in healthy adults aged 18 to 64 years previously vaccinated with 3 doses of licensed SARS-CoV-2 ancestral strain vaccines.

**Protocol Number:** UoM-SARS-CoV-2-01	**Version:** 4.1	**Date**: 18 August 2022

Short Title: Phase I study of adjuvanted SARS-CoV-2 beta variant receptor-binding domain recombinant protein (DoCo-Pro-RBD-1 + MF59®) and mRNA (MIPSCo-mRNA-RBD-1) vaccines in healthy adults.

Investigational Products: (1) DoCo-Pro-RBD-1, with MF59®: dose levels of 5µg, 15µg, and 45µg. (2) MIPSCo-mRNA-RBD-1, in lipid nanoparticle: dose levels of 10µg, 20µg and 50µg.

Study Phase: Phase I

Sponsor Name and Address: The University of Melbourne, Parkville, Vic 3010 Australia.

Manufacturers:
(1) DoCo-Pro-RBD-1 Antigen Drug Product: CSL Innovation for the Doherty Institute, University of Melbourne, Parkville, Vic 3010 Australia; (2) MF59® Adjuvant: Seqirus Inc., 475 Green Oaks Parkway, Holly Springs, North Carolina 27540, USA; (3) MIPSCo-mRNA-RBD-1Antigen Drug Product: IDT Australia Limited for the Monash Institute of Pharmaceutical Sciences, 399 Royal Parade, Parkville, Vic 3052, Australia

**TGA CTN ID:** 04968-1	**Approval Date:** 28 February 2022 (v3.1)
**ClinicalTrials.gov Identifier:** NCT05272605	**Registration date:** 09 March 2022

Study Sites: (1) Vaccine and Immunisation Research Group (VIRGo), Doherty Institute, University of Melbourne, Parkville, Vic 3010 Australia; (2) Royal Melbourne Hospital, Victorian Infectious Disease Service (VIDS), Level 2, Southwest, 300 Grattan Street, Parkville, Vic 3052 Australia

Co-ordinating Principal Investigator: Professor Terry Nolan, Head, VIRGo, Doherty Institute

Principal Investigator: A/Professor Joseph Sasadeusz, Victorian Infectious Disease Service (VIDS), Royal Melbourne Hospital

Investigators: Prof Sharon Lewin, Consultant Physician, VIDS, Royal Melbourne Hospital, and Director of the Doherty Institute; Prof Dale Godfrey, Immunology Theme Leader, Doherty Institute; Dr Irani Thevarajan, Infectious Diseases Physician, VIDS, Royal Melbourne Hospital; Prof Joe Torresi, Infectious Diseases Physician at Knox Private and the Austin Hospitals, and Dept of Microbiology & Immunology, University of Melbourne and Doherty Institute; Dr Briony Price, VIRGo, Doherty Institute, University of Melbourne and Murdoch Children's Research Institute.

Sponsor Medical Monitor: Name and Contact Details are provided in the Manual of Procedures.

Protocol Authors: Prof Terry Nolan; Prof Dale Godfrey; Dr Maryanne Griffith, Clinical Trial Program Coordinator, VIRGo, Doherty Institute; Sabine Braat, Senior Research Fellow (Biostatistics), Melbourne School of Population and Global Health; Prof Julie Simpson, Director of the Methods and Implementation Support for Clinical and Health Research Hub, Melbourne School of Population and Global Health.

SPONSOR SIGNATURE PAGE

**Sponsor:**	The University of Melbourne
**Protocol No:**	UoM-SARS-CoV-2-01
**Investigational Vaccines:**	(1) DoCo-Pro-RBD-1, with MF59® and<br>(2) MIPSCo-mRNA-RBD-1, in lipid nanoparticle
**Protocol Title**:	"*A Phase I, randomised, double-blind, placebo-controlled, dose-escalation study of adjuvanted SARS-CoV-2 beta variant receptor-binding domain recombinant protein (DoCo-Pro-RBD-1 + MF59®) and mRNA ®(MIPSCo-mRNA-RBD-1) vaccines in healthy adults aged 18 to 64 years previously vaccinated with 3 doses of licensed SARS-CoV-2 ancestral strain vaccines*".
**Version & Date:**	Version 4.1 Dated 18 August 2022

The signature below constitutes the approval of this protocol and the appendices and provides the necessary assurances that this trial will be conducted according to all protocol requirements and in accordance with local regulatory requirements and Good Clinical Practice principles as outlined in ICH Guidelines.

**Signature of The University of Melbourne Authorised Signatory**<br>**Name:** Professor Terry Nolan<br>**Date:**

TABLE OF CONTENTS

SPONSOR SIGNATURE PAGE 2
1. PROTOCOL SUMMARY 7
1.1. Synopsis 7
1.2. Study Schema 21
1.2.1. Treatment Groups 21
Lower Dose (then Intermediate and Higher Dose) 21
1.2.2. Study Overview 22
1.2.3. Randomisation Overview 23
1.3. Schedule of Activities: Dose-Escalation and Expanded Phases 24
2. INTRODUCTION 26
2.1. Study Rationale 26
2.2. Background 26
2.3. Risk/Benefit Assessment 27
2.3.1. Known Potential Risks 27
2.3.2. Known Potential Benefits 28
3. OBJECTIVES AND ENDPOINTS 29
4. STUDY DESIGN 32
4.1. Overall Design 32
4.2. Scientific Rationale for Study Design 32
4.3. Justification for Dose 32
4.3.1. Dose Selection: Recombinant protein vaccine 32
4.3.2. Dose Selection: mRNA vaccine 33
4.3.3. Dose Selection: MF59® 33
4.4. End of Study Definition 33
4.5. Dose-Escalation Criteria 33
4.5.1. Opening Rules: Expanded Phase 33
4.6. Study Stopping Criteria 34
4.6.1. Stopping Criteria for Individual Participants 34
4.6.2. Dose-escalation Stopping /Pausing Rules 35
4.6.3. Participants with COVID-19-Like Illness During the Study 36
5. STUDY POPULATION 37
5.1. Inclusion Criteria 37
5.2. Exclusion Criteria 37
5.3. Lifestyle Considerations 39
5.3.1. Meals and Dietary Restrictions 39
5.3.2. Caffeine, Alcohol and Tobacco 39
5.3.3. Physical Activity 39

5.4. Screen Failures 39
6. STUDY PRODUCT 40
6.1. Study Product and Administration 40
6.1.1. Study Product Formulations 40
6.1.2. Dosing and Administration 40
6.1.3. Dose Escalation 41
6.1.4. Dose Modification 41
6.2. Study Product Preparation/Handing/Storage/Accountability 41
6.2.1. Study Product Acquisition and Accountability 41
6.2.2. Study Product Packaging and Labelling 42
6.2.3. Study Product Storage and Stability 42
6.2.4. Study Vaccine Preparation 42
6.3. Measures to Minimise Bias: Randomisation and Blinding 42
6.3.1. Randomisation and Blinding 42
6.3.2. Blinding and Masking Procedures 43
6.3.3. Emergency Unblinding 44
6.4. Study Vaccine Compliance 44
6.5. Concomitant Medications 44
6.6. Dose Modification 45
6.7. Availability of Study Vaccines After the End of the Study 45
7. DISCONTINUATION OF STUDY TREATMENT AND PARTICIPANT DISCONTINUATION /WITHDRAWAL 46
7.1. Discontinuation of Study Treatment 46
7.2. Participant Discontinuation/Withdrawal from the Study 46
7.3. Lost to Follow-Up 47
8. STUDY ASSSESMENTS AND PROCEDURES 48
8.1. Screening Procedures 48
8.2. Safety Assessments 48
8.2.1. Physical Examinations 48
8.2.2. Vital Signs 48
8.2.3. Clinical Safety Laboratory Assessments 49
8.2.4. Interpretation of Screening Assessments 49
8.3. Adverse Events and Serious Adverse Events 49
8.3.1. Definition of Adverse Event (AE) 49
8.3.1.1. Solicited Adverse Events 50
8.3.1.2. Unsolicited Adverse Events 52
8.3.2. Definition of Serious Adverse Event (SAE) 52
8.3.3. Suspected Unexpected Serious Adverse Reaction (SUSAR) 53
8.3.4. Adverse Event of Special Interest (AESIs) 53
8.3.5. Time period and Frequency for Adverse Event Assessment and Follow-Up 54
8.3.6. Assessment of Adverse Event Causality and Outcome 54
8.3.7. Reporting of Adverse Events 54
8.3.8. eDiary 55
8.3.9. Pregnancy 55

8.4. Treatment of Overdose 55
8.5. Pharmacokinetics (Not applicable) 55
8.6. Pharmacodynamics (Not applicable) 55
8.7. Genetics (Not applicable) 55
8.8. Immunogenicity 55
8.8.1. Antibody Responses 56
8.8.2. T-cell Response 56
8.8.3. Memory B cell Staining and Sequencing and Clonotype Analysis 56
9. STATISTICAL CONSIDERATIONS 57
9.1. Statistical Hypotheses 57
9.2. Sample Size Determination 57
9.3. Populations for Analyses 57
9.4. Statistical Analyses 59
9.4.1. Demographic and Baseline Data 59
9.4.2. Safety Analyses 59
9.4.3. Immunogenicity Analyses 60
9.4.4. COVID-19 Cases During the Study 61
9.5. Interim Analyses 61
9.6. Data Safety Monitoring Board (DSMB) 61
10. SUPPORTING DOCUMENTATION AND OPERATING CONSIDERATIONS 62
10.1. Regulatory, Ethical and Study Oversight Considerations 62
10.1.1. Informed Consent Procedure 62
10.1.2. Study Termination and Closure 62
10.1.3. Confidentiality and Privacy 63
10.1.4. Secondary Use of Stored Specimens and Data 63
10.1.5. Key Roles and Study Governance 63
10.1.6. Safety Oversight 64
10.1.7. Clinical Site Monitoring 64
10.1.8. Quality Control (QC) and Quality Assurance (QA) 64
10.1.9. Data Handling and Record Keeping 65
10.1.10. Protocol Deviations 66
10.1.11. Publication and Data Sharing 66
10.2. Abbreviations 67
10.3. Protocol Amendment History 68
11. REFERENCES 69
12. LIST OF TABLES 70
13. LIST OF FIGURES 70
14. APPENDICES 71
APPENDIX 1 71
APPENDIX 2 73
APPENDIX 3 75

STATEMENT OF COMPLIANCE

This study will be performed in accordance with the approved clinical trial protocol, Human Research Ethics Committee (HREC) and informed consent regulations, Good Clinical Practice (GCP) and their applicable amendments, as required by the following:

- Integrated Addendum to ICH E6(R1): Guideline for Good Clinical Practice E6 (R2), 9 November 2016
- Declaration of Helsinki concerning medical research in humans (Revised Brazil, 2013)
- The National Health and Medical Research Council, Australia, Statement on Ethical Conduct in Human Research 2007 (Updated 2018)

Study commencement will only occur after receipt of formal approval from a competent HREC and CTN acknowledgment by the Therapeutic Goods Administration (TGA).

1. PROTOCOL SUMMARY

1.1. Synopsis

**Title**	"A Phase I, randomised, double-blind, placebo-controlled, dose-escalation study of adjuvanted SARS-CoV-2 beta variant receptor-binding domain recombinant protein (DoCo-Pro-RBD-1 + MF59®) and mRNA ®(MIPSCo-mRNA-RBD-1) vaccines in healthy adults aged 18 to 64 years previously vaccinated with 3 doses of licensed SARS-CoV-2 ancestral strain vaccines".
**Protocol Number**	UoM-SARS-CoV-2-01
**Study Sponsor**	The University of Melbourne, Parkville, Victoria 3010 Australia
**Phase**	I
**Study Design**	This is a randomised, double-blind, placebo-controlled, dose-escalation phase I study evaluating the safety and immunogenicity of SARS-CoV-2 beta variant DoCo-Pro-RBD-1 + MF59® vaccine and MIPSCo-mRNA-RBD-1 vaccine. The study will enroll up to 76 healthy adults aged 18 to 64 years, previously vaccinated with *Comirnaty™* (BNT162b2 mRNA) COVID-19 vaccine (Pfizer Australia Pty Ltd) or *Vaxzevria™* (ChAdOx1-S) COVID-19 vaccine (AstraZeneca Pty Ltd)), and boosted with either *Comirnaty™* or *Spikevax™* (mRNA-1273, Moderna).<br><br>The study will consist of two overlapping parts; A **Dose-Escalation Phase** (n=18 participants), an **Expanded Phase** (n=58 participants).<br><br>The aim of the Dose-Escalation Phase and the Expanded Phase of the study is to evaluate the safety and immunogenicity of single doses of DoCo-Pro-RBD-1 + MF59® and MIPSCo-mRNA-RBD-1 in healthy adult participants 18 to 64 years of age who have previously received 2 primary course doses of either *Comirnaty™* or *Vaxzevria™*, and a third booster dose of either *Comirnaty™* or *Spikevax™*. Participants will be randomised to receive DoCo-Pro-RBD-1 + MF59® at three dose levels (5µg, 15µg, or 45µg), RBD-mRNA vaccine at three dose levels (10µg, 20µg or 50µg) or placebo (normal saline). Participants will receive one dose of study vaccine, or placebo, on Day 1 and will remain on study for safety data collection and immunogenicity assessments until Day 181.<br><br>The **Dose-Escalation Phase** and **Expanded Phase** will occur in a stepwise dose-escalation design. Randomisation will be stratified by prior COVID-19 vaccination with *Comirnaty™* or *Vaxzevria™* (See Section 1.3).<br><br>In Sentinel Group 1 (Lower-dose Group), 6 participants previously vaccinated with 2 doses of *Comirnaty™* or 2 doses of *Vaxzevria™* and a third booster dose of either *Comirnaty™* or *Spikevax™* will be randomised in a 1:1:1 ratio to receive either 5µg DoCo-Pro-RBD-1 + MF59®, 10µg MIPSCo-mRNA-RBD-1, or placebo, intramuscularly (IM) in a blinded fashion on Day 1. Participants will be observed for reactogenicity for 7 days following vaccination. Recruitment into the study will be paused and the cumulative 7-day safety data will be reviewed against the Study Stopping Rules. If no Stopping Rules are met, a further 32 participants will be enrolled into Cohorts 1a to 1f in the **Expanded Phase** of the study. They will be randomised in a ratio of 7:7:2 to receive either 5µg DoCo-Pro-RBD-1 + MF59®, 10µg MIPSCo-mRNA-RBD-1, or placebo. Following the completion of Day 8 visits by the study participants, the DSMB will review the combined 7-day post-vaccination safety data for Sentinel Group 1 and Cohorts 1a to 1f in the Expanded Phase. If the data are found to be acceptable, sentinel dosing with 15µg DoCo-Pro-RBD-1 + MF59®, 20µg MIPSCo-mRNA-RBD-1 or placebo in a further 6 participants in Sentinel Group 2 (Intermediate-dose Group) will commence in the same manner and in accordance with the schema in Section 1.2.2. Enrolment will be paused to review the cumulative 7-day safety data against the Stopping Rules. If

	no Stopping Rules are met, a further 13 participants, will be enrolled into in the Expanded Phase and randomised in a ratio of 6:6:1 to receive either 15µg DoCo-Pro-RBD-1 + MF59®, 20µg MIPSCo-mRNA-RBD-1, or placebo.<br><br>Enrolment of the final 6 participants in Sentinel Group 3 (Higher-dose Group) will occur following a positive recommendation by the DSMB following a review of the 7-day safety data in Sentinel Group 2 (Intermediate-dose Group) participants in the Expanded Phase.<br><br>Participants in Sentinel Group 3 (Higher-dose Group) will be randomised to receive 45µg DoCo-Pro-RBD-1 + MF59®, 50µg MIPSCo-mRNA-RBD-1 or placebo. Enrolment will be paused to review the cumulative 7-day safety data against the Stopping Rules. If no Stopping Rules are met, a further 13 participants will be enrolled into Cohorts 3a to 3c in the Expanded Phase and randomised in a ratio of 6:6:1 to receive either 45µg DoCo-Pro-RBD-1 + MF59®, 50µg MIPSCo-mRNA-RBD-1, or placebo.
**Rationale**	The COVID-19 pandemic caused by SARS-CoV-2 has infected over 253 million people, claimed more than 5.1 million lives (Johns Hopkins University, 2021) and adversely affected economies globally. The best chance to overcome this pandemic is with safe and effective vaccines. There are currently over 200 vaccine candidates in active development and more than 40 are in clinical trials. These include whole inactivated virus, viral vectors expressing spike (S), isolated S proteins, including some with structure stabilisation or multimer scaffolds, mRNA, and DNA vaccines. While this is impressive and encouraging, there are known adverse reactions, albeit relatively rare, associated with S-based vaccines, including thrombocytopaenia with thrombosis syndrome (TSS) with the Astra Zeneca vaccine, and myocarditis with the S mRNA vaccines (Pfizer and Moderna) and moreover, with the exception of the locally manufactured Astra Zeneca vaccine, Australia is reliant on vaccines manufactured overseas.<br><br>As the pandemic evolves, mutations are emerging, several of which have appeared in Australia, that are less susceptible to immunity to the original SARS-CoV-2 strain used for all current vaccines. The solution to this problem may lie in a vaccine that can be rapidly tuned, providing optimal immunity to the variants that are currently circulating.<br><br>The SARS-CoV-2 receptor-binding domain (RBD) is located at the tip of the spike and is responsible for the virus adhering to human ACE-2 and subsequent virus infection (Wang, 2020). Moreover, this small region of the virus is the target of over 90% of neutralising antibodies following SARS-CoV-2 infection (Piccoli, 2020). For this reason, the Doherty Institute for Infection and Immunity and the Monash Institute of Pharmaceutical Sciences have developed two RBD-focused vaccine candidates that are efficient to produce and can be rapidly modified to incorporate distinct or multiple RBD variants arising in circulation.<br><br>DoCo-Pro-RBD-1 is based on the SARS-CoV-2 beta variant RBD, generated as a human IgG1 Fc-domain fusion protein, to facilitate production and purification and multimeric presentation to the immune system while also engaging FcR+ antigen-presenting cells for enhanced immunological priming. This vaccine induces high titres of RBD-specific antibodies, including high neutralising antibody titres, in mice following a prime and boost regimen. Immunity induced by this vaccine is durable and protects against virus challenge in a mouse model of SARS-CoV-2 infection, even 100 days following the boost. Because the most immune-evasive variant to date was the beta variant, we used the RBD from this variant as the basis for our vaccine. It has been shown to induce strong neutralising antibody

	immunity against this variant of concern, and protects mice against infection with this strain, while still retaining its potential to neutralise the original ancestral (Wuhan) strain virus.<br><br>MIPSCo-mRNA-RBD-1 is an mRNA vaccine encoding the SARS-CoV-2 beta variant RBD attached to the transmembrane and cytoplasmic domains of the S protein. Injection of the product results in expression of a membrane-anchored RBD, rather than whole S protein. In common with approved mRNA vaccines, the mRNA is formulated in lipid nanoparticles using four lipids all of which have been used in FDA-approved products. This vaccine induces high titres of RBD-specific antibodies, including high neutralising antibody titres, in mice following a prime and boost regimen. Immunity induced by this vaccine is durable and protects against virus challenge in a mouse model of SARS-CoV-2 infection. Because the most immune-evasive variant was the beta variant, we used the RBD from this variant as the basis for our vaccine. It has been shown to induce strong neutralising antibody immunity against this variant of concern, while still retaining its potential to neutralise the original ancestral strain virus.<br><br>The study will be conducted in two overlapping parts (see Figures 2 and 3): The Dose-Escalation Phase with the Expanded Phase is a dose-escalation study of a single booster dose of SARS-CoV-2 beta variant DoCo-Pro-RBD-1 + MF59® at three dose levels (5µg, 15µg, or 45µg), SARS-CoV-2 RBD-mRNA vaccine at three dose levels (10µg, 20µg or 50µg) or placebo (normal saline) in participants previously vaccinated with a 2-dose schedule of *Comirnaty™* or *Vaxzevria™*, and boosted with either *Comirnaty™* or *Spikevax™* at least 3 months (≥90 days) prior to study vaccination.
**Target Population Dose-Escalation Phase**	Healthy adults aged 18 to 64 years of age inclusive, previously vaccinated a 2-dose schedule of *Comirnaty™* or *Vaxzevria™*, and boosted with either *Comirnaty™* or *Spikevax™* at least 3 months (≥90 days) prior to study vaccination.
**Target Population Expanded Phase**	Healthy adults aged 18 to 64 years of age inclusive, previously vaccinated with a 2-dose schedule of *Comirnaty™* or *Vaxzevria™*, and boosted with either *Comirnaty™* or *Spikevax™* at least 3 months (≥90 days) prior to study vaccination.

**Participant Eligibility**	**<u>Inclusion Criteria</u>**<br>To be included in this study, an individual must satisfy all the following criteria:<br>1. Adults 18 to 64 years of age, inclusive at screening, previously vaccinated with a 2-dose schedule of *Comirnaty™* or *Vaxzevria™*.<br>2. ≥3 months (≥90 days) since receipt of a booster dose of either *Comirnaty™* or *Spikevax™*.<br>3. Be in good health as determined by medical history, physical examination, vital signs, and clinical laboratory assessments with no clinically significant abnormalities as judged by the Investigator at screening and randomisation. Vital signs must be within medically acceptable ranges prior to the first vaccination.<br>4. Participants of childbearing potential (defined as any participant who has experienced menarche and who is NOT surgically sterile [ie, hysterectomy, bilateral tubal ligation, or bilateral oophorectomy] or postmenopausal [defined as amenorrhea for at least 12 consecutive months]) must agree to be heterosexually inactive from at least 28 days prior to enrollment and through the end of the study OR agree to consistently use a medically acceptable method of contraception listed below from at least 28 days prior to enrollment and through the end of the study.<br>• Condoms (male or female); Diaphragm; Cervical cap; Intrauterine device; Oral or patch contraceptives; Norplant®, Depo-Provera®, or another regulatory approved contraceptive method; Abstinence, as a form of contraception, is acceptable if in line with the participant's lifestyle.<br>**NOTE:** Periodic abstinence (e.g., calendar, ovulation, symptom-thermal, post-ovulation methods) and withdrawal method (coitus interruptus) are not acceptable forms of contraception.<br>5. Agrees to not participate in any other SARS-CoV-2 prevention or treatment trials for the duration of the study.<br>6. Willing and able to give informed consent prior to study enrollment and to comply with all study procedures.<br>**<u>Exclusion Criteria</u>**<br>If an individual meets any of the following criteria, he or she is ineligible for this study:<br>1. History of SARS-CoV-2 infection (confirmed by PCR or rapid antigen test (RAT)) within 3 months (90 days) prior to randomisation.<br>2. Participants with a BMI > $35kg/m^2$.<br>3. Positive result for rheumatoid factor (RF) at Screening.<br>4. Positive test at Screening for human immunodeficiency virus (Types 1 or 2) antibody, hepatitis B surface antigen or hepatitis C virus antibody.<br>5. Clinical laboratory test results not within normal range and judged to be clinically relevant abnormalities by the investigator.<br>6. History of prior cardiac inflammatory disease (endocarditis, myocarditis or pericarditis).<br>7. History of demyelinating disease or Guillain Barré syndrome.<br>8. Fever (non-axillary temperature >37.5°C) or any other symptoms of infection that have not completely resolved within 3 days prior to Randomisation (Day 1).<br>9. Presence of current active viral infection or bacterial infection, at Screening or Randomisation (Day 1), which is determined by the Investigator to be of clinical significance.<br>10. Participation in research involving receipt of an investigational product (drug/biologic/device) within 90 days prior to the first study vaccination or an intention to participate in another clinical trial at any time.

	11. Received any other vaccine within 30 days prior to the first study vaccination, other than licensed influenza vaccine which can be administered up to 14 days prior to randomization.<br>12. Any known allergies to products contained in the investigational products.<br>13. Any history of anaphylaxis to any prior vaccine, food, drug, toxin or other exposure.<br>14. Autoimmune or immunodeficiency disease/condition (iatrogenic or congenital) requiring ongoing immunomodulatory therapy.<br>**NOTE:** Stable endocrine disorders (e.g., thyroiditis, pancreatitis), including stable diabetes mellitus with no history of diabetic ketoacidosis) are NOT excluded.<br>15. Chronic administration (defined as > 14 continuous days) of immunosuppressant, systemic glucocorticoids, or other immune-modifying drugs within 90 days prior to first study vaccination.<br>**NOTE:** An immunosuppressant dose of glucocorticoid is defined as a systemic dose ≥ 10mg of prednisone per day or equivalent. The use of topical or intranasal glucocorticoids is permitted. Topical tacrolimus and ocular cyclosporin are permitted.<br>16. Received immunoglobulin, blood-derived products, or immunosuppressant drugs or donation of blood/blood products within 90 days prior to vaccination or planned receipt or donation during the study period.<br>17. Thrombocytopaenia, contraindicating intramuscular vaccination, based on the Investigator's judgment.<br>18. Bleeding disorder, or receipt of anticoagulants in the 3 weeks preceding inclusion, contraindicating intramuscular vaccination based on the Investigator's judgement.<br>19. Active cancer (malignancy) on therapy within one year prior to first study vaccination (with the exception of adequately treated non-melanomatous skin carcinoma or lentigo malignancy and uterine cervical carcinoma *in situ* without evidence of disease, at the discretion of the Investigator).<br>20. Participants who are breastfeeding, pregnant or who plan to become pregnant prior to the end of study.<br>21. Suspected or known history of alcohol abuse or drug addiction within 2 years prior to the first study vaccine dose that, in the opinion of the Investigator, might interfere with protocol compliance.<br>22. Have a tattoo/scar/birthmark or any other skin condition affecting the deltoid area that may, in the Investigator's opinion, interfere with injection site assessments.<br>23. Any other condition that, in the opinion of the Investigator, would pose a health risk to the participant if enrolled or could interfere with evaluation of the trial vaccine or interpretation of study results (including neurologic or psychiatric conditions likely to impair the quality of safety reporting).<br>24. Study team member or immediate family member of any study team member(inclusive of Sponsor, CRO, and study site personnel involved in the conductor planning of the study).<br>25. Aboriginal and Torres Strait Islander person aged 50 years or older.

<table>
<tr>
<td>Dose-Escalation Phase</td>
<td>Number of participants: 18
<table>
<tr><th>Group</th><th>Study Vaccine & Dose Level (Administered on Day 1)</th><th>Total N</th></tr>
<tr><td>Sentinel Group 1 (Lower-dose Group)</td><td>5μg DoCo-Pro-RBD-1 + MF59® (n=2), 10μg MIPSCo-mRNA-RBD-1 (n=2) or placebo (n=2)</td><td>6</td></tr>
<tr><td>Sentinel Group 2 (Intermediate-dose Group)</td><td>15μg DoCo-Pro-RBD-1 + MF59® (n=2), 20μg MIPSCo-mRNA-RBD-1 (n=2) or placebo (n=2)</td><td>6</td></tr>
<tr><td>Sentinel Group 3 (Higher-dose group)</td><td>45μg DoCo-Pro-RBD-1 + MF59® (n=2), 50μg MIPSCo-mRNA-RBD-1 (n=2) or placebo (n=2)</td><td>6</td></tr>
</table>
</td>
</tr>
<tr>
<td>Length of Participation</td>
<td>On study (including screening and follow-up): 181 days with vaccine administration on Day 1.</td>
</tr>
<tr>
<td>Expanded Phase</td>
<td>Number of participants: 58
<table>
<tr><th>Cohort</th><th>Prior 2-dose Covid-19 Vaccination</th><th>Study Vaccine (Administered on Day 1)</th><th>Dose Level</th><th>N</th></tr>
<tr><td>1a</td><td>Comirnaty™</td><td>DoCo-Pro-RBD-1 + MF59®</td><td>5μg</td><td>7</td></tr>
<tr><td>1b</td><td>Comirnaty™</td><td>MIPSCo-mRNA-RBD-1</td><td>10μg</td><td>7</td></tr>
<tr><td>1c</td><td>Comirnaty™</td><td>Placebo</td><td>-</td><td>2</td></tr>
<tr><td>1d</td><td>Vaxzevria™</td><td>DoCo-Pro-RBD-1 + MF59®</td><td>5μg</td><td>7</td></tr>
<tr><td>1e</td><td>Vaxzevria™</td><td>MIPSCo-mRNA-RBD-1</td><td>10μg</td><td>7</td></tr>
<tr><td>1f</td><td>Vaxzevria™</td><td>Placebo</td><td>-</td><td>2</td></tr>
<tr><td>2a</td><td>Comirnaty™ Or Vaxzevria™</td><td>DoCo-Pro-RBD-1 + MF59®</td><td>15μg</td><td>6</td></tr>
<tr><td>2b</td><td>Comirnaty™ Or Vaxzevria™</td><td>MIPSCo-mRNA-RBD-1</td><td>20μg</td><td>6</td></tr>
<tr><td>2c</td><td>Comirnaty™ Or Vaxzevria™</td><td>Placebo</td><td>-</td><td>1</td></tr>
<tr><td>3a</td><td>Comirnaty™ Or Vaxzevria™</td><td>DoCo-Pro-RBD-1 + MF59®</td><td>45μg</td><td>6</td></tr>
<tr><td>3b</td><td>Comirnaty™ Or Vaxzevria™</td><td>MIPSCo-mRNA-RBD-1</td><td>50μg</td><td>6</td></tr>
<tr><td>3c</td><td>Comirnaty™ Or Vaxzevria™</td><td>Placebo</td><td>-</td><td>1</td></tr>
</table>
</td>
</tr>
<tr>
<td>Length of Participation</td>
<td>On study (including screening and follow-up): 181 days with vaccine administration on Day 1.</td>
</tr>
</table>

**Interventional Product (IP) Dosage and Mode of Administration**<br><br>**Dose-Escalation Phase and Expanded Phase**	1. **DoCo-Pro-RBD-1 (5µg):** supplied as a solution for preparation for injection, at a concentration of 0.18mg/mL of antigen and diluted to 0.02mg/mL in pH6.5 citrate buffer diluent. The diluted antigen is mixed with an equal volume of MF59® adjuvant. The DoCo-Pro-RBD-1 (5 µg) vaccination regimen will comprise one IM injection (Day 1) of 0.5 mL injection volume at a dose of 5µg antigen.<br>2. **DoCo-Pro-RBD-1 (15µg):** supplied as a solution for preparation for injection, at a concentration of 0.18mg/mL of antigen and diluted to 0.06mg/mL in pH6.5 citrate buffer diluent. The diluted antigen is mixed with an equal volume of MF59® adjuvant. The DoCo-Pro-RBD-1 (15µg) vaccination regimen will comprise one IM injection (Day 1) of 0.5 mL injection volume at a dose of 15 µg antigen.<br>3. **DoCo-Pro-RBD-1 (45µg):** supplied as a solution for preparation for injection, at a concentration of 0.18mg/mL of antigen and mixed with an equal volume of MF59® adjuvant. The DoCo-Pro-RBD-1 (45µg) vaccination regimen will comprise one IM injection (Day 1) of 0.5 mL injection volume at a dose of 45 µg antigen.<br>4. **MIPSCo-mRNA-RBD-1 (10µg):** supplied as a solution for preparation for injection, at a concentration of 100µg/ml antigen and diluted to 20µg/ml in isotonic tromethamine/sucrose buffer solution (pH 7.2-7.4). The MIPSCo-mRNA-RBD-1 (10µg) vaccination regimen will comprise one IM injection (Day 1) of 0.5 mL injection volume at a dose of 10µg antigen.<br>5. **MIPSCo-mRNA-RBD-1 (20µg):** supplied as a solution for preparation for injection, at a concentration of 100µg/ml antigen and diluted to 40µg/ml in isotonic tromethamine/sucrose buffer solution (pH 7.2-7.4). The MIPSCo-mRNA-RBD-1 (20µg) vaccination regimen will comprise one IM injection (Day 1) of 0.5 mL injection volume at a dose of 20ug antigen.<br>6. **MIPSCo-mRNA-RBD-1 (50µg):** supplied as a solution for preparation for injection, at a concentration of 100µg/ml antigen in isotonic tromethamine/sucrose buffer solution (pH 7.2-7.4). The MIPSCo-mRNA-RBD-1 (50µg) vaccination regimen will comprise one IM injection (Day 1) of 0.5 mL injection volume at a dose of 50µg antigen.<br>7. **Placebo:** Normal saline (0.9% sodium chloride, commercially sourced) supplied as a solution for injection. The placebo vaccination regimen will comprise one IM injection (Day 1) of 0.5 mL injection volume.<br><br>All study interventions will be prepared by unblinded study pharmacy staff as 0.5 mL volume in a syringe that will be covered to maintain the blind during IM injection into the deltoid muscle. The IP will be administered by blinded study personnel.
**Primary Objective and Corresponding Endpoints**	Objective: To evaluate the safety and immunogenicity of a single dose of 3 dose levels of DoCo-Pro-RBD-1 + MF59® or MIPSCo-mRNA-RBD-1, in healthy adults aged 18 to 64 years, inclusive and previously vaccinated with a 2-dose schedule of *Comirnaty*™ or *Vaxzevria*™, and boosted with either *Comirnaty*™ or *Spikevax*™ at least 3 months (≥90 days) prior to study vaccination.<br><br>Endpoints:<br>1. Serious adverse events (SAEs), medically attended adverse events (MAAEs) and any adverse events (AEs) leading to study withdrawal at any time during the study [through to completion at Day 181].<br>2. SAEs from Day 1 to 29 (28 days post vaccination).<br>3. Solicited local and systemic reactogenicity AEs within 7 days after vaccination (Day 1) by severity score, duration and peak intensity.<br>4. Unsolicited AEs from Day 1 to Day 29 (28 days post vaccination).

	5. Percentage of participants subjects who achieve a boost response (defined as a 4-fold increase in SARS-CoV-2 neutralising or RBD-specific Ab titres from baseline) by Day 29 (28 days after vaccination).
**Secondary Objectives and Corresponding Endpoints**	Objectives:<br>1. To assess the safety of a single-dose schedule of 3 dose levels of IM administration of each candidate vaccine throughout the study period, within 28 days after vaccination and for 6 months after vaccination.<br>2. To evaluate the immunogenicity as measured by ELISA to detect Ab against the SARS-CoV-2 RBD protein, and by neutralising assay against SARS-CoV-2 virus, at baseline (Day 1), Day 29 (28 days after vaccination), and 3, and 6 months after vaccination using 3 dose levels of each candidate vaccine.<br>3. To assess antigen-specific T-cell response at baseline (Day 1), Day 8 (7 days after vaccination), Day 29 (28 days after vaccination) and day 91 (3 months after vaccination) using 3 dose levels of each candidate vaccine.<br>4. To assess the balance of T-cell immunity, for example type 1 (Th1) versus type 2 (Th2) cytokines at each timepoint where T cells are assessed as per secondary objective 3.<br><br>Endpoints:<br>1. MAAE from Day 1 to 6 months after vaccination using MeDRA classification, severity score and relatedness.<br>2. Percentage of participants who seroconvert by 3-, and 6-months post vaccination. The seroconversion rate will be calculated based on test results reaching quantifiable Ab level after vaccination.<br>3. Geometric mean titre of RBD-specific Ab and nAb titres through 6 months post vaccination, including change from baseline in Ab titres (threshold of ≥ 4-fold increase from baseline titre).<br>4. Geometric mean fold rise (threshold of ≥ 4-fold increase from baseline titre) of RBD-specific Ab and nAb titres through 6 months post vaccination.<br>5. T-cell responses to SARS-CoV-2 RBD-derived peptide antigens; Specific T cell responses including activation, proliferation, and cytokine production, in response to RBD peptide antigens through 3 months post vaccination.
**Exploratory Objectives and Corresponding Endpoints**	Objective:<br>1. To compare antibody reactivity against SARS-CoV-2 beta variant to the ancestral SARS-CoV-2 strain.<br>2. To assess antibody reactivity against other SARS-CoV-2 Variants of Concern (VOCs).<br>3. To assess dominant antibody clonotypes against SARS-CoV-2 RBD.<br>4. To compare the impact of candidate RBD boosts following *Vaxzevria*™ and *Cominarty*™, to promote variant-specific responses.<br><br>Endpoints:<br>1. Geometric mean titre of SARS-CoV-2 variant-RBD-specific Ab and nAb titres through 6 months post last vaccination, including change from baseline in SARS-CoV-2 variant reactive Ab titres.<br>2. Memory B cell (MBC)frequency, specific for RBD including VoC reactive B cells in response to boosting (Dose-Escalation Phase and Expanded Phase of the study (Day 1, Day 8, Day 29, Day 91, Day 181).<br>3. MBC clonotype analysis and mass spectrometry analysis on serum antibodies to align with results from MBC single cell clonotype analysis on a subset of

	samples based on other antibody analysis (Dose-Escalation Phase and Expanded Phase of the study (Day 1, Day 29).

Procedures and Assessments:

For a detailed overview of the study design and schedule of events please refer to Section 1.2.1 (Treatment Groups), Section 1.2.2 (Study Overview), Section 1.2.3 (Randomisation details for all study phases), Section 1.3.1 (Schedule of Assessments - Dose-Escalation Phase (Sentinel Groups 1, 2 and 3) and Expanded Phase (Cohorts 1a to 3c)).

All Participants – Dose-Escalation Phase (Sentinel Groups 1, 2 & 3) and Expanded Phase (Cohorts 1a to 3c)

Once informed consent has been provided, an eligibility assessment will be performed for all study participants during the 14-day screening period. Screening assessments will include the collection of significant medical history data, baseline safety laboratory assessments and physical examination.

Following vaccination, participants in Sentinel Groups 1, 2 and 3 in the Dose-Escalation Phase of the study will be observed for a minimum of 4 hours for hypersensitivity/anaphylactic reactions or any other AEs. Participants in the Expanded Phase of the study will be observed for a minimum of 30 minutes post-vaccination. Events occurring within a 30-minute period following vaccination will be reported as Immediate AEs (IAEs).

All participants will be given access to an eDiary and instructed to record any AEs they experience during the study in the eDiary. During the first 7 days post-vaccination participants will be required to document the frequency and severity of any solicited local or systemic reactions in the eDiary. Adverse events will be collected to Day 29 for participants in the Dose-Escalation Phase and the Expanded Phase of the study. SAEs will be collected to Day 181.

Blood and urine samples for post-vaccination safety laboratory assessments will be collected on Day 7, 29 and Day 181 for participants in the Dose-Escalation Phase, and Day 29 and Day 181 for participants in the Expanded Phase of the study.

Post-vaccination immunogenicity blood samples will be collected on Day 8, Day 29, Day 91, and Day 181 for participants in both the Dose-Escalation Phase and the Expanded Phase of the study.

Vaccination with a licensed influenza vaccine is permitted but must be at least 14 days post study vaccination. Participants should not receive any other vaccine until at least 29 days following study vaccination and not before the scheduled blood draw at Visit 5.

COVID-19-Like Illness

Participants who experience any symptoms indicative of COVID-19-like illness during the study, must undergo diagnostic testing in accordance with Victorian Government public health directives to confirm the presence or absence of SARS-CoV-2 infection. Any diagnostic test confirmed positive result for SARS-CoV-2 in a participant during the study will be counted as an event. If possible, those participants who test positive (either by rapid antigen test (RAT) or PCR) should also be offered virus whole genome sequencing to be conducted at no expense to them through the Peter Doherty Institute.

Stopping/Pausing Rules

A review of the cumulative 7-day post-vaccination safety data after dosing of each of the Sentinel Groups in the Dose-Escalation Phase of the study will be undertaken to identify any safety concerns. Enrollment into the study and/or further vaccination will be paused in the event that any of the Stopping/Pausing Rules listed below are identified. The Stopping/Pausing Rules are considered to have been met only if the events described occur post administration of the study vaccines:

> Rule 1: One or more participant(s) experiences an SAE assessed as *at least possibly related* to investigational product (IP) by the Investigator (applies to both Study Phases);

Rule 2: Four (or more) participants experience the same or similar Grade 3 AE assessed as _at least possibly related_ to investigational product (applies to Expanded Study Phase);

Rule 3: One (or more) participants experiences an AESI assessed as _at least possibly related_ to investigational product (applies to both Study Phases);

Rule 4: Four (or more) participants develop a LASI assessed as _at least possibly related_ to investigational product (applies to Expanded Study Phase); and

Rule 5: The Investigator may, using their discretion, ask for the study to be placed on hold and a DSMB meeting be held for any single event or combination of multiple events which, in the Investigator's clinical judgment, jeopardise the safety of study participants or the reliability of the data.

If any of the above Stopping/Pausing Rules are met following a review of the cumulative 7-day post-vaccination safety following dosing of Sentinel Group 1, 2 or 3 participants in the Dose-Escalation Phase or at any other time during the study, enrollment into the study and administration of study vaccine will be temporarily paused by the Sponsor pending further evaluation. Details of the reported safety events will be shared with the DSMB for their review and recommendations. Case unblinding may be performed if necessary. The Sponsor will carefully consider the DSMB recommendation(s), assess the overall risk/benefit ratio and reach a final decision on further study vaccine administration, continuation, or termination of the study.

Study Evaluations

Safety Assessments

Safety evaluations during the study will include collection of solicited and unsolicited AEs and concomitant medications. participants will be given a digital thermometer to record temperature (non-axillary), a disposable measuring scale to record injection site erythema/redness & swelling. Participants will record and report details of solicited and unsolicited AEs and capture medication details via an electronic Diary (eDiary).

**Adverse Events (Dose-Escalation Phase (Sentinel Groups 1, 2 and 3) and Expanded Phase (Cohorts 1a to 3c)**		
**Event Type**	**Duration**	**Description**
Solicited reactions	7-days post vaccination (Day 1 to Day 7, with Day 1 being the day of vaccination)	Local (vaccination site): Pain, Tenderness, Redness/Erythema and Induration/Swelling.<br>Systemic: Fever, Chills, Nausea, Vomiting, Muscle pain, Joint pain, Headache, Fatigue/Somnolence, Diarrhoea, Malaise
Unsolicited adverse events (AEs)	From the time of Informed Consent to Study Completion on Day 181	Spontaneously reported by participant/identified by Investigator/abnormal vital signs, physical examination findings/abnormal laboratory assessments/solicited reactions with an onset beyond 7 days following vaccination vital signs/ symptom directed examination/ Any other signs, symptoms
Immediate AEs	30 minutes post vaccination	Vital signs/ symptom directed examination/ Any other signs, symptoms
Serious Adverse Events	From the time of Informed Consent to Study Completion on Day 181	AEs meeting seriousness criterion(a)
Adverse Events of Special Interest	From the time of Informed Consent to Study Completion on Day 181	• Anaphylactic reactions<br>• Generalised convulsion<br>• Guillain-Barré Syndrome (GBS)<br>• Acute disseminated encephalomyelitis (ADEM)<br>• Thrombocytopaenia<br>• Vasculitides<br>• New-onset chronic medical conditions (NOCMC)<br>• Enhanced disease following immunisation<br>• Myocarditis, Pericarditis
Laboratory Abnormality of Special Interest	From the time of Informed Consent to Study Completion on Day 181	• Rheumatoid Factor titre of >40IU/mL
**Safety Laboratory Assessments**		
Dose-Escalation Phase<br>(Sentinel Groups 1, 2 and 3) and<br>Expanded Phase (Cohorts 1a to 3c)	At Screening, Day 8 (Visit 3) (Sentinel Group participants only), Day 29 (Visit 5) and Day 181 (Visit 7)	Blood chemistry, Blood haematology (complete blood count with differential), Urinalysis and presence of Rheumatoid

		Factor (IgM anti-Fc) and other anti-Fc antibodies (IgM, IgG and IgA anti-Fc). *Screening only*: hepatitis B surface antigen, hepatitis C virus antibody, Human Immunodeficiency Virus antibody types 1 and 2.

Immunogenicity Assessments

Immunogenicity assessments are applicable to all study participants. The assessments will include SARS-CoV-2 specific antibody binding assays, micro-neutralising and pseudo-virus neutralising assays and Multiplex bead ACE-2-RBD binding inhibition assays, at the time points specified below. In addition, cellular immune responses, memory B cell clonotype and clonotype analysis will be performed as specified below.

Dose-Escalation Phase (Sentinel Groups 1, 2 & 3) and Expanded Phase (Cohorts 1a to 3c):
Day 1 (baseline), Day 8, Day 29, day 91, and Day 181.

Cellular Immune Responses

Peripheral Blood Mononuclear Cell (PBMC) immune responses will be assessed by Whole Blood Stain, antibody secreting cells (ASC), CD4+/CD8+, T follicular helper (Tfh) and activation and cytokine assays (AIMS and ICS) on CD4/CD8 T cells, and ELISpot cytokine assays will be assessed at the following time points:

Dose-Escalation Phase (Sentinel Groups 1, 2 & 3) and Expanded Phase (Cohorts 1a to 3c):
Day 1 (baseline), Day 8 (Whole blood Stain), and Day 29 (AIMS/ICS and ELISpot assays).

Exploratory Assays

Memory B-cell (MBC) staining (Wuhan type versus variants of concern (VoCs), MBC Sequencing (single cell) and serum antibody clonotype analysis will be assessed in at least a subset of study participants at the following time points:

Dose-Escalation Phase (Sentinel Groups 1, 2 & 3) and Expanded Phase (Cohorts 1a to 3c):
MBC staining (WT vs VOC) will be assessed on Day 1, Day 29, Day 91 and Day 181. MBC Sequencing (single cell) and serum antibody clonotype analysis will be assessed on Day 1, Day 29 and Day 91.

Blood volumes for safety laboratory assessments, immunogenicity assessments and cellular immune responses will be detailed in the Laboratory Manual.

Statistical Methods

Sample Size:
This is a first-in-human dose-escalation single-dose Phase I study of the DoCo-Pro-RBD-1 + MF59® and MIPSCo-mRNA-RBD-1 vaccines.

The study is designed to evaluate initial safety and tolerability of a single dose of the two candidate vaccines at three different dose levels in 76 participants who have previously been vaccinated with 2 doses of *Comirnaty™* or *Vaxzevria™*, and boosted with either *Comirnaty™* or *Spikevax™* at least 3 months (90 days) prior to study vaccination.

Analysis Populations:
Safety Set: The Safety Population consists of all enrolled participants who were randomised and received one dose

study vaccine or placebo and will be analysed according to actual treatment received. Using this Safety Population, demographic and baseline data and safety endpoints will be presented during the planned early (Dose-Escalation and Expanded Phase participants) and final analysis.<br><br>**Immunogenicity Population:** The Immunogenicity Population consists of all enrolled participants who were randomised and received one dose of study vaccine or placebo and provided at least one valid immunogenicity response measured at least 7 days after one dose of study vaccine or placebo and will be analysed according to randomisation group. Using this Immunogenicity Population, immunogenicity endpoints will be presented during the planned early (Dose-Escalation and Expanded Phase participants) and final analysis.<br><br>**Per Protocol Population:** The Per Protocol Population consists of all participants included in the Immunogenicity Population who do not have any major protocol deviations affecting the immune response up to 6 months after their last dose of study vaccine or placebo. The definition of the Per Protocol Population will be finalised before database lock. Using this Per Protocol Population, immunogenicity endpoints will be presented during the final analysis.<br><br>**Statistical analysis:**<br>The statistical analysis will be descriptive. No hypotheses will be tested. Any inferential testing or models will be considered explorative in nature. Details of the analyses will be described in the statistical analysis plan.	
**Interim Analysis:**<br>After all participants of the Dose-Escalation Phase and the Expanded Phase of the study have completed Day 29 (i.e., 28 days after vaccination) an interim analysis of all available safety and immunogenicity data up to Day 29 + 1 will be performed. The interim clinical study report (CSR) will include all the safety and immunogenicity analyses as specified in Primary Endpoints 1 to 5 as well T-cell responses to SARS-CoV-2 RBD-derived peptide antigens; Specific T cell responses including activation, proliferation and cytokine production, in response to RBD peptide antigens through 29 days post vaccination.<br><br>The investigator and study participants will remain blinded throughout the study. The final CSR will include the analyses for all study data after all study participants in the Dose-Escalation Phase of the study and the Expanded Phase of the study have completed all planned study visits and database lock and unblinding have occurred.	
**Data Safety Monitoring Board (DSMB)**	An independent DSMB will be appointed to review safety and immunogenicity data during the study. The DSMB will review the post-vaccination safety data after all participants in Sentinel Group 1 (Lower Dose Group) in the Dose-Escalation Phase of the study (as per the schema in Figure 1) and all participants in Cohorts 1a to 1f in the Expanded Phase of the study have completed at least 7 days of safety follow-up post vaccination. If a positive recommendation is made, dosing will commence in Sentinel Group 2 (Intermediate Dose Group). If no Stopping Rules are met following a review of the 7-day post-vaccination safety data in Sentinel Group 2 participants, dosing will commence in Cohorts 2a to 2c in the Expanded Phase of the study. The DSMB will review the 7-day post-vaccination safety data in Sentinel Group 2 participants and all participants in Cohorts 2a to 2c in the Expanded Phase of the study and provide a recommendation as to whether dosing in Sentinel Group 3 (Higher Dose Group) in the Dose-Escalation Phase of the study should proceed.<br><br>The DSMB will also convene if any of the Stopping Rules are met at any time during the conduct of the study.<br><br>The DSMB meeting conduct and time points will be described in a DSMB charter.

**Study Sites**	(1) Vaccine and Immunisation Research Group, Doherty Institute for Infection and Immunity, University of Melbourne; and<br>(2) VIDS, Royal Melbourne Hospital.
**Study Duration**	Estimated start date (First Participant First Visit): April 2022<br>Projected stop date (Last Participant Last Visit): March 2023 Estimated duration: 11 Months

1.2. Study Schema

1.2.1. Treatment Groups

Lower Dose (then Intermediate and Higher Dose)

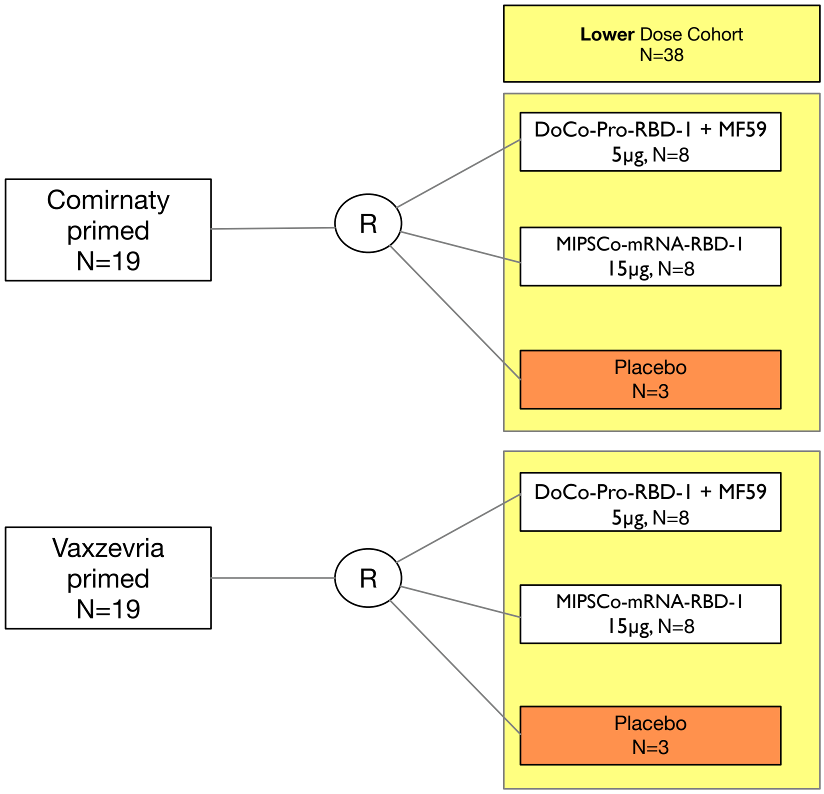

Intermediate and Higher Doses

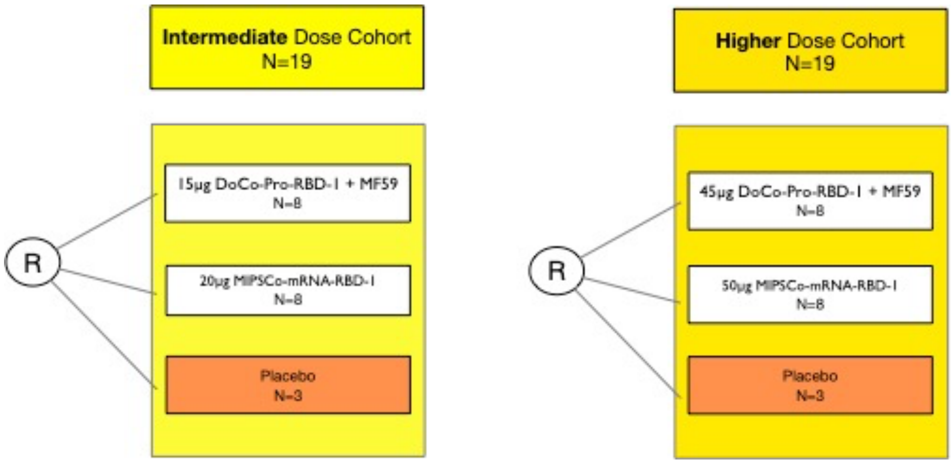

1.2.2.Study Overview

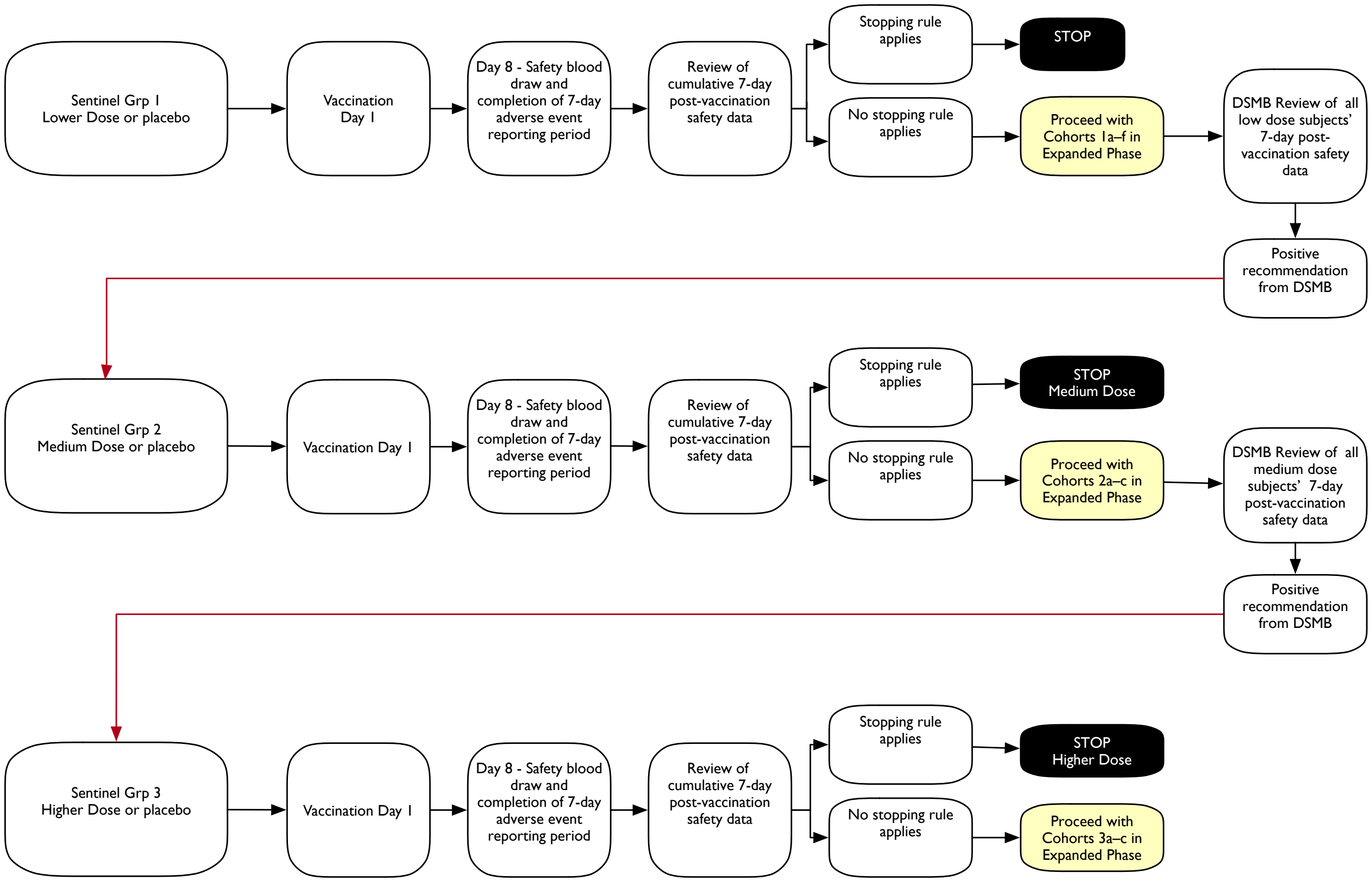

1.2.3. Randomisation Overview

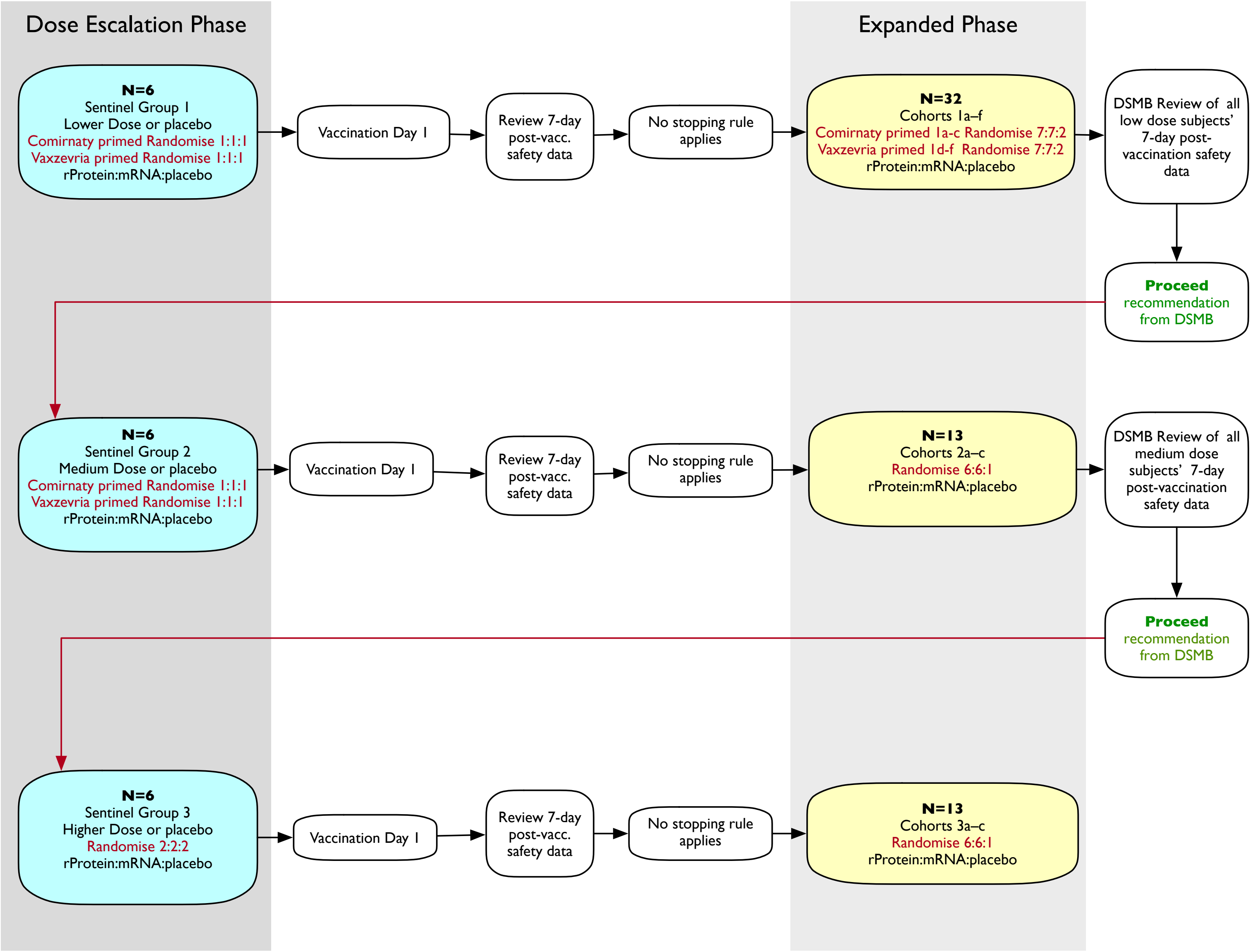

1.3. Schedule of Activities: Dose-Escalation and Expanded Phases

	V1	V2	V3	V4	V5	V6	V7
**Description**	Screening<br>Day –14 to -1	Day 1<br>Vaccination<br>(Vax)	Day 8+2<br>7 days<br>post Vax	Day 15+2<br>14 days<br>post Vax	Day 29+2<br>28 days<br>post Vax	Day 91±3<br>3 months<br>post Vax	Day 181±3<br>6 months<br>post Vax
Informed consent, history, exam	x						
Eligibility	x	x[A, B]					
Pregnancy test – urine (WOCBP), urinalysis	x[C]						
Enrolment and randomisation		x					
Serology & Immunogenicity blood draw		[D, E, F]<br>(50 mL)	[D, E, F]<br>(10 mL)		[D, E, F]<br>(55 mL)	[D, E, F]<br>(50 mL)	[D, F]<br>(20 mL)
Safety bloods	[G]<br>(15 mL)		[H]<br>(10 mL)		[G]<br>(10 mL)		[G]<br>(10 mL)
SARS-CoV-2 Test (RAT or PCR)	Based on symptoms and/or contacts						
Vaccine administration		[I,J]					
eDiary card completion		x	x	x	x		
Assessment of AEs	x	x	x	x[K]	x		
Assessment of SAEs, AESIs and LASIs	x	x	x	x	x	x	x[L]

Footnotes to Section 1.3.1 above:

[A] Reconfirmation of eligibility
[B] Review and update (if necessary) medical history and ongoing medications prior to randomisation
[C] A urine pregnancy test will be performed in women of childbearing potential (WOCBP) at Screening. A repeat test may be performed on Day 1 at the Investigator's discretion. Urine examination includes urine dipstick. If urine dipstick results are abnormal (i.e. contain more than trace abnormalities) a complete urine routine and microscopy should be performed.
[D] An immunogenicity blood sample will be collected prior to study vaccine administration, after reassessment of eligibility. Immunogenicity assessments on Day 1 (baseline), Day 8, Day 29, day 91, and Day 181 will include SARS-CoV-2 specific Binding ELISA and Multiplex ELISA assays, micro-neutralising and pseudo-virus neutralising antibodies and Multiplex binding inhibition assays. Anti-Fc assays (by ELISA) and clonotype analysis will be performed on Day 1, Day 29 and Day 91 samples.
[E] Cellular immune responses will be assessed at Day 1(baseline), Day 8 (Whole blood Stain), and Day 29 and Day 91(AIMS/ICS Assay/ELISpot assay).
[F] Exploratory assays: Memory B-cell staining (ancestral versus variant of concern (VoC)) will be assessed on Day 1, Day 8, Day 29, Day 91 and Day 181. MBC Sequencing (single cell) and serum antibody clonotype analysis will be assessed in a subset of samples based on other antibody assays on Day 1, Day 29 and Day 91.
[G] A safety blood sample will be collected for laboratory assessments including complete Haematology, Biochemistry (including Liver Function Tests, Renal Function Tests, serum electrolytes) and Presence of Rheumatoid Factor. *Screening only assessments*: Hepatitis B surface antigen, hepatitis C virus antibody, Human Immunodeficiency Virus antibody types 1 and 2.
[H] Sentinel Group Participants only: A safety blood sample will be collected for laboratory assessments including complete Haematology, Biochemistry (including Liver Function Tests, Renal Function Tests, serum electrolytes) and Presence of Rheumatoid Factor
[I] Vital signs will be assessed prior to vaccination and at the end of the 30-minute period after vaccination
[J] Participants in Sentinel Groups 1, 2 & 3 will be observed for a minimum of 4 hours post-vaccination
[K] Telephone follow-up visit will occur on Day 15 (Visit 4)
[L] All participants will be scheduled to attend V7 for blood collection and 6-month post-vaccination safety follow-up. However, if any participant discontinues the study early, they will be followed up for safety and contacted with a 6-month post-vaccination safety follow-up telephone call to identify the occurrence of any SAEs or AESIs that had not yet been reported. If discontinuation occurs at a scheduled study visit, the participant will be provided with a memory aid instead of access to an e-Diary for SAE follow-up (Including AESIs) until the 6-month post-vaccination safety follow-up call.

2. INTRODUCTION

2.1. Study Rationale

The purpose of the Dose-Escalation Phase and the Expanded Phase of this study is to evaluate the safety and immunogenicity of single doses of DoCo-Pro-RBD-1 + MF59® and MIPSCo-mRNA-RBD-1 in healthy adult participants 18 to 64 years of age who have previously received 2 doses of *Comirnaty™* or *Vaxzevria™* and a third booster dose of either *Comirnaty™* or *Spikevax™*. Participants will be randomised to receive DoCo-Pro-RBD-1 + MF59® at three dose levels (5µg, 15µg, or 45µg), MIPSCo-mRNA-RBD-1 vaccine at three dose levels (10µg, 20µg or 50µg) or placebo (normal saline). Participants will receive one dose of DoCo-Pro-RBD-1 + MF59®, MIPSCo-mRNA-RBD-1 or placebo and will remain on study for safety and data collection and immunogenicity assessments until day 181.

2.2. Background

The COVID-19 pandemic caused by SARS-CoV-2 has infected over 253 million people, claimed more than 5.1 million lives (Johns Hopkins University, 2021) and adversely affected economies globally. The best chance to overcome this pandemic is with safe and effective vaccines. There are currently over 200 vaccine candidates in active development and more than 40 are in clinical trials. These include whole inactivated virus, viral vectors expressing spike (S), isolated S proteins, including some with structure stabilisation or multimer scaffolds, mRNA, and DNA vaccines. While this is impressive and encouraging, there are known adverse reactions, albeit relatively rare, associated with S-based vaccines, including thrombocytopaenia with thrombosis syndrome (TSS) with the Astra Zeneca vaccine, and myocarditis with the mRNA vaccines (Pfizer and Moderna) and moreover, with the exception of the locally manufactured Astra Zeneca vaccine, Australia is reliant on vaccines manufactured overseas.

As the pandemic evolves, mutations are emerging, several of which have appeared in Australia, that are less susceptible to immunity to the original SARS-CoV-2 strain used for all current vaccines. The solution to this problem may lie in a vaccine that can be rapidly tuned, providing optimal immunity to the variants that are currently circulating.

The SARS-CoV-2 receptor-binding domain (RBD) is located at the tip of the Spike and is responsible for the virus adhering to human ACE-2 and virus infection (Wang, 2020). Moreover, this small region of the virus is the target of over 90% of neutralising antibodies following SARS-CoV-2 infection (Piccoli, 2020). For this reason, the Doherty Institute for Infection and Immunity and the Monash Institute of Pharmaceutical Sciences have developed two RBD-focused vaccine candidates that are efficient to produce and can be rapidly modified to incorporate distinct or multiple RBD variants arising in circulation.

DoCo-Pro-RBD-1 is based on the SARS-CoV-2 beta variant receptor-binding domain, generated as a human IgG1 Fc-domain fusion protein, to facilitate production and purification and multimeric presentation to the immune system while also engaging FcR+ antigen-presenting cells for enhanced immunological priming. This vaccine induces high titres of RBD-specific antibodies, including high neutralising antibody titres, in mice following a prime and boost regimen. Immunity induced by this vaccine is durable and protects against virus challenge in a mouse model of SARS-CoV-2 infection, even 100 days following the boost. Because the most immune-evasive variant to date was the beta variant, we used the RBD from this variant as the basis for our vaccine. It has been shown to induce strong neutralising antibody immunity against this variant of concern, and protects mice against infection with this strain, while still retaining its potential to neutralise the original ancestral (Wuhan) strain virus.

MIPSCo-mRNA-RBD-1 is an mRNA vaccine encoding the SARS-CoV-2 beta variant receptor-binding domain (RBD) attached to the transmembrane and cytoplasmic domains of the spike protein. Injection of the product results in expression of a membrane-anchored RBD, rather than whole spike protein. In common with approved mRNA vaccines, the mRNA is formulated in lipid nanoparticles using four lipids all of which have been used in FDA-approved products. This vaccine induces high titres of RBD-specific antibodies, including high neutralising antibody titres, in mice following a prime and boost regimen. Immunity induced by this vaccine is durable and protects against virus challenge in a mouse model of SARS-CoV-2 infection. Because the most immune-evasive variant to date was the beta variant, we used the RBD from this variant as the basis for our vaccine. It has been shown to induce strong neutralising antibody immunity against this variant of concern, while still retaining its potential to neutralise the original ancestral (Wuhan) strain virus.

The study will be conducted in two overlapping parts (see Figures 2 and 3): The Dose-Escalation Phase with the Expanded Phase is a dose-escalation study of a single booster dose of SARS-CoV-2 beta variant DoCo-Pro-RBD-1 + MF59® at three dose levels (5µg, 15µg, or 45µg), SARS-CoV-2 RBD-mRNA vaccine at three dose levels (10µg, 20µg or 50µg) or placebo (normal saline) in participants previously vaccinated with a 2-dose schedule of *Comirnaty*™ or *Vaxzevria*™ and a third booster dose of either *Comirnaty™* or *Spikevax™*.

2.3. Risk/Benefit Assessment

The following section outlines the known potential risks and benefits of participation in this study.

2.3.1. Known Potential Risks

Due to the lack of clinical experience in humans, there is currently limited information available regarding the safety of DoCo-Pro-RBD-1 + MF59® and mRNA MIPSCo-mRNA-RBD-1 vaccines.

However, data from the nonclinical safety evaluation of DoCo-Pro-RBD-1 + MF59® and mRNA MIPSCo-mRNA-RBD-1, together with adverse events common reported with currently licensed SARS-CoV-2 vaccines and vaccines containing the MF59® adjuvant, which have been administered to millions of people worldwide, suggest that intramuscular injection (IM) of the study vaccines may precipitate transient and self-limiting local adverse events, which typically include injection site pain, swelling and redness, fatigue. Systemic adverse events from vaccination may include headache, myalgia, arthralgia and fever or chills.

The adverse events described above may or may not be related to DoCo-Pro-RBD-1 + MF59® or mRNA MIPSCo-mRNA-RBD-1 and adverse effects may exist which have not yet been detected. A sentinel dosing strategy will be followed to mitigate the risk to study participants. Ongoing safety monitoring of 7-day post-vaccination safety data by a group comprising the Sponsor’s Medical Monitor, the Co-ordinating PI, the PI at the Royal Melbourne Hospital, VIDS (for Sentinel Group 1 participants only) and at least two of the five Investigators. A review of all 7-day post-vaccination data for the lower and intermediate dose levels of the study vaccines will conducted by the data safety Monitoring Board (DSMB). Prospective study stopping rules, as detailed in Section 4.6 will also be applied in the event of safety concerns which warrant further evaluation.

Acute allergic reactions, such as an anaphylactic event, may occur with any vaccine administration. These are serious, but rare, occurrences with an onset that is typically quite rapid. To minimize the risk of hypersensitivity reactions, all participants will be

observed at the study site for at least 30 minutes post vaccination with appropriate medical treatment readily available, if required. Sentinel participants in the Dose-Escalation phase of the study will be observed for 4 hours post-vaccination.

Syncope (fainting) can occur following or prior to vaccination or a blood draw as a psychogenic or vasovagal response to the needle injection.

2.3.2. Known Potential Benefits

Participants are not expected to directly benefit from their participation in this study, but they will contribute to the development of COVID-19 vaccines in general. It is, however, theoretically possible that vaccination with the active study vaccines may induce a protective immune response against SARS-CoV-2 if participants are subsequently exposed to the virus.

Individual participants may also gain information about their general health because of screening assessments, which will include a physical examination, blood and urine tests. Participants found to have a previously undiagnosed condition, which, in the clinical judgment of the Investigator or the Investigator’s delegate, requires further medical attention will, with their consent, be referred for further investigation and treatment.

3. OBJECTIVES AND ENDPOINTS

Table 1 Primary Objectives and Endpoints

**Objectives**	**Endpoints**
**Dose-Escalation Phase and Expanded Phase**	
To evaluate the safety and immunogenicity of a single dose of 3 dose levels of DoCo-Pro-RBD-1 + MF59® or MIPSCo-mRNA-RBD-1, in healthy adults aged 18 to 64 years inclusive, and previously vaccinated with a 2-dose schedule of *Comirnaty*™ or *Vaxzevria*™ and a third booster dose of either *Comirnaty*™ or *Spikevax*™.	1. Serious adverse events (SAEs), medically attended adverse events (MAAEs) and any adverse events (AEs) leading to study withdrawal at any time during the study [through to completion at Day 181].<br>2. SAEs from Day 1 to 29 (28 days post vaccination).<br>3. Solicited local and systemic reactogenicity AEs within 7 days after vaccination (Day 1) by severity score, duration and peak intensity.<br>4. Unsolicited AEs from Day 1 to Day 29 (28 days post vaccination).<br>5. Percentage of participants subjects who achieve a boost response (defined as a 4-fold increase in SARS-CoV-2 neutralising or RBD-specific Ab titres from baseline) by Day 29 (28 days after vaccination).

Table 2 Secondary Objectives and Endpoints

Objectives	Endpoints
**Dose-Escalation Phase and Expanded Phase**	
1. To assess the safety of a single-dose schedule of 3 dose levels of intramuscular administration of each candidate vaccine throughout the study period, within 28 days after vaccination and for 6 months after vaccination.<br>2. To evaluate the immunogenicity as measured by ELISA to detect Ab against the SARS-CoV-2 RBD protein, and by neutralising assay against SARS-CoV-2 virus, at baseline (Day 1), Day 29 (28 days after vaccination), and 3, and 6 months after vaccination using 3 dose levels of each candidate vaccine.<br>3. To assess antigen-specific T-cell response by flow cytometry assays and enzyme-linked immunospot (ELISpot) assays at baseline (Day 1), Day 8 (7 days after vaccination), Day 29 (28 days after vaccination) and M3, and M6 (3, and 6 months after vaccination) after using 3 dose levels of each candidate vaccine.<br>4. To assess the balance of T-cell immunity based on cytokine production at each timepoint where T cells are assessed as per secondary objective 3.	1. MAAEs from Day 1 to 6 months after vaccination using MeDRA classification, severity score and relatedness.<br>2. Percentage of participants who seroconvert by 3-, and 6-months post vaccination. The seroconversion rate will be calculated based on test results reaching quantifiable Ab level after vaccination.<br>3. Geometric mean titre of RBD-specific Ab and nAb titres through 6 months post vaccination, including change from baseline in Ab titres (threshold of ≥ 4-fold increase from baseline titre).<br>4. Geometric mean fold rise (threshold of ≥ 4-fold increase from baseline titre) of RBD-specific Ab and nAb titres through 6 months post vaccination.<br>5. T-cell responses to SARS-CoV-2 RBD-derived peptide antigens; Specific T cell responses including activation, proliferation, and cytokine production, in response to RBD peptide antigens through 6 months post vaccination.

Table 3 Exploratory Objectives and Endpoints

**Objectives**	**Endpoints**
**Dose-Escalation Phase and Expanded Phase**	
1. To compare antibody reactivity against SARS-CoV-2 beta variant to the ancestral SARS-CoV-2 strain.<br>2. To assess antibody reactivity against other SARS-CoV-2 Variants of Concern (VOCs).<br>3. To assess dominant antibody clonotypes against SARS-CoV-2 RBD.<br>4. To compare the impact of RBD boosts following *Vaxzevria*™ and *Cominarty*™, to RBD prime to promote variant-specific responses.	1. Geometric mean titre of SARS-CoV-2 variant-RBD-specific Ab and nAb titres through 6 months post vaccination, including change from baseline in SARS-CoV-2 variant reactive Ab titres.<br>2. Memory B cell (MBC) frequency, specific for RBD including VOC reactive B cells in response to boosting (Dose-Escalation Phase and Expanded Phase of the study (Day 1, Day 8, Day 29, Day 91, Day 181<br>3. MBC clonotype analysis and mass spectrometry analysis on serum antibodies to align with results from MBC single cell clonotype analysis on a subset of samples based on other antibody analysis (Dose-Escalation Phase and Expanded Phase of the study (Day 1, Day 29).

4. STUDY DESIGN

4.1. Overall Design

This is a randomised, double-blind, placebo-controlled, dose-escalation, first-in-human study to assess the safety, reactogenicity and immunogenicity of SARS-CoV-2 beta variant DoCo-Pro-RBD-1 + MF59® and MIPSCo-mRNA-RBD-1 vaccine at three dose levels, administered intramuscularly (IM) as a single booster dose in healthy adults previously vaccinated with two doses of *Cominarty*™ (BNT162b2 [mRNA]) or *Vaxzevria™* (ChAdOx1-S) COVID-19 and a third booster dose of either *Comirnaty™* or *Spikevax™* vaccines. The study will comprise a Dose-Escalation Phase and an Expanded Phase.

The study vaccines, DoCo-Pro-RBD-1 + MF59®, MIPSCo-mRNA-RBD-1 or placebo (normal saline) will be administered IM in the deltoid region of the upper arm.

The study will enroll healthy adults aged 18 to 64 years of age inclusive. Participants in both the Dose-Escalation Phase and Expanded Phase the Lower Dose Cohort of the study will be stratified by prior primary course COVID-19 vaccination with *Cominarty*™ or *Vaxzevria*™, but in the Intermediate and Higher Dose Cohorts, stratification will not be carried out.

4.2. Scientific Rationale for Study Design

The double blind, randomised, placebo-controlled, dose-escalation design of this safety will allow the safety and immunogenicity of DoCo-Pro-RBD-1 + MF59® and MIPSCo-mRNA-RBD-1 to be evaluated with minimal bias and minimized risk to study participants. Placebo groups have been included to act as a control for reactogenicity, safety and immunogenicity assessments.

A healthy adult population, free from significant illness, aged 18 to 64 years inclusive, is an appropriate population to assess to safety and immunogenicity of DoCo-Pro-RBD-1 + MF59® and MIPSCo-mRNA-RBD-1.

Sentinel dosing groups have been included in the study design together with a requirement for reviews of post-vaccination safety data by a Data Safety Monitoring Board (DSMB) in order to mitigate risks to study participants as per the requirements of applicable regulatory guidelines for study designs in healthy volunteers.

4.3. Justification for Dose

4.3.1.Dose Selection: Recombinant protein vaccine

The human therapeutic doses of 5, 15 and 45µg were determined based on:

- The results from a phase I trial with a similar RBD-Fc vaccine with alum adjuvant, which used three different doses of 10, 25 and 50µg per injection where they found strong responses each dose tested but moderately stronger responses at the 50µg dose in 18-59 year olds (Liao et al., 2021); In this study, they report that they did not observe any grade 3 or above adverse events at any dose across the phase I trial. In a subsequent phase II trial of this vaccine, 720 individuals were immunised with 1 or 2 doses of this vaccine at a range of 10, 25 or 50µg and only 1 grade 3 adverse event (2 dose 25ug) was considered to be associated with vaccination.
- Findings from a phase I trial with a SARS-CoV-2 whole spike vaccine with MF59® as an adjuvant, which used three different doses of 5, 15 and 45µg. They found that all three doses were effective but the 15µg dose group was optimal.
- Our own observations in mice suggesting that a low dose of our vaccine (0.3-3µg) can provide comparable or better immunity than a higher dose (10µg).
- The data from our toxicology study demonstrated that a high dose of 50µg DoCo-Pro-RBD-1 ADP + MF59®. vaccine was well tolerated with the toxicology report summarising that no

observed adverse effect levels were identified. This 50µg dose was selected for toxicology as it exceeds the highest actual dose we propose to administer to humans (45µg), without any allometric scaling to account for the much smaller size of rats compared to humans. [For further detail, refer to the DoCo-Pro-RBD-1 Antigen Drug Product Investigator Brochure, Version 1.1 dated 1 February 2022].

4.3.2. Dose Selection: mRNA vaccine

The clinical starting, intermediate and high doses of MIPSCo-mRNA-RBD-1 of 10µg, 20µg and 50µg respectively, was justified by comparing dose-response data obtained in preclinical studies of MIPSCo-mRNA-RBD-1 with published studies using the Moderna *Spikevax*™ product. The dose range required to obtain adequate to strong neutralising antibody response was 1µg-5µg for both vaccines. The clinical dose of *Spikevax*™ is 100µg and the booster dose is 50µg, it can be concluded that scaling from mouse to human can be achieved by multiplying the effective dose in mice by a factor of ten. It is expected that an effective dose of MIPSCo-mRNA-RBD-1 will lie within the range 10µg-50µg, hence the choice of a 10µg starting dose". [Refer to the MIPSCo-mRNA-RBD-1 Antigen Drug Product Investigator Brochure, Version 1.1. dated 1 February 2022].

4.3.3. Dose Selection: MF59®

MF59® has been used in influenza vaccines since 1997. It has been given to over 120 million people worldwide and has an excellent safety record. The 0.25 mL dose of MF59® to be administered in this study is the dose recommended by Seqirus Inc., the manufacturer of MF59®. The same dose of MF59® is used in Fluad® (Seqirus' MF59® adjuvanted influenza vaccine) and was also used in the recent phase I study of the University of Queensland/Seqirus candidate COVID-19 vaccine.

4.4. End of Study Definition

A participant will be considered to have completed the study if they have completed the Day 181 Visit 7 (Dose-Escalation Phase and Expanded Phase).

A participant is considered to have completed the study treatment (receipt of study vaccines/placebo) if they have received one vaccination on Day 1 (Dose-Escalation Phase and Expanded Phase).

The end of the study will be defined as the date of the last visit of the last participant on the study or the last scheduled study procedure shown in the Schedule of activities in Section 1.3 for the last participant in the study.

4.5. Dose-Escalation Criteria

The planned doses of DoCo-Pro-RBD-1 + MF59® and MIPSCo-mRNA-RBD-1 to be administered in this study are listed in Section 6.1.1.

4.5.1. Opening Rules: Expanded Phase

The dosing of participants in Groups 1a to 3c of the Expanded Phase will commence as follows:

- A sentinel cohort of 6 participants randomised to the lower dose of the study vaccines (2 to receive 5µg DoCo-Pro-RBD-1 + MF59®, 2 to receive 10µg MIPSCo-mRNA-RBD-1 and 2 to receive placebo) (Sentinel Group 1) will be vaccinated first at the Royal Melbourne Hospital Clinical Trials Centre.

- These participants will be observed for reactogenicity for 7 days after dosing. Their post-vaccination safety data and Day 8 clinical laboratory data will be reviewed by a group comprising the Sponsor's Medical Monitor, the Co-ordinating PI, the PI at Royal Melbourne Hospital, VIDS (for Sentinel Group 1 participants only) and at least two of the four clinician Investigators against the Study Stopping Rules. Further enrollment into the study will be paused until completion of the review. If no significant clinical or laboratory abnormalities (as defined in Section 4.6.2) are observed, a further 32 participants in Cohorts 1a to 1f in the Expanded Phase will be vaccinated at the Doherty Institute with the 5µg of DoCo-Pro-RBD-1 + MF59®, 10µg of MIPSCo-mRNA-RBD-1 or placebo in a ratio of 7:7:2.
- On completion of Day 8 visits by all study participants in Cohorts 1a to 1f, the Data Monitoring Safety Board (DSMB) will review the 7-day post-vaccination safety data together with the 7-day post-vaccination safety data of the Sentinel Group 1 participants. If the data are found to be acceptable, sentinel dosing with 15µg DoCo-Pro-RBD-1 + MF59®, 20µg MIPSCo-mRNA-RBD-1 or placebo will commence at the Doherty Institute in a further 6 participants in Sentinel Group 2, the intermediate dose group.
- In the Sentinel Group 2 participants, further enrollment into the study will be paused to allow a review of the 7-day safety data post vaccination and Day 8 clinical laboratory data by a group comprising the Sponsor's Medical Monitor, the Co-ordinating PI, and at least two of the four clinician Investigators. If no Stopping Rules are met, a further 13 participants in Cohorts 2a to 2c in the Expanded Phase of the study will be vaccinated at the Doherty Institute with the 15µg of DoCo-Pro-RBD-1 + MF59®, 20µg of MIPSCo-mRNA-RBD-1 or placebo in a ratio of 6:6:1.
- On completion of Day 8 visits by all study participants in Cohorts 2a to 2c, the Data Monitoring Safety Board (DSMB) will review the 7-day post-vaccination safety data together with the 7-day post-vaccination data of the Sentinel Group 2 participants. If the data are found to be acceptable, sentinel dosing with 45µg DoCo-Pro-RBD-1 + MF59®, 50µg MIPSCo-mRNA-RBD-1 or placebo will commence at the Doherty Institute in the final 6 participants in Sentinel Group 3, the higher dose group.
- Further enrollment into the study will be paused to allow a review of the 7-day safety data post vaccination and Day 8 clinical laboratory data by a group comprising the Sponsor's Medical Monitor, the Co-ordinating PI, and at least two other clinician Investigators. If no Stopping Rules are met, a further 13 participants in Cohorts 3a to 3c in the Expanded Phase of the study will be vaccinated at the Doherty Institute with the 45µg of DoCo-Pro-RBD-1 + MF59®, 50µg of MIPSCo-mRNA-RBD-1 or placebo in a ratio of 6:6:1.

.

4.6. Study Stopping Criteria

4.6.1. Stopping Criteria for Individual Participants

Participants will be withdrawn from this study for the following reasons:

- The Investigator or their delegate decide that it would be in the participant's best interest to do so. If the decision is made because the participant experiences a severe

AE appropriate clinical measures must be taken and the Sponsor notified within 24 hours.

- The participant is unwilling to continue in the study.
- Protocol non-compliance, including the use of prohibited medications or blood products.
- An intercurrent illness that prevents further participation in the study.
- Changes in the participant's general health that, in the Investigator's opinion, would pose a health risk to the participant if they were to continue on study or could interfere with the interpretation of the study results.
- Study closure or termination by the Sponsor.
- If a female participant becomes pregnant.
- New information about the study vaccines becomes available which suggests that continued participation in the study may not be in the participant's best interests.

4.6.2. Dose-escalation Stopping /Pausing Rules

Enrolment into the study and/or further vaccination will be paused in the event that any of the Stopping/Pausing Rules listed below are identified. The Stopping/Pausing Rules are considered to have been met only if the events described occur post administration of the study vaccines:

Rule 1: One or more participant(s) experiences an SAE assessed as *at least possibly related* to investigational product (IP) by the Investigator (applies to both Study Phases);

Rule 2: Four (or more) participants experience the same or similar Grade 3 AE assessed as *at least possibly related* to investigational product (applies to the Expanded Study Phase);

Rule 3: One (or more) participants experiences an AESI assessed as *at least possibly related* to investigational product (applies to both Study Phases);
Rule 4: Four (or more) participants develop a LASI assessed as *at least possibly related* to investigational product (applies to the Expanded Study Phase); and

Rule 5: The Investigator, using their discretion, requests that the study to be placed on hold and a DSMB meeting be held for any single event or combination of multiple events which, in the Investigator's clinical judgment, jeopardise the safety of study participants or the reliability of the data.

The study may also be paused or stopped if:

- Required by the Sponsor; or
- Required by the relevant authority; or
- Required by the Human Research Ethics Committee (HREC).

If any of the Study Stopping Rules are met, further enrollment and vaccination of participants will be paused pending a review of the safety data by the Data Safety Monitoring Board.

The Investigator must immediately inform the Sponsor, CRO(s) and the HREC if any of the Study Stopping Rules are met.

4.6.3. Participants with COVID-19-Like Illness During the Study

Enrolled study participants who experience any symptoms of indicative of COVID-19 illness during this study, must undergo testing in accordance with Victorian Government public health directives, to confirm the presence or absence of SARS-CoV-2 infection. If possible, those participants who test positive (either by RAT or PCR tests) should also be offered virus whole genome sequencing, performed by standard procedures, to be conducted at no expense to them through the Peter Doherty Institute. If logistically feasible and acceptable to the participant, nasal swab and specimen collection will be provided by study staff in the home of the participant under full PPE protection for staff. If this occurs, participants will be requested to provide their standard personal identifiers as required by the Victorian Government for PCR virus genomic sequencing. Participants will be provided the results of this testing as soon as it is available to study staff.

Any PCR or RAT-confirmed positive result for SARS-CoV-2 in a participant during the study will be counted as an event. Participants who test positive for SARS-CoV-2 at any time during the study will not be replaced by another participant.

If a participant tests positive for SARS-CoV-2 prior to the Day 8 study visit they will be asked to complete their 7-day post-vaccination safety assessments. They may continue in the study and proceed with planned study visits, at the investigators' discretion, but reattendance at the study site must be consistent with applicable public health guidelines.

.

5. STUDY POPULATION

Prospective approval of protocol deviations to recruitment and enrollment criteria, known as protocol waivers or exemptions, will not be permitted.

5.1. Inclusion Criteria

To be eligible for this study, participants must meet ALL of the following inclusion criteria:

1. Adults 18 to 64 years of age inclusive at screening previously vaccinated with a 2-dose schedule of *Cominarty*™ or *Vaxzevria*™.

2. ≥ 3 months (90 days) since receipt of a booster dose of either *Comirnaty*™ or *Spikevax*™.
3. Be in good health as determined by medical history, physical examination, vital signs, and clinical laboratory assessments with no clinically significant abnormalities as judged by the Investigator at screening and randomisation. Vital signs must be within medically acceptable ranges prior to the first vaccination.

4. Participants of childbearing potential (defined as any participant who has experienced menarche and who is NOT surgically sterile [ie, hysterectomy, bilateral tubal ligation, or bilateral oophorectomy] or postmenopausal [defined as amenorrhea of at least 12 consecutive months]) must agree to be heterosexually inactive from at least 28 days prior to enrollment and through the end of the study OR agree to consistently use a medically acceptable method of contraception listed below from at least 28 days prior to enrollment and through the end of the study:

    a. Condoms (male or female); Diaphragm; Cervical cap; Intrauterine device; Oral or patch contraceptives; Norplant®, Depo-Provera®, or another regulatory approved contraceptive method; Abstinence, as a form of contraception, is acceptable if in line with the participant's lifestyle.

    NOTE: Periodic abstinence (e.g., calendar, ovulation, symptom-thermal, post-ovulation methods) and withdrawal method (coitus interruptus) are not acceptable forms of contraception.

5. Agrees to not participate in any other SARS-CoV-2 prevention or treatment trials for the duration of the study.

6. Willing and able to give informed consent prior to study enrollment and to comply with all study procedures.

5.2. Exclusion Criteria

Potential study participants will be excluded from the study if ANY of the following criteria apply:

1. History of test-confirmed (by PCR, rapid antigen test (RAT) to SARS-CoV-2) COVID-19 infection within 3 months (90 days) prior to randomisation.

2. Participants with a BMI > 35kg/m^2.

3. Positive result for rheumatoid factor (RF) at Screening.

4. Positive test at Screening for human immunodeficiency virus (Types 1 or 2) antibody, hepatitis B surface antigen or hepatitis C virus antibody.

5. Clinical laboratory test results not within normal range and judged to be clinically relevant abnormalities by the investigator.

6. History of prior cardiac inflammatory disease (endocarditis, myocarditis or pericarditis).

7. History of demyelinating disease or Guillain Barré syndrome.

8. Fever (non-axillary temperature >37.5°C) or any other symptoms of infection that have not completely resolved within 3 days prior to Randomisation (Day 1).

9. Presence of current active viral infection or bacterial infection, at Screening or Randomisation (Day 1), which is determined by the Investigator to be of clinical significance.

10. Participation in research involving receipt of an investigational product (drug/biologic/device) within 90 days prior to the first study vaccination or an intention to participate in another clinical trial at any time during the conduct of this study.

11. Received any other vaccine within 30 days prior to the first study vaccination, other than licensed influenza vaccine which can be administered up to 14 days prior to randomization.

12. Any known allergies to products contained in the investigational products.

13. Any history of anaphylaxis to any prior vaccine, food, drug, toxin or other exposure.

14. Autoimmune or immunodeficiency disease/condition (iatrogenic or congenital) requiring ongoing immunomodulatory therapy.
    NOTE: Stable endocrine disorders (e.g., thyroiditis, pancreatitis), including stable diabetes mellitus with no history of diabetic ketoacidosis) are NOT excluded.

15. Chronic administration (defined as > 14 continuous days) of immunosuppressant, systemic glucocorticoids, or other immune-modifying drugs within 90 days prior to first study vaccination.
    NOTE: An immunosuppressant dose of glucocorticoid is defined as a systemic dose ≥ 10mg of prednisone per day or equivalent. The use of topical or intranasal glucocorticoids is permitted. Topical tacrolimus and ocular cyclosporin are permitted.

16. Received immunoglobulin, blood-derived products, or immunosuppressant drugs or donation of blood/blood products within 90 days prior to vaccination or planned receipt or donation during the study period.

17. Thrombocytopaenia, contraindicating intramuscular vaccination, based on the Investigator's judgment.

18. Bleeding disorder, or receipt of anticoagulants in the 3 weeks preceding inclusion, contraindicating intramuscular vaccination based on the Investigator's judgement.

19. Active cancer (malignancy) on therapy within one year prior to first study vaccination (with the exception of adequately treated non-melanomatous skin carcinoma or lentigo malignancy and uterine cervical carcinoma in situ without evidence of disease, at the discretion of the Investigator).

20. Participants who are breastfeeding, pregnant or who plan to become pregnant prior to the end of study.

21. Suspected or known history of alcohol abuse or drug addiction within 2 years prior to the first study vaccine dose that, in the opinion of the Investigator, might interfere with protocol compliance.

22. Have a tattoo/scar/birthmark or any other skin condition affecting the deltoid area that may, in the Investigator's opinion, interfere with injection site assessments.

23. Any other condition that, in the opinion of the Investigator, would pose a health risk to the participant if enrolled or could interfere with evaluation of the trial vaccine or interpretation of study results (including neurologic or psychiatric conditions likely to impair the quality of safety reporting).

24. Study team member or immediate family member of any study team member (inclusive of Sponsor, CRO, and study site personnel involved in the conduct or planning of the study).

25. Aboriginal and Torres Strait Islander person aged 50 years or older.

5.3. Lifestyle Considerations

No lifestyle restrictions other those mentioned in Section 5.2 and those mentioned below will be applied during this study.

5.3.1. Meals and Dietary Restrictions

No meals or dietary restrictions will be applied during the study.

5.3.2. Caffeine, Alcohol and Tobacco

No specific restrictions regarding the consumption of caffeine or alcohol will be imposed during the study. However, participants will be discouraged from consuming alcohol on the day(s) of vaccination.

5.3.3. Physical Activity

No specific restrictions regarding strenuous activity will be imposed during the study.

5.4. Screen Failures

Screen failures are defined as participants who consent to participate in the study but are not subsequently randomised to study vaccine(s)/placebo. To comply with regulatory requirements, the following information regarding individuals who are screen failures will be collected: demographics, screen failure details, eligibility criteria and any SAE(s).

Participants may be re-screened once at the discretion of the PI (or designee) in consultation with the sponsor's Medical Monitor. There may be instances in which an eligible participant is unable to be vaccinated with 14 days of their screening period. In these circumstances, the participant will be required to sign a new Participant Informed Consent Form (PICF) and all screening procedures will be repeated. Re-screened participants will be assigned a new participant identification number.

6. STUDY PRODUCT

The term 'study product' refers to the investigational study vaccines which will be evaluated as part of the study objectives; DoCo-Pro-RBD-1 + MF59®and MIPSCo-mRNA-RBD-1 and the placebo as well as the investigational study vaccine components, which include the following:

- DoCo-Pro-RBD-1 Antigen Drug Product (CSL Innovation Pty Ltd; 0.18 mg/mL; 0.4 mL volume in a single use vial)
- MF59® adjuvant (Seqirus Inc.; 0.25 mL volume in pre-filled, single use 1mL syringe)
- DoCo-Pro-RBD-1 Antigen Diluent (10mM Citrate formulation buffer (pH 6.5)) (CSIRO; 0.4 mL volume in a single use vial)
- MIPSCo-mRNA-RBD-1 Antigen Drug Product (IDT Australia Limited; 0.1 mg/mL; 2mL volume in a single use vial)
- MIPSCo-mRNA-RBD-1 Antigen Diluent (Sterile isotonic tromethamine/sucrose buffer solution (pH 7.2-7.4)) (IDT Australia Limited; 2 mL volume in a single use vial).
- Sodium Chloride Injection BP 0.9% (Placebo) (Pfizer Australia Pty Ltd; 10 mL single use ampoule)

6.1. Study Product and Administration

6.1.1.Study Product Formulations

Study Product	Formulation	Dose Strength	Dose (IM)	Dosage Form
DoCo-Pro-RBD-1 + MF59®	10 µg/mL + 50% v/v<br>30 µg/mL + 50% v/v<br>90 µg/mL + 50% v/v	5 µg<br>15 µg<br>45 µg	0.5 mL<br>0.5 mL<br>0.5 mL	Suspension for injection
MIPSCo-mRNA-RBD-1 (in lipid nanoparticle)	20 µg/mL<br>(0.307 mg/mL total lipids)<br>40 µg/mL<br>(0.614 mg/mL total lipids)<br>100 µg/mL<br>(1.535 mg/mL total lipids)	10 µg<br><br>20 µg<br><br>50 µg	0.5 mL<br><br>0.5 mL<br><br>0.5 mL	Suspension for injection
Sodium Chloride Injection BP 0.9% (Placebo)	90 mg/10mL	0.9%	0.5 mL	Solution for injection

6.1.2.Dosing and Administration

Only eligible participants enrolled in the study will receive study vaccine and only authorised study personnel will prepare or administer study vaccines.

Study vaccine administration should be preceded by a review of the participant's medical history and a physical examination.

Standard immunisation practices are to be observed and care should be taken to administer the study vaccines intramuscularly. DoCo-Pro-RBD-1 + MF59®, MIPSCo-mRNA-RBD-1, DoCo-Pro-RBD-1 and placebo should not be administered intravenously, subcutaneously, or by intradermal route under any circumstances.

Each vaccine dose of 0.5 mL will be administered IM into the deltoid region of the upper arm. The needle should be introduced at a 90° angle through the skin and advanced deep into the muscle mass. The study vaccine should be injected, and the needle should be withdrawn when all the contents are delivered.

Vaccination should be performed in a setting where emergency resuscitation equipment is available. Study personnel and supervision must be readily available in case of rare anaphylactic or any severe allergic reactions following administration of the study vaccine.

6.1.3. Dose Escalation

Please refer to Section 1.2.2 and Section 4.5.1.

6.1.4. Dose Modification

No dose modifications will be made.

6.2. Study Product Preparation/Handing/Storage/Accountability

6.2.1. Study Product Acquisition and Accountability

All study product will be shipped to The Royal Melbourne Hospital Pharmacy upon request and approval from the Sponsor.

The PI/designee is responsible for study product distribution and disposition and has ultimate responsibility for study product accountability. The PI may delegate this responsibility to the study site's clinical trial pharmacist and/or authorised study personnel.

The study site's clinical trial pharmacist and/or designated study personnel will be responsible for maintaining complete records and documentation of study product receipt, accountability, dispensation, storage conditions and final disposition. Study product accountability records and dispensing logs should include, but are not limited to the following: study protocol number; participant ID; allocated study product; dosage level; lot number or other identification number; expiration or retest date; date of receipt of the study product; quantity received; quantity dispensed as dose level per participant; balance of study product currently available; disposition of study product if not dispensed to a study participant (e.g. disposed/destroyed), date of study vaccine preparation; time of vaccine preparation; expiration of vaccine preparation and volume/amount of vaccine drawn up for administration.

The time of study vaccine administration to the participant will be recorded in the eCRF. All study product(s), including the dose of DoCo-Pro-RBD-1 + MF59® or MIPSCo-mRNA-RBD-1, DoCo-Pro-RBD-1 antigen diluent and MIPSCo-mRNA-RBD-1 antigen diluent and vial admixtures, whether administered or not, must be documented on the appropriate study product accountability record or dispensing log. The Sponsor's delegated unblinded monitoring staff will verify the study product accountability records and dispensing logs in accordance with the site monitoring plan.

The following must be retained for product accountability:

- Used and unused DoCo-Pro-RBD-1 and MIPSCo-mRNA-RBD-1antigen drug product vials
- Used and used DoCo-Pro-RBD-1 antigen diluent and MIPSCo-mRNA-RBD-1 antigen diluent vials
- Used and unused MF59® syringes
- Used and used normal saline ampoules
- Used mixing vials
- DoCo-Pro-RBD-1, MIPSCo-mRNA-RBD-1, MF59®, DoCo-Pro-RBD-1 antigen diluent, MIPSCo-mRNA-RBD-1 antigen diluent outer cartons/containers

All used supplies noted above must be sequestered from the unused supplies and retained until study conclusion when a final reconciliation of shipped, used and used study product will be undertaken by the study monitor(s). Any discrepancies noted must be investigated, resolved and documented prior to the return or destruction of unused study product, as directed by the Sponsor.

If study product is destroyed at the site, it must be destroyed as biohazardous waste, in accordance with the relevant standard operating procedure (SOP) and documented in the study files. A certificate of destruction will be provided to the Sponsor.

6.2.2. Study Product Packaging and Labelling

The Sponsor will ensure that all labelling of study product complies with regulatory requirements.

Details regarding study product labelling and packaging are provided in the Pharmacy Manual.

6.2.3. Study Product Storage and Stability

The following study products should be stored at 2 to 8°C:

- DoCo-Pro-RBD-1 Antigen Drug Product
- MF59® adjuvant
- DoCo-Pro-RBD-1 Antigen Diluent
- MIPSCo-mRNA-RBD-1 Antigen Diluent
- Normal saline (0.9%) (placebo).

MIPSCo-mRNA-RBD-1 Antigen Drug Product should be stored at -80°C or below.

Further details of the storage of study product will be provided in the Pharmacy Manual.

The PI or designee must confirm appropriate temperature conditions have been maintained during transit for all study product received and any discrepancies are reported and resolved before use of the study vaccines.

Only participants enrolled in the study may receive a study vaccine and only authorised study site personnel may supply or administer the study vaccine.

All study product must be stored in a secure, environmentally controlled and monitored area in accordance with the labelled storage conditions with access limited to the PI and authorised study site personnel.

The expiry date or retest date for each of the study products will be provided on the product labelling.

Stability studies for the DoCo-Pro-RBD-1 Antigen Drug Product and MIPSCo-mRNA-RBD-1 Antigen Drug Product are ongoing.

6.2.4. Study Vaccine Preparation

Please refer to the protocol-specific Pharmacy Manual for a detailed overview of study vaccine preparation. Vaccine preparation will be performed by an unblinded pharmacist on the day of vaccine administration to the participant.

Only eligible participants enrolled in the study will receive study vaccine and only authorised study personnel will administer study vaccine.

6.3. Measures to Minimise Bias: Randomisation and Blinding

6.3.1. Randomisation and Blinding

Computer-generated randomisation lists will be supplied by an independent statistician based at the University of Melbourne (Melbourne, Australia) to the Director of Biometrics, Southern Star Research (SSR) for uploading into the IBM Clinical Development randomisation system prior to study commencement. IBM Clinical Development is the

electronic data capture (EDC) platform licensed by SSR and is compliant with ICH CGP and regulatory guidelines including HIPAA and 21CRF Part 11. The randomisation lists will be stored securely in IBM Clinical Development database system.

All study participants will be randomised, using an Interactive Web Response System (IWRS) as per the randomisation lists. Before the study is initiated, sites will receive the log-in information and appropriate training for the IWRS will be provided.

For the Lower Dose Cohort, randomisation in the Dose-Escalation and Expanded Phase will occur on Day 1 in a stepwise dose-escalation design, stratified by prior COVID-19 vaccination with *Comirnaty*™ or *Vaxzevria*™. Participants previously vaccinated with a booster dose of *Cominarty*™ or *Spikevax*™ who provide informed consent and meet all other eligibility criteria will be randomly assigned by block randomisation to receive DoCo-Pro-RBD-1 + MF59® or MIPSCo-mRNA-RBD-1 or placebo on Day 1 in a 1:1:1 ratio in the Dose-Escalation Phase and 7:7:2 ratio in the Expanded Phase cohorts 1a to 1f as outlined in Figure 1.2.3.

For the Intermediate and Higher Dose Cohorts, randomisation in the Dose-Escalation and Expanded Phase will occur on Day 1 in a stepwise dose-escalation design, but not stratified by prior COVID-19 vaccination with *Comirnaty*™ or *Vaxzevria*™. Participants previously vaccinated with a booster dose of *Cominarty*™ or *Spikevax*™ who provide informed consent and meet all other eligibility criteria will be randomly assigned by block randomisation to receive DoCo-Pro-RBD-1 + MF59® or MIPSCo-mRNA-RBD-1 or placebo on Day 1 in a 1:1:1 ratio in the Dose-Escalation Phase and 6:6:1 ratio in the Expanded Phase in each of these two dose level groups (intermediate, higher) as outlined in Figure 1.2.3.

Participants will be assigned a unique study identifier at randomisation which cannot be reassigned.

6.3.2. Blinding and Masking Procedures

The Dose-Escalation and Expanded Phase of this study will be placebo controlled. Study participants, study site personnel, the Sponsor, the Sponsor's delegated data management vendor and biostatisticians responsible for analysis and reporting of data will be blinded to study vaccine allocation.

An unblinded dosing team, not involved with the study participant's evaluation will prepare and administer the study vaccine/placebo doses. Study vaccine administration will be performed in a closed area to ensure that other blinded site personnel are not unblinded.

Study participants, study site personnel, the Sponsor, the Sponsor's delegated data management vendor and biostatisticians responsible for analysis and reporting of data will be blinded to the investigational study vaccine allocation. An unblinded dosing team, not involved with the study participant's evaluation will prepare and administer the study vaccine doses. Due to the visual differences between the investigational study vaccines, study vaccine administration will be performed in a closed area to ensure that other blinded site personnel are not unblinded.

In the event of an emergency, the study vaccine allocation code can be broken. The Sponsor should be informed as soon as possible of a code break.

6.3.3. Emergency Unblinding

The Principal Investigator (PI) will be responsible for determining if unblinding of a participant's study vaccine allocation is warranted where the participant experiences a medical emergency. The PI must make every attempt to contact the Sponsor/Sponsor's designee prior to unblinding a participant's study vaccine allocation unless this could delay emergency treatment of the participant. If a participant's study vaccine allocation is unblinded, the Sponsor must be notified within 24 hours after breaking the blind. The date and reason for unblinding must be recorded in the source documentation. The PI must provide a rationale for the necessity of unblinding based on the expectation that knowledge of the participant's study vaccine allocation would have a meaningful impact on the participant's medical care.

Unblinding will be facilitated through IWRS. The details and documentation regarding unblinding will be described in the relevant manual.

6.4. Study Vaccine Compliance

Study vaccine allocation, timing and mode of administration cannot be changed. Any departures from the intended regimen will be recorded in the eCRF.

Each dose of study vaccine will be administered by an unblinded member of the study team that is qualified to administer the study vaccines. The study participant identification will be reconfirmed at the time of vaccination. The date, time and location of each study vaccine administration will be entered into the eCRF.

6.5. Concomitant Medications

Any medication (including over-the-counter or prescription medicines, vitamins, supplements and/or herbal supplement) that a participant is receiving at the time of enrollment and/or receives during the study will be recorded in the eCRF together with:

- Reason for use.
- Dates of administration including start and end dates.
- Dose and frequency of dosing.

The use of an excluded medication/therapy/vaccination is a protocol violation and must be recorded in the eCRF.

Prohibited medications/vaccinations include (please also refer to Section 5.2):

- Other investigational products (drugs/biologics/devices) within 90 days prior to Day 1 or during the study period.
- Chronic administration of corticosteroids (defined as >14 continuous days) of immunosuppressant (defined as ≥10mg of prednisone per day or equivalent), systemic corticosteroids, or other immune-modifying drugs within 90 days prior to Day 1.
- Immunoglobulin, blood-derived products, or immunosuppressant drugs or donation of blood products within 90 days prior to vaccination or planned receipt during the study period.
- Vaccination with a licensed influenza vaccine is permitted but not within 14 days prior to study vaccination, and not within 14 days following study vaccination. Participants should not receive any other vaccine within 30 days prior to receipt of study vaccination, and not until at least 29 days following study vaccination and not before the scheduled blood draw at Visit 5.

6.6. Dose Modification

No dose modifications are planned for this study.

6.7. Availability of Study Vaccines After the End of the Study

The Sponsor will not provide access to study vaccines, or any additional medical care, to study participants after they have completed the study.

7. DISCONTINUATION OF STUDY TREATMENT AND PARTICIPANT DISCONTINUATION /WITHDRAWAL

7.1. Discontinuation of Study Treatment

The primary reasons which would lead to a discontinuation of study treatment on Day 1 in the Dose-Escalation or Expanded Phase of the study are as follows and must be documented in the eCRF:

- Withdrawal of consent by participant.
- Participant lost to follow-up.
- Participant is a protocol violator (did not meet inclusion criteria or met an exclusion criterion and was inadvertently enrolled).
- Positive test for SARS-CoV-2 infection less than 3 months (90 days) prior to randomisation at the Day 1 Visit.
- Termination of the study by the Sponsor.
- Other (specify).

The Study Stopping/Pausing Rules are detailed in Section 4.6.3. The procedures to be followed if a participant has a COVID-19 Like Illness whilst on study are also described in Section 4.6.3.

7.2. Participant Discontinuation/Withdrawal from the Study

A participant may be withdrawn from the study at any time at their own request or they may be withdrawn at any time at the discretion of the Investigator (or Investigator's delegate) for reasons of safety or failure to comply with the study requirements.

If a participant withdraws their consent for their continued participation in the study, every effort must be made to complete study assessments as detailed in Section 1.3 as thoroughly as possible up until the date of withdrawal. A participant who is withdrawn or withdraws from the study may undergo safety assessments if deemed appropriate in the Investigator's judgement.

The Sponsor will retain and continue to use all data and biological samples collected before the time of withdrawal of consent in data analyses.

A participant who withdraws their consent may request that any biological samples collected but not tested be destroyed. The Investigator must comply with the request and document the destruction of the samples in the participant's source data.

Refer to Section 1.3 for the data which must be collected following study withdrawal.

Participants who withdraw from the study will not be replaced.

Participants who are withdrawn from the study by the Investigator because of an AE or SAE must be followed up until resolution of the event. Participants withdrawn from the study due to an AE or SAE will not be replaced.

The predominant reason for study withdrawal by a participant must be documented in the eCRF. The Investigator will document the reason for withdrawal as follows:

- Adverse event(s)
- Protocol violation
- Withdrawal of consent (Investigator to provide reason for withdrawal if disclosed by the participant)
- Lost to follow-up

- Termination of the study by the Sponsor, or
- Other (specify).

7.3. Lost to Follow-Up

In the event a participant fails to attend the study site for a scheduled study visit the following actions must be taken:

- Study personnel must attempt to contact the participant and ascertain whether they wish to continue their participation in the study. If they are agreeable to continuing, the missed visit should be rescheduled as soon as practicable and the importance of adhering to the study visit schedule reiterated.
- Before a participant is deemed to be lost to follow-up, the investigator or Investigator's delegate must make three (3) attempts to contact the participant, including forwarding a registered letter to the participant's last known mailing address. Each attempt to contact the participant must be documented in the participant's source data.
- If the participant cannot be contacted after three documented attempts, they will be considered lost to follow-up.

8. STUDY ASSSESMENTS AND PROCEDURES

Study assessments for the **Dose-Escalation Phase**, the **Expanded Phase** and their timings are summarized in Section 1.3. Further details of the procedures to be undertaken at each study visit for Dose-Escalation Phase participants, and Expanded Phase participants will be provided in the Manual of Procedures (MOP).

Protocol waivers or exemptions are not permitted unless necessary for the management of immediate safety concerns.

Immediate safety concerns should be discussed with the Sponsor or the Sponsor's Medical Monitor, immediately upon occurrence or awareness to determine if the participant(s) should discontinue study vaccine/placebo.

Adherence to the study design requirements, including those specified in Section 1.3, is essential and required for study conduct.

8.1. Screening Procedures

All Screening evaluations must be completed and reviewed to confirm that potential study participants meet all eligibility criteria and do not meet any exclusion criteria. The Investigator will maintain a Screening Log to record details of all participants screened and to confirm eligibility or record reasons for Screening failure, as applicable.

8.2. Safety Assessments

Planned timepoints for all safety assessments are provided in Section 1.3.1.

8.2.1. Physical Examinations

A physical examination will be performed only at Screening and may include the examination of the following: general appearance, weight and height, abdomen, head and neck, eyes, ears, nose, throat, chest/respiratory, heart/cardiovascular, gastrointestinal/liver, musculoskeletal/extremities, dermatological/skin, thyroid, lymph nodes and neurological. Any findings made will be recorded in the eCRF. At other study visits, only a brief symptom-directed physical examination will be performed at the Investigator's or the Investigator delegate's discretion.

If the Investigator determines that the participant's health on the day of vaccination temporarily precludes vaccination, the visit will be rescheduled at the Investigator's discretion.

Any abnormality observed during the physical examination will managed in accordance with local medical practice or by referral to an appropriate healthcare provider.

8.2.2. Vital Signs

Vital sign assessments will be performed prior to dosing on Day 1 for participants in both the Dose-Escalation Phase and Expanded Phase of the study. At all other study visits, the assessments will be performed when the participant attends the study site.

The assessments will include the following resting vital signs and must be recorded in the eCRF:

- Systolic/diastolic blood pressure.

- Respiratory rate.
- Body temperature.
- Pulse rate after at least 5 minutes rest in a seated position.

8.2.3. Clinical Safety Laboratory Assessments

Blood for haematology and biochemical and serology analysis and urine for urinalysis will be collected from all study participants at the time points specified in Section 1.3.1. Urine pregnancy testing will be performed in women of childbearing potential at the Screening Visit and before each vaccination.

Refer to Appendix 1 for a complete list of the clinical laboratory assessments to be performed during the study.

Haematology, biochemistry, serology and urine assessments will be performed at Melbourne Health Shared Pathology Service as per local practice using standardized and validated procedures.

The Investigator will review the laboratory report, document this review, and record any clinically relevant changes occurring during the study in the AE section of the eCRF. The laboratory report must be filed with the source documents.

Clinically significant abnormal laboratory findings are those which are not associated with an underlying disease, unless judged by the investigator to be more severe than expected for the participant's condition. Abnormal laboratory findings will be graded according to the criteria listed in Appendix 2.

All clinical laboratory tests with values considered to be clinically significant abnormal during participation in the study or within 30 days of the study vaccine should be repeated until the value(s) return to normal or baseline or are no longer considered to be significantly abnormal by the investigator or Medical Monitor. If such values do not return to normal/baseline within a period judged reasonable by the Investigator, the aetiology should be identified, and the Sponsor notified.

Refer to Section 8.3.4 for the clinical laboratory abnormality which qualifies as a Laboratory Abnormality of Special Interest (LASI).

All protocol-required laboratory safety assessments, as defined in Appendix 1, must be conducted in accordance with the Laboratory Manual and the Schedule of Activities detailed in Section 1.3.1 and Section 3.1.2.

8.2.4. Interpretation of Screening Assessments

Screened participants will be excluded if a laboratory result falls into the range of Grade 2 or greater OR is a Grade 1 value that is deemed clinically significant in the opinion of the Investigator.

8.3. Adverse Events and Serious Adverse Events

8.3.1. Definition of Adverse Event (AE)

An AE is any untoward medical occurrence in a participant, including an abnormal laboratory finding, symptom, or disease (new or exacerbated) temporally associated with the use of study vaccine/placebo, irrespective of whether it is considered related to the study vaccine.

8.3.1.1. Solicited Adverse Events

Solicited adverse events are pre-defined local (vaccination site) and systemic AEs that occur more frequently or are known to be associated with immunisation and are therefore actively monitored as potential indicators of vaccine reactogenicity. The Investigator will assess the causality of all local and systemic AEs.

The following solicited AEs will be monitored in all participants for a period of 7 days following vaccination:

Table 4 List of Solicited Adverse Events

Local Solicited Adverse Events	Systemic Solicited Adverse Events
• Pain at the vaccination site<br>• Tenderness<br>• Erythema/Redness<br>• Induration/Swelling	• Fever<br>• Chills<br>• Nausea<br>• Vomiting<br>• Muscle pain<br>• Joint pain<br>• Headache<br>• Fatigue/Somnolence<br>• Diarrhoea<br>• Malaise (general discomfort)

Any AE predefined as a solicited AE, which is identified within the first 7 days post vaccination and continues beyond Day 8 will be remain a solicited AE until resolution.

The severity of solicited AEs will be graded as MILD (Grade 1), MODERATE (Grade 2) or SEVERE (Grade 3) based on the following criteria:

Table 5 Severity Grading for Solicited Local and Systemic Adverse Events

	**Mild (Grade 1)**	**Moderate (Grade 2)**	**Severe (Grade 3)**
**Solicited Local Adverse Events**			
Pain at vaccination site	Does not interfere with daily activity	Repeated use of non-narcotic pain reliever >24 hours or interferes with daily activity	Any use of narcotic pain reliever or prevents daily activity
Tenderness	Mild discomfort to touch	Discomfort with movement	Significant discomfort at rest
Erythema/Redness	2.5 - 5 cm	5.1 - 10 cm	>10 cm
Induration/Swelling	2.5 - 5 cm and does not interfere with daily activity	5.1 - 10 cm or interferes with daily activity	>10cm or prevents daily activity
**Solicited Systemic Adverse Events**			
Fever **°C** (Oral)	38.0 – 38.4	38.5 – 38.9	39.0-40.0
Chills	Present but does not interfere with daily activity	Interferes with daily activity	Prevents daily activity
Nausea	Nausea present but not interfering with daily activities	Nausea present leading to decreased oral intake	Nausea present leading to minimal to no oral intake
Vomiting	No interference with activity or 1-2 episodes in 24 hours	Some interference with activity or >2 episodes in 24 hours	Prevents daily activity, ≥4 episodes in 24 hours, or 2 or more episodes per day prolonged on several days
Muscle pain	Present but does not interfere with activity	Interferes with activity, or some use of non-narcotic pain reliever	Prevents daily activity; use of narcotic pain reliever
Joint pain	Present but does not interfere with activity	Interferes with activity, or some use of non-narcotic pain reliever	Prevents daily activity; use of narcotic pain reliever
Headache	Present but does not interfere with activity	Interferes with activity, or some use of non-narcotic pain reliever	Prevents daily activity; use of narcotic pain reliever
Fatigue/Somnolence	Present but does	Interferes with	Prevents daily

	not interfere with activity	activity	activity
Diarrhoea	2 - 3 loose stools or <400g /24 hours	4 - 5 loose stools or 400-800g /24 hours	6 or more watery stools or >800g / 24 hours or requires outpatient IV hydration
Malaise (General discomfort)	No interference with daily activity	Some interference with usual and social activity, no treatment	Significant, prevents usual daily and social activity or requires treatment

8.3.1.2. Unsolicited Adverse Events

An unsolicited AE is an AE that is not categorized as a 'solicited' AE in Section 8.3.1.1 above. Participants will be instructed to record unsolicited AEs in the eDiary from Day 1 to Day 29 (Dose-Escalation Phase and Expanded Phase participants).

Potential unsolicited AEs may be medically attended (MAAEs) (defined as symptoms or illnesses requiring hospitalisation, or emergency department visit, or visit to or by a health care provider) or are of concern to the participant. In the event of a MAAE, participants will be instructed to contact the study site as soon as possible to report the event(s). Detailed information about the MAAE will be collected by authorised study site personnel during the interview and will be documented in the participant's records.

Unsolicited AEs that are not medically attended or perceived to be of concern by participants will be collected by review of participant diaries at the next study visit.

The severity of unsolicited AEs will be graded based on the following criteria:

Table 6 Severity Grading for Unsolicited Adverse Events

Mild (Grade 1)	Events that are usually transient and may require only minimal or no treatment or therapeutic intervention and generally do not interfere with the participant's usual activities of daily living.
Moderate (Grade 2)	Events that are usually alleviated with additional specific therapeutic intervention. The event interferes with usual activities of daily living, causing discomfort but poses no significant or permanent risk of harm to the participant.
Severe (Grade 3)	Events that interrupt usual activities of daily living, or significant affects clinical status, or may require intensive therapeutic intervention. Severe events are usually incapacitating.

8.3.2. Definition of Serious Adverse Event (SAE)

A SAE is defined as any untoward medical occurrence in a study participant that:

- Results in death.
- Is life-threatening:

    - The term 'life-threatening' refers to an event in which the participant was at risk at the time of the event. It does not refer to an event, which hypothetically might have caused death, if it were more severe.
- Requires inpatient hospitalization or prolongation of an existing hospitalization:
    - Hospitalisation for elective treatment of a pre-existing condition that did not worsen from baseline is not considered an AE.
- Results in persistent disability /incapacity or substantial disruption of the participant's ability to conduct normal life functions.
- Is a congenital anomaly/birth defect.
- Is a medically important event:
    - Other important medical events which may not result in death, be life threatening, or require hospitalization may be considered an SAE when, based upon appropriate clinical judgment, they may jeopardise the participant and may require medical or surgical intervention to prevent one of the outcomes listed above.

8.3.3. Suspected Unexpected Serious Adverse Reaction (SUSAR)

A Suspected Unexpected Serious Adverse Event (SUSAR) is defined as adverse event that occurs in a study participant, which is assessed by the Sponsor and or Principal Investigator as being serious, unexpected, and having a reasonable possibility of a causal relationship to the investigational product or an adverse reaction that is both serious and expected.

8.3.4. Adverse Event of Special Interest (AESIs)

The following events, irrespective of their severity, are defined AESs for the purposes of this study:

- Anaphylactic reactions
- Generalised convulsion
- Guillain-Barré Syndrome (GBS)
- Acute disseminated encephomyelitis (ADEM)
- Thrombocytopenia
- Vasculitides
- New-onset chronic medical conditions (NOCMC)
- Enhanced disease following immunisation
- Myocarditis, Pericarditis.

The following is defined as a LASI:

- Rheumatoid Factor titre of >40IU/mL.

Once an AESI is diagnosed in a study participant, the Investigator or designate must record the AESI in the relevant eCRF page and report the AESI or LASI in an expedited manner, as detailed in Section 8.3.5.

Where enough evidence exists to make any of the above diagnoses, the AE must be reported as an AESI. Symptoms or signs which may or may not represent the above diagnoses, should be recorded and reported as AEs but not as an AESI until the definitive diagnosis has been made and alternative diagnoses have been eliminated or shown to be less likely.

8.3.5. Time period and Frequency for Adverse Event Assessment and Follow-Up

All AEs, SAEs and AESIs will be collected for the durations specified in the Schedule of Activities in Section 1.3.

Any medical occurrences that occur prior to administration of study vaccine/placebo but after the participant has provided informed consent will be recorded in the Medical History section of the eCRF and not as an AE. The exacerbation of a chronic or intermittent preexisting condition including an increase in frequency and/or intensity of the condition after administration of study vaccine/placebo will be considered an AE.

All SAES and AESIs must be recorded and reported to the Sponsor's Medical Monitor within 24 hours of the Investigator becoming aware of the SAE or AESI. The Investigator will submit any updated information to the Sponsor's Medical Monitor within 24 hours of receipt of the information.

8.3.6. Assessment of Adverse Event Causality and Outcome

The PI or qualified designee must use their clinical judgment to assess and determine the relationship between study vaccine and the occurrence of each AE or SAE. Alternative causes, such as underlying disease(s), concomitant therapy, and other risk factors as well as the temporal relationship of the evet to study vaccine administration must be considered and investigated.

The AE must be characterised as related or unrelated as follows:

- **Related:** The AE is known to occur with the study intervention, there is a reasonable possibility that the study intervention caused the AE, or there is a temporal relationship between the study intervention and the event. Reasonable possibility means that there is evidence to suggest a causal relationship between the study intervention and the AE.
- **Unrelated:** There is not a reasonable possibility that the administration of the study intervention caused the event, there is no temporal relationship between the study intervention and event onset, or an alternate aetiology has been established.

The PI or qualified designee will assess the outcome of all AEs recorded during the study as:

- Recovered/resolved
- Recovering/resolving
- Not yet recovered/not yet resolved
- Recovered with sequelae/resolved with sequelae or
- Fatal (applicable to SAEs only).

8.3.7. Reporting of Adverse Events

All AEs/SAEs/AESIs should be entered into the relevant eCRF pages. In addition, all SAEs and AESIs, including both serious and non-serious AESIs, should be reported to the Sponsor or Sponsor's designee using the **AESI/SAE Reporting Form within 24 hours of awareness via email**. Any follow-up information for an AESI or SAE case should also be reported within 24 hours of awareness.

In instances where a participant's medical records are requested, all participant identifiers, with the exception of the participant number, will be redacted on the copies of the medical records before submission.

Copies of all correspondence relating to the reporting of AESs/SAEs should be maintained in the Investigator site files.

8.3.8. eDiary

At the vaccination visit, participants will be trained in the use of an eDiary to record AEs. They will be instructed to measure and record oral body temperature any local/general AEs for 7 days after vaccination and unsolicited AEs from Day 1 to Day 29 (both Dose-Escalation Phase and Expanded Phase participants).

Participants will be instructed to contact the study site if they develop any signs or symptoms they perceive as serious.

The Investigator or authorised study personnel will review the completed Participant Diary during discussions with the participant at the study intervals detailed in the Schedule of Activities in Section 1.3.1.

8.3.9. Pregnancy

A pregnancy in a study participant is not defined as an AE or SAE. However, any pregnancy complication or elective termination of a pregnancy will be reported as an AE or SAE. A spontaneous abortion will be considered an SAE.

Any participant who becomes pregnant while participating in the study will discontinue the study vaccine where applicable.

Any pregnancy that occurs during study participation through to Day 181 for Dose-Escalation Phase and Expanded Phase participants will recorded in the eCRF and followed to outcome.

8.4. Treatment of Overdose

Not applicable in this study.

8.5. Pharmacokinetics (Not applicable)

Pharmacokinetic parameters will not be evaluated in this study.

8.6. Pharmacodynamics (Not applicable)

Pharmacodynamic parameters will not be evaluated in this study.

8.7. Genetics (Not applicable)

Genetics will not be evaluated in this study.

8.8. Immunogenicity

Details of blood sample collection, biological sample handling, and analysis will be presented in the Laboratory Manual.

All blood samples collected for immunological analysis will be transferred to the Department of Microbiology and Immunology at the University of Melbourne, Peter Doherty Institute where they will be tested in the various immunological assays outlined in this protocol.

An aliquot (less than 2 mL) of some of these serum samples will be forwarded to: (1) Flinders University, South Australia, for anti-Fc antibody analysis (Prof. Tom Gordon's laboratory); (2) the National Institutes of Health (NIH), National Institute of Allergy and Infectious Diseases (NIAID), Vaccine Immunology Program, Bethesda, Maryland, USA (Dr. Adrian McDermott's laboratory), for analysis of antibody binding and neutralising activity in comparison to serum samples from other COVID-19 vaccines currently in use or in clinical trials and 3) Duke-NUS Medical School in Singapore (Dr. Linfa Wang's laboratory), for analysis of antibody binding and neutralising activity against different variants of sarbecoviruses, and in comparison to serum samples from recipients of other COVID-19 vaccines currently in use or in clinical trials.

Repeat or unscheduled samples may be collected for safety reasons or if there are technical issues with the processing of samples.

8.8.1. Antibody Responses

Immunogenicity assessments are applicable to all study participants. The assessments will include SARS-CoV-2 specific binding ELISA and Multiplex bead ELISA assays, neutralising antibodies based on micro-neutralising and pseudo-virus neutralising assays and Multiplex bead ACE-2-RBD binding inhibition assays at the time points specified below:

- **Dose-Escalation Phase (Sentinel Groups 1, 2 & 3) & Expanded Phase (Cohorts 1a to 3c):**
  Day 1 (Baseline), Day 8, Day 29, day 91, and Day 181.

In addition, anti-Fc assays (by ELISA) and clonotype analysis by mass spectrometry will be performed on Day 1, Day 29 and Day 91 samples in the Dose-Escalation Phase and the Expanded Phase of the study.

8.8.2. T-cell Response

Peripheral Blood Mononuclear Cell (PBMC) immune responses will be assessed by Whole Blood Stain (ASC, CD4/CD8, T follicular helper (Tfh)), and AIMS/ICS/ELISpot assays for T cell activation and cytokine production in all study participants at the following time points:

- **Dose-Escalation Phase (Sentinel Groups 1, 2 & 3) & Expanded Phase (Cohorts 1a to 3c)**:
  Day 1(baseline), Day 8 (Whole blood Stain), and Day 29 and Day 91 (AIMS/ICS/ELISpot Assay).

8.8.3. Memory B cell Staining and Sequencing and Clonotype Analysis

Memory B-cell staining (Wuhan type versus variants of concern (VoCs) and MBC Sequencing (single cell) and serum antibody clonotype analysis will be assessed in all study participants at the following time points:

- **Dose-Escalation Phase (Sentinel Groups 1, 2 & 3) and Expanded Phase (Cohorts 1a to 3c)**:
  Memory B-Cell staining (WT vs VOC) will be assessed on Day 1, Day 8, Day 29, Day 91 and Day 181. MBC Sequencing (single cell) and serum antibody clonotype analysis will be assessed on a subset of samples based on antibody analysis on Day 1, Day 29 and Day 91.

9. STATISTICAL CONSIDERATIONS

9.1. Statistical Hypotheses

The statistical analysis will be descriptive, no hypotheses will be tested. Any inferential testing or models will be considered exploratory in nature.

9.2. Sample Size Determination

Since this is a first-in-human dose-escalation single-dose Phase I study of the DoCo-Pro-RBD-1 + MF59® and MIPSCo-mRNA-RBD-1 vaccines, the statistical analysis will not include formal hypothesis testing and therefore the sample size calculation is not based on statistical power.

The Dose-Escalation Phase and the Expanded Phase of the study are designed to evaluate initial safety of a single dose of the two candidate vaccines at three different dose levels in 76 participants who have previously been COVID-19 vaccinated with a 2-dose schedule of *Comirnaty™* or *Vaxzevria™* and boosted with either *Comirnaty™* or *Spikevax™* (mRNA-1273, Moderna). For the Lower Dose group, eight participants will be included in each dose-level of the two candidate vaccines within each priming vaccine group, and nine participants in the placebo group. For the intermediate and higher dose, eight participants will be included in each dose level of the two candidate vaccines and three participants in the placebo group. The probability of observing at least one event (e.g. solicited reaction, adverse event, or LASI) depends on the number of participants vaccinated and the underlying true event rate in the study population. Tables 7 and 8 describe the probability of observing 0, 1, or 4+ events in eight participants or 32 participants for a range of true event rates.

Table 7 Probability of observing events by vaccine dose-group* in the Dose-Escalation and Expanded Phase (n=8 participants)

True event rate	Probability (0/8)	Probability (1/8)	Probability (4+/8)
1%	92%	7%	0%
5%	66%	28%	0%
10%	43%	38%	0%
20%	17%	34%	6%
30%	6%	20%	19%

* 5µg, 15µg, or 45µg DoCo-Pro-RBD-1 + MF59®; 10µg, 20µg, or 50µg MIPSCo-mRNA-RBD-1

Table 8 Probability of observing events by vaccine group* in the Dose-Escalation and Expanded Phase (n=32 participants)

True event rate	Probability (0/32)	Probability (1/32)	Probability (4+/32)
1%	72%	23%	0%
5%	19%	33%	7%
10%	3%	12%	40%
20%	0%	1%	91%
30%	0%	0%	99%

* DoCo-Pro-RBD-1 + MF59® or MIPSCo-mRNA-RBD-1, across both priming vaccination groups.

9.3. Populations for Analyses

Participants will be considered enrolled once they have provided informed consent and their study eligibility has been confirmed. Three analysis populations will be used: the Safety Population, the Immunogenicity Population, and the Per Protocol Population. The populations will be defined as follows:

Table 9: Analysis Populations

Population	Description
Safety	All enrolled participants who were randomised and received one dose of study vaccine or placebo.<br><br>Participants will be analysed according to actual treatment received.<br><br>Using this Safety Population, demographic and baseline data and safety endpoints will be presented during the planned early (Dose-Escalation and Expanded Phase participants) and final analysis.
Immunogenicity	All enrolled participants who were randomised and received one dose of study vaccine or placebo and provided at least one valid immunogenicity response measured at least 7 days after one dose of study vaccine or placebo.<br><br>Participants will be analysed according to randomised group.<br><br>Using this Immunogenicity Population, immunogenicity endpoints will be presented during the planned early (Dose-Escalation and Expanded Phase participants) and final analysis.
Per Protocol	All participants included in the Immunogenicity Population who do not have any major protocol deviations affecting the immune response up to 6 months after vaccination.<br><br>The definition of the Per Protocol Population will be finalised before database lock.<br><br>Using this Per Protocol Population, immunogenicity endpoints will be presented during the final analysis.

9.4. Statistical Analyses

A detailed statistical analysis plan describing all analyses to be performed will be finalised before the database lock of the planned interim analysis and the final analysis.

For the Dose-Escalation and Expanded Phase of the study, data listings will be sorted by priming vaccine (*Comirnaty™* or *Vaxzevria™*) and dose group, participant identifier, and timepoint.

Data will be summarised as described in the sections below. Summaries will consist of descriptive statistics whereby continuous variables will be summarized using n (non-missing sample size), mean, standard deviation, median, minimum, and maximum and categorical variables using counts and percentages (based on the non-missing sample size) of observed category levels.
For the Dose-Escalation and Expanded Phase of the study, summaries will be presented by primer (*Comirnaty™* or *Vaxzevria™*) and dose group. The dose groups are defined as follows:
5µg (lower), 15µg (intermediate), and 45µg (higher) DoCo-Pro-RBD-1 + MF59®;
10µg (lower), 20µg (intermediate), and 50µg (higher) MIPSCo-mRNA-RBD-1;
placebo.

All confidence intervals (CIs) will be two-sided 95% CIs. Where applicable, CIs will be obtained within each dose group. Comparative analyses will consist of qualitatively describing the difference.

Missing data will not be imputed, and outliers will not be excluded.

9.4.1. Demographic and Baseline Data

Demographic and baseline characteristics will be summarised, including age, sex and ethnic origin.

9.4.2. Safety Analyses

Clinical safety laboratory data will be summarised at each scheduled time point, including changes from baseline. The number and percentage of participants experiencing any deviation from the laboratory parameter reference ranges listed in Appendix 2 will be tabulated.

Concomitant medication will be coded using WHODrug with the version most recent at the time of database lock. The number and percentage of participants with any concomitant medication will be summarised, including by anatomical main group (level 1) and pharmacological subgroup (level 3).

The number and percentage of participants experiencing any solicited reactions as well as the number of solicited reactions will be summarised for local and systemic reactions separately, including by event type (e.g. pain at vaccination site).

All unsolicited AEs will be coded using MedDRA with the version most recent at the time of database lock. The number and percentage of participants experiencing any AE, any vaccine-related AE, any severe (intensity) AE, any SAE, death, discontinuation due to AE, any severe (intensity) vaccine-related AE, any vaccine-related SAE, any immediate AE, and any AESI or LASI will be presented in a table. In addition, any AEEs will be tabulated by system organ class and preferred term, by severity (intensity), and by causality. For AEs occurring more than once for a participant, the maximal severity and strongest causal relationship to the study vaccines.

9.4.3. Immunogenicity Analyses

Immunogenicity markers will be summarised at each scheduled time point, including changes from baseline. Immunogenicity in terms of antibody titres and seroconversion will be summarised as detailed below.

Antibody titres will be logarithmically transformed (base 10) and presented visually with dotplots at each time point and spaghetti plots to visualise within-participant changes from baseline. Geometric mean titres (GMTs) of the SARS-CoV-2 specific neutralising antibodies and GMTs of anti-SARS-CoV-2 IgG antibodies (ELISA) along with their associated two-sided 95% CIs will be computed by exponentiation (power of 10) of the corresponding log-transformed means and 95% CI.

The Geometric Mean Fold Rise (GMFR; GMT post vaccination / GMT at baseline) will be provided with its two-sided 95% Cis, by exponentiation (power of 10) of the corresponding difference in means of log 10-transformed SARS-CoV-2 specific neutralising antibody titres (95% CI) within an individual participant between post vaccination and baseline assessments. The number and percentage of participants with a ≥ 2-fold increase (2-fold, 3-fold or 4-fold increase) in SARS-CoV-2 specific neutralising antibody titres from baseline will be presented, together with their two-sided 95% CIs based on the exact-binomial method by Clopper and Pearson.

The number and percentage of participants who seroconvert (defined as a 4-fold increase in SARS-CoV-2 neutralising or RBD-specific antibody titres from baseline) by Day 29 will be presented, together with their two-sided 95% CIs based on the exact-binomial method by Clopper and Pearson.

9.4.4. COVID-19 Cases During the Study

The number of participants with a COVID-19 like illness that undergo COVID-19 testing (by rapid antigen test or nasopharyngeal swab for viral PCR) to confirm the presence or absence of SARS-CoV-2 infection as well as the incidence of COVID-19 positive results will be individually listed and summarized using summary statistics for each vaccine group.

9.5. Interim Analyses

One interim analysis will be performed after all participants in the Dose-Escalation Phase and the Expanded Phase of the study have completed their Day 29 visit (i.e., 28 days after vaccination).

The safety data available at the time of this interim analysis will be summarized in a blinded fashion. All unblinded vaccine cohort summaries will be prepared by an independent unblinded statistician not associated with the study conduct or the development of the SAP.

The interim clinical study report (CSR) will include all the safety and immunogenicity analyses as specified in Primary Endpoints 1 to 5 as well T-cell responses to SARS-CoV-2 RBD-derived peptide antigens; Specific T cell responses including activation, proliferation, and cytokine production, in response to RBD peptide antigens through 29 days post vaccination.

The interim analysis will be performed for informative reasons only and will not affect continuation of the study. No statistical adjustment will be made for this interim analysis.

The Investigator and study participants will remain blinded throughout the study. The final CSR will include the analyses for all study data after all study participants in the Dose-Escalation Phase and the Expanded Phase of the study have completed all planned study visits and database lock and unblinding have occurred.

9.6. Data Safety Monitoring Board (DSMB)

An independent DSMB will be appointed to review safety and immunogenicity data during the study. The DSMB will review the post-vaccination safety data after all participants in Sentinel Group 1 (Lower Dose Group) in the Dose-Escalation Phase of the study (as per the schema in Figure 1) and all participants in Cohorts 1a to 1f in the Expanded Phase of the study have completed at least 7 days of safety follow-up post vaccination. If a positive recommendation is made, dosing will commence in Sentinel Group 2 (Intermediate Dose Group). If no Stopping Rules are met following a review of the 7-day post-vaccination safety data in Sentinel Group 2 participants, dosing will commence in Cohorts 2a to 2c in the Expanded Phase of the study. The DSMB will review the 7-day post-vaccination safety data in Sentinel Group 2 participants and all participants in Cohorts 2a to 2c in the Expanded Phase of the study and provide a recommendation as to whether dosing in the Sentinel Group 3 (higher Dose Group) in the Dose-Escalation Phase of the study should proceed.

The DSMB will also convene if any of the Stopping Rules are met at any time during the conduct of the study. The DSMB will review the study data and provide their opinion to the Sponsor as to whether there are safety concerns and if the study can continue as planned or if any changes should be implemented or if the study should be terminated. The Sponsor will carefully consider DSMB recommendations, assess the overall risk benefit ratio and reach a final decision regarding further continuation or termination of the study.

The composition, responsibilities, and procedures of the DSMB will be described in detail a DSMB charter.

No statistical adjustment will be made for the monitoring analyses conducted by the DSMB.

10. SUPPORTING DOCUMENTATION AND OPERATING CONSIDERATIONS

10.1. Regulatory, Ethical and Study Oversight Considerations

This study will be conducted in conformity with the principles of ICH GCP.

An NHMRC-certified Human Research Ethics Committee (HREC) will review and approve this protocol, associated informed consent documentation, recruitment materials participant-facing material, prior to the recruitment, screening, and enrollment of participants.

Any amendments to the protocol or informed consent documentation will be approved by the HREC before they are implemented. The PI will notify the HREC of deviations from the protocol and reportable SAEs in accordance with the HREC requirements.

10.1.1. Informed Consent Procedure

The Investigator or his/her representative will explain the nature of the study to the participant or his/her legally authorized representative (if applicable) and answer all questions regarding the study.

Study participants must be informed that their participation is voluntary. Participants or their legally authorized representative will be required to sign a Participant Informed Consent Form (PICF) that meets the requirements of ICG GCP Guidelines and the approving Human Research Ethics Committee (HREC).

The participant's source document must include a statement that written informed consent was obtained before the participant was entered into the study and the date the written consent was obtained. The authorized person obtaining the informed consent must also sign the PICF.

Participants must be re-consented to the most recent version of the PICF during their participation in the study. A copy of the PICF(s) must be provided to the participant or the participant's legally authorized representative.

A participant who is rescreened is not required to sign another PICF if the rescreening occurs within 14 days of their previous PICF signature date.

10.1.2. Study Termination and Closure

The Sponsor reserves the right to close the study site(s) or terminate the study at any time for any reason at the sole discretion of the Sponsor. Study sites will be closed upon study completion. A study site will be considered closed when all required documentation and study supplies have been collected and a study site closure visit has been conducted.

The investigator may initiate the closure of a study site(s) at any time, provided there is reasonable cause and sufficient notice is given in advance of the intended termination.

Reasons for the early closure of a study site by the Sponsor or Investigator may include but are not limited to:

- Failure of the Investigator to comply with the protocol, the requirements of the HREC, the Sponsor's procedures or GCP guidelines.
- Inadequate recruitment of participants by the Investigator.
- Discontinuation of further study vaccine development.

10.1.3. Confidentiality and Privacy

The PI must ensure that the privacy of study participants privacy is maintained and that all study records are kept confidential.

Each participant in this study will be assigned a unique identifier by the Sponsor. Any participant records or datasets which are transferred to the Sponsor, or their designee will contain the identifier only. Participant names or any information which could identify the participant will not be transferred.

Participants must be informed that their personal, study-related data will be used by the Sponsor in accordance with local data protection and privacy laws. Participants must also be informed that their medical records may be examined by Clinical Quality Assurance (QA) auditors or other authorised personnel appointed by the Sponsor, by appropriate HREC representatives and by inspectors from the Therapeutic Goods Administration (TGA) in order to verify the study data collected.

All documentation containing participant information, including but not limited to source documentation, must be stored in locked filing cabinets/cupboards at the study site with access limited to authorised study personnel to maintain confidentiality. All records that contain names or other personal identifiers, such as PICFs, must be stored separately from study records which are identified by a unique identifier.

10.1.4. Secondary Use of Stored Specimens and Data

By enrolling in this study all participants will be providing consent for their remaining blood samples being used by the Sponsor in future research related to the study vaccines or the development COVID-19 vaccines in general.

Stored samples will be de-identified and logged into a secure database.

Long term storage of the samples will occur with the Sponsor (or designee storage facility).

10.1.5. Key Roles and Study Governance

This study is sponsored by the University of Melbourne (UoM). Decisions related to the study will be made by a group comprising a group comprising the Sponsor's Medical Monitor, the Co-ordinating PI, the PI at Royal Melbourne Hospital, VIDS (for Sentinel Group 1 participants only) and at least two of the five Investigators.

The administrative structure of the study will be as follows:

Table 10 Study Administrative Structure

Function	Responsible Entity
Study Operations Management	Doherty Institute, University of Melbourne (UoM)
Medical Monitoring	Doherty Institute, UoM
Study Master File	Southern Star Research (SSR)
Randomisation Code	Methods and Implementation Support for Clinical and Health research Hub (MISCH), UoM
Data Management	SSR
Clinical Supply Management	Doherty Institute, UoM
Pharmacy Services	Royal Melbourne Hospital Clinical Trials Pharmacy
Quality Assurance Auditing	UoM

Pharmacovigilance	UoM
Biostatistics	MISCH, UoM
Medical Writing	Doherty Institute, UoM
Laboratory Assessments	Doherty Institute, UoM and Melbourne Health Shared Pathology Service
Data Safety Monitoring Board	Doherty Institute, UoM

10.1.6. Safety Oversight

A group a group comprising the Sponsor's Medical Monitor, the Co-ordinating PI, the PI at the Royal Melbourne Hospital, VIDS (for Sentinel Group 1 participants only) and at least two of the five Investigators will meet at the following time points to review AE data and to ensure no stopping rules have been met:

- After Sentinel Group 1 (Lower-dose Group) participants have completed Day 7.
- After Sentinel Group 2 (Intermediate-dose Group) participants have completed Day 7.
- After Sentinel Group 3 (Higher-dose Group) participants have completed Day 7.

The Data Safety Monitoring Board (DSMB) will meet at the following time points to review AE data and to ensure no stopping rules have been met:

- After Cohort 1a to 1f participants in the Expanded Phase have completed Day 7.
- After Cohort 2a to 2f participants in the Expanded Phase have completed Day 7.

10.1.7. Clinical Site Monitoring

Clinical site monitoring is conducted to ensure that the rights and well-being of study participants are protected and that the reported study data are accurate, complete, and verifiable. It also ensures that the study is conducted in compliance with the approved study protocol, ICH GCP and applicable regulatory requirement(s)and sponsor requirements.

This study will be monitored by representatives of Southern Star Research (SSR) in accordance with SSR Standard Operating Procedures at a frequency and level of detail proscribed by the Sponsor. Monitoring visits will include, but are not limited to, review of study files, accountability records, eCRFs, PICFs, medical and laboratory reports, study vaccine storage records, training records and protocol and GCP compliance. Site monitors will have access to each participating site, study personnel and all study documentation according to the Sponsor-approved site monitoring plan. Study monitors will meet with the Principal Investigator and all participating study investigators to discuss any problems and outstanding issues and will document all site visit findings and discussions.

10.1.8. Quality Control (QC) and Quality Assurance (QA)

All participant data relating to the study will be recorded in the eCRF unless transmitted to the Sponsor (or designee) electronically (e.g. laboratory data or electronic diary cards). The PI/delegate is responsible for verifying that data entries are accurate and correct by physically or electronically signing the eCRF.

The PI must maintain accurate documentation (source data) that supports the information entered into the eCRF and permit study-related monitoring, audits, HREC review, and regulatory agency inspections and provide direct access to source data documentation for these purposes.

The Sponsor or designee will be responsible for the data management of this study including quality checking of the data. Study monitors will perform ongoing source data verification to confirm that:

- data entered into the eCRF by authorised study personnel are accurate, complete and verifiable from source documents
- the safety and rights of participants are being protected and
- the study is being conducted in accordance with the currently approved protocol, ICH GCP and all applicable regulatory requirements.

The Sponsor reserves the right to allow for remote monitoring, where necessary. Any remote monitoring will be performed in accordance with relevant standard operating procedures.

10.1.9. Data Handling and Record Keeping

10.1.9.1. Data Collection and Management Responsibilities

Data collection is the responsibility of the study personnel at each study site under the supervision of the Principal Investigator or site investigator. Each study site must maintain complete and accurate source documentation.

All participant data relating to the study will be recorded in the eCRF unless transmitted to Southern Star Research, the Sponsor's designee, electronically (e.g., laboratory data or electronic diary card data). The Investigator is responsible for verifying that data entries are accurate and correct by electronically signing the eCRF.

The investigator must permit study-related monitoring, audits, HREC review and regulatory agency inspections and provide direct access to source data documents.

Southern Star Research is responsible for the data management of this study including quality checking of the data.

Study monitors employed by Southern Star Research will perform ongoing source data verification to confirm that data entered into the eCRF by authorized study site personnel are accurate, complete, and verifiable from source documents; that the safety and rights of participants are being protected; and that the study is being conducted in accordance with the currently approved protocol and any other study agreements, ICH GCP and all applicable regulatory requirements.

The Sponsor reserves the right to allow for remote monitoring in special situations. Any such monitoring will be performed in accordance with Southern Star Research standard operating procedures.

10.1.9.2. Study Record Retention

Records and documents, including signed PICFs pertaining to the conduct of this study must be retained by the Investigator for 15 years after the signing of the final study report unless local regulations or institutional policies require a longer retention period. No records may be destroyed during the retention period without the written approval of the Sponsor. No records may be transferred to another location or party without written notification to the Sponsor.

10.1.9.3. Source Records

The Investigator/study site should maintain adequate and accurate source documents and study records that include all participant observations on each of the study participants.

Source data should be attributable, legible, contemporaneous, original, accurate, and complete. Changes to source data should be traceable, should not obscure the original entry, and should be explained if the reason for the change(s) is not readily apparent (e.g., via an audit trial).

Source documents providence evidence for the existence of the study participant and substantiate the integrity of the data collected. Source documents must be filed at the Investigator's study site.

Data reported in the eCRF or entered into the eCRF that are transcribed from source documents must be consistent with the source documents or the discrepancies must be explained.

10.1.10.Protocol Deviations

A protocol deviation is any non-compliance with the study protocol. The non-compliance may be on the part of the participant, the Investigator or study personnel. Following a deviation(s), corrective actions should be developed by the study site and implemented promptly. All individual participant protocol deviations must be addressed in the participant's source data record.

It is the responsibility of the Principal Investigator, study investigators and personnel to identify and report deviations within a timely manner. Protocol deviations must be notified to the HREC per their guidelines. A completed Protocol Deviation Form must be maintained in the study files, as well as in the participant's source data record if the deviation is participant specific.

10.1.11.Publication and Data Sharing

This study has been registered at www.ClinicalTrials.gov.

A Clinical Study Report (CSR), which complies with ICH-E3 guidelines, will be prepared by the Sponsor or designee on completion of the study and signed by the PI(s).

A peer reviewed publication will be prepared by the Co-ordinating PI for submission to an international journal. Authorship of any publications will be in accordance with International Committee of Medical Journal Editors authorship requirements.

10.2. Abbreviations

ACE	Angiotensin converting enzyme
AE	Adverse Event
AESI	Adverse Event of Special Interest
ANZCTR	Australian and New Zealand Clinical Trial Register
ASC	Antibody Secreting Cells
BMI	Body Mass Index
BP	Blood Pressure
CI	Confidence Interval
CoV	Coronavirus
COVID-19	Coronavirus Disease 2019
CTN	Clinical Trial Notification Scheme
eCRF	Electronic Case Report Form
DSMB	Data Safety Monitoring Board
EDC	Electronic Data Capture
eDiary	Electronic Diary
ELISA	Enzyme-Linked Immunosorbent Assay
ELISpot	ELISA Spot assay
GCP	Good Clinical Practice
GMFR	Geometric Mean Fold Rise
GMT	Geometric Mean Titre
HIPAA	US Health Insurance Portability and Accountability Act 1996
HIV	Human Immunodeficiency Virus
HR	Heart Rate
HREC	Human Ethics Research Committee
IB	Investigator Brochure
ICH	International Council for Harmonisation
ICS	Intracellular Cytokine Staining
IgA	Immunoglobulin A
IgG	Immunoglobulin G
IgM	Immunoglobulin M
IL	Interleukin
IM	Intramuscular
IWRS	Interactive Web Response Systems
LASI	Laboratory Abnormality of Special Interest
LNP	Lipid nanoparticle
MAAE	Medically-Attended Adverse Event
MBC	Memory B cell
MedDRA	Medical Dictionary for Regulatory Activities
MF59®	Proprietary product name for MF59C.1 adjuvant manufactured by Seqirus Inc.
MISCH	Methods and Implementation Support for Clinical and Health research Hub, University of Melbourne
mL	Millilitre
MOP	Manual of Procedures
N	Number (typically refers to participants)
NaCl	Sodium Chloride
NIAID	National Institute of Allergy and Infectious Diseases (USA)
NIH	National Institutes of Health (USA)
NOCMC	New Onset Chronic Medical Condition

PBMC	Peripheral Blood Mononuclear Cell
PCR	Polymerase Chain Reaction
PI	Principal Investigator
PICF	Participant Information Consent Form
PP	Per Protocol
QA	Quality Assurance
QC	Quality Control
RAT	Rapid Antigen Test
RNA	Ribonucleic acid
SAE	Serious Adverse Event
SAP	Statistical Analysis Plan
SARS	Severe Acute Respiratory Syndrome
SARS-CoV	SARS Coronavirus
SARS-CoV-2	SARS Coronavirus-2
SOA	Schedule of Assessments
SUSAR	Suspected Unexpected Serious Adverse Reaction
TGA	Therapeutic Goods Administration
µg	Microgram
VE	Vaccine Efficacy
VIDS	Victorian Infectious Diseases Service
VOC	Variants of Concern
WHO	World Health Organisation
WHODrug	World Health Organisation Drug Dictionary
WT	Wuhan Type (ancestral strain)
WOCBP	Women of Childbearing Potential

10.3. Protocol Amendment History

**Date**	**Version No.**	**Comment**
29 November 2021	1.0	Submitted to RMH HREC on 29 November 2021
03 February 2022	3.1	Approved by RMH HREC on 16 February 2022
30 March 2022	3.2	Amendments to Protocol v3.1 submitted to RMH HREC on 04 April 2022
02 June 2022	3.3	Amendments to Protocol v3.2 submitted to RMH HREC on 02 June 2022
01 August 2022	4	Amendments to Protocol v3.3 submitted to RMH HREC on 01 August 2022
18 August 2022	4.1	Amendment to Protocol v4 submitted to RMH HREC on 18 August 2022

11. REFERENCES

Johns Hopkins University (2021) *COVID-19 Dashboard by the Center for Systems Science and Engineering (CSSE) at Johns Hopkins University (JHU)*, *Johns Hopkins Coronavirus Resource Center*. Available at: https://coronavirus.jhu.edu/ (Accessed: 15 October 2021).

Wang, N. *et al.* (2020) 'Subunit Vaccines Against Emerging Pathogenic Human Coronaviruses', *Frontiers in Microbiology*, 11, p. 298. doi:10.3389/fmicb.2020.00298.

Piccoli, L. *et al.* (2020) 'Mapping Neutralizing and Immunodominant Sites on the SARS-CoV-2 Spike Receptor-Binding Domain by Structure-Guided High-Resolution Serology', *Cell*, 183(4), pp. 1024-1042.e21. doi:10.1016/j.cell.2020.09.037.

Liao, Y. et al. (2021) 'Safety and immunogenicity of a recombinant interferon-armed RBD dimer vaccine (V-01) for COVID-19 in healthy adults: a randomized, double-blind, placebo-controlled, Phase I trial', Emerging Microbes & Infections, 10(1), pp. 1589–1597. doi:10.1080/22221751.2021.1951126.

12. LIST OF TABLES

Table 1: Primary Objectives and Endpoints
Table 2: Secondary Objectives and Endpoints
Table 3: Exploratory Objectives and Endpoints
Table 4: Listed of Solicited AEs
Table 5: Severity Grading for Solicited Local and Systemic AEs
Table 6: Severity Grading for Unsolicited AEs
Table 7: Probability of observing events by vaccine dose-group* in the Dose-Escalation and Expanded Phase (n=8 participants)
Table 8: Probability of observing events by vaccine group* in the Dose-Escalation and Expanded Phase (n=24 participants)
Table 9: Analysis Populations
Table 10: Study Administrative Structure
Table 11: Protocol-specified Laboratory Assessments
Table 12: Thresholds for Laboratory Abnormalities

13. LIST OF FIGURES

Section 1.2.1 Treatment Groups
Section 1.2.2 Study Overview
Section 1.2.3 Randomisation Overview
Section 1.3 Schedule of Activities: Dose-Escalation Phase and Expanded Phase

14. APPENDICES

APPENDIX 1

The tests detailed in Table 11 will be performed by Melbourne Health Shared Pathology services (safety evaluations) and the Doherty Institute, University of Melbourne.

Additional safety laboratory assessments may be performed at any time during the study if deemed necessary in the Investigator's clinical judgment.

Table 11 Protocol-specified Laboratory Assessments

**Laboratory Assessments**	**Parameters**
Haematology	• White blood cell count<br>• Red blood cell count<br>• Platelet count<br>• Haemoglobin<br>• White cell differential count (neutrophils, lymphocytes, monocytes, eosinophils, basophils)<br>• Mean corpuscular volume (MCV)<br>• Mean corpuscular haemoglobin (MCH)<br>• Haematocrit (HCT)<br>• Mean corpuscular haemoglobin concentration (MCHC)<br>• Red cell distribution width (RDW)<br>• Mean platelet volume (MPV)<br>• Activated partial thromboplastin time (APPT)<br>• Prothrombin time (PT)<br>• INR
Clinical Chemistry	• Alanine aminotransferase (ALT)<br>• Aspartate Aminotransferase (AST)<br>• Gamma glutamyl transferase (GGT)<br>• Alkaline phosphatase (ALP)<br>• Total bilirubin<br>• Creatinine<br>• Urea<br>• Electrolytes (bicarbonate, sodium, potassium and chloride)<br>• Glucose (random)<br>• Rheumatoid factor (IgM anti-Fc)
Viral serology	• Hepatitis B surface antigen<br>• Hepatitis C antibody<br>• Human immunodeficiency virus antibody (types 1 and 2)

Urine Dipstick	• Glucose<br>• Protein<br>• Blood<br>• Leucocytes
Urinalysis (If urine dipstick results are abnormal, i.e. contain more than trace abnormalities)	• pH<br>• Specific gravity<br>• Protein<br>• Glucose<br>• Bilirubin<br>• Blood<br>• Nitrites<br>• Urobilinogen<br>• Leucocytes
Other	• Highly sensitive urine human chorionic gonadotropin (hCG) pregnancy test
Immunogenicity tests	• Anti-SARS-CoV-2 neutralising antibody (ACE-2-RBD protein-based)<br>• Anti-SARS-CoV-2 neutralising antibody (cell-based microneutralisation)<br>• Anti-SARS-CoV-2 neutralising antibody (pseudo-virus)<br>• Antigen specific T-cell immune response<br>• ELISA antibody binding assay<br>• Multiplex bead antibody binding assay<br>• Multiplex bead RBD-ACE2 antibody inhibition assay<br>• ELISpot T cell cytokine assay<br>• T-cell immune response balance (Th1 and Th2)<br>• Memory B cell sequencing clonotype analysis<br>• Serum antibody clonotype analysis

APPENDIX 2

Table 12: Thresholds for Laboratory Abnormalities

Endpoint	Mild (Grade 1)	Moderate (Grade 2)	Severe (Grade 3)
**Above normal WBC – cell/mm^3**	10,800 – 15,000	15,001 – 20,000	≥ 20,001
**Below normal WBC – cell/mm^3**	2,500 – 3,500	1,500 – 2,499	≤ 1,499
**Hemoglobin (Female) – gm/dL**	11.0 – 11.4	9.5 – 10.9	≤ 9.4
**Hemoglobin (Female) (Change from baseline value) – gm/dL**	Any decrease – 1.5	1.6 – 2.0	≥ 2.1
**Hemoglobin (Male) – gm/dL**	12.5 – 13.0	10.5 – 12.4	< 10.5
**Hemoglobin (Male) (change from baseline value) – gm/dL**	Any decrease – 1.5	1.6 – 2.0	≥ 2.1
**Platelets decreased – cell/mm^3**	125,000 – 140,000	100,000 – 124,000	≤ 99,000
**Absolute neutrophil decrease – cell/mm^3**	1,500 – 2,000	1,000 – 1,499	< 999
**Absolute lymphocyte decrease – cell/mm^3**	750 – 1,000	500 – 749	< 499
**Absolute eosinophil – cell/mm^3**	650 – 1500	1501 - 5000	> 5000
**Sodium – Hyponatremia – mEq/L**	132 – 134	130 – 131	< 130
**Sodium – Hypernatremia – mEq/L**	144 – 145	146 – 147	> 148
**Potassium – Hyperkalemia – mEq/L**	5.1 – 5.2	5.3 – 5.4	> 5.4
**Potassium – Hypokalemia – mEq/L**	3.4 – 3.5	3.2 – 3.3	< 3.3
**Blood Urea Nitrogen (BUN) – mg/dL**	23 – 26	27 – 31	> 31 or require dialysis
**Creatinine – mg/dL**	1.5 – 1.7	1.8 – 2.0	≥ 2.1 or requires dialysis
**Glucose – Hyperglycemia (random) – mg/dL**	110 - 125	126 - 200	> 200
**Total protein – Hypoproteinemia – g/dL**	5.5 – 6.0	5.0 – 5.4	< 5.0
**LFT (ALT, AST) (Increase by factor)**	1.1 – 2.5 x ULN	2.6 – 5.0 x ULN	≥ 5.1 ULN
**Alkaline phosphate (Increase by factor)**	1.1 – 2.0 x ULN	2.1 – 3.0 x ULN	≥ 3.1 ULN
**Bilirubin – with any increase in LFT (Increase by factor)**	1.1 – 1.25 x ULN	1.26 – 1.5 x ULN	≥ 1.51 ULN
**Bilirubin – with normal LFT (Increase by factor)**	1.1 – 1.5 x ULN	1.6 – 2.0 x ULN	≥ 2.1 ULN

**Endpoint**	**Mild (Grade 1)**	**Moderate (Grade 2)**	**Severe (Grade 3)**
**PT – increase by factor (prothrombin time)**	1.05 – 1.10 x ULN	1.11 – 1.20 x ULN	1.21 – 1.25 x ULN
**PTT – increase by factor (partial thromboplastin time)**	1.05 – 1.2 x ULN	1.21 – 1.4 x ULN	1.41 – 1.5 x ULN
**Urine - glucose**	Trace	1+	2+
**Urine - protein**	Trace	1+	2+
**Urine – blood (microscopic - RBCs per high power field (rbc/hpf)**	1 - 10	11 - 50	> 50 and/or gross blood

Abbreviations: ALT, alanine aminotransferase; AST, aspartate aminotransferase; dL, deciliter; gm, gram; L, liter; LFT, liver function test; mEq, milliequivalent; mg, milligram; mm^3, cubic millimetre; PT, prothrombin time; PTT,partial thromboplastin time; RBC, red blood cell; ULN, upper limit of normal; WBC, white blood cell.

APPENDIX 3

CO-ORDINATING PRINCIPAL INVESTIGATOR'S SIGNATURE PAGE

I confirm I have read the protocol entitled "*A Phase I, randomised, double-blind, placebo-controlled, dose-escalation study of adjuvanted SARS-CoV-2 beta variant receptor-binding domain recombinant protein (DoCo-Pro-RBD-1 + MF59®) and mRNA (MIPSCo-mRNA-RBD-1) vaccines in healthy adults aged 18 to 64 years previously vaccinated with 3 doses of licensed SARS-CoV-2 ancestral strain vaccines"*, Version 4.1 dated 17 August 2022 in its entirety and agree to conduct the study accordingly.

I also agree to perform this study in accordance with the ethical principles that have their origin in the Declaration of Helsinki, the International Conference on Harmonization (ICH) guideline for Good Clinical Practice (GCP), and the applicable regulatory requirement(s).

I will also appropriately direct and assist the personnel at the study site who will be involved in the conduct of the study.

Investigator's Name	Professor Terry Nolan
Institution Name	Vaccine and Immunisation Research Group, Doherty Institute
Signature with date	

PRINCIPAL INVESTIGATOR'S SIGNATURE PAGE

I confirm I have read the protocol entitled "*A Phase I, randomised, double-blind, placebo-controlled, dose-escalation study of adjuvanted SARS-CoV-2 beta variant receptor-binding domain recombinant protein (DoCo-Pro-RBD-1 + MF59®) and mRNA (MIPSCo-mRNA-RBD-1) vaccines in healthy adults aged 18 to 64 years previously vaccinated with 3 doses of licensed SARS-CoV-2 ancestral strain vaccines.*", Version 4.1 dated 17 August 2022 in its entirety and agree to conduct the study accordingly.

I also agree to perform this study in accordance with the ethical principles that have their origin in the Declaration of Helsinki, the International Conference on Harmonization (ICH) guideline for Good Clinical Practice (GCP), and the applicable regulatory requirement(s).

I will also appropriately direct and assist the personnel at the study site who will be involved in the conduct of the study.

Investigator's Name	Associate Professor Joseph Sasadeusz
Institution Name	Victorian Infectious Disease Service (VIDS), Royal Melbourne Hospital
Signature with date	
